# Enantioselective palladium-catalyzed diboration of 1,1-disubstituted allenes†
†Electronic supplementary information (ESI) available. CCDC 1517472 and 1517578. For ESI and crystallographic data in CIF or other electronic format see DOI: 10.1039/c7sc01254c



**DOI:** 10.1039/c7sc01254c

**Published:** 2017-05-16

**Authors:** Jiawang Liu, Ming Nie, Qinghai Zhou, Shen Gao, Wenhao Jiang, Lung Wa Chung, Wenjun Tang, Kuiling Ding

**Affiliations:** a State Key Laboratory of Organometallic Chemistry , State Key Laboratory of Bio-Organic and Natural Products Chemistry , Shanghai Institute of Organic Chemistry , Chinese Academy of Sciences , 345 Ling Ling Rd , Shanghai 200032 , China . Email: tangwenjun@sioc.ac.cn ; Email: kding@sioc.ac.cn; b Department of Chemistry , South University of Science and Technology of China , Shenzhen 518055 , China . Email: oscarchung@sustc.edu.cn; c University of Chinese Academy of Sciences , Beijing 100049 , China; d Collaborative Innovation Center of Chemical Science and Engineering , Tianjin 300071 , China

## Abstract

A practical and enantioselective palladium-catalyzed diboration of 1,1-disubstituted allenes is developed by employing a P-chiral monophosphorus ligand, BI-DIME. A series of diboronic esters containing a chiral tertiary boronic ester moiety are formed in excellent yields and ee’s with the palladium loading as low as 0.2 mol%. DFT calculations revealed a concerted mechanism of oxidative addition of bis(pinacolato)diboron and allene insertion, as well as a critical dispersion effect on the origins of the enantioselectivity. The method is successfully applied to the concise and enantioselective synthesis of brassinazole.

## Abstract 2

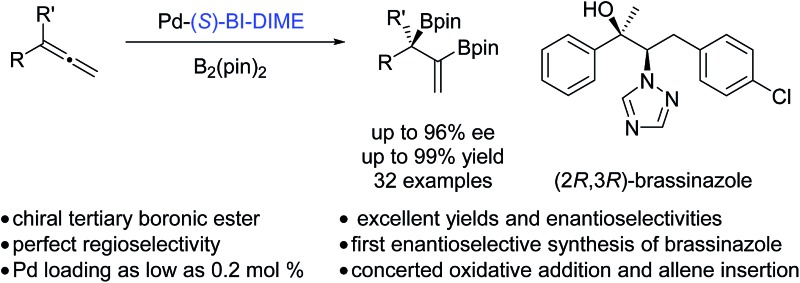
Enantioselective palladium-catalyzed diboration of 1,1-disubstituted allenes is developed for the first time by employing a P-chiral monophosphorus ligand, BI-DIME.

## 

Chiral boronic esters have become versatile building blocks in synthetic organic chemistry.^1^ The synthesis of chiral tertiary boronic esters has attracted considerable interest recently and some notable methods have been developed (Fig. 1), including the Aggarwal–Matteson lithiation–borylation methodology from chiral secondary alcohols,^2^ asymmetric hydroboration of 1,1-disubstituted alkenes,^3^ and asymmetric borylation of allylic carbonates^4^ and Michael acceptors,^5^ as well as tertiary halides.^6^ The asymmetric diboration of 1,1-disubstituted alkenes or allenes^7^ would not only provide a chiral tertiary boronic ester moiety, but also form an additional boronic ester component for further transformations. Work by Morken on asymmetric diboration^8,9^ with various transition metal catalysts (Rh, Pt, or Pd) has provided significant progress in forming secondary boronic ester products with excellent ee’s. However, the asymmetric diboration of 1,1-disubstituted alkenes or allenes to form chiral tertiary boronic esters remains challenging, with either low ee’s or low yields.^9*b*^ Herein we communicate our results on palladium-catalyzed asymmetric diboration of 1,1-disubstituted allenes that have led to a series of diboronic esters containing a chiral tertiary boronic ester moiety in excellent enantioselectivities and yields with the employment of a P-chiral monophosphorus ligand BI-DIME. The chiral diboronic ester products are useful chiral building blocks, and have led to the first enantioselective synthesis of a specific brassinosteroid biosynthetic inhibitor—brassinazole.^19^

**Fig. 1:**
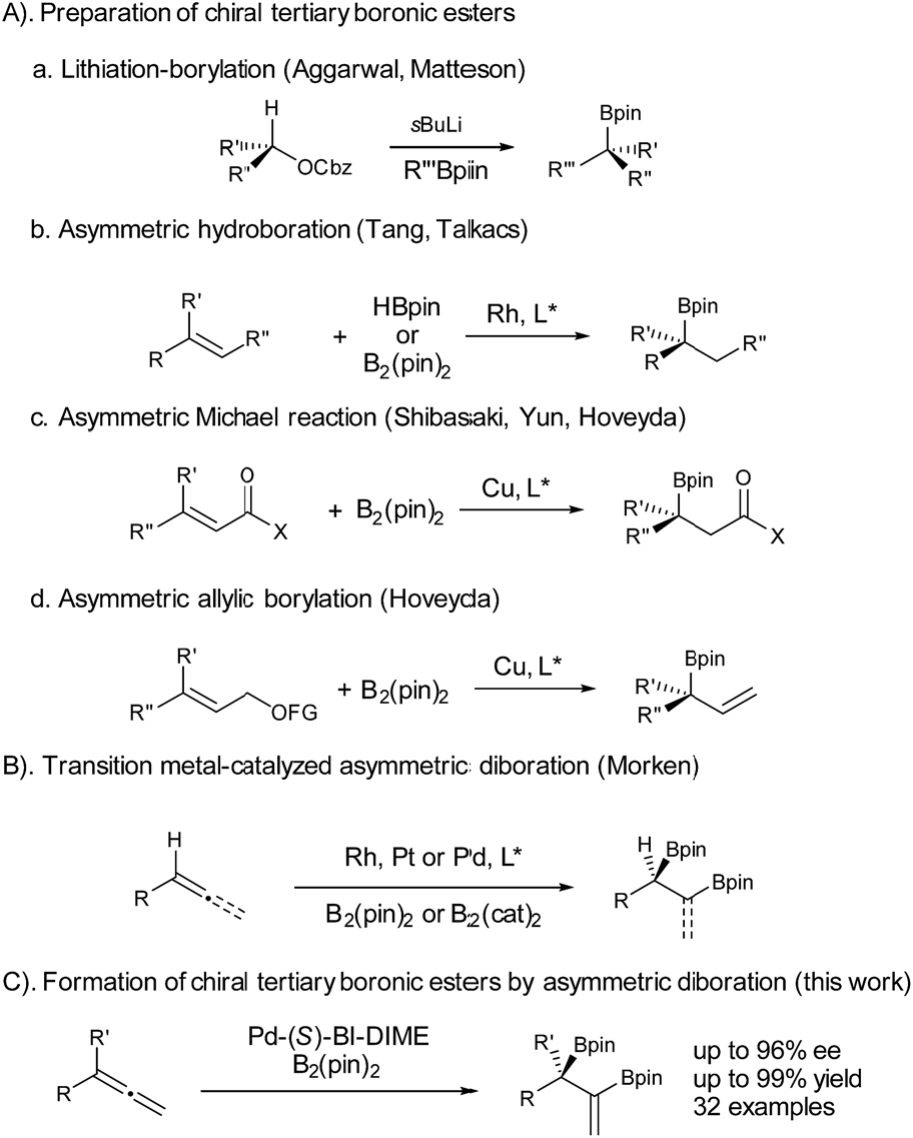
Formation of chiral tertiary boronic esters by asymmetric diboration.

We chose to use buta-2,3-dien-2-ylbenzene (**1a**) as the substrate to investigate the palladium-catalyzed asymmetric diboration of 1,1-disubstituted allenes (Table 1). The reactions were carried out at rt in cyclohexane for 24 h with bis(pinacolato)diboron as the reagent in the presence of Pd_2_(dba)_3_ (1 mol%) and a chiral phosphorus ligand (2.5 mol%) (Table 1). It was found that chelating phosphorus ligands such as BINAP and SDP did not provide any reactivity (entries 1–2).

**Table 1:** Enantioselective Pd-catalyzed diboration of buta-2,3-dien-2-ylbenzene (**1a**) with bis(pinacolato)diboron (**2**)

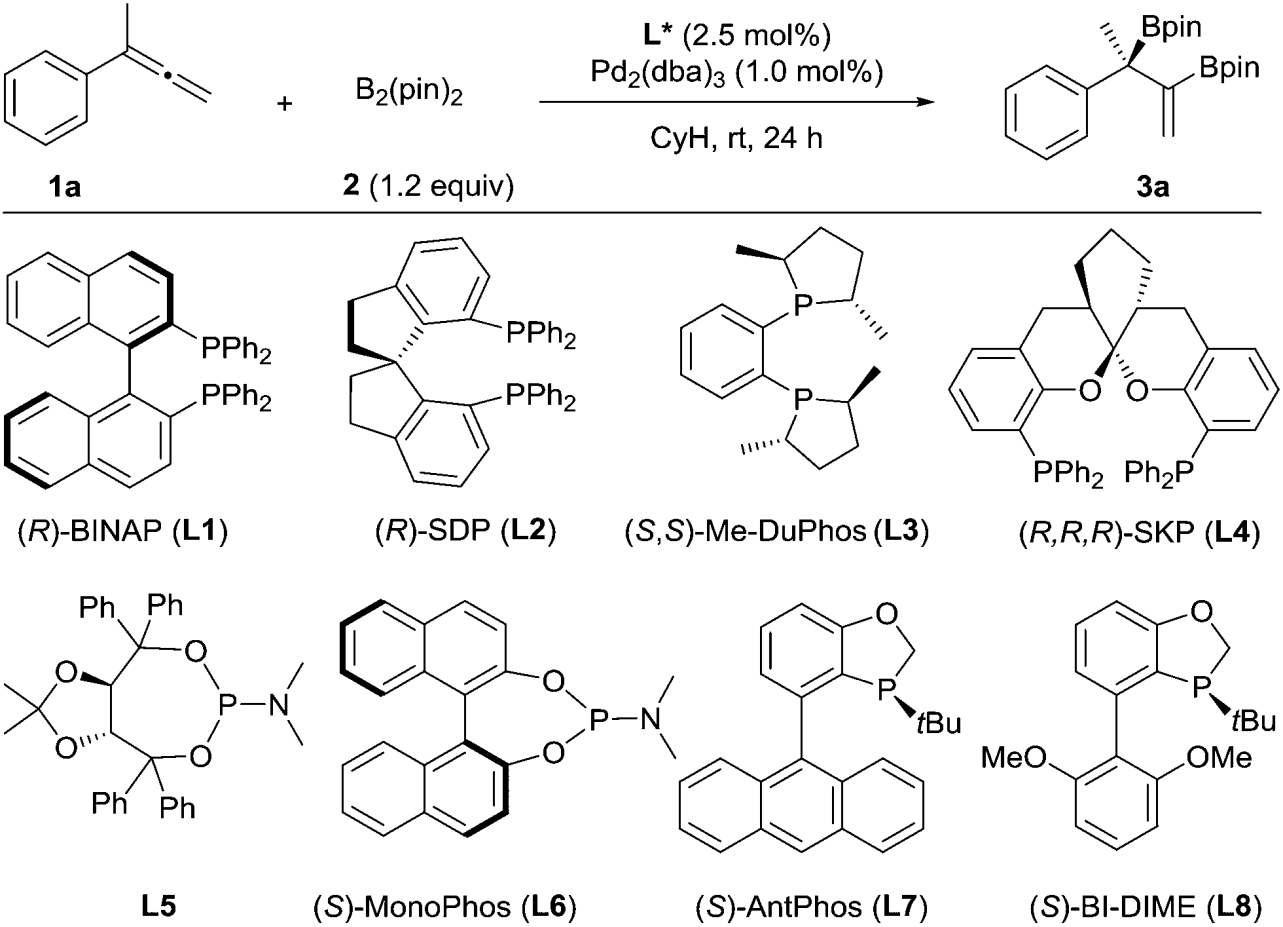				
Entry^*a*^	L*	Solvent	Yield^*b*^ (%)	ee^*c*^ (%)
1	**L1**	CyH	0	—
2	**L2**	CyH	0	—
3	**L3**	CyH	15	52
4	**L4**	CyH	95	59
5	**L5**	CyH	76	79
6	**L6**	CyH	45	12
7	**L7**	CyH	60	91
8	**L8**	CyH	98	94
9	**L8**	Toluene	82	92
10	**L8**	THF	51	91
11	**L8**	Dioxane	27	92
12	**L8**	DCM	0	—
13^*d*^	**L8**	CyH	33	92
14^*e*^	**L8**	CyH	97	94

^*a*^Unless otherwise specified, the reactions were performed under nitrogen at rt for 24 h with **1a** (0.20 mmol), **2** (0.24 mmol), L* (2.5 mol%) and Pd_2_(dba)_3_ (1.0 mol%) in the specified solvent. Product **3a** was the only detectable product. The *R* absolute configuration of **3a** was determined by comparing its optical rotation with reported data.^14^

^*b*^Isolated yields.

^*c*^Determined by HPLC on a chiral IC-3 column.

^*d*^Pd(OAc)_2_ instead of Pd_2_(dba)_3_ was employed as the precursor.

^*e*^ **1a** (36.0 mmol), Pd_2_(dba)_3_ (0.1 mmol%), **L8** (0.25 mmol%), 72 h.

A low yield (15%) and ee (52% ee) were observed when Me-DuPhos was employed as the ligand (entry 3). It should be noted that the diboration occurred exclusively on the substituted double bond of the allene, providing product **3a**, which contains both a tertiary boronic ester moiety and an alkenyl boronic ester moiety. Interestingly, the SKP ligand^10^ (**L4**) with a large bite angle led to an excellent yield (95%) and a moderate ee (59% ee) (entry 4). We thus predicted that the reaction could be better promoted with a monophosphorus ligand, as observed by Morken in the diboration of monosubstituted allenes.^9^ Thus, a TADDOL-derived monophosphoramidite ligand, **L5**, led to the formation of **3a** in 76% yield and 79% ee (entry 5). Another monophosphoramidite ligand, **L6**, derived from a chiral BINOL backbone, proved to be less effective, indicating the importance of the ligand scaffold for both the reactivity and enantioselectivity of the reaction. Encouragingly, the P-chiral monophosphorus ligand AntPhos^11^ (**L7**) provided a moderate yield (60%) and an excellent ee (91% ee) (entry 7). Further study of the P-chiral phosphorus ligands developed in our laboratory showed that BI-DIME^12^ (**L8**) provided an almost quantitative yield and the highest ee (94%) (entry 8). Screening of the solvent showed that the reaction was facilitated with a nonpolar and non-coordinating solvent, as a diminished yield was observed in toluene, THF or dioxane. No reaction was observed when dichloromethane was employed (entries 8–12). A Pd(0) precursor appeared to be advantageous for the reaction since a diminished yield was observed when Pd(OAc)_2_ was applied (entry 13). Finally, the diboration was studied at a low catalytic loading (0.2 mol% Pd, 0.25 mol% **L8**) and at a gram scale (36 mmol **1a**, 4.8 g). The product, **3a** (13.6 g) was obtained in 97% yield and in 94% ee (entry 14), demonstrating the practicality of this asymmetric transformation.

The substrate scope of this asymmetric diboration was then investigated. As depicted in Table 2, a series of diboronic esters with various electronic properties and substitution patterns on the benzene ring (**3b–p**) were smoothly formed at rt in excellent ee’s and yields with Pd-**L8** as the catalyst. The enantioselectivities obtained were slightly higher with substrates having electron-donating substituents, but substituents such as fluoro groups (**3d**) and trifluoromethyl groups (**3g**) were well applicable. Substrates with an *ortho* substituent (**3n–p**) were also tolerable. The reactions of both 1- and 2-naphthyl substrates provided excellent yields and ee’s (**3q–r**). A chiral furyl product, **3s**, was also synthesized successfully. Substrates with multiple substituents on the benzene ring were equally effective for the transformation (**3t–u**). In order to test the chemoselectivity between an allene and an olefin, a substrate containing both moieties was subjected to diboration and only the allene moiety was reactive under the current conditions, forming product **3v** in 99% yield and 96% ee. Both cyclic and heterocyclic substrates were applicable to smoothly afford **3w** and **3x**, respectively, in excellent yields and ee’s. Switching the methyl substituent on the allene with an ethyl group resulted in an inferior ee (72% ee, **3y**). The introduction of a cyclopropyl group instead of the methyl substituent was less effective (**3z**). 1,1-Dialkylallenes were also applicable. While a moderate ee (67%) was obtained for product **3aa**, bearing two primary alkyl substituents on the quaternary stereocenter, high ee’s (87% and 91%) were obtained for products **3ab** and **3ac**, respectively, which contain both a methyl and a secondary alkyl group at the chiral center. A good ee was also achieved for **3ad**, bearing a tertiary alkyl group. Finally, the diboration of 1,1-diarylallenes was studied. While a low ee was obtained for product **3ae**, indicating little difference between the phenyl substituent and the *para*-tolyl group, a moderate ee (75%) was achieved for product **3af**, bearing both a phenyl and an *ortho*-tolyl substituent at the quaternary stereocenter.

**Table 2:** Enantioselective Pd-catalyzed diboration of 1,1′-disubstituted allenes^*a*^

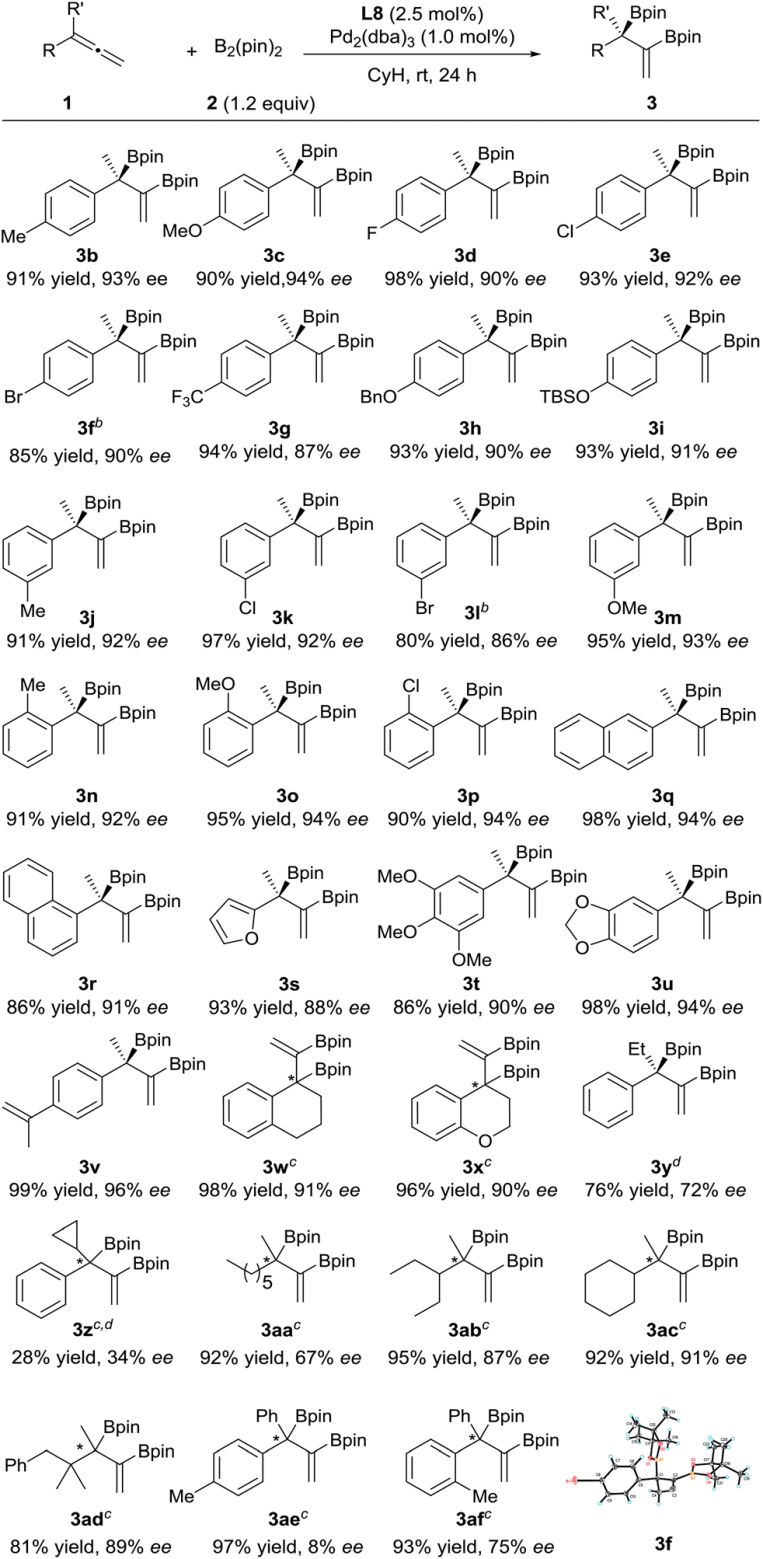

^*a*^Unless otherwise specified, the reactions were performed under nitrogen at rt for 24 h with **1** (0.20 mmol), **2** (0.24 mmol), **L8** (2.5 mol%) and Pd_2_(dba)_3_ (1.0 mol%) in cyclohexane (2.0 mL). The yields of the isolated products are shown here. The ee values were determined by HPLC on a chiral column. The *R* absolute configuration of **3f** was determined by X-ray crystallographic analysis;^13^ the others were assigned by analogy.

^*b*^Pd_2_(dba)_3_ (2.0 mol%) and **L8** (5.0 mol%) were employed.

^*c*^The absolute configurations were not determined.

^*d*^Incomplete conversions.

To understand the mechanism of this catalytic asymmetric reaction, the diboration of **1a** was investigated with a scalemic mixture of ligand **L8**. A perfect linear relationship between the ee of **L8** and the ee of product **3a** was observed, indicating that the reaction was catalyzed by a palladium catalyst composed of a single monophosphorus ligand, **L8**. Variation of the Pd : **L8** ratio from 1 : 1, 1 : 2, and 2 : 1 did not lead to a significant change in either the yield or the enantioselectivity, further demonstrating the presence of a single **L8** in the active palladium catalyst. To understand the perfect regioselectivity and the stereochemical model of this asymmetric diboration, the energetics of the catalytic transformation were calculated at the B3LYP-D3/6-31G(d)+SDD level. As shown in Fig. 2a, the reaction initiates from the palladium species **RC**, which undergoes oxidative addition of bis(pinacolato)diboron concerted with allene insertion, where the boryl group migrates to the middle carbon of the allene to give an η^3^ Pd(ii)-allyl intermediate, **INT_Si_** or **INT_Re_**. Notably, this initial oxidative boryl migration^16^ is in good agreement with the perfect regioselectivity of diboration on the internal double bond of the allene, and this process is an irreversible and stereo-determining step of the transformation.^15^ **TS1_Re_** is computed to be lower in free energy than **TS1_Si_** by 1.4 kcal mol^–1^, which is in qualitative agreement with the observed enantioselectivity (Δ*G*_exp_: 2.1 kcal mol^–1^).

**Fig. 2:**
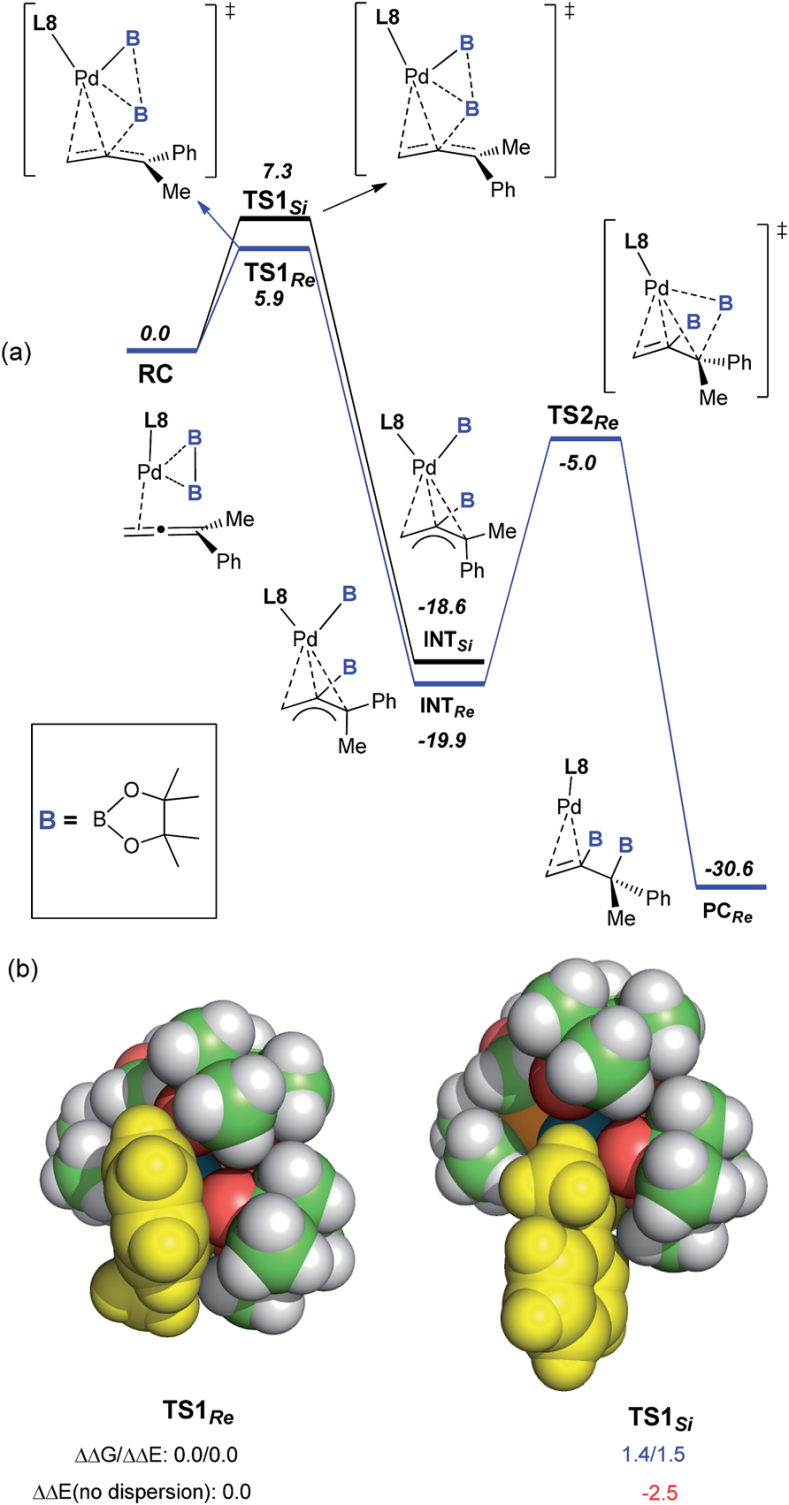
(a) Free-energy profile (in kcal mol^–1^) for the Pd-catalyzed asymmetric diboration of buta-2,3-dien-2-ylbenzene (**1a**) with (*S*)-BI-DME (**L8**) as the ligand at the B3LYP-D3/6-31G(d)+SDD level.^15^ (b) VdW representation of the optimized **TS1_Re_** and **TS1_Si_**, with relative free and electronic energies in kcal mol^–1^.

Distortion/interaction analysis^17^ reveals that there is a greater dispersion interaction between the phenyl group^17*b*^ on the allene and a boryl group in **TS1_Re_** than in **TS1_Si_**, which is considered as the key factor for the enantioselectivity (Fig. 2b).^15^ When excluding the dispersion contribution, the enantioselectivity is computed to be reversed. Finally, the reductive elimination *via* **TS2_Re_** proceeds from **INT_Re_** to produce **PC_Re_**, with a barrier of about 14.9 kcal mol^–1^. In contrast to Morken’s proposal that the oxidative addition of diboron to Pd proceeded prior to migratory insertion and that the oxidative addition of diboron to Pd was computed as the rate-determining step,^9*l*^ our calculations on the diboration of **1a** revealed a concerted mechanism of oxidative addition of bis(pinacolato)diboron and allene insertion, provided the first computational insight into the origins of the enantioselectivity (molecular details and a critical dispersion effect), and disclosed the final reductive elimination step as the rate-determining step.

Chiral diboronic ester products are versatile building blocks in organic synthesis. For example, Aggarwal reported a stereospecific allylation between the diboronic ester (*S*)-**3a** and benzaldehyde to form a tetrasubstituted alkene after a Suzuki–Miyaura cross-coupling.^14,18,9k^ To further explore the synthetic applications of such chiral diboronic esters, an enantioselective synthesis of brassinazole,^19^ a specific inhibitor of brassinosteroid biosynthesis, was studied with (*R*)-**3a** as the starting material (Scheme 1). Surprisingly, despite its significant biological properties, its enantioselective synthesis had not been reported to our knowledge. We envisioned that the diboration product (*R*)-**3a** would provide rapid and efficient access to brassinazole through simple transformations. Thus, (*R*)-**3a** was subjected to a one-pot, three step sequence: (a) hydroboration by treatment with 9-BBN;^20^ (b) Suzuki–Miyaura coupling with 1-chloro-4-iodobenzene; and (c) oxidation under conditions of H_2_O_2_/NaOH. The chiral diol **4** was formed smoothly with a *cis*/*trans* ratio of 4 : 1 in 79% overall yield. Under Ley’s conditions,^21^ diol **4** was readily oxidized to form the corresponding sulfate, **5**, which was isolated as a pure *cis* product in 78% yield. Finally, treatment of sulfate **5** with 1,2,4-triazole under conditions of NaH/DMF yielded (2*R*,3*R*)-brassinazole (**6**) in 80% yield. The absolute configuration was confirmed by X-ray crystallography.^13^ It should be noted that sulfate **5** proceeded first through an intramolecular S_N_2 reaction to form an epoxide, which was subsequently attacked by 1,2,4-triazole to undergo a 2^nd^ S_N_2 reaction, yielding product **6** with net retention of stereochemistry. Compound **4** was also transformed to its diastereomer **7** through an oxidation–reduction procedure, which ultimately led to the formation of (2*R*,3*S*)-brassinazole (**8**) *via* sulfate formation and nucleophilic substitution. Thus, we accomplished a concise and first enantioselective synthesis of brassinazole.

**Scheme 1:**
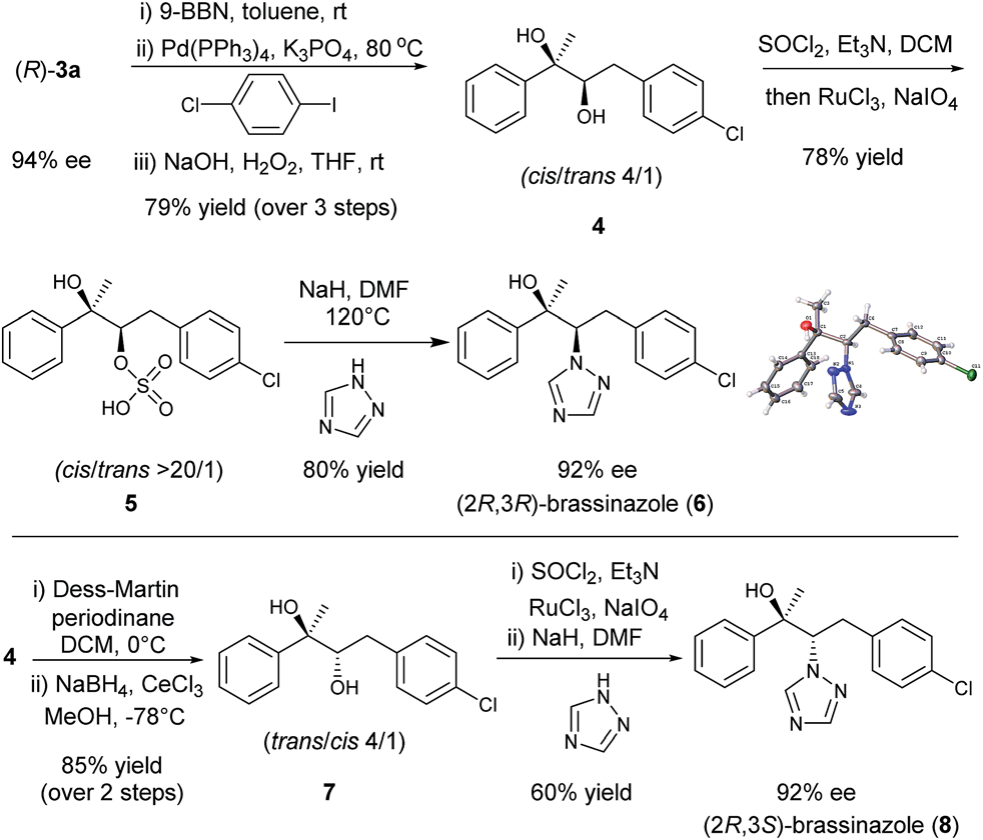
Asymmetric synthesis of brassinazole.

## Conclusions

In summary, we have developed the practical and enantioselective palladium-catalyzed diboration of 1,1-disubstituted allenes. This has led to the synthesis of a series of diboronic esters containing a chiral tertiary boronic ester with excellent yields and enantioselectivities, with the palladium loading as low as 0.2 mol%. The reaction enjoys a broad substrate scope and good functional group compatibility. The chiral ligand BI-DIME has proven to be crucial for the success of the reaction. DFT calculations identified a concerted mechanism of oxidative addition of bis(pinacolato)diboron and allene insertion, and revealed a critical dispersion effect on the origins of the enantioselectivity. Finally, the application of the chiral diboronic ester to a concise and first enantioselective synthesis of brassinazole has been successfully demonstrated.
