# Supplementary material for: Enantioselective palladium-catalyzed diboration of 1,1-disubstituted allenes
Source: Chem Sci. 2017 May 16;8(7):5161–5. doi: 10.1039/c7sc01254c (PMC5615263; doi:10.1039/c7sc01254c)
Supplement: Supplementary file 1 [file SC-008-C7SC01254C-s001.pdf]

Electronic Supplementary Material (ESI) for Chemical Science.
This journal is © The Royal Society of Chemistry 2017

Supporting Information

for

Enantioselective Palladium-Catalyzed Diboration of 1,1-Disubstituted Allenes

Jiawang Liu,§ Ming Nie,§ Qinghai Zhou, Shen Gao, Wenhao Jiang, Lung Wa Chung,* Wenjun Tang,* Kuiling Ding*

State Key Laboratory of Organometallic Chemistry and State Key Laboratory of Bioorganic & Natural Products Chemistry, Shanghai Institute of Organic Chemistry, Chinese Academy of Sciences, 345 Lingling Road, Shanghai, China, 200032

Department of Chemistry, South University of Science and Technology of China, Shenzhen, China, 518055

E-mail: tangwenjun@sioc.ac.cn; kding@sioc.ac.cn; oscarchung@sustc.edu.cn

Table of contents

1. **General information and materials……………………………..…………..SI-2**
2. **Synthetic procedures for chiral ligands….………….……….…………….……..SI-2**
3. **General procedures for the synthesis of 1,1-disubstituted allenes……...…SI-3**
4. **General procedures for palladium-catalyzed diboration of 1,1-disubstituted allenes…….………….……….……….……….…………….………….………..SI-16**
5. **Analytical data of chiral diboronate products …………...…………….……SI-23**
6. **Procedure for a gram scale reaction…...……….……...…………….………SI-43**
7. **Computational studies for Pd-catalyzed asymmetric diboration..…..……SI-44**
8. **Asymmetric synthesis of (2*R*, 3*R*)- and (2*R*, 3*S*)-brassinazole……………SI-66**
9. **Nonlinear effect study……………………………………………………SI-72**
10. **References.…………………………………………………..……SI-73**
11. **NMR and HPLC spectra…………………………………………………SI-75**

1. General Information and Materials

Unless otherwise noted, all reactions and manipulations were performed using standard Schlenk techniques or in a glovebox. Anhydrous CH_2Cl_2, DCE, DMF, 1,4-Dioxane, $CHCl_3$ and acetic ether were distilled from CaH_2 under an atmosphere of argon. Anhydrous THF, Et_3N and toluene were distilled from sodium benzophenone ketyl under an atmosphere of argon. Anhydrous PhF and CH_3CN were distilled from P_2O_5 under an atmosphere of argon. Anhydrous MeOH and EtOH were distilled from magnesium turnings under argon atmosphere. Unless otherwise noted, all palladium salts, (*R*)-BINAP, (*R*)-SegPhos, (*R*)-SDP were purchased from commercial sources and were used without further purification.

Melting points were measured on a RY-I apparatus and uncorrected. ^{1}H, ^{13}C, ^{11}B, ^{19}F and ^{31}P NMR spectra were recorded on Agilent 400 MHz, Varian 400 MHz or Agilent 500 MHz spectrometers. Chemical shifts (δ values) were reported in ppm downfield from internal TMS for ^{1}H NMR, $CDCl_3$ for ^{13}C NMR, external CF_3CO_2H for ^{19}F NMR, external $BF_3{\cdot}Et_2O$ for ^{11}B NMR and external 85% H_3PO_4 for ^{31}P NMR respectively. Optical rotations were determined using a Perkin Elmer 341 MC polarimeter or Jasco 1030-P. The IR spectra were measured on a BRUKER TENSOR 27 FT-IR spectrometer. MS was measured on Agilent 5973N (EI) or Agilent 1100 Series LC/MSD (ESI) or Shimadzu LCMS-2010EV, BRUKERDALTONICS APEX III or Agilent Technologies 6224 TOF LC/MS spectrometer (HR-ESI) or Waters GCT CA 176 (HR-EI) mass spectrometers. HPLC analyses were performed on a Jasco 2089 liquid chromatograph or Agilent 1260 Infinity liquid chromatograph. Column chromatography was performed with silica gel (200-300 mesh).

2. Synthetic procedures for chiral ligands

The chiral SKP ligand series **L4a-i** were synthesized according to procedures described in our previous reports.[1]

Racemic and chiral BI-DIME were prepared according to a procedure reported previously in our laboratory.[2] Chiral AntPhos was prepared according to a procedure reported previously in our laboratory.[3] Ligand **L13-23** were also prepared according

to a procedure previously reported in our laboratory.[4]

3. General procedures for the synthesis of 1,1-disubstituted allenes

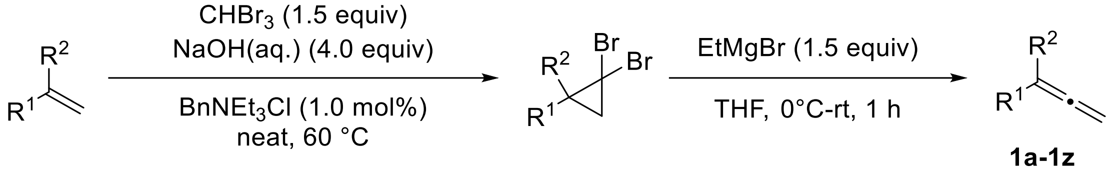

3.1 General Procedures :

The 1,1-disubstituted allenes **1a-1z, 1aa, 1ab, 1ae, 1af** were prepared according to a general procedure reported by Krische and co-workers.[5a]

Preparation of 1,1-Dibromocyclopropanes: 1,1-Dibromocyclopropanes were prepared according to a similar procedure described for (2,2-dibromo-1-methylcyclopropyl)benzene.

(2,2-Dibromo-1-methylcyclopropyl)benzene: To a mixture of 1-methyl-1-phenylethylene (6.5 mL, 50 mmol), bromoform (6.6 mL, 75 mmol), and triethylbenzylammonium chloride (114 mg, 0.5 mmol) was added dropwise a solution of NaOH (8.0 g) in water (8.0 mL) over 1 h. The resulting mixture was stirred vigorously at 60 °C for 24 h, then cooled to room temperature, and quenched with water (50 mL). The mixture was extracted with dichloromethane and the organic phase was separated, dried over sodium sulfate, and concentrated. The residue was purified by column chromatography over silica gel with hexane as eluent to give (2,2-dibromo-1-methylcyclopropyl)benzene (13.7 g, 95% yield) as yellow oil.

Preparation of 1,1-disubstituted allenes: 1,1-Disubstituted allenes were prepared according to a similar procedure described for buta-2,3-dien-2-ylbenzene (**1a**).

Buta-2,3-dien-2-ylbenzene (1a): To a stirred solution of (2,2-dibromo-1-methylcyclopropyl)benzene (13.0 g, 45 mmol) in dry THF (50 mL) at 0 °C was added dropwise ethylmagnesium bromide (67.5 mL, 67.5 mmol, 1.0 M in THF) under nitrogen over 0.5 h. After stirred at 0 °C for 1 h, the mixture was quenched with 3M hydrochloric acid solution (20 mL) and diluted with ethyl ether (200 mL). The organic phase was washed with water (50 mL), dried over magnesium sulfate, and concentrated.

The residue was purified by column chromatography over silica gel with *n*-pentane as eluent to give buta-2,3-dien-2-ylbenzene (**1a**) (5.7 g, 97% yield) as colorless oil.

Preparation of buta-2,3-dien-2-ylcyclohexane (1ac): The allene **1aa** was synthesized according to a procedure reported by Tsuji and co-workers. [5b]

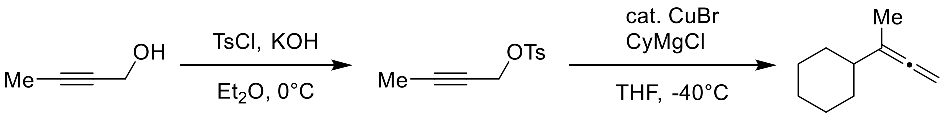

1aa

To a solution of 2-butyn-1-ol (7.5 g, 100 mmol) in diethyl ether (200 mL) at 0 °C was added *p*-toluenesulfonyl chloride (22.9 g, 120 mmol) and crushed KOH (35 g) sequentially. The resulting mixture was stirred at 0 °C for 1 h before it was poured into an ice-water mixture. Ethyl ether (100 mL) was added and the organic phase was separated, dried over magnesium sulfate, and concentrated to give propargyl tosylate as a light yellow oil.

To this oil in a dried 500 mL 3-necked flask was added CuBr (1.4 g, 10 mmol) and THF (200 mL). To the mixture at -40 °C was charged dropwise CyMgCl (2.0 M in ethyl ether, 55 mL, 110 mmol) over 1 h. The resulting mixture was stirred at the same temperature for 3.5 h before it was quenched by saturated NH_4Cl solution (100 mL). Ethyl ether (200 mL) was added and the organic layer was separated, dried over magnesium sulfate, and concentrated. The residue was purified by column chromatography over silica gel with *n*-pentane as eluent to give buta-2,3-dien-2-ylcyclohexane (**1aa**, 8.5 g, 63% yield over 2 steps) as colorless oil.

Preparation of (2,2,3-trimethylpenta-3,4-dien-1-yl)benzene (1ad): The allene **1ad** was synthesized according to a procedure reported by Breit and co-workers. [5l]

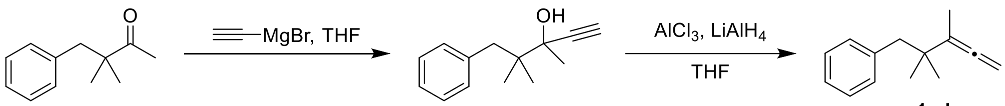

1ad

The 3,3-dimethyl-4-phenylbutan-2-one (7.5 g, 42.6 mmol) was added to a solution of ethynylmagnesium bromide (102 mL, 51.1 mmol, 1.2 equiv) in THF (0.5 M). The

reaction mixture was heated under reflux until ketone was consumed completely as monitored by TLC. After cooling to room temperature, the solution was quenched by the addition of H_2O (5 ml). The crude mixture was concentrated until most of the THF was removed. The residue was dissolved in diethyl ether (200 mL) and saturated NH_4Cl solution (100 mL) was added. The phases were separated and the aqueous layer was extracted with diethyl ether (100 mL *3). The combined organic phases were washed with brine and dried over magnesium sulfate, and concentrated. The residue was purified by column chromatography over silica gel with hexane and ethyl acetate as eluent to give 3,4,4-trimethyl-5-phenylpent-1-yn-3-ol (6.7 g, 78% yield) as slightly yellow oil.

A 3-neck flask equipped with a reflux condenser was charged with $AlCl_3$ (2.4 g, 17.8 mmol, 0.75 equiv) and THF (1 mL / 100 mg $AlCl_3$). When all $AlCl_3$ was suspended the mixture was cooled to 0 ^oC followed by a slow addition of a suspension of $LiAlH_4$ (2.1 g, 53.4 mmol, 2.25 equiv) in THF (1 mL / mmol $LiAlH_4$). After stirring for 15 minutes, 3,4,4-trimethyl-5-phenylpent-1-yn-3-ol (4.8 g, 23.7 mmol, 1.0 equiv) was added as a solution in THF (2 M). The reaction mixture was heated under reflux overnight (15 h). It was cooled to 0 ^oC and quenched by slow addition of H_2O (0.1 mL / mmol alcohol), aq. NaOH (15%, 0.1 mL / mmol alcohol) and additional H_2O (0.3 mL / mmol alcohol). The precipitate was filtered and washed with pentane (3 × 3 mL /mmol alcohol). The combined organic layers were dried over sodium sulfate, concentrated in vacuo, and purified by column chromatography over silica gel with *n*-pentane as eluent to give (2,2,3-trimethylpenta-3,4-dien-1-yl)benzene (**1ad**, 2.2 g, 50% yield) as colorless oil.

3.2 Analytical Data of 1,1-disubstituted allenes

Buta-2,3-dien-2-ylbenzene (1a)[5c]

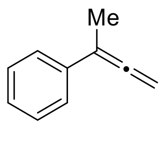

Colorless oil, 97% yield from its 2,2-dibromocyclopropyl precursor. ^{1}H NMR (400

MHz, $CDCl_3$) δ 7.41 (d, *J* = 8.0 Hz, 2H), 7.32 (t, *J* = 8.0 Hz, 2H), 7.19 (t, *J* = 8.0 Hz, 1H), 5.02 (q, *J* = 2.8 Hz, 2H), 2.09 (t, *J* = 2.8 Hz, 3H) ppm; ^{13}C NMR (100 MHz, $CDCl_3$) δ 208.9, 136.6, 128.3, 126.5, 125.6, 99.7, 76.9, 16.6 ppm. The ^{1}H NMR and ^{13}C NMR spectra are in agreement with those reported in the literature.[5c]

1-(Buta-2,3-dien-2-yl)-4-methylbenzene (1b)[5d]

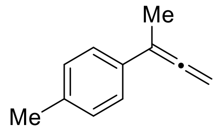

Colorless oil，94% yield from its 2,2-dibromocyclopropyl precursor. ^{1}H NMR (400 MHz, $CDCl_3$) δ 7.30 (t, *J* = 6.0 Hz, 2H), 7.13 (t, *J* = 6.0 Hz, 2H), 5.00 (s, 2H), 2.34-2.33 (m, 3H), 2.09-2.07 (m, 3H) ppm; ^{13}C NMR (100 MHz, $CDCl_3$) δ 208.8, 136.2, 133.7, 129.0, 125.5, 99.6, 76.8, 21.0, 16.7 ppm. The ^{1}H NMR and ^{13}C NMR spectra are in agreement with those reported in the literature.[5d]

1-(Buta-2,3-dien-2-yl)-4-methoxybenzene (1c)

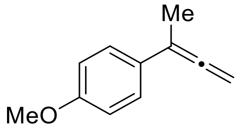

Colorless oil，96% yield from its 2,2-dibromocyclopropyl precursor. ^{1}H NMR (400 MHz, $CDCl_3$) δ 7.35-7.31 (m, 2H), 6.89-6.85 (m, 2H), 5.00 (q, *J* = 2.8 Hz, 2H), 3.80 (s, 3H), 2.07 (t, *J* = 2.8 Hz, 3H) ppm; ^{13}C NMR (100 MHz, $CDCl_3$) δ 208.5, 158.4, 128.9, 126.7, 113.7, 99.2, 76.8, 55.3, 16.8 ppm. The ^{1}H NMR and ^{13}C NMR spectra are in agreement with those reported in the literature.[5a]

1-(Buta-2,3-dien-2-yl)-4-fluorobenzene (1d) [5a]

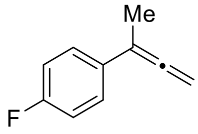

Colorless oil，87% yield from its 2,2-dibromocyclopropyl precursor. ^{1}H NMR (400

MHz, $CDCl_3$) δ 7.37-7.33 (m, 2H), 7.00 (t, J = 8.4 Hz, 2H), 5.01 (d, J = 2.4 Hz, 2H), 2.07 (t, J = 2.4 Hz, 3H) ppm; ^{13}C NMR (100 MHz, $CDCl_3$) δ 208.7 (d, $J_{(F,C)}$ = 1.6 Hz), 161.7 (d, $J_{(F,C)}$ = 244.5 Hz), 132.6 (d, $J_{(F,C)}$ = 3.0 Hz), 127.1 (d, $J_{(F,C)}$ = 8.0 Hz), 115.1 (d, $J_{(F,C)}$ = 21.4 Hz), 99.0, 77.1, 16.8 ppm; ^{19}F NMR (376 MHz, $CDCl_3$) δ -116.4 ppm. The ^{1}H NMR, ^{13}C NMR spectra and ^{19}F NMR are in agreement with those reported in the literature.[5a]

1-(Buta-2,3-dien-2-yl)-4-chlorobenzene (1e) [5d]

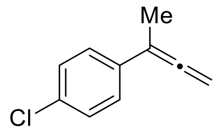

White solid，97% yield from its 2,2-dibromocyclopropyl precursor. ^{1}H NMR (400 MHz, $CDCl_3$) δ 7.33-7.25 (m, 4H), 5.03 (q, J = 3.2 Hz, 2H), 2.07 (t, J = 3.2 Hz, 3H) ppm; ^{13}C NMR (100 MHz, $CDCl_3$) δ 208.9, 135.2, 132.2, 128.3, 126.9, 99.0, 77.3, 16.6 ppm. The ^{1}H NMR and ^{13}C NMR spectra are in agreement with those reported in the literature.[5d]

1-Bromo-4-(buta-2,3-dien-2-yl)benzene (1f) [5e]

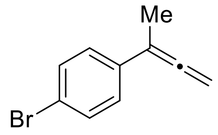

White solid，94% yield from its 2,2-dibromocyclopropyl precursor. ^{1}H NMR (400 MHz, $CDCl_3$) δ 7.43 (d, J = 8.4 Hz, 2H), 7.26 (d, J = 8.0 Hz, 2H), 5.02 (q, J = 2.8 Hz, 2H), 2.06 (t, J = 2.8 Hz, 3H) ppm; ^{13}C NMR (100 MHz, $CDCl_3$) δ 208.8, 135.7, 131.3, 127.2, 120.4, 99.1, 77.4, 16.6 ppm. The ^{1}H NMR and ^{13}C NMR spectra are in agreement with those reported in the literature.[5e]

1-(Buta-2,3-dien-2-yl)-4-(trifluoromethyl)benzene (1g) [5a]

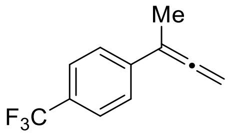

Colorless oil, 94% yield from its 2,2-dibromocyclopropyl precursor. ^{1}H NMR (400 MHz, $CDCl_3$) δ 7.56 (d, J = 8.4 Hz, 2H), 7.49 (d, J = 8.0 Hz, 2H), 5.09-5.08 (m, 2H), 2.10 (t, J = 3.2 Hz, 3H) ppm; ^{13}C NMR (100 MHz, $CDCl_3$) δ 209.5, 140.6, 128.4 (q, $J_{(F,C)}$ = 32.2 Hz), 125.8, 125.1 (q, $J_{(F,C)}$ = 3.8 Hz), 124.4 (q, $J_{(F,C)}$ = 270.7 Hz), 99.1, 77.5, 16.4 ppm; ^{19}F NMR (376 MHz, $CDCl_3$) δ -62.4 ppm. The ^{1}H NMR, ^{13}C NMR spectra and ^{19}F NMR are in agreement with those reported in the literature.[5a]

1-(Benzyloxy)-4-(buta-2,3-dien-2-yl)benzene (1h)

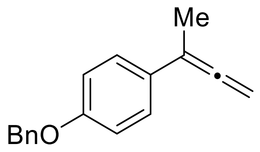

White solid, 96% yield from its 2,2-dibromocyclopropyl precursor. ^{1}H NMR (400 MHz, $CDCl_3$) δ 7.43-7.31 (m, 7H), 6.93 (d, J = 8.8 Hz, 2H), 5.05 (s, 2H), 4.99-4.98 (m, 2H), 2.06 (t, J = 3.2 Hz, 3H) ppm; ^{13}C NMR (100 MHz, $CDCl_3$) δ 208.6, 157.6, 137.0, 129.2, 128.5, 127.9, 127.4, 126.7, 114.7, 99.3, 76.8, 70.0, 16.8 ppm. ESI-MS m/z: 237.1 $[M+H]^+$; HRMS (ESI) m/z: calcd. for $C_{17}H_{17}O^+$: 237.1274, Found: 237.1282 $[M+H]^+$.

(4-(Buta-2,3-dien-2-yl)phenoxy)(tert-butyl)dimethylsilane (1i)

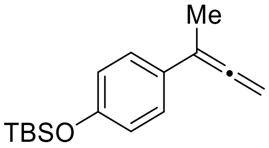

Light yellow oil, 92% yield from its 2,2-dibromocyclopropyl precursor. ^{1}H NMR (400 MHz, $CDCl_3$) δ 7.26 (d, J = 8.8 Hz, 2H), 6.80 (d, J = 8.4 Hz, 2H), 4.98 (q, J = 2.8 Hz, 2H), 2.06 (t, J = 2.8 Hz, 3H), 0.98 (s, 9H), 0.19 (s, 6H) ppm; ^{13}C NMR (100 MHz, $CDCl_3$) δ 208.6, 154.5, 129.5, 126.7, 120.0, 99.4, 76.8, 25.7, 18.2, 16.8, -4.4 ppm. ESI-MS m/z: 261.1 $[M+H]^+$; HRMS (ESI) m/z: calcd. for $C_{16}H_{25}OSi^+$: 261.1669, Found: 261.1679 $[M+H]^+$.

1-(Buta-2,3-dien-2-yl)-3-methylbenzene (1j)

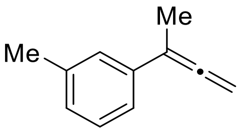

Colorless oil, 87% yield from its 2,2-dibromocyclopropyl precursor. ^{1}H NMR (400 MHz, $CDCl_3$) δ 7.22-7.21 (m, 3H), 7.03-7.01 (m, 1H), 5.01 (q, *J* = 2.8 Hz, 2H), 2.35 (s, 3H), 2.09-2.08 (m, 3H) ppm; ^{13}C NMR (100 MHz, $CDCl_3$) δ 208.9, 137.8, 136.6, 128.2, 127.3, 126.4, 122.7, 99.7, 76.8, 21.5, 16.7 ppm. EI-MS m/z: 144.0 $[M]^+$; HRMS (EI) m/z: calcd. for $C_{11}H_{12}^+$: 144.0934, Found: 144.0941 $[M]^+$.

1-(Buta-2,3-dien-2-yl)-3-chlorobenzene (1k)

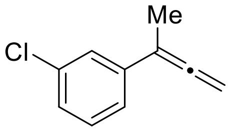

Colorless oil, 97% yield from its 2,2-dibromocyclopropyl precursor. ^{1}H NMR (400 MHz, $CDCl_3$) δ 7.37 (s, 1H), 7.28-7.21 (m, 2H), 7.17-7.15 (m, 1H), 5.05 (q, *J* = 3.2 Hz, 2H), 2.06 (t, *J* = 3.2 Hz, 3H) ppm; ^{13}C NMR (100 MHz, $CDCl_3$) δ 209.0, 138.8, 134.3, 129.4, 126.5, 125.7, 123.7, 99.0, 77.5, 16.5 ppm. EI-MS m/z: 164 $[M]^+$; HRMS (EI) m/z: calcd. for $C_{11}H_{12}^+$: 164.0393, Found: 164.0388 $[M]^+$.

1-Bromo-3-(buta-2,3-dien-2-yl)benzene (1l) [5g]

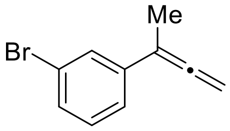

Light yellow oil, 100% yield from its 2,2-dibromocyclopropyl precursor. ^{1}H NMR (400 MHz, $CDCl_3$) δ 7.53 (t, *J* = 2.0 Hz, 1H), 7.33-7.30 (m, 2H), 7.19-7.15 (m, 1H), 5.06 (q, *J* = 3.2 Hz, 2H), 2.06 (t, *J* = 3.2 Hz, 3H) ppm; ^{13}C NMR (100 MHz, $CDCl_3$) δ 209.0, 139.1, 129.7, 129.4, 128.6, 124.2, 122.6, 98.9, 77.5, 16.5 ppm. The ^{1}H NMR and ^{13}C NMR spectra are in agreement with those reported in the literature. [5g]

1-(Buta-2,3-dien-2-yl)-3-methoxybenzene (1m)[5h]

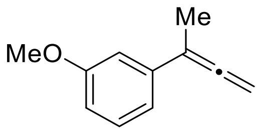

Light yellow oil, 98% yield from its 2,2-dibromocyclopropyl precursor. ^{1}H NMR (400 MHz, $CDCl_3$) δ 7.26-7.22 (m, 1H), 7.05-6.98 (m, 1H), 6.96 (t, *J* = 2.1 Hz, 1H), 6.76 (dd, *J* = 8.2, 2.6 Hz, 1H), 5.02 (q, *J* = 3.2 Hz, 2H), 3.81 (s, 3H), 2.08 (t, *J* = 3.1 Hz, 3H) ppm. The ^{1}H NMR spectra is in agreement with those reported in the literature. [5h]

1-(Buta-2,3-dien-2-yl)-2-methylbenzene (1n) [5i]

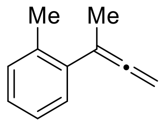

Colorless oil，93% yield from its 2,2-dibromocyclopropyl precursor. ^{1}H NMR (400 MHz, $CDCl_3$) δ 7.23-7.08 (m, 4H), 4.74 (q, *J* = 3.2 Hz, 2H), 2.36 (s, 3H), 2.04 (t, *J* = 3.2 Hz, 3H) ppm; ^{13}C NMR (100 MHz, $CDCl_3$) δ 207.6, 137.7, 135.8, 130.5, 127.5, 126.9, 125.8, 98.9, 74.2, 20.4, 20.3 ppm. The ^{1}H NMR and ^{13}C NMR spectra are in agreement with those reported in the literature.[5i]

1-(Buta-2,3-dien-2-yl)-2-methoxybenzene(1o) [5e]

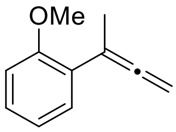

Colorless oil，95% yield from its 2,2-dibromocyclopropyl precursor. ^{1}H NMR (500 MHz, $CDCl_3$) δ 7.24-7.19 (m, 2H), 6.92 (td, *J* = 7.5, 1.1 Hz, 1H), 6.89-6.85 (m, 1H), 4.79 (q, *J* = 3.2 Hz, 2H), 3.83 (s, 3H), 2.09 (t, *J* = 3.2 Hz, 3H) ppm; ^{13}C NMR (126 MHz, $CDCl_3$) δ 209.4， 157.0 , 129.3 , 128.4 , 127.4 , 120.8 , 111.5 , 73.7 , 55.62, 19.4 ppm. The ^{1}H NMR and ^{13}C NMR spectra are in agreement with those reported in the literature.[5e]

1-(Buta-2,3-dien-2-yl)-2-chlorobenzene (1p) [5g]

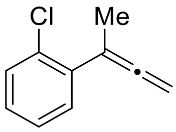

Colorless oil，72% yield from its 2,2-dibromocyclopropyl precursor. ^{1}H NMR (500 MHz, $CDCl_3$) δ 7.40-7.34 (m, 1H), 7.30 (dd, *J* = 7.6, 1.9 Hz, 1H), 7.23 (td, *J* = 7.4, 1.5 Hz, 1H), 7.18 (td, *J* = 7.7, 1.9 Hz, 1H), 4.84 (q, *J* = 3.2 Hz, 2H), 2.09 (t, *J* = 3.3 Hz, 3H) ppm. The ^{1}H NMR is in agreement with those reported in the literature.[5g]

2-(Buta-2,3-dien-2-yl)naphthalene (1q)[5e]

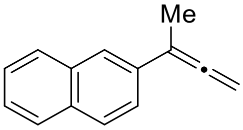

White solid, 89% yield from its 2,2-dibromocyclopropyl precursor. ^{1}H NMR (400 MHz, $CDCl_3$) δ 7.81-7.71 (m, 4H), 7.64-7.62 (m, 1H), 7.46-7.40 (m, 2H), 5.10 (q, *J* = 3.2 Hz, 2H), 2.21 (t, *J* = 3.2 Hz, 3H) ppm; ^{13}C NMR (100 MHz, $CDCl_3$) δ 209.6, 134.0, 133.6, 132.3, 127.9, 127.6, 127.5, 126.1, 125.6, 124.9, 123.3, 100.1, 77.2, 16.7 ppm. The ^{1}H NMR and ^{13}C NMR spectra are in agreement with those reported in the literature.[5e]

1-(Buta-2,3-dien-2-yl)naphthalene (1r)[5e]

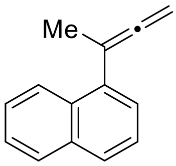

Light yellow oil, 89% yield from its 2,2-dibromocyclopropyl precursor. ^{1}H NMR (500 MHz, $CDCl_3$) δ 8.25-8.19 (m, 1H), 7.89-7.85 (m, 1H), 7.81-7.76 (m, 1H), 7.55-7.48 (m, 2H), 7.46 (d, *J* = 7.7 Hz, 1H), 7.45-7.42 (m, 1H), 4.86 (q, *J* = 3.3 Hz, 2H), 2.22 (t, *J* = 3.2 Hz, 3H) ppm; ^{13}C NMR (126 MHz, $CDCl_3$) δ 208.2, 136.6, 134.1 , 131.1 , 128.6 , 127.6 , 126.0 , 125.8 , 125.62 , 125.58 , 125.1 , 98.3 , 74.4 , 21.3 ppm. The ^{1}H NMR and ^{13}C NMR spectra are in agreement with those reported in the literature.[5e]

2-(Buta-2,3-dien-2-yl)furan (1s)

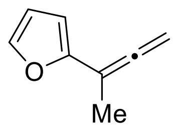

Colorless oil, 95% yield from its 2,2-dibromocyclopropyl precursor. ^{1}H NMR (400 MHz, $CDCl_3$) δ 7.40-7.35 (m, 1H), 6.39 (dd, *J* = 3.3, 1.9 Hz, 1H), 6.26-6.12 (m, 1H), 5.12 (q, *J* = 3.1 Hz, 2H), 2.00 (t, *J* = 3.1 Hz, 3H) ppm; ^{13}C NMR (100 MHz, $CDCl_3$) δ 208.2, 150.9, 142.1, 111.5, 106.2, 93.1, 78.3, 15.9 ppm. EI-MS m/z: 120.0 $[M]^+$; HRMS (EI) m/z: calcd. for $C_8H_8O^+$: 120.0575, Found: 120.0578 $[M]^+$.

5-(Buta-2,3-dien-2-yl)-1,2,3-trimethoxybenzene (1t)

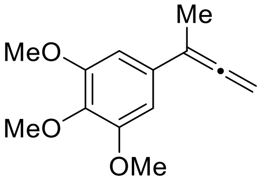

Light yellow solid, 45% yield from its 2,2-dibromocyclopropyl precursor. ^{1}H NMR (400 MHz, $CDCl_3$) δ 6.64 (s, 2H), 5.03 (q, *J* = 3.2 Hz, 2H), 3.87 (s, 6H), 3.84 (s, 3H), 2.09 (t, *J* = 3.2 Hz, 3H) ppm; ^{13}C NMR (100 MHz, $CDCl_3$) δ 208.8, 153.1, 137.0, 132.4, 103.0, 99.8, 77.1, 60.9, 56.1, 16.8 ppm. ESI-MS m/z: 221.1 $[M+H]^+$; HRMS (ESI) m/z: calcd. for $C_{13}H_{17}O_3^+$: 221.1172, Found: 221.1172 $[M+H]^+$.

5-(Buta-2,3-dien-2-yl)benzo[d][1,3]dioxole (1u) [5a]

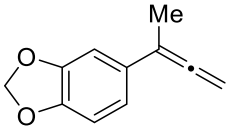

White solid, 86% yield from its 2,2-dibromocyclopropyl precursor. ^{1}H NMR (500 MHz, $CDCl_3$) δ 6.97-6.93 (m, 1H), 6.86-6.83 (m, 1H), 6.80-6.76 (m, 1H), 5.95 (s, 2H), 5.01 (q, *J* = 3.1 Hz, 2H), 2.06 (t, *J* = 3.2 Hz, 3H) ppm; ^{13}C NMR (126 MHz, $CDCl_3$) δ 208.8, 147.9, 146.5, 130.9, 118.8, 108.1, 106.6, 101.1, 99.7, 77.2, 17.1 ppm. The ^{1}H NMR and ^{13}C NMR spectra are in agreement with those reported in the literature. [5a]

1-(Buta-2,3-dien-2-yl)-4-(prop-1-en-2-yl)benzene (1v)

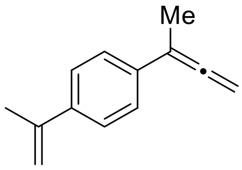

White solid, 96% yield from its 2,2-dibromocyclopropyl precursor. ^{1}H NMR (400 MHz, $CDCl_3$) δ 7.48-7.41 (m, 2H), 7.41-7.35 (m, 2H), 5.41-5.37 (m, 1H), 5.10-5.06 (m, 1H), 5.04 (q, *J* = 3.1 Hz, 2H), 2.18-2.13 (m, 3H), 2.1 (t, *J* = 3.2 Hz, 3H) ppm; ^{13}C NMR (100 MHz, $CDCl_3$) δ 209.2, 143.0, 139.5, 135.9, 125.61, 125.56, 112.2, 99.7, 77.1, 21.9, 16.8 ppm. EI-MS m/z: 170.0 $[M]^+$; HRMS (EI) m/z: calcd. for $C_{13}H_{14}^+$: 170.1096, Found: 170.1102 $[M]^+$.

1-Vinylidene-1,2,3,4-tetrahydronaphthalene (1w) [5e]

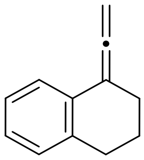

Colorless oil, 96% yield from its 2,2-dibromocyclopropyl precursor. ^{1}H NMR (500 MHz, $CDCl_3$) δ 7.52-7.47 (m, 1H), 7.17-7.12 (m, 1H), 7.12-7.07 (m, 2H), 5.07 (t, *J* = 3.3 Hz, 2H), 2.81 (t, *J* = 6.2 Hz, 2H), 2.62-2.56 (m, 2H), 1.94-1.88 (m, 2H) ppm; ^{13}C NMR (126 MHz, $CDCl_3$) δ 206.7, 136.5, 131.3, 129.3, 127.0, 126.6, 126.2, 101.1, 78.0, 30.2, 28.8, 22.9 ppm. The ^{1}H NMR and ^{13}C NMR spectra are in agreement with those reported in the literature.[5e]

4-Vinylidenechromane (1x) [5g]

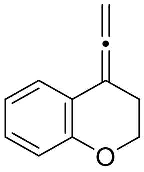

Light yellow oil, 93% yield from its 2,2-dibromocyclopropyl precursor. ^{1}H NMR (500 MHz, $CDCl_3$) δ 7.41-7.35 (m, 1H), 7.15-7.08 (m, 1H), 6.94-6.87 (m, 1H), 6.87-6.82 (m, 1H), 5.16 (t, *J* = 3.3 Hz, 2H), 4.25 (dd, *J* = 6.0, 5.1 Hz, 2H), 2.78-2.71 (m, 2H) ppm; ^{13}C NMR (126 MHz, $CDCl_3$) δ 205.1, 154.1, 128.4, 127.2, 121.1, 118.5, 117.5, 96.2,

79.6, 66.0, 27.9 ppm. The ^{1}H NMR and ^{13}C NMR spectra are in agreement with those reported in the literature. [5g]

Penta-1,2-dien-3-ylbenzene (1y) [5g]

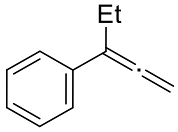

Colorless oil, 96% yield from its 2,2-dibromocyclopropyl precursor. ^{1}H NMR (400 MHz, $CDCl_3$) δ 7.43-7.41 (m, 2H), 7.35-7.30 (m, 2H), 7.22-7.18 (m, 1H), 5.12-5.10 (m, 2H), 2.47-2.40 (m, 2H), 1.19-1.14 (m, 3H) ppm; ^{13}C NMR (100 MHz, $CDCl_3$) δ 208.3, 136.5, 128.3, 126.5, 125.9, 106.6, 78.8, 22.3, 12.4 ppm. The ^{1}H NMR and ^{13}C NMR spectra are in agreement with those reported in the literature. [5g]

(1-Cyclopropylpropa-1,2-dien-1-yl)benzene (1z) [5e]

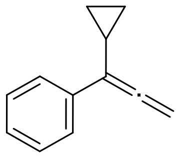

Light yellow oil, 93% yield from its 2,2-dibromocyclopropyl precursor. ^{1}H NMR (400 MHz, $CDCl_3$) δ 7.56 (d, *J* = 8.0 Hz, 2H), 7.32 (t, *J* = 7.6 Hz, 2H), 7.19 (t, *J* = 7.6 Hz, 1H), 5.07 (d, *J* = 2.8 Hz, 2H), 1.57-1.50 (m, 1H), 0.88-0.83 (m, 2H), 0.56-0.52 (m, 2H) ppm; ^{13}C NMR (100 MHz, $CDCl_3$) δ 207.8, 136.7, 128.3, 126.7, 126.2, 108.2, 79.2, 10.3, 6.8 ppm. The ^{1}H NMR and ^{13}C NMR spectra are in agreement with those reported in the literature. [5e]

3-methylnona-1,2-diene (1aa)[5m]

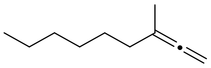

Colorless oil, 60% yield from its 2,2-dibromocyclopropyl precursor. ^{1}H NMR (400 MHz, $CDCl_3$) δ 4.57 (h, *J* = 3.2 Hz, 2H), 1.95-1.89 (m, 2H), 1.67 (t, *J* = 3.1 Hz, 3H), 1.46-1.38 (m, 2H) , 1.31-1.25 (m, 6H), 0.89 (t, *J* = 6.6 Hz, 3H) ppm; ^{13}C NMR (100 MHz, $CDCl_3$) δ 206.3, 98.6, 73.9, 33.6, 31.9, 29.1, 27.5, 22.8, 18.9, 14.3 ppm. The ^{1}H

NMR and ^{13}C NMR spectra are in agreement with those reported in the literature.[5m]

4-ethyl-3-methylhexa-1,2-diene (1ab)[5n]

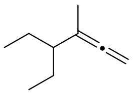

Colorless oil, 65% yield from its 2,2-dibromocyclopropyl precursor. ^{1}H NMR (500 MHz, $CDCl_3$) δ 4.56 (qd, *J* = 3.1, 1.4 Hz, 2H), 1.82-1.71 (m, 1H), 1.61 (t, *J* = 3.2 Hz, 3H), 1.44-1.33 (m, 4H), 0.86 (t, *J* = 7.4 Hz, 6H) ppm. The ^{1}H NMR spectra are in agreement with those reported in the literature.[5n]

Buta-2,3-dien-2-ylcyclohexane (1ac)[5b]

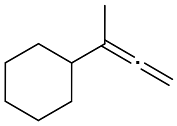

Colorless oil, 85% yield. ^{1}H NMR (400 MHz, $CDCl_3$) δ 4.59 (app quintet, *J* = 3.2 Hz, 2H), 1.81-1.62 (m, 9H), 1.29-1.08 (m, 5H) ppm. The ^{1}H NMR spectra are in agreement with those reported in the literature.[5b]

Buta-2,3-dien-2-ylcyclohexane (1ad)[5l]

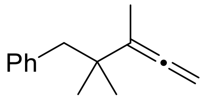

Colorless oil, 50% yield from its propargylic alcohol. ^{1}H NMR (500 MHz, $CDCl_3$) δ 7.27-7.22 (m, 2H), 7.21-7.17 (m, 1H), 7.13-7.08 (m, 2H), 4.47 (q, *J* = 3.1 Hz, 2H), 2.64 (s, 2H), 1.76 (t, *J* = 3.1 Hz, 3H), 0.99 (s, 6H) ppm; ^{13}C NMR (126 MHz, $CDCl_3$) δ 206.5, 139.3, 130.6, 127.7, 126.0, 105.6, 74.5, 46.8, 36.9, 26.8, 15.4 ppm. The ^{1}H NMR and ^{13}C NMR spectra are in agreement with those reported in the literature.[5l]

1-methyl-4-(1-phenylpropa-1,2-dien-1-yl)benzene (1ae)[5k]

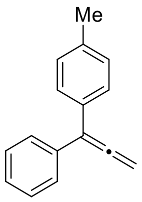

Light yellow oil, 95% yield from its 2,2-dibromocyclopropyl precursor. ^{1}H NMR (500 MHz, $CDCl_3$) δ 7.39-7.30 (m, 4H), 7.28-7.23 (m, 3H), 7.17-7.13 (m, 2H), 5.24 (s, 2H), 2.36 (s, 3H) ppm; ^{13}C NMR (126 MHz, $CDCl_3$) δ 209.8, 137.0, 136.4, 133.3, 129.1, 128.39, 128.36, 128.32, 127.1, 109.0, 77.9, 21.2 ppm. The ^{1}H NMR and ^{13}C NMR spectra are in agreement with those reported in the literature.[5k]

1-methyl-2-(1-phenylpropa-1,2-dien-1-yl)benzene (**1af**)[5j]

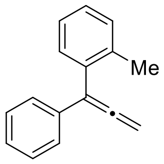

Colorless oil, 80% yield from its its 2,2-dibromocyclopropyl precursor. ^{1}H NMR (500 MHz, $CDCl_3$) δ 7.30-7.17 (m, 9H), 5.16 (s, 2H), 2.20 (s, 3H) ppm; ^{13}C NMR (126 MHz, $CDCl_3$) δ 208.3, 137.0, 136.4, 135.5, 130.4, 130.3, 128.43, 127.7, 126.8, 126.7, 126.0, 107.2, 77.6, 20.1 ppm. The ^{1}H NMR and ^{13}C NMR spectra are in agreement with those reported in the literature.[5j]

4. General procedures for palladium-catalyzed diboration of 1,1-disubstituted allenes

4.1. General procedure for palladium-catalyzed diboration of 1,1-disubstituted allenes

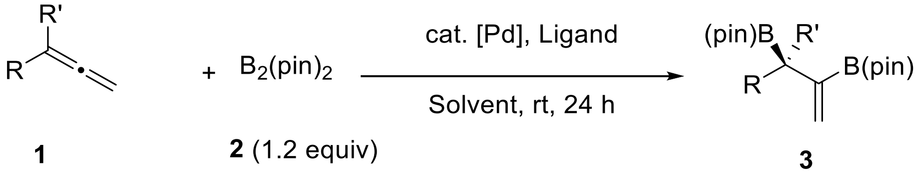

To a 25 mL flame-dried Schlenk tube was added palladium precursor (2.0 mol % Pd) and chiral ligand (2.5 mol %), then purged with nitrogen three times. Solvent (2.0 mL) was added and the mixture was stirred at rt for 0.5 h. Then buta-2,3-dien-2-

ylbenzene (**1a,** 0.2 mmol, 1.0 equiv) and B_2pin_2 (0.24 mmol, 1.2 equiv) were added in one portion. The resulting mixture was stirred under nitrogen at rt for 24 h, and then quenched with saturated NH_4Cl solution (5 mL). EtOAc (10 mL) was added and the organic layer was separated, washed with brine, dried over sodium sulfate, and concentrated. The residue was purified by flash chromatography on silica gel and the enantiomeric excess was determined by chiral HPLC.

4.2. Asymmetric diboration of 1a: optimization of reaction conditions

Table S1 Asymmetric diboration of **1a:** solvent optimization[a]

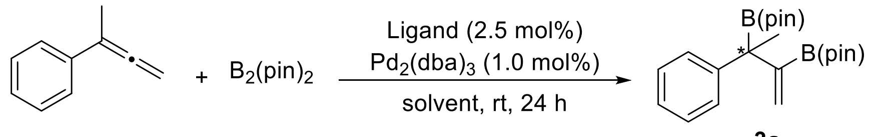

Entry	Ligand	Solvent	Yield (%)[b]	ee (%)[c]
1	(*S*, *S*, *S*)-SKP **L4**	Toluene	96	-51
2[d]	(*S*, *S*, *S*)-SKP **L4**	Toluene	48	-56
3	(*S*, *S*, *S*)-SKP **L4**	DCM	42	-36
4	(*S*, *S*, *S*)-SKP **L4**	THF	93	-47
5	(*S*, *S*, *S*)-SKP **L4**	CH_3CN	41	-20
6	(*S*, *S*, *S*)-SKP **L4**	MeOH	13	-40
7	(*S*, *S*, *S*)-SKP **L4**	DMF	41	-24
8	(*S*, *S*, *S*)-SKP **L4**	EA	73	-38
9	(*S*, *S*, *S*)-SKP **L4**	*n*-Hexane	88	-59
10	(*S*, *S*, *S*)-SKP **L4**	PhF	51	-49
11	(*S*, *S*, *S*)-SKP **L4**	1,4-dioxane	96	-48
12	(*S*, *S*, *S*)-SKP **L4**	Et_2O	93	-53
13	(*S*, *S*, *S*)-SKP **L4**	Benzene	92	-47

14	(*S*, *S*, *S*)-SKP **L4**	*p*-xylene	80	-50
15	(*S*, *S*, *S*)-SKP **L4**	CyH	95	-59
16[e]	(*S*, *S*, *S*)-SKP **L4**	CyH	85	-59
17	(*S*)-BI-DIME **L8**	CyH	98	94
18	(*S*)-BI-DIME **L8**	Toluene	82	92
19	(*S*)-BI-DIME **L8**	THF	51	91
20	(*S*)-BI-DIME **L8**	1,4-dioxane	27	92
21	(*S*)-BI-DIME **L8**	DCM	N.R.	N.D.

[a] Unless otherwise noted, all reactions were performed at rt for 24 h in the presence of **1a** (0.2 mmol), **2** (0.24 mmol), Ligand (2.5 mol%) and $Pd_2(dba)_3$ (1.0 mol%) in a specified solvent (2.0 mL). The reactions have an excellent regioselectivity and the **3a** was the only product. [b] Yield of the isolated **3a**. [c] The *ee* value was determined by HPLC using a chiral column. [d] The reaction was performed at 0 °C. [e] (*S*, *S*, *S*)-**SKP L4** (1.2 mol%) and $Pd_2(dba)_3$ (0.05 mol%) were used. N.R. = No Reaction; N.D. = Not Determined.

Table S2 Asymmetric diboration of **1a:** chiral ligand[a]

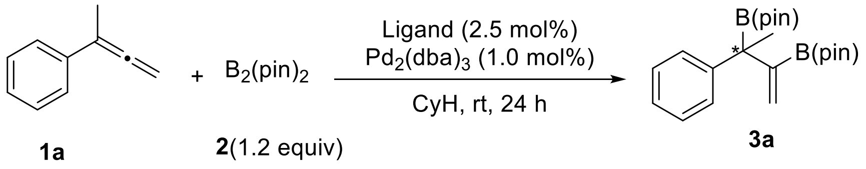

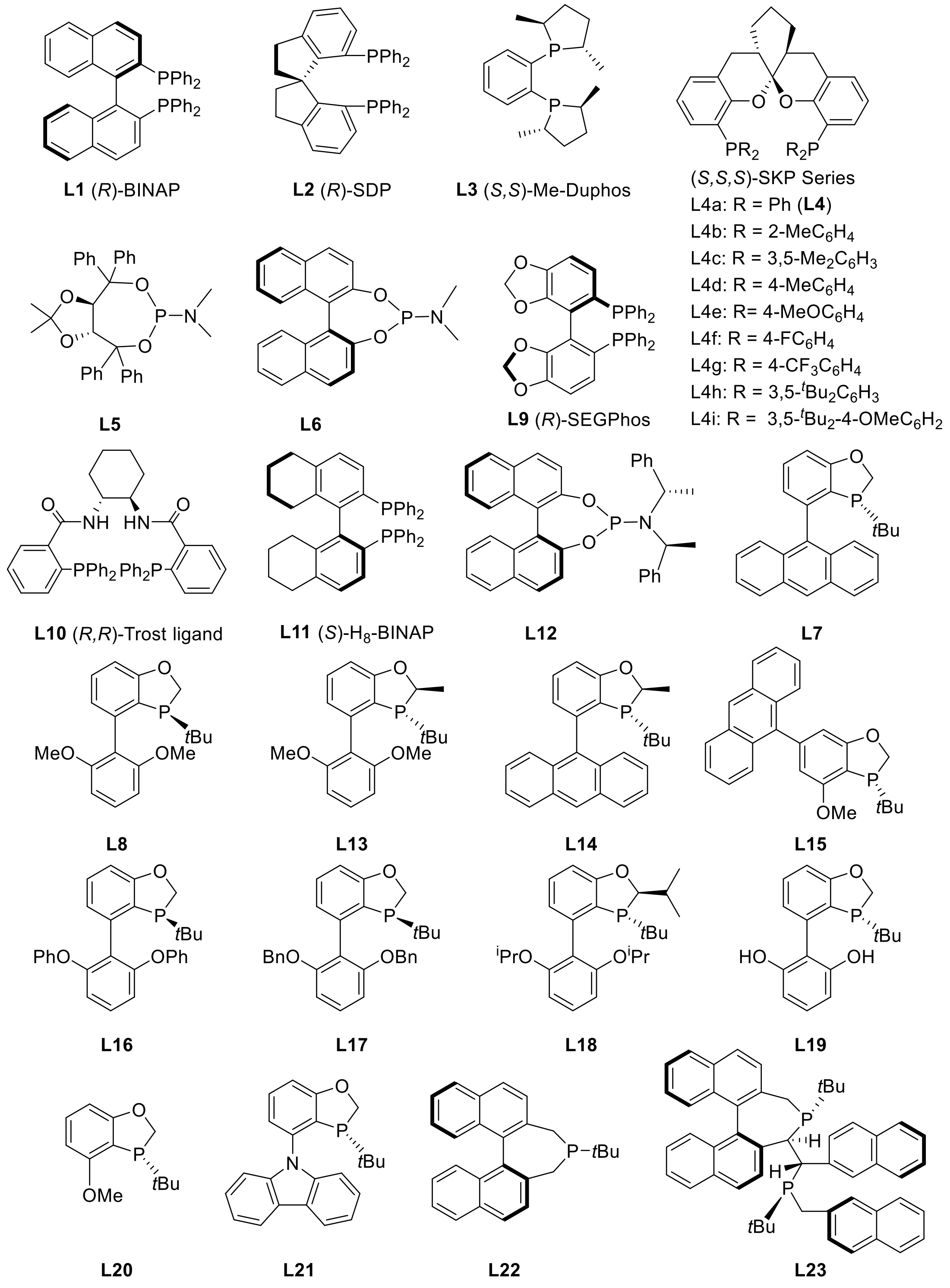

Entry	Ligand	Yield (%)[b]	ee (%)[c]
1	**L1**	N.R.	N.D.
2	**L2**	N.R.	N.D.

3	**L3**	15	52
4	**L4a**	95	59
5	**L4b**	97	39
6	**L4c**	68	43
7	**L4d**	92	63
8	**L4e**	85	72
9	**L4f**	95	76
10	**L4g**	97	78
11	**L4h**	97	74
12	**L4i**	97	82
13	**L5**	76.	79.
14	**L6**	45.	12.
15	**L7**	60	91
16	**L8**	98	94
17	**L9**	N.R.	N.D.
18	**L10**	N.R.	N.D.
19	**L11**	N.R.	N.D.
20	**L12**	26	5
21	**L13**	88	35
22	**L14**	69	24
23	**L15**	96	19
24	**L16**	92	94

25	**L17**	95	92
26	**L18**	N.R.	N.D.
27	**L19**	<10	6
28	**L20**	<10	0
29	**L21**	92	91
30	**L22**	88	28
31	**L23**	26	82

[a] Unless otherwise noted, all reactions were performed at rt for 24 h in the presence of **1a** (0.2 mmol), **2** (0.24 mmol), ligand (2.5 mol%) and $Pd_2(dba)_3$ (1.0 mol%) in CyH (2.0 mL). The reactions have an excellent regioselectivity and **3a** was the only product. [b] Yield of the isolated **3a**. [c] The *ee* value was determined by HPLC using a chiral column. N.R. = No Reaction; N.D. = Not Determined.

Table S3 Asymmetric diboration of **1a** catalyzed by Pd-(*S*)-BI-DIME: optimization of palladium precursors[a]

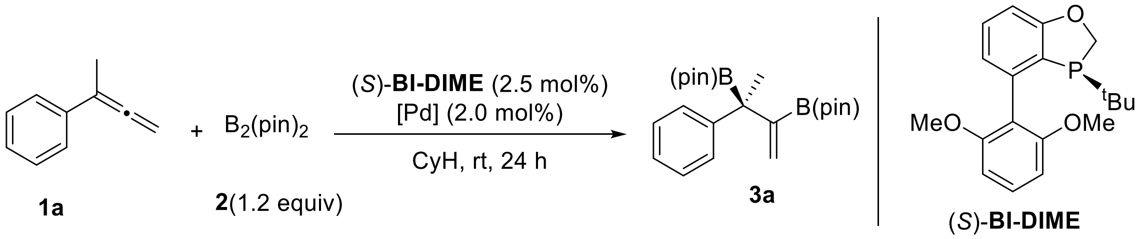

Entry	[Pd]	Yield (%)[b]	ee (%)[c]
1	$Pd_2(dba)_3$	98	94
2	$Pd(dba)_2$	87	95
3	$Pd(PPh_3)_4$	80	54
4	$Pd(OAc)_2$	33	92
5	$[PdCl(Cinnamyl)]_2$	N.R.	N.D.
6	$PdBr_2$	N.R.	N.D.

7	$PdCl_2(PPh_3)_2$	N.R.	N.D.

[a] Unless otherwise noted, all reactions were performed at rt for 24 h in the presence of **1a** (0.2 mmol), **2** (0.24 mmol), (*S*)-BI-DIME (2.5 mol%) and [Pd] (2.0 mol% in terms of palladium atom) in CyH (2.0 mL). The reactions have an excellent regioselectivity and the **3a** was the only product. [b] Yield of the isolated **3a**. [c] The *ee* value was determined by HPLC using a chiral column. N.R. = No Reaction; N.D. = Not Determined.

Table S4 Asymmetric diboration of **1a** catalyzed by Pd-(*S*)-BI-DIME: Effects of Pd/ligand ratio [a]

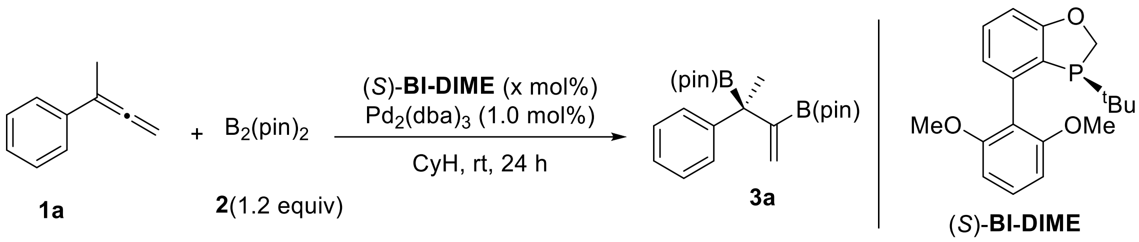

Entry	[Pd]/L	Yield (%)[b]	ee (%)[c]
1	1:1	98	94
2	1:2	99	93
3	2:1	95	91
4	No Ligand	<5%	N.D.
5	No $Pd_2(dba)_3$	N.R.	N.D.
6	Pd(0)[(*S*)-BI-DIME]$_2$ [d]	99	94

[a] Unless otherwise noted, all reactions were performed at rt for 24 h in the presence of **1a** (0.2 mmol), **2** (0.24 mmol), (*S*)-BI-DIME (x mol%) and $Pd_2(dba)_3$ (1.0 mol%) in CyH (2.0 mL). The reactions have an excellent regioselectivity and the **3a** was the only product. [b] Yield of the isolated **3a**. [c] The *ee* value was determined by HPLC using a chiral column. [d] The Pd(0)[(*S*)-BI-DIME]$_2$ complex (1.0 mol%) was used. N.R. = No Reaction; N.D. = Not determined.

Table S5 Asymmetric diboration of **1a** catalyzed by Pd-(*S*)-BI-DIME: Study of catalyst loading[a]

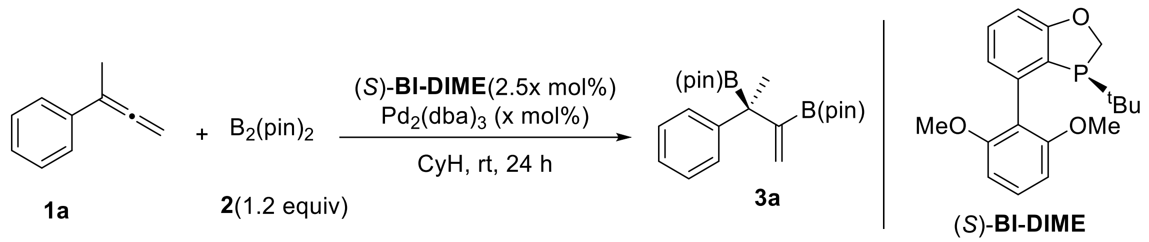

Entry	$Pd_2(dba)_3$	Yield (%)[b]	ee (%)[c]
1	1.0 mol%	98	94
2	0.5 mol%	95	91
3	0.1 mol%	85	94
4[d]	0.5 mol%	96	94
5[d]	0.1 mol%	96	93
6[e]	0.1 mol%	97	94

[a] Unless otherwise noted, all reactions were performed at rt for 24 h in the presence of **1a** (4.0 mmol), **2** (4.8 mmol), (*S*)-BI-DIME (2.5x mol%) and $Pd_2(dba)_3$ (x mol%) in CyH (20.0 mL). The reactions have an excellent regioselectivity and the **3a** was the only product. [b] Yield of the isolated **3a**. [c] The *ee* value was determined by HPLC using a chiral column. [d] 48 h. [e] 72 h.

5. Analytical data of chiral diboronate products

(*R*)-2,2'-(3-Phenylbut-1-ene-2,3-diyl)bis(4,4,5,5-tetramethyl-1,3,2-dioxaborolane) (3a)

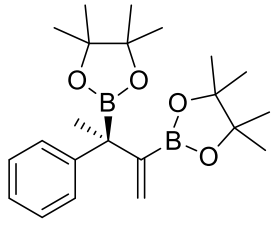

White solid, 98% yield, M.p. 70-72 °C. $[\alpha]_D^{20}$ = 53.6 (*c* 0.80, $CHCl_3$), 94% *ee* [determined by HPLC analysis using a Chiralcel IC-3 column; *n*-Hex/*i*-PrOH = 99:1, 0.7 mL/min, λ = 214 nm; t_R (major) = 5.21 min; t_R (minor) = 6.51 min]. ^{1}H NMR (400 MHz, $CDCl_3$) δ 7.37-7.35 (m, 2H), 7.27-7.23 (m, 2H), 7.14-7.11 (m, 1H), 5.82 (d, *J* =

2.8 Hz, 1H), 5.18 (d, *J* = 2.8 Hz, 1H), 1.52 (s, 3H), 1.24 (s, 6H), 1.22-1.20 (m, 18H) ppm; ^{13}C NMR (100 MHz, $CDCl_3$) δ 144.9, 128.4, 127.6, 126.8, 125.2, 83.4, 83.3, 24.9, 24.8, 24.4, 24.3, 22.3 ppm; ^{11}B NMR (128 MHz, $CDCl_3$) δ 33.1, 30.8 ppm. IR (neat) ν 2979, 1308, 1140, 1089, 884, 701 cm^{-1}; ESI-MS m/z: 402.3 $[M+NH_4]^+$; HRMS (ESI) m/z: calcd. for $C_{22}H_{38}{}^{10}B_2NO_4^+$: 400.3054, Found: 400.3052 $[M+NH_4]^+$.

(*R*)-2,2'-(3-(*p*-Tolyl)but-1-ene-2,3-diyl)bis(4,4,5,5-tetramethyl-1,3,2-dioxaborolane) (3b)

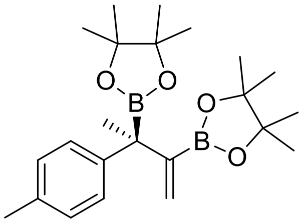

White solid, 91% yield, M.p. 88-89 °C. $[\alpha]_D^{20}$ = 51.7 (*c* 0.80, $CHCl_3$), 93% *ee* [determined by HPLC analysis using a Chiralcel OD-H column; *n*-Hex/*i*-PrOH = 99.9:0.1, 0.7 mL/min, λ = 214 nm; t_R (major) = 5.14 min; t_R (minor) = 6.48 min]. ^1H NMR (400 MHz, $CDCl_3$) δ 7.24 (d, *J* = 8.0 Hz, 2H), 7.07 (d, *J* = 8.0 Hz, 2H), 5.81 (d, *J* = 2.8 Hz, 1H), 5.15 (d, *J* = 2.8 Hz, 1H), 2.29 (s, 3H), 1.50 (s, 3H), 1.26-1.22 (m, 24 H) ppm; ^{13}C NMR (100 MHz, $CDCl_3$) δ 141.6, 134.4, 128.4, 128.3, 126.8, 83.3, 83.2, 24.9, 24.8, 24.33, 24.28, 22.3, 20.9 ppm. ^{11}B NMR (128 MHz, $CDCl_3$) δ 33.2, 30.8 ppm; IR (neat) ν 2975, 1348, 1305, 1143, 1093, 854 cm^{-1}; ESI-MS m/z: 416.3 $[M+NH_4]^+$; HRMS (ESI) m/z: calcd. for $C_{23}H_{40}{}^{10}B_2NO_4^+$: 414.3211, Found: 414.3208 $[M+NH_4]^+$.

(*R*)-2,2'-(3-(4-Methoxyphenyl)but-1-ene-2,3-diyl)bis(4,4,5,5-tetramethyl-1,3,2-dioxaborolane) (3c)

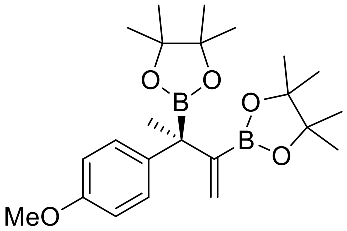

White solid, 90% yield, M.p. 95-97 °C. $[\alpha]_D^{20}$ = 60.3 (*c* 0.80, $CHCl_3$), 94% *ee* [determined by HPLC analysis using a Chiralcel ID-3 column; *n*-Hex/*i*-PrOH = 99:1,

0.7 mL/min, λ = 214 nm; t_R (major) = 6.40 min; t_R (minor) = 7.06 min]. ^{1}H NMR (400 MHz, $CDCl_3$) δ 7.28 (d, *J* = 8.4 Hz, 2H), 6.81 (d, *J* = 8.8 Hz, 2H), 5.80 (d, *J* = 2.8 Hz, 1H), 5.18 (d, *J* = 2.8 Hz, 1H), 3.77 (s, 3H), 1.49 (s, 3H), 1.26-1.21 (m, 24 H) ppm; ^{13}C NMR (100 MHz, $CDCl_3$) δ 157.2, 136.8, 129.3, 126.5, 113.0, 83.3, 83.2, 55.0, 24.8, 24.7, 24.33, 24.27 22.4 ppm; ^{11}B NMR (128 MHz, $CDCl_3$) δ 33.0, 31.0 ppm. IR (neat) ν 2978, 1510, 1373, 1304, 1140, 1089, 845 cm^{-1}; ESI-MS m/z: 432.2 $[M+NH_4]^+$; HRMS (ESI) m/z: calcd. for $C_{23}H_{40}{}^{10}B_2NO_5{}^+$: 430.3160, Found: 430.3157 $[M+NH_4]^+$.

(*R*)-2,2'-(3-(4-Fluorophenyl)but-1-ene-2,3-diyl)bis(4,4,5,5-tetramethyl-1,3,2-dioxaborolane) (3d)

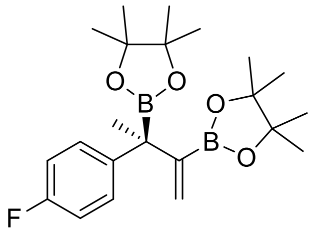

White solid, 98% yield, M.p. 69-71 °C. $[\alpha]_D^{20}$ = 22.0 (*c* 1.00, $CHCl_3$), 90% *ee* [determined by HPLC analysis using a Chiralcel IC-3 column; *n*-Hex/*i*-PrOH = 99:1, 0.7 mL/min, λ = 214 nm; t_R (major) = 4.86 min; t_R (minor) = 5.16 min]. ^{1}H NMR (400 MHz, $CDCl_3$) δ 7.35-7.31 (m, 2H), 6.96-6.91 (m, 2H), 5.83 (d, *J* = 2.8 Hz, 1H),5.22 (d, *J* = 2.8 Hz, 1H), 1.49 (s, 3H), 1.24 (s, 6H), 1.22 (s, 6H), 1.20 (s, 6H), 1.19 (s, 6H) ppm; ^{13}C NMR (100 MHz, $CDCl_3$) δ 160.8 (d, $J_{(F,C)}$ = 242.0 Hz), 140.7 (d, $J_{(F,C)}$ = 3.0 Hz), 129.8 (d, $J_{(F,C)}$ = 7.5 Hz), 126.6, 114.3 (d, $J_{(F,C)}$ = 21.4 Hz), 83.5, 83.3, 24.8, 24.7, 24.4, 24.3, 22.6 ppm; ^{19}F NMR (376 MHz, $CDCl_3$) δ -118.9 ppm; ^{11}B NMR (128 MHz, $CDCl_3$) δ 33.0, 30.8 ppm. IR (neat) ν 2977, 1507, 1373, 1349, 1306, 1142, 1094, 854 cm^{-1}; ESI-MS m/z: 420.2 $[M+NH_4]^+$; HRMS (ESI) m/z: calcd. for $C_{22}H_{37}{}^{10}B_2FNO_4{}^+$: 418.2960, Found: 418.2957 $[M+NH_4]^+$.

(*R*)-2,2'-(3-(4-Chlorophenyl)but-1-ene-2,3-diyl)bis(4,4,5,5-tetramethyl-1,3,2-dioxaborolane) (3e)

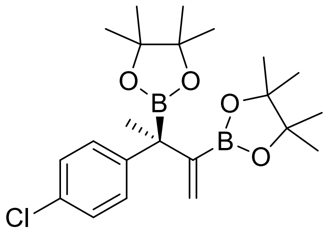

White solid, 93% yield, M.p. 67-69 °C. $[\alpha]_D^{20}$ = 33.7 (*c* 1.00, $CHCl_3$), 92% *ee* [determined by HPLC analysis using a Chiralcel ID-3 column; *n*-Hex/*i*-PrOH = 99:1, 0.7 mL/min, λ = 214 nm; t_R (major) = 5.29 min; t_R (minor) = 5.63 min]. ^{1}H NMR (400 MHz, $CDCl_3$) δ 7.31 (d, *J* = 8.4 Hz, 2H), 7.22 (d, *J* = 8.8 Hz, 2H), 5.84 (d, *J* = 2.8 Hz, 1H), 5.23 (d, *J* = 2.8 Hz, 1H), 1.48 (s, 3H), 1.26-1.19 (m, 24H) ppm; ^{13}C NMR (100 MHz, $CDCl_3$) δ 143.8, 130.8, 129.8, 127.7, 126.9, 83.5, 83.3, 24.8, 24.7, 24.4, 24.3, 22.4 ppm; ^{11}B NMR (128 MHz, $CDCl_3$) δ 33.2, 30.9 ppm. IR (neat) ν 2978, 1347, 1314, 1143, 1096, 854 cm^{-1}; ESI-MS m/z: 436.2 $[M+NH_4]^+$; HRMS (ESI) m/z: calcd. for $C_{22}H_{37}{}^{10}B_2ClNO_4^+$: 434.2664, Found: 434.2664 $[M+NH_4]^+$.

(*R*)-2,2'-(3-(4-Bromophenyl)but-1-ene-2,3-diyl)bis(4,4,5,5-tetramethyl-1,3,2-dioxaborolane) (3f)

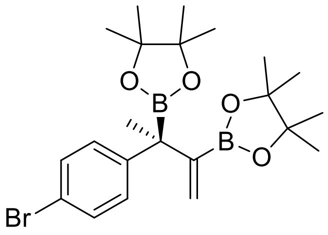

White solid, 85% yield, M.p. 87-88 °C. $[\alpha]_D^{20}$ = 30.9 (*c* 1.00, $CHCl_3$), 90% *ee* [determined by HPLC analysis using a Chiralcel ID-3 column; *n*-Hex/*i*-PrOH = 99:1, 0.7 mL/min, λ = 214 nm; t_R (major) = 5.34 min; t_R (minor) = 5.70 min]. ^{1}H NMR (400 MHz, $CDCl_3$) δ 7.37 (d, *J* = 8.4 Hz, 2H), 7.25 (d, *J* = 8.4 Hz, 2H), 5.84 (d, *J* = 2.4 Hz, 1H), 5.23 (d, *J* = 2.0 Hz, 1H), 1.47 (s, 3H), 1.23-1.19 (m, 24H) ppm; ^{13}C NMR (100 MHz, $CDCl_3$) δ 144.4, 130.6, 130.3, 127.0, 119.1, 83.5, 83.4, 24.8, 24.7, 24.4, 24.3, 22.4 ppm; ^{11}B NMR (128 MHz, $CDCl_3$) δ 33.0, 30.9 ppm. IR (neat) ν 2976, 1303, 1143, 1097, 886, 855, 711 cm^{-1}; ESI-MS m/z: 485.1 $[M+Na]^+$; HRMS (ESI) m/z: calcd. for $C_{22}H_{37}{}^{10}B_2BrNO_4^+$: 478.2159, Found: 478.2161 $[M+NH_4]^+$.

(*R*)-2,2'-(3-(4-(Trifluoromethyl)phenyl)but-1-ene-2,3-diyl)bis(4,4,5,5-tetramethyl-1,3,2-dioxaborolane) (3g)

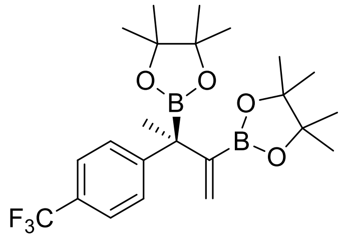

White solid, 94% yield, M.p. 76-78 °C. $[\alpha]_D^{20}$ = 30.6 (*c* 1.00, $CHCl_3$), 87% *ee* [determined by HPLC analysis using a Chiralcel IC-3 column; *n*-Hex/*i*-PrOH = 99.5:0.5, 0.4 mL/min, λ = 214 nm; t_R (major) = 9.68 min; t_R (minor) = 10.33 min]. ^1H NMR (400 MHz, $CDCl_3$) δ 7.52-7.47 (m, 4H), 5.88 (d, *J* = 2.8 Hz, 1H), 5.26 (d, *J* = 2.4 Hz, 1H), 1.52 (s, 3H), 1.24 (s, 6H), 1.23 (s, 6H), 1.19 (s, 6H), 1.18 (s, 6H) ppm; ^{13}C NMR (100 MHz, $CDCl_3$) δ 144.9, 128.7, 127.3 (q, $J_{(F,C)}$ = 32.5 Hz), 127.2, 124.5 (q, $J_{(F,C)}$ = 270.6 Hz), 124.48 (q, $J_{(F,C)}$ = 3.3 Hz), 83.6, 83.4, 24.72, 24.71, 24.4, 24.3, 22.4 ppm; ^{19}F NMR (376 MHz, $CDCl_3$) δ -62.2 ppm; ^{11}B NMR (128 MHz, $CDCl_3$) δ 33.3, 30.8 ppm. IR (neat) ν 2977, 1611, 1321, 1143, 1118, 1072, 853, 705, 680 cm^{-1}; ESI-MS m/z: 453.2 $[M+H]^+$; HRMS (ESI) m/z: calcd. for $C_{23}H_{37}{}^{10}B_2F_3NO_4^+$: 468.2928, Found: 468.2929 $[M+NH_4]^+$.

(*R*)-2,2'-(3-(4-(Benzyloxy)phenyl)but-1-ene-2,3-diyl)bis(4,4,5,5-tetramethyl-1,3,2-dioxaborolane) (3h)

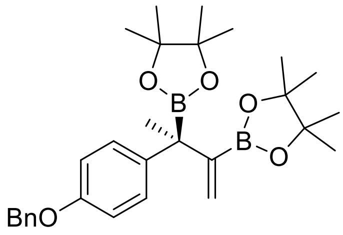

White solid, 93% yield, M.p. 89-91 °C. $[\alpha]_D^{20}$ = 22.7 (*c* 1.00, $CHCl_3$), 90% *ee* [determined by HPLC analysis using a Chiralcel IC-3 column; *n*-Hex/*i*-PrOH = 99:1, 0.7 mL/min, λ = 214 nm; t_R (major) = 7.32 min; t_R (minor) = 10.40 min]. ^1H NMR (400 MHz, $CDCl_3$) δ 7.43-7.41 (m, 2H), 7.38-7.34 (m, 2H), 7.31-7.26 (m, 3H), 6.90-6.87 (m, 2H), 5.81 (d, *J* = 2.8 Hz, 1H), 5.20 (d, *J* = 2.8 Hz, 1H), 5.02 (s, 2H), 1.49 (s, 3H), 1.25 (s, 6H), 1.24 (s, 6H), 1.20 (s, 6H), 1.19 (s, 6H) ppm; ^{13}C NMR (100 MHz, $CDCl_3$) δ

156.5, 137.3, 137.2, 129.4, 128.4, 127.7, 127.4, 126.5, 114.0, 83.3, 83.2, 69.8, 24.8, 24.7, 24.4, 24.3, 22.4 ppm; ^{11}B NMR (128 MHz, $CDCl_3$) δ 32.0 ppm. IR (neat) ν 2974, 1670, 1601, 1508, 1307, 1239, 1138, 833, 698 cm^{-1}; ESI-MS m/z: 508.3 $[M+NH_4]^+$; HRMS (ESI) m/z: calcd. for $C_{29}H_{44}{}^{10}B_2NO_5^+$: 506.3473, Found: 506.3469 $[M+NH_4]^+$.

(*R*)-(4-(2,3-Bis(4,4,5,5-tetramethyl-1,3,2-dioxaborolan-2-yl)but-3-en-2-yl)phenoxy)(tert-butyl)dimethylsilane (3i)

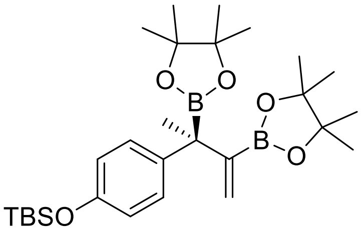

White solid, 93% yield, M.p. 91-93 ºC. $[\alpha]_D^{20}$ = 30.7 (*c* 1.00, $CHCl_3$), 91% *ee* [determined by HPLC analysis using a Chiralcel IC-3 column; *n*-Hex/*i*-PrOH = 99:1, 0.5 mL/min, λ = 214 nm; t_R (major) = 6.34 min; t_R (minor) = 6.96 min]. ^1H NMR (400 MHz, $CDCl_3$) δ 7.20 (d, *J* = 8.8 Hz, 2H), 6.73 (d, *J* = 8.8 Hz, 2H), 5.79 (d, *J* = 3.2 Hz, 1H), 5.20 (d, *J* = 2.8 Hz, 1H), 1.48 (s, 3H), 1.23-1.20 (s, 24H), 0.97 (s, 9H), 0.17 (s, 6H) ppm; ^{13}C NMR (100 MHz, $CDCl_3$) δ 153.1, 137.5, 129.3, 126.4, 119.2, 83.3, 83.2, 25.7, 24.9, 24.7, 24.4, 24.3, 22.4, 18.2, -4.5 ppm; ^{11}B NMR (128 MHz, $CDCl_3$) δ 32.1 ppm. IR (neat) ν 2968, 1305, 1253, 1145, 1086, 914, 837, 683 cm^{-1}; ESI-MS m/z: 532.4 $[M+NH_4]^+$; HRMS (ESI) m/z: calcd. for $C_{28}H_{52}{}^{10}B_2NO_5Si^+$: 530.3868, Found: 530.3862 $[M+NH_4]^+$.

(*R*)-2,2'-(3-(*m*-Tolyl)but-1-ene-2,3-diyl)bis(4,4,5,5-tetramethyl-1,3,2-dioxaborolane) (3j)

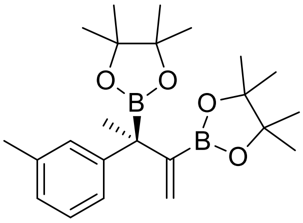

White solid, 91% yield, M.p. 84-86 ºC. $[\alpha]_D^{20}$ = 35.4 (*c* 1.00, $CHCl_3$), 92% *ee* [determined by HPLC analysis using a Chiralcel IC-3 column; *n*-Hex/*i*-PrOH = 99:1,

0.7 mL/min, λ = 214 nm; t_R (major) = 5.01 min; t_R (minor) = 5.78 min]. ^{1}H NMR (400 MHz, $CDCl_3$) δ 7.16-7.15 (m, 3H), 6.95-6.93 (m, 1H), 5.81 (d, *J* = 2.8 Hz, 1H), 5.15 (d, *J* = 2.8 Hz, 1H), 2.30 (s, 3H), 1.51 (s, 3H), 1.25-1.22 (m, 24H) ppm; ^{13}C NMR (100 MHz, $CDCl_3$) δ 144.6, 136.9, 129.2, 127.5, 126.9, 125.9, 125.4, 83.4, 83.3, 24.9, 24.8, 24., 24.2, 22.2, 21.5 ppm; ^{11}B NMR (128 MHz, $CDCl_3$) δ 33.3, 31.0 ppm. IR (neat) ν 2977, 1350, 1303, 1143, 1096, 851, 705 cm^{-1}; ESI-MS m/z: 416.3 $[M+NH_4]^+$; HRMS (ESI) m/z: calcd. for $C_{22}H_{40}{}^{10}B_2NO_4{}^+$: 414.3211, Found: 414.3207 $[M+NH_4]^+$.

(*R*)-2,2'-(3-(3-Chlorophenyl)but-1-ene-2,3-diyl)bis(4,4,5,5-tetramethyl-1,3,2-dioxaborolane) (3k)

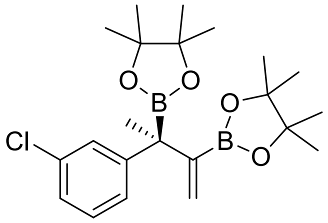

White solid, 97% yield, M.p. 81-83 °C. $[\alpha]_D^{20}$ = 40.4 (*c* 1.00, $CHCl_3$), 92% *ee* [determined by HPLC analysis using a Chiralcel ID-3 column; *n*-Hex/*i*-PrOH = 99:1, 0.7 mL/min, λ = 214 nm; t_R (major) = 5.13 min; t_R (minor) = 5.69 min]. ^{1}H NMR (400 MHz, $CDCl_3$) δ 7.37 (t, *J* = 1.6 Hz, 1H), 7.27-7.24 (m, 1H), 7.17 (t, *J* = 8.0 Hz, 1H), 7.12-7.09 (m, 1H), 5.85 (d, *J* = 2.4 Hz, 1H), 5.25 (d, *J* = 2.8 Hz, 1H), 1.49 (s, 3H), 1.24 (s, 6H), 1.22 (s, 6H), 1.20 (s, 6H), 1.19 (s, 6H) ppm; ^{13}C NMR (100 MHz, $CDCl_3$) δ 147.6, 133.5, 128.8, 128.7, 127.0, 126.7, 125.3, 83.6, 83.4, 24.8, 24.7, 24.4, 24.3, 22.3 ppm; ^{11}B NMR (128 MHz, $CDCl_3$) δ 33.2, 30.7 ppm. IR (neat) ν 2976, 1589, 1462, 1311, 1138, 840, 786 cm^{-1}; ESI-MS m/z: 436.2 $[M+NH_4]^+$; HRMS (ESI) m/z: calcd. for $C_{22}H_{37}{}^{10}B_2ClNO_4{}^+$: 434.2664, Found: 434.2659 $[M+NH_4]^+$.

(*R*)-2,2'-(3-(3-Bromophenyl)but-1-ene-2,3-diyl)bis(4,4,5,5-tetramethyl-1,3,2-dioxaborolane) (3l)

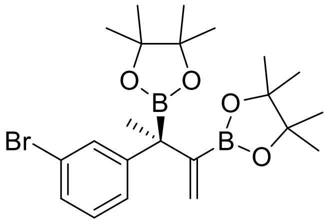

White solid, 80% yield, M.p. 97-99 °C. $[\alpha]_D^{20}$ = 29.0 (*c* 1.00, $CHCl_3$), 86% *ee* [determined by HPLC analysis using a Chiralcel ID-3 column; *n*-Hex/*i*-PrOH = 99:1, 0.7 mL/min, λ = 214 nm; t_R (major) = 5.05 min; t_R (minor) = 5.38 min]. ^{1}H NMR (400 MHz, $CDCl_3$) δ 7.52 (t, *J* = 1.6 Hz, 1H), 7.32-7.25 (m, 2H), 7.12 (t, *J* = 8.0 Hz, 1H), 5.85 (d, *J* = 2.8 Hz, 1H), 5.25 (d, *J* = 2.8 Hz, 1H), 1.48 (s, 3H), 1.24 (s, 6H), 1.22 (s, 6H), 1.20 (s, 6H), 1.19 (s, 6H) ppm; ^{13}C NMR (100 MHz, $CDCl_3$) δ 147.9, 131.6, 129.1, 128.2, 127.1, 127.0, 122.0, 83.6, 83.4, 24.8, 24.7, 24.4, 24.3, 22.2 ppm; ^{11}B NMR (128 MHz, $CDCl_3$) δ 33.2, 30.8 ppm. IR (neat) ν 2971, 1602, 1307, 1255, 1145, 1087, 914, 838, 778 cm^{-1}; ESI-MS m/z: 480.2 $[M+NH_4]^+$; HRMS (ESI) m/z: calcd. for $C_{22}H_{37}{}^{10}B_2BrNO_4^+$: 478.2159, Found: 478.2154 $[M+NH_4]^+$.

(*R*)-2,2'-(3-(3-Methoxyphenyl)but-1-ene-2,3-diyl)bis(4,4,5,5-tetramethyl-1,3,2-dioxaborolane) (3m)

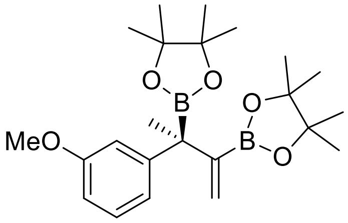

Colorless oil, 95% yield, $[\alpha]_D^{20}$ = 45.7 (*c* 0.68, $CHCl_3$), 94% *ee* [determined by HPLC analysis using a Chiralcel IC-3 column; *n*-Hex/*i*-PrOH = 99:1, 0.7 mL/min, λ = 214 nm; t_R (major) = 7.76 min; t_R (minor) = 8.28 min]. ^{1}H NMR (500 MHz, $CDCl_3$) δ 7.19-7.14 (m, 1H), 6.97-6.92 (m, 2H), 6.72-6.67 (m, 1H), 5.81 (d, *J* = 2.9 Hz, 1H), 5.20 (d, *J* = 2.9 Hz, 1H), 3.77 (s, 3H), 1.51 (s, 3H), 1.27-1.18 (m, 24H) ppm; ^{13}C NMR (126 MHz, $CDCl_3$) δ 159.2, 146.7, 128.4, 126.8, 121.0, 114.2, 110.8, 83.4, 83.3, 55.0, 24.89, 24.86, 24.4, 24.3, 22.4 ppm; ^{11}B NMR (128 MHz, $CDCl_3$) δ 33.2, 30.5 ppm. IR (neat) ν 2980, 1594, 1305, 1141, 961, 853, 699 cm^{-1}; ESI-MS m/z: 415.7 $[M+H]^+$; HRMS (ESI) m/z: calcd. for $C_{23}H_{37}{}^{10}B_2O_5^+$: 413.2894, Found: 413.2897 $[M+H]^+$.

(R)-2,2'-(3-(o-Tolyl)but-1-ene-2,3-diyl)bis(4,4,5,5-tetramethyl-1,3,2-dioxaborolane) (3n)

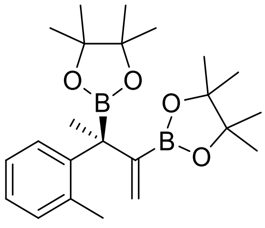

White solid, 91% yield, M.p. 67-69 °C. $[\alpha]_D^{20}$ = 34.2 (*c* 1.00, $CHCl_3$), 92% *ee* [determined by HPLC analysis using a Chiralcel OD-H column; *n*-Hex/*i*-PrOH = 99.9:0.1, 0.7 mL/min, λ = 214 nm; t_R (major) = 10.91 min; t_R (minor) = 14.04 min]. ^{1}H NMR (400 MHz, $CDCl_3$) δ 7.28 (d, *J* = 7.6 Hz, 1H), 7.17-7.12 (m, 1H), 7.10-7.07 (m, 2H), 5.61 (d, *J* = 2.8 Hz, 1H), 5.06 (d, *J* = 2.8 Hz, 1H), 2.21(s, 3H), 1.64 (s, 3H), 1.25-1.21 (m, 24H) ppm; ^{13}C NMR (100 MHz, $CDCl_3$) δ 143.5, 137.3, 130.5, 127.3, 125.6, 125.4, 125.3, 83.3, 82.8, 24.8, 24.5, 24.4, 23.1, 21.6 ppm; ^{11}B NMR (128 MHz, $CDCl_3$) δ 33.6, 31.1 ppm. IR (neat) ν 2977, 1463, 1301, 1142, 1086, 849, 730, 673 cm^{-1}; ESI-MS m/z: 416.2 $[M+NH_4]^+$; HRMS (ESI) m/z: calcd. for $C_{22}H_{40}{}^{10}B_2NO_4^+$: 414.3211, Found: 414.3209 $[M+NH_4]^+$.

(*R*)-2,2'-(3-(2-Methoxyphenyl)but-1-ene-2,3-diyl)bis(4,4,5,5-tetramethyl-1,3,2-dioxaborolane) (3o)

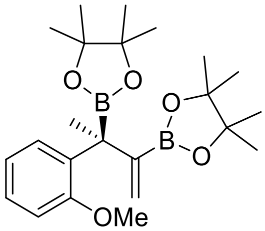

White solid, 95% yield, M.p. 102-104 °C. $[\alpha]_D^{20}$ = 23.9 (*c* 0.70, $CHCl_3$), 94% *ee* [determined by HPLC analysis using a Chiralcel IC-3 column; *n*-Hex/*i*-PrOH = 99:1, 0.7 mL/min, λ = 214 nm; t_R (minor) = 6.50 min; t_R (major) = 6.87 min]. ^{1}H NMR (500 MHz, $CDCl_3$) δ 7.21-7.13 (m, 2H), 6.91 (dt, *J* = 7.5, 1.1 Hz, 1H), 6.80 (dd, *J* = 8.2, 1.1 Hz, 1H), 5.65 (d, *J* = 3.0 Hz, 1H), 5.02 (d, *J* = 3.0 Hz, 1H), 3.74 (s, 3H), 1.55 (s, 3H), 1.27-1.22 (m, 24H) ppm; ^{13}C NMR (126 MHz, $CDCl_3$) δ 156.7, 135.4, 127.9, 126.5, 125.6, 120.8, 110.0, 82.8, 55.2, 24.85, 24.83, 24.64, 24.58, 21.9 ppm; ^{11}B NMR (128

MHz, $CDCl_3$) δ 33.3, 31.4 ppm. IR (neat) ν 2979, 1328, 1291, 1143, 1086, 846, 749 cm^{-1}; ESI-MS m/z: 437.5 $[M+Na]^+$; HRMS (ESI) m/z: calcd. for $C_{23}H_{37}{}^{10}B_2O_5{}^+$: 413.2894, Found: 413.2891 $[M+H]^+$.

(*R*)-2,2'-(3-(2-Chlorophenyl)but-1-ene-2,3-diyl)bis(4,4,5,5-tetramethyl-1,3,2-dioxaborolane) (3p)

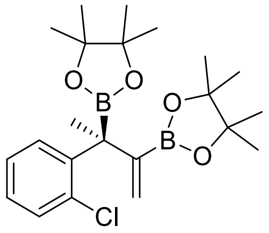

White solid, 90% yield, M.p. 105-107 °C. $[\alpha]_D^{20}$ = 20.0 (*c* 0.80, $CHCl_3$) 93% *ee* [determined by HPLC analysis using a Chiralcel ID-H column; *n*-Hex/*i*-PrOH = 99:1, 0.7 mL/min, λ = 214 nm; t_R (major) = 5.37 min ; t_R (minor) = 5.69 min]. ^1H NMR (500 MHz, $CDCl_3$) δ 7.33-7.28 (m, 2H), 7.21 (td, *J* = 7.6, 1.5 Hz, 1H), 7.11 (td, *J* = 7.6, 1.7 Hz, 1H), 5.72 (d, *J* = 2.7 Hz, 1H), 5.19 (d, *J* = 2.7 Hz, 1H), 1.63 (s, 3H), 1.26-1.21 (m, 24H) ppm; ^{13}C NMR (126 MHz, $CDCl_3$) δ 144.1, 134.8, 129.4, 129.2, 126.8, 126.5, 126.4, 83.4, 82.9, 24.8, 24.7, 24.60, 24.58, 22.5 ppm; ^{11}B NMR (128 MHz, $CDCl_3$) δ 32.8, 30.4 ppm. IR (neat) ν 2978, 1314, 1141, 1099, 847, 722 cm^{-1}; ESI-MS m/z: 419.6 $[M+H]^+$; HRMS (ESI) m/z: calcd. for $C_{22}H_{34}{}^{10}B_2O_4Cl^+$: 417.2399, Found: 417.2398 $[M+H]^+$.

(*R*)-2,2'-(3-(Naphthalen-2-yl)but-1-ene-2,3-diyl)bis(4,4,5,5-tetramethyl-1,3,2-dioxaborolane) (3q)

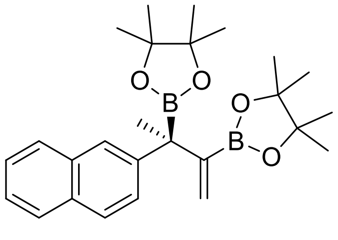

White solid, 98% yield, M.p. 136-138 °C. $[\alpha]_D^{20}$ = 41.9 (*c* 1.00, $CHCl_3$), 94% *ee* [determined by HPLC analysis using a Chiralcel OD-3+ IB-3 column; *n*-Hex/*i*-PrOH = 99:1, 0.4 mL/min, λ = 214 nm; t_R (minor) = 10.51 min; t_R (major) = 10.78 min]. ^1H

NMR (400 MHz, $CDCl_3$) δ 7.78-7.75 (m, 3H), 7.71 (d, *J* = 8.8 Hz, 1H), 7.55 (dd, *J* = 8.8, 2.0 Hz, 1H), 7.42-7.36 (m, 2H), 5.86 (d, *J* = 2.8 Hz, 1H), 5.19 (d, *J* = 2.8 Hz, 1H), 1.63 (s, 3H), 1.24 (s, 6H), 1.21-1.20 (m, 18H) ppm; ^{13}C NMR (100 MHz, $CDCl_3$) δ 142.6, 133.6, 131.7, 128.1, 127.8, 127.4, 127.2, 126.6, 126.1, 125.3, 124.9, 83.5, 83.3, 24.9, 24.8, 24.4, 24.3, 22.3 ppm; ^{11}B NMR (128 MHz, $CDCl_3$) δ 33.2, 31.3 ppm. IR (neat) ν 2979, 1308, 1137, 1092, 850, 746 cm^{-1}; ESI-MS m/z: 457.2 [M+Na]$^+$; HRMS (ESI) m/z: calcd. for $C_{26}H_{40}{}^{10}B_2NO_4^+$: 450.3211, Found: 450.3207 [M+NH$_4$]$^+$.

(*R*)-2,2'-(3-(Naphthalen-1-yl)but-1-ene-2,3-diyl)bis(4,4,5,5-tetramethyl-1,3,2-dioxaborolane) (3r)

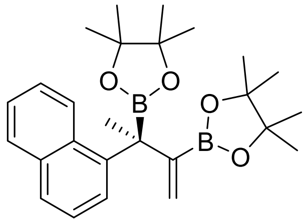

White solid, 86% yield, M.p. 104-106 °C. $[\alpha]_D^{20}$ = 26.3 (*c* 0.46, $CHCl_3$), 91% *ee* [determined by HPLC analysis using a Chiralcel IC-3+OD-3 column; *n*-Hex/*i*-PrOH = 99:1, 0.4 mL/min, λ = 214 nm; t_R (major) = 19.55 min; t_R (minor) = 20.63 min]. ^{1}H NMR (500 MHz, $CDCl_3$) δ 8.06-8.00 (m, 1H), 7.81-7.76 (m, 1H), 7.70-7.65 (m, 1H), 7.52-7.48 (m, 1H),7.48-7.42 (m, 1H), 7.39-7.31 (m, 2H), 5.66 (d, *J* = 2.8 Hz, 1H), 4.98 (d, *J* = 2.8 Hz, 1H), 1.80 (s, 3H), 1.28-1.07 (m, 24H) ppm; ^{13}C NMR (126 MHz, $CDCl_3$) δ 142.3, 134.0, 132.6, 128.3, 127.7, 127.2, 126.5, 125.7, 124.7, 124.6, 124.5, 83.6, 83.0, 24.86, 24.77, 24.6, 24.3, 23.6 ppm; ^{11}B NMR (128 MHz, $CDCl_3$) δ 34.5, 31.2 ppm. IR (neat) ν 2977, 1302, 1143, 1092, 848, 777 cm^{-1}; ESI-MS m/z: 457.5 [M+Na]$^+$; HRMS (ESI) m/z: calcd. for $C_{26}H_{40}{}^{10}B_2NO_4^+$: 450.3211, Found: 450.3208 [M+NH$_4$]$^+$.

(*R*)-2,2'-(3-(Furan-2-yl)but-1-ene-2,3-diyl)bis(4,4,5,5-tetramethyl-1,3,2-dioxaborolane) (3s)

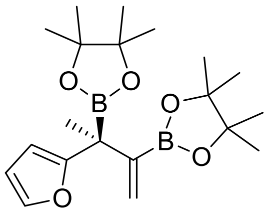

White solid, 93% yield, M.p.82-84 °C. $[\alpha]_D^{20}$ = 24.9 (*c* 0.50, $CHCl_3$), 88% *ee* [determined by HPLC analysis using a Chiralcel IC-3 column; *n*-Hex/*i*-PrOH = 99:1, 0.7 mL/min, λ = 214 nm; t_R (major) = 7.11 min; t_R (minor) = 10.45 min]. ^{1}H NMR (500 MHz, $CDCl_3$) δ 7.33-7.30 (m, 1H), 6.28 (dd, *J* = 3.2, 1.8 Hz, 1H), 6.15-6.10 (m, 1H), 5.80 (d, *J* = 2.7 Hz, 1H), 5.13 (d, *J* = 2.7 Hz, 1H), 1.47(s, 3H), 1.28-1.17 (m, 24H), ppm; ^{13}C NMR (126 MHz, $CDCl_3$) δ 158.8, 140.8, 126.9, 109.8, 105.8, 83.7, 83.4, 25.0, 24.7, 24.4, 24.3, 20.8 ppm; ^{11}B NMR (128 MHz, $CDCl_3$) δ 33.1, 30.6 ppm; IR (neat) ν 2978, 1315, 1140, 1102, 846, 722 cm^{-1}; ESI-MS m/z: 375.3 $[M+H]^+$; HRMS (ESI) m/z: calcd. for $C_{20}H_{33}{}^{10}B_2O_5{}^+$: 373.2581, Found: 373.2579 $[M+H]^+$.

(*R*)-2,2'-(3-(3,4,5-Trimethoxyphenyl)but-1-ene-2,3-diyl)bis(4,4,5,5-tetramethyl-1,3,2-dioxaborolane) (3t)

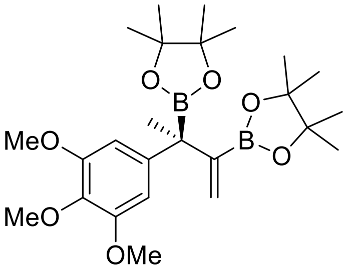

White solid, 86% yield, M.p.110-112 °C. $[\alpha]_D^{20}$ = 32.6 (*c* 1.00, $CHCl_3$), 90% *ee* [determined by HPLC analysis using a Chiralcel IC-3 column; *n*-Hex/*i*-PrOH = 95:5, 0.7 mL/min, λ = 214 nm; t_R (minor) = 15.23 min; t_R (major) = 17.72 min]. ^{1}H NMR (400 MHz, $CDCl_3$) δ 6.61 (s, 2H), 5.80 (d, *J* = 2.8 Hz, 1H), 5.20 (d, *J* = 2.8 Hz, 1H), 3.82 (s, 9H), 1.51 (s, 3H), 1.27-1.22 (m, 24H) ppm; ^{13}C NMR (100 MHz, $CDCl_3$) δ 152.3, 140.6, 135.6, 126.6, 105.6, 83.4, 83.2, 60.7, 55.8, 24.92, 24.88, 24.33, 24.31, 22.4 ppm; ^{11}B NMR (128 MHz, $CDCl_3$) δ 30.9 ppm. IR (neat) ν 2979, 1586, 1310, 1129, 1094, 845 cm^{-1}; ESI-MS m/z: 475.3 $[M+H]^+$; HRMS (ESI) m/z: calcd. for $C_{25}H_{41}{}^{10}B_2O_7{}^+$: 473.3106, Found: 473.3104 $[M+H]^+$.

(*R*)-2,2'-(3-(Benzo[d][1,3]dioxol-5-yl)but-1-ene-2,3-diyl)bis(4,4,5,5-tetramethyl-1,3,2-dioxaborolane) (3u)

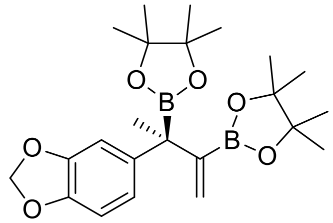

Pale yellow solid, 98% yield, M.p. 103-105 °C. $[\alpha]_D^{20}$ = 41.8 (*c* 0.91, $CHCl_3$), 94% *ee* [determined by HPLC analysis using a Chiralcel IC-3 column; *n*-Hex/*i*-PrOH = 99:1, 0.7 mL/min, λ = 214 nm; t_R (major) = 9.59 min; t_R (minor) = 10.81 min]. ^{1}H NMR (500 MHz, $CDCl_3$) δ 6.93 (d, *J* = 1.8 Hz 1H), 6.81-6.78 (m, 1H), 6.73-6.69 (m, 1H), 5.89 (s, 2H), 5.81 (d, *J* = 2.9 Hz, 1H), 5.24 (d, *J* = 2.8 Hz, 1H), 1.46 (s, 3H), 1.28-1.17 (m, 24H) ppm; ^{13}C NMR (126 MHz, $CDCl_3$) δ 147.1, 145.0, 139.2, 126.6, 121.3, 109.3, 107.5, 100.5, 83.4, 83.3, 24.84, 24.80, 24.4, 24.3, 22.7 ppm; ^{11}B NMR (128 MHz, $CDCl_3$) δ 33.5, 30.9 ppm; IR (neat) ν 2981, 1478, 1310, 1140, 1088, 1033, 932, 849, 816 cm^{-1}; ESI-MS m/z: 429.3$[M+H]^+$; HRMS (ESI) m/z: calcd. for $C_{23}H_{35}{}^{10}B_2O_6{}^+$: 427.2687, Found: 427.2684 $[M+H]^+$.

(*R*)-2,2'-(3-(4-(Prop-1-en-2-yl)phenyl)but-1-ene-2,3-diyl)bis(4,4,5,5-tetramethyl-1,3,2-dioxaborolane) (3v)

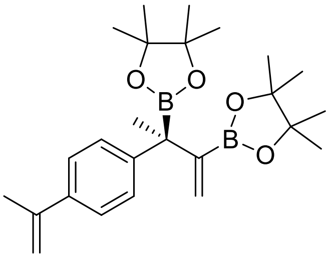

White solid, 99% yield, M.p. 84-86 °C. $[\alpha]_D^{20}$ = 38.2 (*c* 0.87, $CHCl_3$), 96% *ee* [determined by HPLC analysis using a Chiralcel IC-3 column; *n*-Hex/*i*-PrOH = 99:1, 0.7 mL/min, λ = 214 nm; t_R (major) = 5.55 min; t_R (minor) = 6.42 min]. ^{1}H NMR (500 MHz, $CDCl_3$) δ 7.41-7.36 (m, 2H), 7.35-7.29 (m, 2H), 5.83 (d, *J* = 2.9 Hz, 1H), 5.39-5.35 (m, 1H), 5.21 (d, *J* = 2.9 Hz, 1H), 5.04-4.99 (m, 1H), 2.17-2.12 (m, 3H), 1.52 (s, 3H), 1.30-1.15 (m, 24H) ppm; ^{13}C NMR (126 MHz, $CDCl_3$) δ 144.4, 143.1, 137.8,

128.3, 126.9, 124.8, 111.2, 83.4, 83.3, 24.9, 24.8, 24.4, 24.3, 22.4, 21.8 ppm; ^{11}B NMR (128 MHz, $CDCl_3$) δ 33.4, 31.4 ppm; IR (neat) ν 2978, 1307, 1143, 1093, 964, 849, 693 cm^{-1}; ESI-MS m/z: 425.4 $[M+H]^+$; HRMS (ESI) m/z: calcd. for $C_{25}H_{39}{}^{10}B_2O_4^+$: 423.3097, Found: 423.3102 $[M+H]^+$.

(+)-4,4,5,5-Tetramethyl-2-(1-(1-(4,4,5,5-tetramethyl-1,3,2-dioxaborolan-2-yl)-1,2,3,4-tetrahydronaphthalen-1-yl)vinyl)-1,3,2-dioxaborolane (3w)

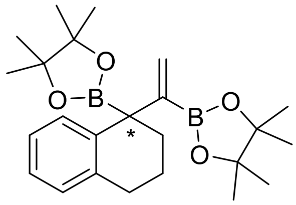

White solid, 98% yield, M.p.125-127 °C. $[\alpha]_D^{20}$ = 58.3 (*c* 0.82, $CHCl_3$), 91% *ee* [determined by HPLC analysis using a Chiralcel IC-3 column; *n*-Hex/*i*-PrOH = 99:1, 0.7 mL/min, λ = 214 nm; t_R (major) = 5.29 min; t_R (minor) = 6.21 min]. ^{1}H NMR (400 MHz, $CDCl_3$) δ 7.25-7.19 (m, 1H), 7.06-7.01 (m, 3H), 5.93 (d, *J* = 3.2 Hz, 1H), 4.88 (d, *J* = 3.2 Hz, 1H), 2.78-2.64 (m, 2H), 2.12-2.01 (m, 2H), 1.66-1.53 (m, 2H), 1.27 (s, 6H), 1.26 (s, 6H), 1.24 (s, 6H), 1.22 (s, 6H) ppm; ^{13}C NMR (100 MHz, $CDCl_3$) δ 138.0, 130.9, 130.1, 129.6, 125.1, 124.6, 83.4, 83.3, 30.2, 30.0, 25.3, 25.0, 24.3, 24.1, 17.9 ppm; ^{11}B NMR (128 MHz, $CDCl_3$) δ 33.4, 30.8 ppm. IR (neat) ν 2977, 1309, 1264, 1136, 851, 743, 662 cm^{-1}; ESI-MS m/z: 428.3 $[M+NH_4]^+$; HRMS (ESI) m/z: calcd. for $C_{24}H_{40}{}^{10}B_2NO_4^+$: 426.3211, Found: 426.3202 $[M+NH_4]^+$.

(+)-4,4,5,5-Tetramethyl-2-(1-(4-(4,4,5,5-tetramethyl-1,3,2-dioxaborolan-2-yl)chroman-4-yl)vinyl)-1,3,2-dioxaborolane (3x)

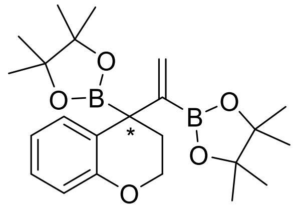

Pale yellow solid, 96% yield, M.p. 101-103 °C. $[\alpha]_D^{20}$ = 76.7 (*c* 0.81, $CHCl_3$), 90% *ee* [determined by HPLC analysis using a Chiralcel IC-3 column; *n*-Hex/*i*-PrOH = 99:1, 0.7 mL/min, λ = 214 nm; t_R (major) = 7.11 min; t_R (minor) = 8.72 min]. ^{1}H NMR (500

MHz, $CDCl_3$) δ 7.18-7.12 (m, 1H), 7.09-7.02 (m, 1H), 6.83-6.74 (m, 2H), 6.07 (d, *J* = 3.0 Hz, 1H), 5.10 (d, *J* = 3.0 Hz, 1H), 4.15-4.08 (m, 1H), 3.98-3.82 (m, 1H), 2.45-2.35 (m, 1H), 2.09-2.02 (m, 1H), 1.30-1.23 (m, 18H), 1.21 (s, 6H) ppm; ^{13}C NMR (126 MHz, $CDCl_3$) δ 155.1, 131.9, 131.2, 127.1, 123.1, 119.3, 117.2, 83.7, 83.5, 61.8, 29.5, 25.3, 25.0, 24.3, 24.1 ppm; ^{11}B NMR (128 MHz, $CDCl_3$) δ 33.1, 31.0 ppm. IR (neat) ν 2976, 1372, 1306, 1137, 1092, 854, 753 cm^{-1}; ESI-MS m/z: 435.4 $[M+Na]^+$; HRMS (ESI) m/z: calcd. for $C_{23}H_{35}{}^{10}B_2O_5^+$: 411.2738, Found: 411.2732 $[M+H]^+$.

(*R*)-2,2'-(3-Phenylpent-1-ene-2,3-diyl)bis(4,4,5,5-tetramethyl-1,3,2-dioxaborolane) (3y)

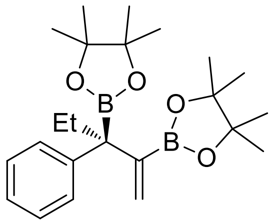

Colorless oil, 76% yield, $[\alpha]_D^{20}$ = 14.4 (*c* 1.00, $CHCl_3$), 72% *ee* [determined by HPLC analysis using a Chiralcel OD-H column; *n*-Hex/*i*-PrOH = 99.9:0.1, 0.7 mL/min, λ = 214 nm; t_R (minor) = 7.50 min; t_R (major) = 8.00 min]. ^{1}H NMR (400 MHz, $CDCl_3$) δ 7.39 (d, *J* = 7.6 Hz, 2H), 7.21 (t, *J* = 7.6 Hz, 2H), 7.10-7.06 (m, 1H), 5.94 (d, *J* = 2.8 Hz, 1H), 5.60 (d, *J* = 2.8 Hz, 1H), 2.11-1.97 (m, 2H), 1.20 (s, 12H), 1.09 (s, 6H), 1.07 (s, 6H), 0.81 (t, *J* = 7.6 Hz, 3H) ppm; ^{13}C NMR (100 MHz, $CDCl_3$) δ 144.4, 129.2, 127.4, 127.3, 124.8, 83.1, 83.0, 27.1, 24.6, 24.5, 24.4, 10.6 ppm; ^{11}B NMR (128 MHz, $CDCl_3$) δ 33.1, 30.8 ppm; IR (neat) ν 2976, 1600, 1345, 1303, 1140, 1105, 967, 850, 699 cm^{-1}; ESI-MS m/z: 399.2 $[M+H]^+$; HRMS (ESI) m/z: calcd. for $C_{23}H_{40}{}^{10}B_2NO_4^+$: 414.3211, Found: 414.3208 $[M+NH_4]^+$.

(+)-2,2'-(1-Cyclopropyl-1-phenylprop-2-ene-1,2-diyl)bis(4,4,5,5-tetramethyl-1,3,2-dioxaborolane) (3z)

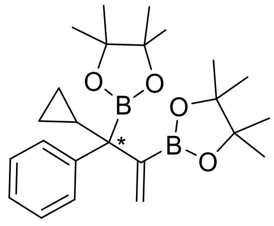

Colorless oil, 28% yield, $[\alpha]_D^{20}$ = 11.3 (*c* 1.00, $CHCl_3$), 34% *ee* [determined by HPLC analysis using a Chiralcel IC-3 column; *n*-Hex/*i*-PrOH = 99:1, 0.7 mL/min, λ = 214 nm; t_R (major) = 4.99 min; t_R (minor) = 5.35 min]. ^1H NMR (400 MHz, $CDCl_3$) δ 7.54-7.51 (m, 2H), 7.22-7.19 (m, 2H), 7.12-7.10 (m, 1H), 5.99 (d, *J* = 3.2 Hz, 1H), 5.78 (d, *J* = 2.8 Hz, 1H), 1.23-1.22 (m, 12H), 1.14-1.11 (m, 7H), 1.04 (s, 6H), 0.48-0.42 (m, 2H), 0.31-0.29 (m, 2H) ppm; ^{13}C NMR (100 MHz, $CDCl_3$) δ 144.1, 129.8, 127.4, 127.1, 125.0, 83.2, 83.1, 24.7, 24.6, 24.5, 24.2, 17.2, 2.6, 2.3 ppm; ^{11}B NMR (128 MHz, $CDCl_3$) δ 32.6, 31.1 ppm. IR (neat) ν 2981, 1351, 1304, 1138, 961, 855, 698 cm^{-1}; ESI-MS m/z: 411.2 $[M+H]^+$; HRMS (ESI) m/z: calcd. for $C_{24}H_{40}{}^{10}B_2NO_4^+$: 426.3208, Found: 426.3211 $[M+NH_4]^+$.

(-)-2,2'-(3-methylnon-1-ene-2,3-diyl)bis(4,4,5,5-tetramethyl-1,3,2-dioxaborolane) (3aa)

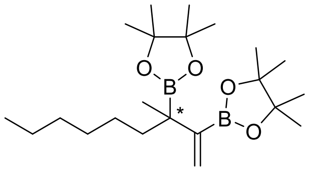

Colorless oil, 92% yield, $[\alpha]_D^{20}$ = -2.0 (c 0.60, $CHCl_3$), 67% ee [determined by HPLC analysis using a Chiralcel OX-3 column; *n*-Hex/*i*-PrOH = 99.5:0.5, 0.5 mL/min, λ = 214 nm; tR (minor) = 17.49 min; tR (major) = 19.88 min]. ^1H NMR (500 MHz, $CDCl_3$) δ 5.81 (d, *J* = 2.9 Hz, 1H), 5.51 (d, *J* = 2.9 Hz, 1H), 1.70-1.62 (m, 1H), 1.57-1.45 (m, 1H), 1.33-1.01 (m, 35H), 0.85 (t, *J* = 6.8 Hz, 3H) ppm; ^{13}C NMR (126 MHz, $CDCl_3$) δ 125.4, 83.3, 83.1, 36.0, 32.0, 30.3, 24.9, 24.8, 24.8, 24.7, 24.6, 22.8, 20.0, 14.2 ppm; ^{11}B NMR (128 MHz, $CDCl_3$) δ 33.8, 30.5 ppm. IR (neat) ν 2977, 2928, 1378, 1370, 1339, 1300, 1147, 968, 854, 723, 684, 671 cm^{-1}; ESI-MS m/z: 415.5 $[M+Na]^+$; HRMS (ESI) m/z: calcd. for $C_{22}H_{42}{}^{10}B_2NaO_4^+$: 413.3234, Found: 413.3237 $[M+Na]^+$.

(-)-2,2'-(4-ethyl-3-methylhex-1-ene-2,3-diyl)bis(4,4,5,5-tetramethyl-1,3,2-dioxaborolane) (3ab)

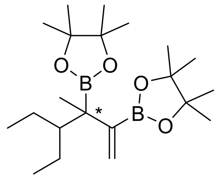

White soild, 95% yield, M.p. 65-68 °C. $[\alpha]_D^{20}$ = -6.0 (c 1.086, $CHCl_3$), 87% ee [determined by HPLC analysis using a Chiralcel OX-3 column; *n*-Hex/*i*-PrOH = 99.5:0.5, 0.5 mL/min, λ = 214 nm; tR (minor) = 14.84 min; tR (major) = 18.50 min]. ^{1}H NMR (500 MHz, $CDCl_3$) δ 5.74 (d, *J* = 2.7 Hz, 1H), 5.50 (d, *J* = 2.7 Hz, 1H), 1.86-1.77 (m, 1H), 1.50-1.15 (m, 28H), 1.01 (s, 3H), 0.96 (t, *J* = 7.5 Hz, 3H), 0.89 (t, *J* = 7.4 Hz, 3H) ppm; ^{13}C NMR (126 MHz, $CDCl_3$) δ 144.5, 143.0, 129.3, 128.6, 128.2, 127.6, 127.5, 125.6, 125.1, 83.3, 83.2, 36.8, 34.4, 28.2, 24.8, 24.7, 24.6, 24.6 ppm; ^{11}B NMR (128 MHz, $CDCl_3$) δ 33.0, 30.5 ppm. IR (neat) ν 2975, 2931, 1389, 1333, 1299, 1145, 1106, 966, 852, 703 cm^{-1}; ESI-MS m/z: 401.4 $[M+Na]^+$; HRMS (ESI) m/z: calcd. for $C_{21}H_{40}{}^{10}B_2O_4^+$: 399.3078, Found: 399.3078 $[M+Na]^+$.

(-)-2,2'-(3-Cyclohexylbut-1-ene-2,3-diyl)bis(4,4,5,5-tetramethyl-1,3,2-dioxaborolane) (3ac)

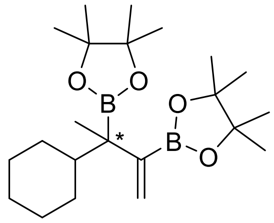

White solid, 92% yield, M.p.51-53 °C. $[\alpha]_D^{20}$ = -3.5 (*c* 0.64, $CHCl_3$), 91% *ee* [determined by HPLC analysis using a Chiralcel IC-3 + ID-3 column; *n*-Hex/*i*-PrOH = 99:1, 0.4 mL/min, λ = 214 nm; t_R (major) = 18.57 min; t_R (minor) = 20.88min]. ^{1}H NMR (500 MHz, $CDCl_3$) δ 5.76 (d, *J* = 2.8 Hz, 1H), 5.50 (d, *J* = 2.8 Hz, 1H), 1.91 (tt, *J* = 11.9, 2.8 Hz, 1H), 1.73–1.65 (m, 3H), 1.65–1.53 (m, 2H), 1.33–1.14 (m, 26H), 1.12–1.05 (m, 2H), 1.02 (s, 3H), 0.89–0.80 (m, 1H) ppm; ^{13}C NMR (126 MHz, $CDCl_3$) δ 125.6, 82.9, 82.8, 42.0, 30.1, 28.1, 27.3, 27.2, 27.0, 24.74, 24.69, 24.64, 24.63, 16.2

ppm; ^{11}B NMR (128 MHz, $CDCl_3$) δ 33.9, 30.7 ppm. IR (neat) ν 2977, 2921, 1337, 1299, 1141, 1092, 960, 853, 721, 674 cm^{-1}; ESI-MS m/z: 413.5 $[M+Na]^+$; HRMS (ESI) m/z: calcd. for $C_{22}H_{40}{}^{10}B_2NaO_4^+$: 411.3078, Found: 411.3071 $[M+Na]^+$.

(-)-2,2'-(3,4,4-trimethyl-5-phenylpent-1-ene-2,3-diyl)bis(4,4,5,5-tetramethyl-1,3,2-dioxaborolane) (3ad)

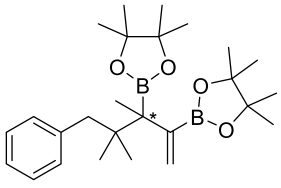

White soild, 81% yield, M.p.119-122 °C. $[\alpha]_D^{20}$ = -17.8 (c 3.4, $CHCl_3$), 89% ee [determined by HPLC analysis using a Chiralcel PC-3 column; Actone/H_2O = 80:20, 0.7 mL/min, λ = 214 nm; tR (minor) = 5.51 min; tR (major) = 5.96 min]. ^1H NMR (500 MHz, $CDCl_3$) δ 7.25-7.20 (m, 2H), 7.19-7.11 (m, 3H), 6.01 (d, J = 2.8 Hz, 1H), 5.71 (d, J = 2.8 Hz, 1H), 2.79-2.68 (m, 2H), 1.30-1.22 (m, 27H), 0.83 (s, 3H), 0.80 (s, 3H) ppm; ^{13}C NMR (126 MHz, $CDCl_3$) δ 140.9, 131.4, 129.8, 127.4, 125.4, 83.4, 82.9, 44.8, 38.5, 25.2, 25.1, 24.8, 24.7, 23.5, 23.3, 17.7 ppm; ^{11}B NMR (128 MHz, $CDCl_3$) δ 33.6, 31.0 ppm. IR (neat) ν 2977, 2929, 1371, 1273, 1317, 1295, 1273, 1143, 1083, 966, 864, 745, 704 cm^{-1}; ESI-MS m/z: 463.4 $[M+Na]^+$; HRMS (ESI) m/z: calcd. for $C_{26}H_{42}{}^{10}B_2O_4^+$: 461.3234, Found: 461.3237 $[M+Na]^+$.

(-)-2,2'-(1-phenyl-1-(p-tolyl)prop-2-ene-1,2-diyl)bis(4,4,5,5-tetramethyl-1,3,2-dioxaborolane) (3ae)

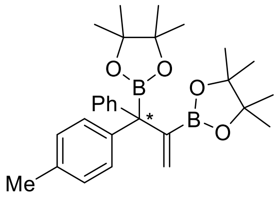

Colorless oil, 97% yield. $[\alpha]_D^{20}$ = -0.4 (c 1.76, $CHCl_3$), 8% ee [determined by HPLC analysis using a Chiralcel OD-3 column; *n*-Hex/*i*-PrOH = 100:0, 1.0 mL/min, λ = 214 nm; tR (minor) = 6.70 min; tR (major) = 7.29 min]. ^1H NMR (500 MHz, $CDCl_3$) δ 7.35-7.29 (m, 2H), 7.26-7.21 (m, 2H), 7.17-7.12 (m, 3H), 7.07-7.02 (m, 2H), 6.05 (d, J = 2.8

Hz, 1H), 5.24 (d, J = 2.8 Hz, 1H), 2.31 (s, 3H), 1.27 (d, J = 1.7 Hz, 12H), 1.11 (d, J = 7.2 Hz, 12H) ppm; ^{13}C NMR (126 MHz, $CDCl_3$) δ 144.4, 141.0, 134.6, 130.5, 130.3, 128.3, 128.2, 127.4, 125.2, 83.7, 83.3, 24.7, 24.6, 24.5, 24.4, 21.0 ppm; ^{11}B NMR (128 MHz, $CDCl_3$) δ 31.7 ppm. IR (neat) ν 2978, 2926, 1599, 1347, 1302, 1145, 1132, 967, 857, 844, 753, 702, 666 cm^{-1}; ESI-MS m/z: 483.3 $[M+Na]^+$; HRMS (ESI) m/z: calcd. for $C_{28}H_{39}{}^{10}B_2O_4^+$: 459.3098, Found: 459.3102 $[M+H]^+$.

(+)-2,2'-(1-phenyl-1-(o-tolyl)prop-2-ene-1,2-diyl)bis(4,4,5,5-tetramethyl-1,3,2-dioxaborolane) (3af)

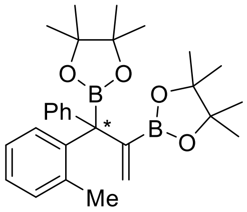

Colorless oil, 93% yield. $[\alpha]_D^{20}$ = 2.7 (c 4.84, $CHCl_3$), 75% ee [determined by HPLC analysis using a Chiralcel OD-3 column; *n*-Hex/*i*-PrOH = 100:0, 1.0 mL/min, λ = 214 nm; tR (major) = 8.30 min; tR (minor) = 9.97 min]. ^{1}H NMR (500 MHz, $CDCl_3$) δ 7.38-7.31 (m, 2H), 7.25-7.20 (m, 2H), 7.17-7.13 (m, 2H), 7.12-7.02 (m, 3H), 6.04 (d, J = 2.8 Hz, 1H), 5.27 (d, J = 2.8 Hz, 1H), 2.07 (s, 3H), 1.22 (d, J = 1.9 Hz, 12H), 1.12 (d, J = 14.2 Hz, 12H) ppm; ^{13}C NMR (126 MHz, $CDCl_3$) δ 143.30, 143.25, 138.3, 131.2, 131.0, 130.6, 129.3, 127.5, 125.9, 125.3, 125.1, 83.8, 83.3, 25.0, 24.8, 24.7, 24.6, 22.4 ppm; ^{11}B NMR (128 MHz, $CDCl_3$) δ 32.2 ppm. IR (neat) ν 2977, 2930, 1598, 1343, 1303, 1265, 969, 855, 843, 743, 743, 702, 666 cm^{-1}; ESI-MS m/z: 483.4 $[M+Na]^+$; HRMS (ESI) m/z: calcd. for $C_{28}H_{39}{}^{10}B_2O_4^+$: 459.3099, Found: 459.3102 $[M+H]^+$.

Crystal structural data of 3f

Single crystal of the product **3f** was obtained by recrystallization from CH_2Cl_2/*n*-hexane. X-ray diffractional data and the refinement were shown in Table S6. The absolute configuration of **3f** was determined to be (*R*) by X-ray crystallographic analysis (Figure *S1*).

Table 6. Crystal data and structure refinement for 3f.

Identification code	mo_dm16225_0m	
Empirical formula	C22 H31 B2 Br O4	
Formula weight	461.00	
Temperature	130 K	
Wavelength	0.71073 Å	
Crystal system	Monoclinic	
Space group	P 1 21 1	
Unit cell dimensions	a = 11.1006(17) Å	α= 90°.
	b = 6.9951(10) Å	β= 94.930(3)°.
	c = 30.827(5) Å	γ = 90°.
Volume	2384.8(6) $Å^3$	
Z	4	
Density (calculated)	1.284 Mg/m^3	
Absorption coefficient	1.747 mm^{-1}	
F(000)	960	
Crystal size	0.3 x 0.2 x 0.18 mm^3	
Theta range for data collection	1.841 to 30.591°.	
Index ranges	-12<=h<=15, -9<=k<=10, -44<=l<=43	
Reflections collected	24139	
Independent reflections	14501 [R(int) = 0.0379]	
Completeness to theta = 26.000°	99.9 %	
Absorption correction	Semi-empirical from equivalents	
Max. and min. transmission	0.7461 and 0.6136	
Refinement method	Full-matrix least-squares on F^2	
Data / restraints / parameters	14501 / 1 / 541	
Goodness-of-fit on F^2	0.944	
Final R indices [I>2sigma(I)]	R1 = 0.0494, wR2 = 0.0935	
R indices (all data)	R1 = 0.0995, wR2 = 0.1093	
Absolute structure parameter	0.020(6)	
Extinction coefficient	n/a	
Largest diff. peak and hole	0.544 and -0.461 e.$Å^{-3}$	

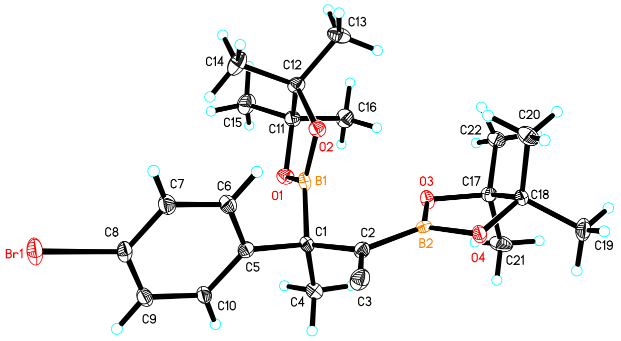

Figure S1. X-ray crystal structure of (*R*)-**3f**

The CIF files of **3f** can be obtained from the Cambridge Crystallographic Data Centre using deposition numbers 1517472. Copies of the data can be obtained, free of charge, on application to the CCDC, 12 Union Road, Cambridge CB2 1EZ, UK [fax: +44 (1223) 336 033; e-mail: deposit@ccdc.cam.ac.uk].

6. Procedure for a gram scale reaction

To a flame-dried Schlenk tube was charged $Pd_2(dba)_3$ (33.7mg, 0.1 mol %) and **L8** (30.1 mg, 0.25 mol %) and the mixture was purged with nitrogen three times. Cyclohexane (50 mL) was added and the resulting mixture was stirred at rt for 0.5 h. To the mixture was charged substrate **1a** (4.8 g, 36.8 mmol, 1.0 equiv) and B_2pin_2 (11.2 g, 44.2 mmol, 1.2 equiv) in one portion. The resulting mixture was stirred at rt for 72 h and then quenched with saturated NH_4Cl solution (50 mL). EtOAc (100 mL) was added and the organic layer was separated, washed with brine, dried over sodium sulfate, and concentrated. The crude product was purified by flash chromatography on silica gel to afford product **3a** (13.6 g, 97 %, 94 % ee) as white solid.

7. Computational studies for Pd-catalyzed asymmetric diboration

7.1. Computational results and discussion

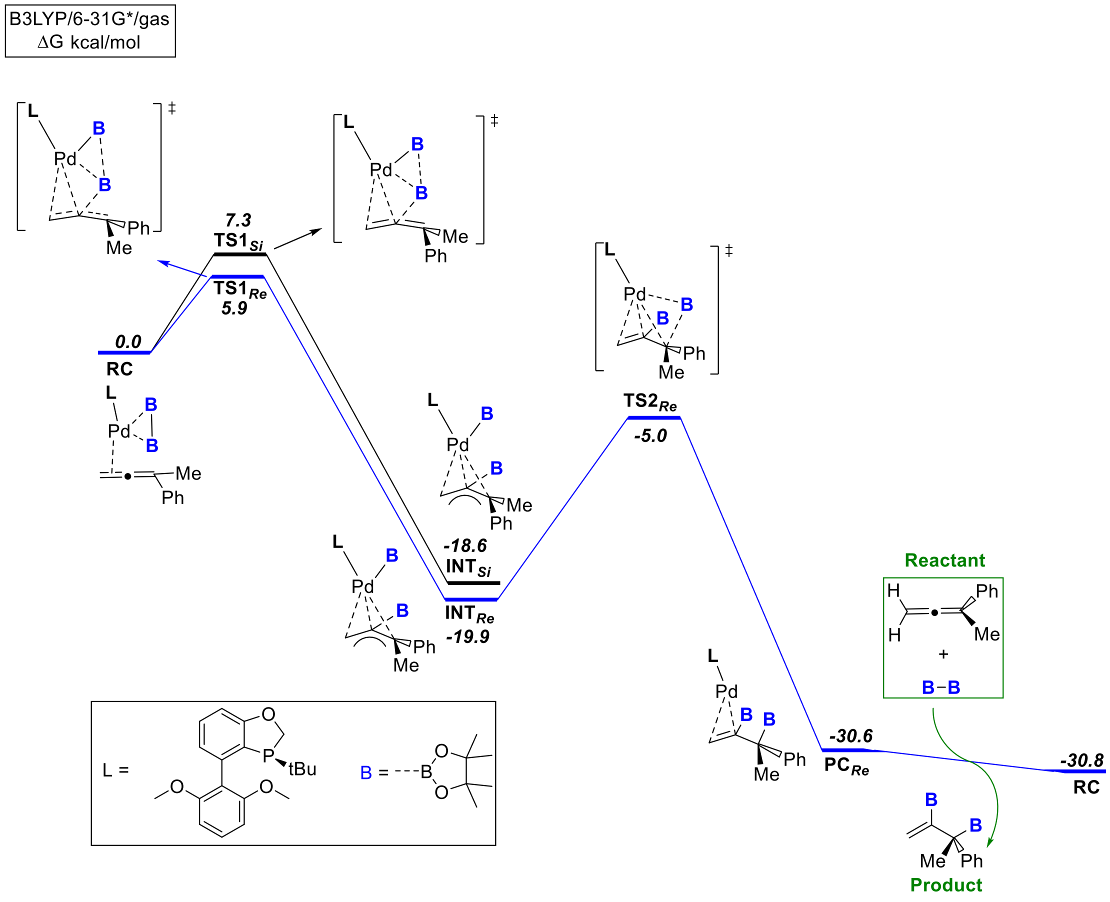

Figure S2. Free-energy profile (in kcal/mol) for the Pd-catalyzed asymmetric diboration of allene with the chiral phosphorus ligand (**L**=(S)-BI-DME) at the B3LYP-D3/6-31G(d)+SDD level.[6]

As shown in Figure S2, the Pd-allene-diborane reaction complex **RC** was chosen as the starting point of the catalytic cycle. The reaction initiates with oxidative-addition of diboration concerted with allene insertion (via **TS1*Re*** or **TS1*Si***), in which the boryl group is favored to migrate to the middle carbon of the allene. It is noted that the initial oxidative boryl migration transition state **TS1*Re*** is higher in free energy than reductive elimination transition state (**TS2*Re***) by 10.9 kcal/mol, and thus, the initial oxidative boryl migration process is an irreversible and stereo-determining step. Our calculations also show that **TS1*Re*** is kinetically lower in free energy than **TS1*Si*** by 1.4 kcal/mol (Figure S3). These results qualitatively agree to the experimental observation (94%

ee→ ΔG: 2.1 kcal/mol, according to Eyring Equation). The formation of the resultant η^3 Pd(II)-allyl intermediates **INT*Re*** and **INT*Si***, in which the other boryl group is above the allyl plane, are computed to be thermodynamically favorable (-18.6~-19.9 kcal/mol). Similarly, the formation of the (R)-form intermediate **INT*Re*** is thermodynamically lower in free energy than **INT*Si*** by 1.3 kcal/mol.

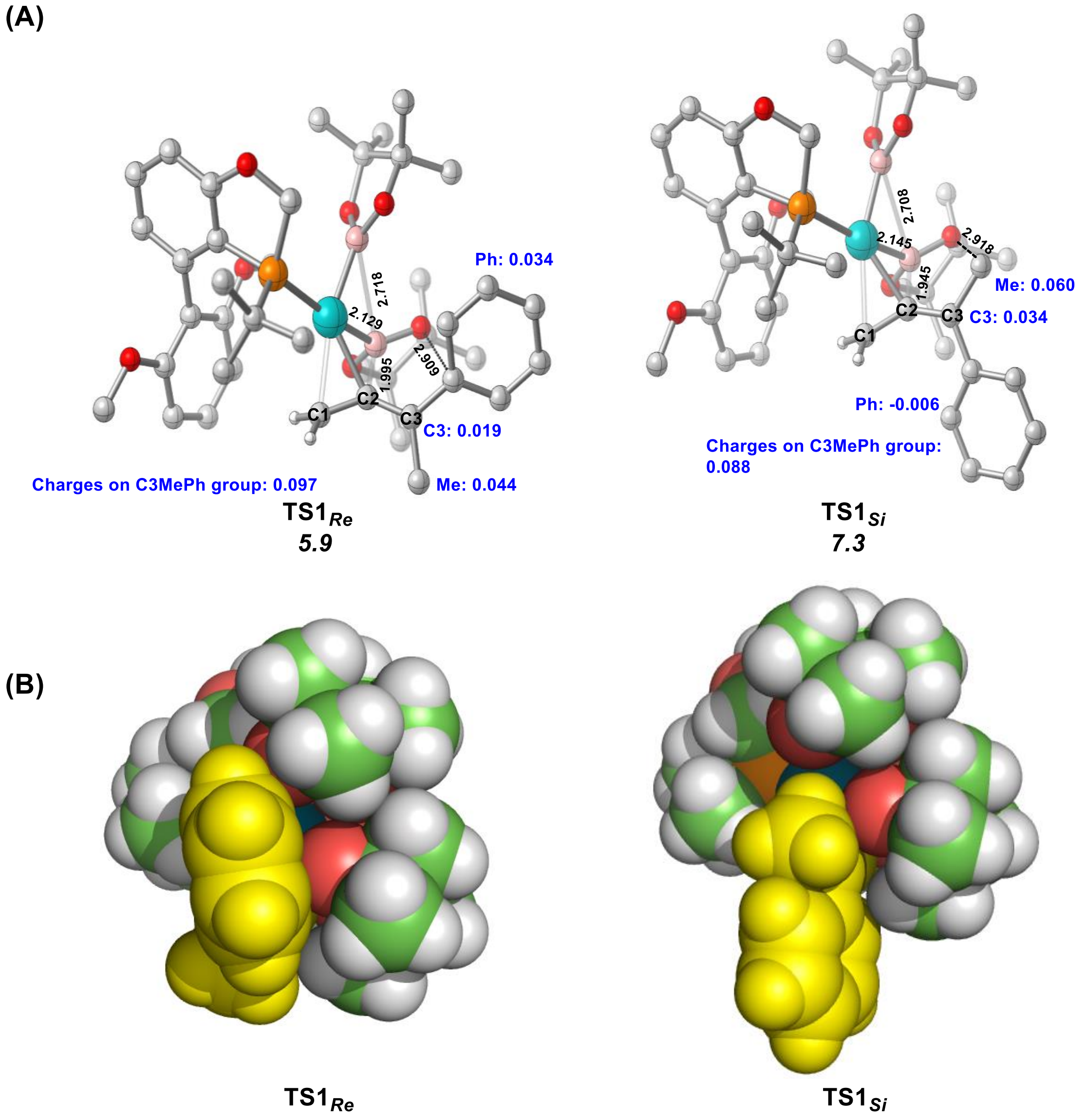

Figure S3. (A) 3D model of the optimized geometry of **TS1*Re*** and **TS1*Si*** with the selected structural parameters, at the B3LYP-D3/6-31G(d)+SDD level.[1] Bold italic numbers below are relative free Gibbs energies in kcal/mol. Bond lengths are given in Å; blue bold numbers are NPA charges on the specified groups or atoms. (B) van der Waals representation models of **TS1*Re*** and **TS1*Si*** (more dispersion interactions for **TS1*Re***).

As shown in Scheme S1, our distortion/interaction analysis[7] reveals that a larger interaction energy in **TS1*Re*** than **TS1*Si*** is the key factor to favor the observed (*R*)-form

product via **TS1*Re***. We further found that dispersion interaction plays an essential role in the enantioselectivity (Scheme S3 and Figure S3B). When excluding the dispersion contribution, **TS1*Re*** becomes unfavorable to **TS1*Si*** by 2.5 kcal/mol in electronic energy, that should lead to the opposite enantioselectivity. What's more, when the phenyl group of the allene in these two transition states is simply substituted by a hydrogen atom without the geometry optimization, single-point energy calculations also suggests that **TS1*Re-H*** is 1.1 kcal/mol higher in electronic energy than **TS1*Si-H***. These results suggest that dispersion interaction (between the phenyl group on the allene and the two boryl group, see belows) favors the observed enantioselectivity. As shown in Scheme S3, 7 groups are divided in **TS1*Re*** and **TS1*Si*** for the detailed dispersion analysis. **TS1*Re*** was computed to gain 4.0 kcal/mol more dispersion interactions than **TS1*Si***, prompting the stability of **TS1*Re***. Also, the dispersion contribution between the large phenyl (Ph) group and the two boryl groups (B1, B2) are the major contributor: their dispersion interactions contribute -4.8 kcal/mol. Whereas, the dispersion interactions between the small Me group and the two boryl groups are smaller (1.7 kcal/mol).

(A)

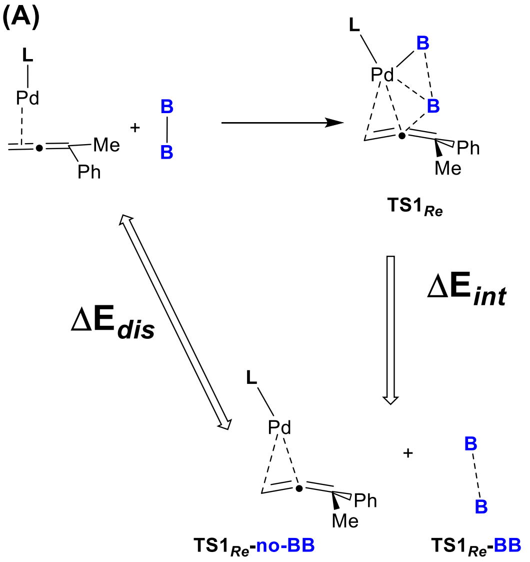

(B)

Entry	ΔE_{dis} (kcal/mol)	ΔE_{int} (kcal/mol)	ΔE_{dis} + ΔE_{int} (kcal/mol)
**TS1*Re***	112.4	-130.1	-17.7
**TS1*Si***	111.0	-127.2	-16.2
ΔΔE	+1.4	-2.9	-1.5

Scheme S1. (A).The calculated distortion energy (**E*dis***) and interaction energy (**E*int***) of **TS1*Re*** as an example. **(B).** The distortion energy (**E*dis***) and interaction energy (**E*int***) are calculated for **TS1*Re*** and **TS1*Si*** at the B3LYP-D3/6-31G(d)+SDD level. [1]

Single-Point Energy Calculation

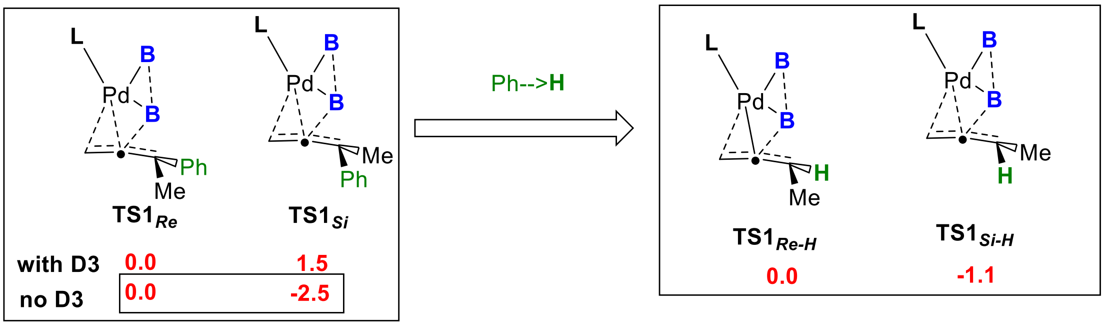

Scheme S2. (A). Relative electronic energies of **TS1*Re*** and **TS1*Si*** in the presence of dispersion (with D3) and in the absence of dispersion (no D3). **(B).** Single-point energy calculation for substituting the Ph group of the allene by hydrogen atom in **TS1*Re*** and **TS1*Si*** at the B3LYP-D3/6-31G(d)+SDD level. [1]

Natural population analysis (Figure S3-A) suggests that the total charges on the Ph group of the allene is slightly increased from **RC** (0.022 e) to **TS1*Re*** (0.034 e; v.s. -0.006 e in **TS1*Si***). Such increasing charge on the Ph group of the allene in **TS1*Re*** and decreasing charge on the Ph group of the allene in **TS1*Si*** might explain the observed slightly lower ee values with an electron-withdrawing group on the Ph group (e.g. CF_3), and explain the observed slightly higher ee values with an electron-donating group on the Ph group (e.g. OMe).

(A)

Interacting groups (i--j)	E_{disp} for **TS1$_{Re}$** (kcal/mol)	E_{disp} for **TS1$_{Si}$** (kcal/mol)	$\Delta E_{disp(Re\text{-}Si)}$ (kcal/mol)
1--1	-12.94	-12.92	-0.02
2--1	-5.44	-5.42	-0.02
2--2	-13.03	-13.03	0.00
3--1	-3.96	-2.33	**-1.63**
3--2	-3.26	-0.11	**-3.15**
3--3	-2.64	-2.64	0.00
4--1	-1.13	-1.90	**0.77**
4--2	-0.05	-0.97	**0.92**
4--3	-1.78	-1.79	0.01
4--4	-0.05	-0.05	0.00
5--1	-0.18	-0.18	0.00
5--2	-0.39	-0.38	-0.01
5--3	-0.51	-0.20	-0.31
5--4	-0.11	-0.20	0.09
5--5	-4.05	-4.06	0.01
6--1	-4.15	-4.30	0.15
6--2	-2.98	-2.87	-0.11
6--3	-0.12	-0.16	0.04
6--4	-0.06	-0.05	-0.01
6--5	-3.46	-3.40	-0.06
6--6	-7.72	-7.72	0.00
7--1	-6.60	-6.61	0.01
7--2	-8.30	-8.03	-0.27
7--3	-4.05	-3.24	-0.81
7--4	-1.55	-1.91	0.36
7--5	-11.24	-11.22	-0.02
7--6	-12.22	-12.28	0.06
7--7	-9.33	-9.29	-0.04
**Total**	**-121.30**	**-117.26**	**-4.04**

(B)

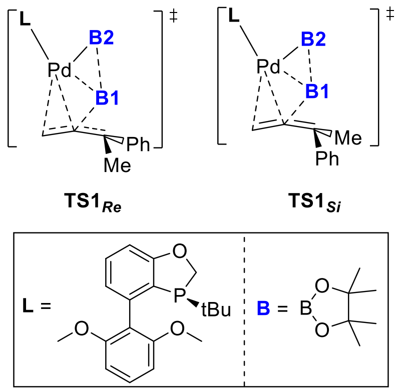

(C)

group entry	group symbol
1	**B1**
2	**B2**
3	Ph
4	Me
5	tBu
6	2,6-di-OMe-Ph
7	Other atoms

Scheme S3. (A). Dispersion interactions analysis among fragments in **TS1*Re*** and **TS1*Si***. **(B)**. Representative structures of **TS1*Re*** and **TS1*Si***. **(C)**. Fragment table.

Finally, reductive elimination via **TS2*Re*** proceeds from **INT*Re*** to produce the product complex **PC*Re*** with a computed barrier of about 14.9 kcal/mol. This step is also exergonic by 10.7 kcal/mol. A thermodynamically favorable ligand-exchange process with allene and diborate reagent takes place to release the final product and regenerate **RC** for the next catalytic cycle. Our proposed catalytic cycle is qualitatively similar to that reported by the Morken group (the initial oxidative addition of the diborane as the rate-determining step),[8] but we found a concerted mechanism for the first step, the first computational insight on the origin of the enantioselectivity (such as molecular details and an important role of the dispersion effect) and the final reductive elimination step as the rate-determining step in this study.

7.2 Computational details

All the optimization and frequency calculations were performed at B3LYP-D3[9,10] method (with SDD basis set and its ECP[10] for Pd and 6-31G(d) basis set for the other atoms).[11] Harmonic vibrational frequency calculations (at 298.15 K) are performed to ensure that one imaginary frequency for all transition states and no imaginary frequencies for all intermediates. IRC calculations were carried out to ensure that the transition states connect to their corresponding reactant complex and product complex. Gaussian 09 software package[12] was used in all the DFT calculations. The 3D models were generated with CYL view[13] and Pymol[14]. Dispersion analysis were done by DFTD3 Program.[15]

7.3 Cartesian coordinates and energies of all the optimized structure

RC

Opt @ B3LYP/6-31G(d)/SDD in gas phase
SCF Done: E(RB3LYP) = -2642.57728517 A.U.
Zero-point correction= 0.920743 (Hartree/Particle)
Sum of electronic and thermal Free Energies= -2641.743734 A.U.

C,-3.984445557,-2.8269916234,-1.9547501602
H,-4.3504905972,-2.844022411,-2.9884172952
H,-3.4536641303,-3.7597005769,-1.7491288588
H,-4.8647489561,-2.7954366044,-1.298316277
C,-3.0777563485,-1.6268490876,-1.7224596781
C,-1.9245776578,-1.8369150369,-1.0852469309
C,-0.9040895265,-2.6391754638,-0.6876978299
H,-0.7540498936,-2.9281655535,0.3520633088
H,-0.3114373426,-3.1737538856,-1.4263231422
B,-1.9798826364,0.2060380113,0.7949708097
O,-3.0680702923,1.034479363,0.602323787
O,-2.1170323747,-0.5287759071,1.9709892358
Pd,-0.3793887962,-0.3963992443,-0.3947222456
B,-0.3706167772,1.613588863,0.1564478632
O,-0.1485651728,2.2267139819,1.3794785075
O,-0.3692302962,2.5563093988,-0.8686284878
C,-3.485659334,-0.3773029742,2.4362229167
C,-3.8977489093,0.9935844788,1.7952046317
C,0.2535384905,3.601988341,1.1406713395
C,-0.3582022022,3.8841412454,-0.2760666058
C,-5.3568058485,1.1001727841,1.3633936404
H,-5.5273322925,2.075060716,0.8961316366
H,-6.0266358471,1.0051609316,2.2262781076
H,-5.6095852505,0.3359076582,0.6267428164
C,-3.5016964202,2.1960541476,2.6605254366
H,-4.0895977845,2.2402090696,3.5833494942
H,-3.6835147572,3.1160925586,2.0972286033
H,-2.4377546165,2.1569252088,2.9100780106
C,-4.2796417761,-1.5650324758,1.8784477966
H,-5.3177883843,-1.5567979972,2.2276037093
H,-3.8058902373,-2.4942448072,2.2106251812

H,-4.2694344884,-1.5553710201,0.785483259
C,-3.4786909705,-0.4113017023,3.9616516623
H,-3.155680977,-1.4000910541,4.304244322
H,-4.4817372416,-0.2231612356,4.3617686748
H,-2.7917145456,0.3305458003,4.3753381368
C,0.4710407911,4.8110500019,-1.161187638
H,-0.0068795803,4.9058861705,-2.1417215396
H,1.4850720241,4.4344908827,-1.3142803914
H,0.5372257298,5.8110053695,-0.7167121343
C,-1.8174062896,4.3524628776,-0.2335795827
H,-2.2147755354,4.362733663,-1.252626423
H,-1.9105281327,5.3584768338,0.1897362158
H,-2.4253925349,3.6527869328,0.3433591559
C,1.7883904099,3.615997335,1.162710738
H,2.1315957523,3.1986839152,2.1139228378
H,2.187336545,4.6309529951,1.0598713789
H,2.2047359041,2.9923180149,0.3672771787
C,-0.2986827851,4.4729381218,2.2651477809
H,-0.0747691918,5.5301761595,2.0815071638
H,0.165140896,4.1872469206,3.2152755942
H,-1.3792140732,4.3563147083,2.3667850261
C,-3.5579280677,-0.2991217612,-2.1731782158
C,-4.9328338711,-0.0115974851,-2.2242919561
C,-2.6582925569,0.7115196503,-2.5542684537
C,-5.3880643955,1.2532061145,-2.5971505967
H,-5.6573496966,-0.7723782426,-1.9493460066
C,-3.1111346554,1.9734128341,-2.9272736499
H,-1.5916958106,0.5110973229,-2.5293063947
C,-4.4791132819,2.2542061444,-2.9457149059
H,-6.4560636478,1.4562724805,-2.6125085813
H,-2.3863924704,2.737553214,-3.1923299838
H,-4.8342283074,3.2402752908,-3.2342405736
C,5.7597496527,0.4631141611,0.9343391952
C,4.947240733,-0.5485805063,1.4541689489
C,3.7125759937,-0.8485635906,0.8654169466
C,3.3227996383,-0.1258162694,-0.2748984146
C,4.1315775501,0.9167347168,-0.7566663495
C,5.3597837171,1.2173803796,-0.1695044866
C,2.8032123025,-1.8672065529,1.4672540119
C,3.0146005381,-3.2456982967,1.298007106
C,2.0967621462,-4.1826442533,1.791580849
C,0.97783551,-3.7257541346,2.4898380239
C,0.7733371434,-2.3705201064,2.7331194825
C,1.7052970243,-1.4425586522,2.2424852672
P,1.8447237848,-0.3054135378,-1.3324642159
C,2.2986491538,1.3322951985,-2.1279526507
O,3.6820058323,1.6285945471,-1.8313101045
C,2.3580742788,-1.5381681797,-2.7019990597
C,1.2023479811,-1.5498399949,-3.7214186828
C,2.5393104811,-2.9284620752,-2.0719371114
C,3.6760087568,-1.152631515,-3.3979515466
H,6.712249623,0.6820775175,1.4098115064
H,5.2604296438,-1.1046437383,2.3320613694
H,5.9668079958,2.024045394,-0.5669858787
H,0.2552361358,-4.4471780789,2.8628911167
H,1.6633533888,2.1184388571,-1.7191500616
H,2.1816472814,1.3165988917,-3.213696684

H,1.0663991822,-0.5711965641,-4.1984406756
H,1.4191645091,-2.2741460006,-4.5176789371
H,0.2495562595,-1.8262430708,-3.2585173767
H,3.4324701046,-2.9547053762,-1.4444956744
H,2.6568488487,-3.6783340701,-2.8662474701
H,1.6909485617,-3.222637202,-1.449930778
H,3.9324817044,-1.9247199688,-4.135960612
H,3.614072278,-0.1992014312,-3.9305566106
H,4.5010654176,-1.0884433516,-2.6815054327
H,2.2455948168,-5.2452295519,1.645285625
H,-0.1098684866,-2.0344938831,3.2599711812
O,4.163281469,-3.5826421612,0.632632799
O,1.6387065111,-0.1079892915,2.4818526144
C,4.4005637641,-4.9511596112,0.3468758248
H,3.6050981039,-5.3750835718,-0.2809447264
H,5.3457236905,-4.9814764945,-0.1987163049
H,4.4932512817,-5.546376925,1.2651623212
C,0.7425246741,0.3492750601,3.4941199177
H,0.9557340494,1.4101108488,3.6139476538
H,-0.2965418531,0.2292105504,3.1827329158
H,0.9277834069,-0.1839634767,4.4370607494

TS1$_{Re}$
Opt @ B3LYP/6-31G(d)/SDD in gas phase
SCF Done: E(RB3LYP) = -2642.56948295 A.U.
Zero-point correction= 0.920730 (Hartree/Particle)
Sum of electronic and thermal Free Energies= -2641.734291 A.U.

C,-3.8696677324,-2.2713287892,-1.9029678704
H,-4.1342754016,-2.3810409061,-2.9618175244
H,-3.3171874267,-3.1553344317,-1.5772608903
H,-4.8141833981,-2.2474691759,-1.3400800673
C,-3.0537467395,-1.0132519122,-1.6766228069
C,-1.9475559224,-1.1051716201,-0.8801946516
C,-0.961163482,-2.0679912497,-0.6665390776
H,-0.6585847956,-2.3707120283,0.332917182
H,-0.7035296647,-2.743427184,-1.4795612191
B,-2.0648772885,0.2578986855,0.5157370898
O,-3.0661580723,1.2104829228,0.4422202086
O,-2.0759572255,-0.3965493676,1.749630604
Pd,-0.2160137116,0.0932729096,-0.5643198778
B,-0.2060562461,2.1231697831,-0.1565380539
O,-0.1021886119,2.7402341854,1.0813943947
O,-0.2148099307,3.0760416622,-1.1735240321
C,-3.3597678302,-0.120780717,2.3698610946
C,-3.7601774561,1.2526865353,1.7139758907
C,0.224906108,4.1394186456,0.8709868399
C,-0.3436256785,4.3927477107,-0.5674126154
C,-5.2512284811,1.4218330624,1.4297793396
H,-5.4198298293,2.3890729151,0.9461694006
H,-5.8342488218,1.3919090669,2.3578526213
H,-5.6183312444,0.6479136285,0.7527315
C,-3.2246827186,2.4643471246,2.4845698913
H,-3.7106475438,2.5690380129,3.4604677512
H,-3.4300194949,3.3678596623,1.9031032353
H,-2.1427625404,2.3931700273,2.6179073338

C,-4.2935999503,-1.2727917171,1.9755298932
H,-5.2804242232,-1.1726912654,2.4397817691
H,-3.8461876486,-2.2168982263,2.3013472033
H,-4.4159567835,-1.3161816274,0.8900623545
C,-3.1679393845,-0.0896998704,3.883231732
H,-2.8635209007,-1.0810477852,4.235417697
H,-4.1027984355,0.1794549453,4.3880608433
H,-2.3958338617,0.6256816949,4.1745070916
C,0.4439837576,5.3947632096,-1.4055922459
H,-0.0035187159,5.4738554968,-2.4018767252
H,1.4862778496,5.0899706628,-1.5268829668
H,0.4239792933,6.3885743197,-0.942976265
C,-1.837412213,4.7378157586,-0.5750770037
H,-2.1993841558,4.7315241574,-1.6067265141
H,-2.0282672769,5.7268579304,-0.1444584011
H,-2.4062143355,3.9834678448,-0.0257950073
C,1.7538425926,4.2533234399,0.9545489048
H,2.0851099602,3.8625905004,1.9215758463
H,2.0890751362,5.2921384167,0.8640352751
H,2.2422188761,3.6579105273,0.177846199
C,-0.4197162444,4.9694970058,1.977105158
H,-0.2480216381,6.0389146321,1.8077625645
H,0.0225804973,4.7038090377,2.9432811804
H,-1.4944340826,4.7927829881,2.0385568353
C,-3.5567522207,0.2415062787,-2.2560091939
C,-4.9385017434,0.4603780798,-2.426448911
C,-2.6719775975,1.2571784259,-2.6662204289
C,-5.4152319538,1.6596184138,-2.949558503
H,-5.6484210863,-0.3032272818,-2.1232374364
C,-3.1494272019,2.4533103625,-3.1960532289
H,-1.6034741141,1.105285205,-2.5599285688
C,-4.5222602859,2.664091114,-3.3358609997
H,-6.4863085741,1.8123713593,-3.0562742483
H,-2.4396842441,3.2184239037,-3.4970429718
H,-4.8954737235,3.5973140452,-3.749963705
C,5.9476493494,0.6409714768,0.8542021558
C,5.0753419211,-0.3214625988,1.3700398288
C,3.8170198198,-0.5331656178,0.7924077263
C,3.4628922261,0.2307754573,-0.3322737697
C,4.3382993455,1.2174678749,-0.816597942
C,5.5890716762,1.4299238688,-0.2389801075
C,2.867322714,-1.5161312144,1.38871024
C,3.0553974749,-2.9011493271,1.2456846961
C,2.12683097,-3.8131225219,1.7638787315
C,1.0183787205,-3.3242844856,2.4560556666
C,0.829542372,-1.9607088825,2.6627970843
C,1.769202942,-1.0572152423,2.1431725539
P,1.9839784372,0.1516008794,-1.3945987917
C,2.5424049656,1.7531646593,-2.1949572237
O,3.9382043867,1.9580481003,-1.8913858691
C,2.3890778874,-1.1357058321,-2.7453929865
C,1.2469192832,-1.0584354712,-3.7763425101
C,2.419024019,-2.5279598564,-2.095280803
C,3.7460251441,-0.8743026005,-3.4234095596
H,6.9173074544,0.7904295622,1.3217626592
H,5.3611591084,-0.9108808455,2.2352395294
H,6.2461709538,2.1949783156,-0.6389837145

H,0.2868120487,-4.0261123718,2.8481280238
H,1.9571628057,2.5839774181,-1.798080129
H,2.4298491973,1.7362245459,-3.2811674478
H,1.1745662821,-0.0689041213,-4.2446917543
H,1.4262010048,-1.7882755732,-4.5767325926
H,0.279889651,-1.2849877877,-3.3156158025
H,3.2932853785,-2.6377703252,-1.4503906531
H,2.4758136463,-3.2944435603,-2.8803856464
H,1.5250498414,-2.7175041798,-1.495642897
H,3.9359528452,-1.6647565572,-4.1616965514
H,3.7800324004,0.0830496647,-3.9523274636
H,4.566184708,-0.8911339206,-2.6983334407
H,2.2573517115,-4.8805475904,1.6369272005
H,-0.0513186871,-1.6033185819,3.1790172011
O,4.1884961498,-3.2692090129,0.5693720579
O,1.7113818994,0.2861376118,2.3303267326
C,4.3978016897,-4.6462504751,0.3022985692
H,3.5845422672,-5.0662148353,-0.3050081765
H,5.3329905822,-4.7013575139,-0.2583412537
H,4.4957530382,-5.2281198749,1.2285554701
C,0.740224509,0.8020491457,3.2380463432
H,0.9302152314,1.8721845119,3.2903862205
H,-0.2715303628,0.6402457722,2.8612398265
H,0.8590853642,0.3431066021,4.2297375154

TS1*Si*
Opt @ B3LYP/6-31G(d)/SDD in gas phase
SCF Done: E(RB3LYP) = -2642.56710619 A.U.
Zero-point correction= 0.921049 (Hartree/Particle)
Sum of electronic and thermal Free Energies= -2641.732068 A.U.

C,-2.9255837148,0.6076692598,2.6182902628
H,-3.9583048189,0.9625966051,2.5165704847
H,-2.7267879732,-0.1261320627,1.8375153132
H,-2.8439894404,0.0877395596,3.5829357014
C,-1.9330560229,1.7430744054,2.5711780327
C,-0.8913623828,1.7406795235,1.6862847647
C,0.4496292765,2.1090467518,1.6962281366
H,0.9101669197,2.639850006,0.866728413
H,0.9737933059,2.1329161496,2.6489251886
B,-1.6167374929,1.4530846886,-0.0958022683
O,-2.9836659457,1.2459046813,-0.228499298
O,-1.1856216994,2.5043152399,-0.9061226286
Pd,-0.0346975225,0.1610209983,0.5601164792
B,-1.1257444622,-1.1646263131,-0.5865753735
O,-1.3474191396,-1.1461311953,-1.9566536582
O,-1.7128290311,-2.290413707,-0.0135380459
C,-2.3622973991,3.2473076123,-1.317797674
C,-3.4915354551,2.1544303992,-1.2392725694
C,-1.8997157477,-2.4320518102,-2.3484035776
C,-2.555609948,-2.9256923396,-1.0138725255
C,-4.8503319002,2.6719052843,-0.7721079513
H,-5.5600651885,1.8398362663,-0.7220395789
H,-5.2462991438,3.4179391196,-1.4711319472
H,-4.7868260645,3.1200487034,0.2218469413
C,-3.6454461987,1.3499255433,-2.5345380328

H,-4.0191897317,1.9720026538,-3.354629846
H,-4.3659446744,0.5446343017,-2.3629842092
H,-2.6975216314,0.8894048149,-2.8223136138
C,-2.545242497,4.3831457584,-0.301696948
H,-3.389635355,5.0287281532,-0.5653967168
H,-1.6345472647,4.9900770287,-0.2853593265
H,-2.7031686176,3.990919298,0.7065581516
C,-2.1058813359,3.8190922585,-2.7091892666
H,-1.2855014253,4.5430724508,-2.6620748985
H,-2.9949290254,4.3360211853,-3.0879596379
H,-1.8269011737,3.0373997992,-3.4191688562
C,-2.5206572758,-4.4353664716,-0.7967696925
H,-2.9693890468,-4.678587622,0.1718069117
H,-1.4980512475,-4.8202937522,-0.8007025287
H,-3.0909258757,-4.9529368582,-1.577237154
C,-3.9739961349,-2.3826199336,-0.7978503077
H,-4.2880099671,-2.6188302667,0.2237970643
H,-4.6921807964,-2.8293486149,-1.4940938976
H,-3.9890526378,-1.2946598008,-0.9081321332
C,-0.718786525,-3.2988661397,-2.8086614782
H,-0.1901417489,-2.776684923,-3.6120987221
H,-1.0519321254,-4.2720048158,-3.1851182289
H,-0.003012607,-3.4630495392,-1.9980543917
C,-2.8722170619,-2.2238209244,-3.5055122493
H,-3.3588098913,-3.1688246756,-3.7740456807
H,-2.3298151937,-1.8591282858,-4.3843684671
H,-3.6419866943,-1.4921843225,-3.2570948573
C,-2.1461586504,2.8933660342,3.4750529244
C,-1.4580127385,4.1163067997,3.3113017246
C,-3.0838968665,2.8146063475,4.5267840498
C,-1.6852445149,5.1926565078,4.1625366901
H,-0.7506395468,4.2176732334,2.4961546397
C,-3.305071856,3.8929839993,5.3826041335
H,-3.6391564492,1.8977628686,4.6892634877
C,-2.6081942259,5.0891504304,5.2081180267
H,-1.143107434,6.1216912281,4.0049458762
H,-4.0274072486,3.7953301191,6.189111762
H,-2.7835892644,5.9306853972,5.8728413619
C,4.9517384762,-2.7170071274,-2.1214003502
C,4.757220443,-1.3359269038,-2.0321073029
C,3.775541106,-0.8030347639,-1.1867548484
C,3.0074754318,-1.6901526173,-0.4147396929
C,3.1929857334,-3.0768700786,-0.5428817166
C,4.1674974032,-3.6069965983,-1.3869524999
C,3.5270516041,0.6664901574,-1.1463797138
C,4.4206174024,1.5475279079,-0.5143681351
C,4.1485887015,2.9195638135,-0.4377240396
C,2.9807464619,3.4074738974,-1.0251565406
C,2.0987702468,2.570032545,-1.7021891882
C,2.3852019076,1.1987130955,-1.7776001099
P,1.7446227029,-1.3568281949,0.8576535008
C,1.3129617722,-3.1815961546,0.8222440647
O,2.4002263386,-3.900635876,0.2033373375
C,2.7017190343,-1.1960754073,2.5014145821
C,1.6402406959,-1.1523672158,3.6174917397
C,3.4947165852,0.1203346548,2.475607903
C,3.6793353004,-2.3619952905,2.7326157531

H,5.7162121986,-3.1081375615,-2.7875994271
H,5.3603366282,-0.6587680786,-2.6282282087
H,4.2920482922,-4.6822290711,-1.4609528559
H,2.7569789033,4.4690078938,-0.9568279577
H,0.4003688279,-3.328240343,0.2418633847
H,1.1591909641,-3.5943658319,1.8217060465
H,1.0497451137,-2.0756977873,3.6642318301
H,2.1357867421,-1.0293068284,4.5895504269
H,0.9471293757,-0.3160649203,3.4775639614
H,4.3152292541,0.0674971633,1.7565943693
H,3.9243399003,0.3042693911,3.4698696213
H,2.8629145677,0.9729134554,2.2135994121
H,4.2132267227,-2.2005575554,3.6785395535
H,3.1760499228,-3.3312872003,2.8002865782
H,4.4256478921,-2.422541344,1.933869677
H,4.82517092,3.59895614,0.0655668309
H,1.1847356518,2.9657091605,-2.1231753859
O,5.5422202809,0.9625313502,0.0113272921
O,1.620236899,0.3008890978,-2.4506068982
C,6.445638876,1.7736531084,0.7441185581
H,5.9591922451,2.2305308933,1.6165960081
H,7.2392387575,1.1043311624,1.0819565288
H,6.8804895011,2.5636466205,0.1171650539
C,0.5694954515,0.7921754888,-3.2797021371
H,0.1517459489,-0.0846774891,-3.7701049026
H,-0.2108201653,1.2668941907,-2.6820154282
H,0.9648951971,1.4974064005,-4.0243716188

INT$_{Si}$
Opt @ B3LYP/6-31G(d)/SDD in gas phase
SCF Done: E(RB3LYP) = -2642.61022440 A.U.
Zero-point correction= 0.924139 (Hartree/Particle)
Sum of electronic and thermal Free Energies= -2641.773314 A.U.

C,-3.8549505435,0.5050044014,0.758860015
H,-4.6609309382,1.1775220061,1.0861917041
H,-3.8330027988,0.5087612001,-0.3319169851
H,-4.1071512007,-0.5042773955,1.0953925631
C,-2.5226127925,0.9293241,1.3610257117
C,-1.7357540348,1.99088072,0.7713148555
C,-0.4720823137,2.3547946748,1.2916085542
H,0.1413514667,3.0511209705,0.7279941761
H,-0.2236699175,2.2452433428,2.3413852672
B,-2.1667333808,2.6850408078,-0.5643437698
O,-3.4749220964,2.9119171198,-0.9169594682
O,-1.2769465005,3.221114868,-1.4705174892
Pd,-0.6002706128,0.0834242389,0.6537835536
B,-1.484045215,-1.6458635836,0.0088949507
O,-1.6361792901,-1.957845114,-1.3355857533
O,-1.9555955657,-2.6918564725,0.8000218109
C,-2.0371515538,4.0646980369,-2.379736628
C,-3.4822974571,3.4560404428,-2.2641709657
C,-1.9251373573,-3.3790431482,-1.443135658
C,-2.5941147118,-3.6791465427,-0.0583401823
C,-4.6206784992,4.4654085173,-2.3757969186
H,-5.5802493789,3.9505255328,-2.2663305834

H,-4.6058471092,4.9580424068,-3.3547285998
H,-4.5570468556,5.2283387637,-1.5971700431
C,-3.7221602316,2.2747540749,-3.2132558283
H,-3.7970132956,2.6025889679,-4.2552252626
H,-4.6592121906,1.7830592122,-2.9348489211
H,-2.9196944539,1.535322302,-3.1341604737
C,-1.9368269176,5.4935406766,-1.8333986363
H,-2.4337266944,6.2133131881,-2.4920062096
H,-0.880387592,5.7663134391,-1.7506852658
H,-2.3835910324,5.5623555107,-0.8367138662
C,-1.4135532173,3.98230512,-3.7696774248
H,-0.4134742174,4.4275998726,-3.7556530865
H,-2.0206907156,4.5336663682,-4.4963922431
H,-1.3191234553,2.9482126643,-4.1075269018
C,-2.313466849,-5.0666567933,0.5109715533
H,-2.8027549031,-5.1720209073,1.4849883745
H,-1.2427846534,-5.2330922957,0.650483481
H,-2.7053026226,-5.8451305532,-0.1540097518
C,-4.0978518419,-3.3833720852,-0.0361471802
H,-4.4478427344,-3.400533477,1.0008568019
H,-4.664572305,-4.1271249726,-0.6065047476
H,-4.3057158936,-2.3912513459,-0.4473463719
C,-0.5789786645,-4.0918468253,-1.6299094471
H,-0.0586139781,-3.6477440652,-2.4834715593
H,-0.7122095574,-5.1633398461,-1.8156413151
H,0.0613188761,-3.9692449521,-0.7522851712
C,-2.8203689258,-3.6038078062,-2.6573998551
H,-3.1374268796,-4.6515294447,-2.7182864673
H,-2.2682271034,-3.3626348589,-3.5719246942
H,-3.7093850768,-2.969926056,-2.6212410355
C,-2.439719752,0.6723555738,2.8442915778
C,-2.3854693009,1.741987922,3.7534927266
C,-2.4712010788,-0.6355701394,3.3640333979
C,-2.328956893,1.5166703735,5.1304427767
H,-2.3839862331,2.7595832471,3.3742229023
C,-2.4147657566,-0.860139829,4.7386641088
H,-2.503063694,-1.4758662625,2.678391246
C,-2.335490647,0.2136070644,5.6304472181
H,-2.2838726979,2.3629348786,5.8117576983
H,-2.4257354712,-1.8804029773,5.1150221653
H,-2.2860475015,0.0362179012,6.7016996537
C,4.1252914507,-3.3957572204,-1.7240711904
C,4.0094477179,-2.027792612,-1.9841796922
C,3.1929728459,-1.2126779831,-1.1905831005
C,2.4994428439,-1.7945854216,-0.1158733849
C,2.6264247455,-3.1716330312,0.1258207657
C,3.4339860247,-3.9860316573,-0.6678884183
C,3.0782743263,0.2413404193,-1.4915963553
C,4.1351295585,1.1203832387,-1.2019581974
C,4.0208795871,2.4936707282,-1.4493774475
C,2.8290740164,2.9803011688,-1.987146196
C,1.7703599835,2.1345957919,-2.3045117307
C,1.9055939738,0.7583223565,-2.0711454565
P,1.385085717,-1.0352142809,1.1163200418
C,1.0962051906,-2.7562467006,1.8449439995
O,1.9347920763,-3.7148896348,1.1659379947
C,2.4796486406,-0.2304042436,2.4629144394

C,1.5993041466,-0.0956877726,3.7229767909
C,2.9178591603,1.1693536747,2.000854671
C,3.7285321778,-1.0807120363,2.7522344672
H,4.7603214965,-4.0111065535,-2.355979157
H,4.5534907616,-1.5790882621,-2.8090892028
H,3.5061711671,-5.0465167072,-0.4503951483
H,2.724096437,4.0479934424,-2.1625265089
H,0.0565277764,-3.0538803041,1.7106766995
H,1.3425855845,-2.7892782724,2.908746759
H,1.3007674055,-1.0645527474,4.1374788845
H,2.1651219221,0.4305195003,4.5026394969
H,0.6847600533,0.4726637813,3.5253285719
H,3.6551722452,1.1068589065,1.2004473956
H,3.3819353209,1.6988748293,2.8440462589
H,2.0729395632,1.7630052005,1.6431690287
H,4.3121287161,-0.6083924142,3.5538395116
H,3.4843912122,-2.0971711641,3.0802051554
H,4.3667190663,-1.1527053406,1.8654016194
H,4.8276891747,3.1767431519,-1.2142205698
H,0.8459216216,2.5426786302,-2.6856533741
O,5.233973957,0.5366158927,-0.6264719774
O,0.9566565097,-0.1635588331,-2.3784870935
C,6.3150157657,1.3687059139,-0.2404735218
H,6.0055796701,2.1100184475,0.5089737834
H,7.0610487405,0.7027605352,0.1975898728
H,6.7547815492,1.8888502617,-1.1022621226
C,-0.2409130535,0.2768091702,-3.0080119694
H,-0.8539926769,-0.6145115251,-3.1187619616
H,-0.7691060629,0.9981435894,-2.3790064739
H,-0.0239612968,0.7297503288,-3.9865560399

INT*Re*
Opt @ B3LYP/6-31G(d)/SDD in gas phase
SCF Done: E(RB3LYP) = -2642.61172433 A.U.
Zero-point correction= 0.923357 (Hartree/Particle)
Sum of electronic and thermal Free Energies= -2641.775462 A.U.

C,-2.3895260726,0.3848828539,-3.1917015523
H,-1.7037095435,-0.3777694671,-3.5664840477
H,-3.3746068642,0.1780566619,-3.6384507458
H,-2.0367504851,1.3521065543,-3.5609998363
C,-2.4744453266,0.3982747766,-1.668753328
C,-2.4627099679,-0.8473780674,-0.9338159264
C,-1.7785343408,-1.9928112646,-1.386612772
H,-1.7341582258,-2.862167801,-0.7376505519
H,-1.5827762622,-2.1838706919,-2.4379630473
B,-3.1748950689,-1.0307080373,0.4510705827
O,-4.4819421903,-0.6815306571,0.6668489847
O,-2.6151498229,-1.6946882526,1.523160006
Pd,-0.3592757999,-0.1029780687,-1.0758411548
B,0.0749815073,1.8920862065,-1.0820207505
O,0.1772111247,2.67537769,0.0505609869
O,0.2594142109,2.6392509118,-2.2336541709
C,-3.7075099505,-2.0386818135,2.4245884043
C,-4.7861928859,-0.9547933503,2.0613770673
C,0.6505357908,3.9946707719,-0.3414205487

C,0.2712582131,4.0500569773,-1.866287013
C,-6.234610264,-1.4233597831,2.1569529415
H,-6.902578454,-0.6048028087,1.8714655028
H,-6.4774109092,-1.7227157532,3.182975125
H,-6.4282624312,-2.2651560093,1.4887198837
C,-4.600033982,0.3587125949,2.8299253679
H,-4.8981293068,0.2517254184,3.8782136198
H,-5.2115653546,1.1345654901,2.3618353096
H,-3.5598730169,0.6943680956,2.7920738848
C,-4.1353248983,-3.4649170973,2.0601355029
H,-4.9172221448,-3.8360605362,2.7308217543
H,-3.2655805696,-4.1246046443,2.1373212176
H,-4.5057046664,-3.5103473645,1.0313129827
C,-3.2028624725,-1.9880028469,3.8632842185
H,-2.475836012,-2.7888913192,4.031931189
H,-4.0317553881,-2.1307505663,4.5656691978
H,-2.7186710397,-1.0347457918,4.087210133
C,1.2843041679,4.7636036009,-2.7577407028
H,0.9377978446,4.740145162,-3.7962275741
H,2.2623973524,4.2788140436,-2.7161210424
H,1.3962666199,5.8125330301,-2.4591605274
C,-1.141670161,4.5857104108,-2.1213292218
H,-1.4026015617,4.4093035102,-3.169703628
H,-1.2038986896,5.6617209842,-1.9249962321
H,-1.8801563548,4.0713188528,-1.5023971551
C,2.162605599,4.0080914476,-0.0914289686
H,2.3517489827,3.7265587348,0.9487599718
H,2.5926063597,4.9996750628,-0.272065286
H,2.6704561482,3.2838633398,-0.7323645845
C,-0.0504977929,5.0286107709,0.5353146024
H,0.1979222404,6.0466619848,0.213406513
H,0.2785646195,4.9109175446,1.5734550297
H,-1.1349941495,4.9015684634,0.5061991052
C,-3.191878931,1.5824181871,-1.0935410681
C,-4.1321768242,2.3039703266,-1.8502335322
C,-2.9397434325,2.034937086,0.2136622349
C,-4.7912865181,3.4165568769,-1.3219547128
H,-4.3594670782,1.9992288966,-2.8659372287
C,-3.5979175411,3.138305936,0.7479520442
H,-2.1701144793,1.5406421172,0.7930895025
C,-4.5309936386,3.8424029381,-0.0195470833
H,-5.5095300834,3.9530079875,-1.9371681247
H,-3.3633610763,3.4631707275,1.7588124686
H,-5.0347117219,4.7151343708,0.3873960777
C,5.3565859984,1.0592644032,1.1343513458
C,4.5645246194,0.0876814999,1.7503736028
C,3.4694361625,-0.4770283804,1.0840675635
C,3.191159582,-0.0645400718,-0.2293848858
C,3.9987494932,0.9150036802,-0.8281577766
C,5.0816222668,1.4877811074,-0.1624836287
C,2.6287348697,-1.4852833961,1.7856157207
C,3.1036725979,-2.7864942776,2.0217673731
C,2.3128251446,-3.7321168099,2.6870527095
C,1.035040748,-3.3608709046,3.1086953365
C,0.5397521509,-2.0772794847,2.8998605213
C,1.3467442454,-1.135252161,2.2464107696
P,1.8709046792,-0.5795705551,-1.3854908763

C,2.6547348554,0.5360941925,-2.7013608382
O,3.6867136864,1.3313479991,-2.0880562917
C,2.3088240624,-2.328028068,-2.0305881835
C,1.6827632743,-2.457714348,-3.4347077295
C,1.665121805,-3.3838706693,-1.1160382451
C,3.8307251904,-2.5413093476,-2.0843168197
H,6.1971468408,1.4892281476,1.6724636647
H,4.787122729,-0.240320937,2.7606348395
H,5.6790329603,2.2435045888,-0.6614394428
H,0.4105409521,-4.0944910956,3.6122995213
H,1.9166762965,1.2194207648,-3.1190856323
H,3.1120294926,-0.0538251898,-3.501344897
H,2.1421393223,-1.7892724493,-4.1706873044
H,1.8212721031,-3.4841301978,-3.7979277717
H,0.6062342029,-2.2538740392,-3.4112894575
H,2.0896208994,-3.3703526138,-0.1122234036
H,1.8369802887,-4.3816483596,-1.5424222628
H,0.5869391783,-3.2274300827,-1.0262373021
H,4.0446163979,-3.5235494353,-2.5270166902
H,4.3397548472,-1.7876105401,-2.6963210064
H,4.2687363013,-2.5164390146,-1.0816655084
H,2.6689851649,-4.7395208111,2.8628986727
H,-0.4619597193,-1.8207572828,3.2120493833
O,4.3510937766,-3.0496639514,1.5203951783
O,0.9857614907,0.151434277,2.0158388152
C,4.8584023968,-4.3690394383,1.6315642376
H,4.2105634049,-5.0942100378,1.1204554084
H,5.8362466647,-4.353274619,1.1464302944
H,4.9796858506,-4.6705036075,2.6808564597
C,-0.2337830775,0.6364243461,2.5648588601
H,-0.303072305,1.668704327,2.2241301501
H,-1.089388417,0.0632875345,2.1953066457
H,-0.2108365046,0.5916419041,3.6635835862

TS2*Re*
Opt @ B3LYP/6-31G(d)/SDD in gas phase
SCF Done: E(RB3LYP) = -2642.59045374 A.U.
Zero-point correction= 0.923595 (Hartree/Particle)
Sum of electronic and thermal Free Energies= -2641.751743 A.U.

C,2.0981825618,0.9160386992,2.9416433146
H,1.0977017686,1.0761552204,3.3473826312
H,2.5738802042,0.1098214663,3.516934428
H,2.6486089802,1.844825989,3.0966959549
C,2.0305076446,0.531254871,1.4554634788
C,1.3586597589,-0.8242246909,1.2812680422
C,0.2811260947,-1.2400437063,2.0280345595
H,-0.1466056472,-2.226450095,1.8742089802
H,-0.1399584222,-0.6687269903,2.8513534008
B,2.1082322998,-1.9396608183,0.4621927785
O,2.9344544672,-2.8032158841,1.1346115786
O,2.0604276193,-2.1922695542,-0.8865082901
Pd,-0.1383416821,0.7156041806,0.4470146315
B,1.2202325905,2.2803310813,0.6672250689
O,1.7150573466,2.8232119789,-0.510101024
O,1.2018198773,3.225369227,1.6809077502

C,2.8661361304,-3.3738674802,-1.1660085134
C,3.7127821302,-3.5468761558,0.160585319
C,2.2705413659,4.1366116617,-0.255568128
C,1.7526421918,4.4774228406,1.2130747978
C,3.8454714347,-4.9858120455,0.656265944
H,4.4119128802,-4.9942894614,1.5924797619
H,4.3832057664,-5.6009570521,-0.0741347227
H,2.8713472995,-5.4406734376,0.850050887
C,5.0920360255,-2.8816828941,0.1070150984
H,5.7623851588,-3.4025553803,-0.5850622979
H,5.5343373955,-2.9114511902,1.107305155
H,5.018272961,-1.8344450199,-0.1905734674
C,1.8908072121,-4.5277921865,-1.421765825
H,2.4214188131,-5.4587603407,-1.6444975564
H,1.2559286179,-4.2849404626,-2.2792865483
H,1.2427719598,-4.6936185715,-0.5554567832
C,3.6865833758,-3.0866471261,-2.4215873688
H,3.0118493592,-2.9388848085,-3.2714679341
H,4.3507003181,-3.9263817655,-2.6544675285
H,4.286005233,-2.1819066821,-2.3033566019
C,0.6262082919,5.5146050684,1.2657210869
H,0.2482701575,5.5755555835,2.2911174748
H,-0.20532165,5.2305854355,0.6193891188
H,0.9759499028,6.5078396792,0.9647661892
C,2.8544555767,4.8833462556,2.1967782525
H,2.4153705713,5.0213298906,3.1901189655
H,3.3292249544,5.8241135917,1.8966150439
H,3.623947672,4.1120097804,2.2733639548
C,1.7582001352,5.0757961315,-1.3496422239
H,2.1111033439,4.7175865236,-2.3218624889
H,2.1324029538,6.0953681261,-1.2038791454
H,0.6669403144,5.1058627408,-1.3820128107
C,3.7953410593,4.0122327774,-0.3710974874
H,4.2885248706,4.9748933341,-0.1974163393
H,4.0463126605,3.6689490596,-1.3785170254
H,4.1951286383,3.2756401809,0.3279025018
C,3.3605093915,0.5995834824,0.7323101244
C,4.5752200927,0.7577538185,1.4212121856
C,3.4204860856,0.5193660269,-0.6723768862
C,5.7924657564,0.8424094395,0.7379443042
H,4.581691645,0.812697004,2.5040163708
C,4.6311194164,0.5941455745,-1.3551440471
H,2.4955451251,0.4357465907,-1.230098708
C,5.8295436764,0.7636014423,-0.6539275122
H,6.7134959038,0.966669763,1.3021034028
H,4.6360825556,0.5421036725,-2.4412281864
H,6.7744046116,0.8356132569,-1.1857896546
C,-5.4674430464,1.234751006,-3.0009649239
C,-4.9377097454,0.0634550816,-2.4523125193
C,-3.8533851298,0.1164264279,-1.5667283697
C,-3.3348953414,1.3718040178,-1.2173706938
C,-3.8442523203,2.5315706964,-1.8197608917
C,-4.9186427989,2.4838799213,-2.7060930659
C,-3.1981572397,-1.1306021132,-1.0860084435
C,-3.8156193151,-2.0017169259,-0.1739381508
C,-3.1594992744,-3.1542155044,0.2797836045
C,-1.8804119997,-3.4336090916,-0.2014320652

C,-1.2487521105,-2.6016174382,-1.1215838469
C,-1.9137840642,-1.452933667,-1.5668757146
P,-2.0713799534,1.8184327368,0.0272547813
C,-2.0397848841,3.5218922707,-0.7562428033
O,-3.2623261716,3.7244097808,-1.4917776861
C,-3.0747237905,2.0910413786,1.6296955658
C,-2.1552789201,2.8227063631,2.6259047642
C,-3.4463427359,0.7039413745,2.1862385819
C,-4.3593967292,2.9059385505,1.4016447747
H,-6.3058238613,1.1721359678,-3.6897442219
H,-5.3491876121,-0.9034376611,-2.7237296002
H,-5.2978257043,3.3993380385,-3.1481896072
H,-1.3633721933,-4.323148393,0.1507123963
H,-1.1892869824,3.5730773512,-1.4466543681
H,-1.9588660544,4.3242629898,-0.0219116967
H,-1.8633009682,3.8164032172,2.2683862687
H,-2.6863119916,2.9556599024,3.5781117047
H,-1.2344030456,2.2629777353,2.8162158919
H,-4.1245076128,0.1727012995,1.5120658996
H,-3.9555949379,0.8252849763,3.1521219233
H,-2.5590108925,0.0811807647,2.34209038
H,-4.8806545946,3.0318875475,2.3598502739
H,-4.1566076476,3.9037069979,1.0001627238
H,-5.0417012863,2.3974189515,0.7134888852
H,-3.6273361491,-3.8231714701,0.9913735722
H,-0.2434978788,-2.8149524207,-1.4500890907
O,-5.0677877795,-1.6255728227,0.2332554891
O,-1.3949974074,-0.5801603148,-2.4734136186
C,-5.7054604383,-2.3877641929,1.2452173906
H,-5.112963168,-2.4036203977,2.1700195875
H,-6.6588648307,-1.8909559888,1.4350186497
H,-5.8937364277,-3.4192986277,0.9181968938
C,-0.0868904403,-0.8142790628,-2.9826184928
H,0.1204027959,0.0253750216,-3.6484104003
H,0.658403955,-0.8498707836,-2.1841409758
H,-0.0471236494,-1.7530331774,-3.553636697

PC*Re*
Opt @ B3LYP/6-31G(d)/SDD in gas phase
SCF Done: E(RB3LYP) = -2642.63057240 A.U.
Zero-point correction= 0.925032 (Hartree/Particle)
Sum of electronic and thermal Free Energies= -2641.792519 A.U.

C,-4.2849204965,-1.2952528066,1.5581731745
H,-3.9435240054,-1.5520501716,2.5640462547
H,-4.8454553277,-0.35805966,1.6085704828
H,-4.9662471281,-2.0892666305,1.2263185724
C,-3.0534806027,-1.1661883309,0.6136526414
C,-2.1518452608,-0.0227501722,1.1711892811
C,-1.2308875881,-0.2467891632,2.2022675468
H,-0.8653608878,0.5924345973,2.7935701281
H,-1.1295153606,-1.224629027,2.6677455421
B,-2.6774957971,1.4288403729,0.9591880971
O,-4.0056906154,1.7030512563,0.7169882464
O,-1.9190136172,2.5824659486,1.0497960938
Pd,-0.0364708122,-0.1789007055,0.3920821635

B,-2.3370720626,-2.5877166263,0.663618331
O,-1.9283610091,-3.321857274,-0.4251739408
O,-2.1849964764,-3.2771014459,1.8479071741
C,-2.8499681655,3.7003374656,1.1231344437
C,-4.126784577,3.1143942499,0.4200126704
C,-1.7274366175,-4.6890202442,0.0274869023
C,-1.436350391,-4.4885215904,1.5574012462
C,-5.456438129,3.6163880277,0.9755977654
H,-6.2816234024,3.1439447026,0.4334088102
H,-5.5428498597,4.7020433791,0.8526248316
H,-5.5653577903,3.3726611497,2.0346575525
C,-4.1061222185,3.2468311981,-1.1085671855
H,-4.2736298154,4.2809312715,-1.428184058
H,-4.8922846312,2.6118790771,-1.5263240028
H,-3.1561354054,2.8982524311,-1.5221770887
C,-3.0606478118,3.9913476113,2.6142290633
H,-3.7069119786,4.861233914,2.7711853856
H,-2.0880754517,4.1906020558,3.0745644129
H,-3.5055855554,3.1284685499,3.1197723385
C,-2.2414839556,4.9144801959,0.430369744
H,-1.3423326976,5.2365353592,0.9652277487
H,-2.954041128,5.7471984891,0.427639104
H,-1.9665682793,4.6954709533,-0.6035239759
C,0.0319591982,-4.171989796,1.8497242451
H,0.131468872,-3.8642542632,2.8949746754
H,0.368242653,-3.3395304833,1.2271431315
H,0.6792585027,-5.0386339705,1.6785725652
C,-1.9410428571,-5.6047293342,2.4657104127
H,-1.7090551004,-5.3640131144,3.5080796457
H,-1.4536304719,-6.5544091694,2.2168303903
H,-3.0225313075,-5.7324244089,2.381781584
C,-0.5922244453,-5.3160540618,-0.7739472158
H,-0.8803922485,-5.3867745907,-1.8275431294
H,-0.3758228003,-6.3266152649,-0.4088172531
H,0.3199483366,-4.7210504505,-0.7132087512
C,-3.0424511606,-5.4311599141,-0.2433544127
H,-2.9647246693,-6.4950894558,0.0035058453
H,-3.2891495472,-5.3344875836,-1.3047256088
H,-3.863131597,-4.9959671167,0.3357469127
C,-3.4511437842,-0.8487982107,-0.8358834612
C,-4.7768957369,-0.6375561646,-1.2364586959
C,-2.4553833162,-0.7501185429,-1.8233529336
C,-5.095395448,-0.3288936331,-2.5621920512
H,-5.5786062137,-0.6901788308,-0.50919879
C,-2.7652293767,-0.4447526939,-3.1476868094
H,-1.4166319986,-0.9267953926,-1.5437862616
C,-4.0925597226,-0.2275668109,-3.5262614957
H,-6.1347584983,-0.166220085,-2.8380796534
H,-1.9688483326,-0.3899332004,-3.8865725271
H,-4.3401284137,0.0095564251,-4.5577665799
C,4.9341540734,0.1221151158,-3.5623672009
C,4.4354358654,1.2235749448,-2.8641812922
C,3.553206523,1.0547107273,-1.7874075908
C,3.182443433,-0.2482230515,-1.4194245807
C,3.6909827698,-1.3418100024,-2.1402928209
C,4.567615371,-1.1770859731,-3.2104006837
C,3.0506473705,2.2472087667,-1.0501580903

C,3.9220328588,3.0054001317,-0.246566017
C,3.4655687411,4.1182707966,0.4692663349
C,2.1197411848,4.4701175936,0.3740677893
C,1.2344676831,3.7496851506,-0.4211745322
C,1.7043490797,2.6431866218,-1.1415607315
P,2.1256456607,-0.8336958592,-0.0386604165
C,2.2315145248,-2.5489131521,-0.8083316831
O,3.3255271405,-2.5960657125,-1.7402931641
C,3.3042882372,-0.9438847873,1.4599064183
C,2.6553887997,-1.8875908368,2.4885856783
C,3.4157596051,0.4635254827,2.072748939
C,4.7014031471,-1.4549044669,1.0707133585
H,5.6139506756,0.2772097649,-4.3960070678
H,4.7297455802,2.2290801954,-3.1471651326
H,4.9413397347,-2.046626552,-3.7409410474
H,1.7530381084,5.3264232921,0.934308194
H,1.2970359723,-2.7418701986,-1.3482390213
H,2.3852510891,-3.3393029875,-0.0707522694
H,2.6130554722,-2.9232128599,2.1345082926
H,3.2514723495,-1.8833037148,3.4107306621
H,1.6366362197,-1.5696316329,2.7355617705
H,3.9490096901,1.1421937629,1.4056934335
H,3.978168714,0.4070470174,3.0149318211
H,2.4293987361,0.8910693525,2.2851789418
H,5.3299578971,-1.5250738324,1.9686743561
H,4.6655664311,-2.4478127235,0.6088595258
H,5.1913121126,-0.7732627048,0.3682340851
H,4.133131745,4.6955554176,1.0966251533
H,0.1942555342,4.0336945431,-0.4624088175
O,5.2110376436,2.5443846702,-0.1931506948
O,0.9224613461,1.896314699,-1.9709244562
C,6.1273225787,3.1983339378,0.6686798674
H,5.7934761697,3.1605699387,1.7145864764
H,7.068182598,2.6534168752,0.5700160751
H,6.2830969195,4.2459026331,0.3771591099
C,-0.4277761869,2.3051692271,-2.1655188443
H,-0.8647006572,1.581188443,-2.8518691011
H,-0.9837571002,2.2888220572,-1.2237653872
H,-0.4747530493,3.310373599,-2.6080469147

Allene
Opt @ B3LYP/6-31G(d)/SDD in gas phase
SCF Done: E(RB3LYP) = -387.038192396 A.U.
Zero-point correction= 0.166653 (Hartree/Particle)
Sum of electronic and thermal Free Energies= -386.905965 A.U.

C,4.9250386586,2.0855302259,-1.1061272125
H,5.9168080362,1.6941633707,-1.3654308983
H,4.6063885158,2.764354597,-1.9006398845
H,5.031304866,2.6652714583,-0.1805872325
C,3.924825996,0.9567826922,-0.9285318945
C,2.8555509358,0.9206523579,-1.6979902487
C,1.7975629622,0.88967073,-2.4632264316
H,0.868903164,1.3848265727,-2.1795113189
H,1.7961088784,0.367977085,-3.4202620647
C,4.1752328981,-0.0806124661,0.1087882751

C,5.3197414543,-0.0287832463,0.9217185119
C,3.2731488565,-1.1438933398,0.3059197774
C,5.5543837331,-1.0027498171,1.8952599566
H,6.0391474537,0.7742683986,0.8013013814
C,3.5075475872,-2.113071,1.275343278
H,2.3820874181,-1.2017434596,-0.3126654338
C,4.6517671944,-2.0490635055,2.0781043404
H,6.448054689,-0.9386790247,2.5107013074
H,2.7949379824,-2.9233462983,1.4067445189
H,4.8342919403,-2.806636911,2.8353793229
----

pinB-Bpin
Opt @ B3LYP/6-31G(d)/SDD in gas phase
SCF Done: E(RB3LYP) = -822.578383351 A.U.
Zero-point correction= 0.366676 (Hartree/Particle)
Sum of electronic and thermal Free Energies= -822.258069 A.U.

B,-2.7801965122,-0.0375659533,1.7497910113
O,-3.983710764,-0.6831837279,1.5874612148
O,-1.7884418029,-0.8731747731,2.2078306535
B,-2.5439746736,1.6147703593,1.4199207827
O,-1.9661091598,2.4942301851,2.3054971448
O,-2.9100778638,2.2165555193,0.2386304925
C,-2.3008194854,-2.2352821186,2.1536549461
C,-3.8554828762,-2.0081479122,2.1780794165
C,-2.1423080704,3.8371006505,1.7702764239
C,-2.3497317571,3.5607352368,0.2374118229
C,-4.6680465005,-2.9945625294,1.3440627233
H,-5.7319704842,-2.7470665705,1.414086818
H,-4.5290111542,-4.0174660347,1.7127633506
H,-4.3828564147,-2.9592272067,0.2905465289
C,-4.4315199243,-1.9105487616,3.5964767562
H,-4.4360317889,-2.8837234268,4.0985915007
H,-5.4610185926,-1.5450217091,3.5351667955
H,-3.8569092144,-1.2046993622,4.2042129782
C,-1.7970310832,-2.8382485022,0.8361481634
H,-2.0821556423,-3.8910430368,0.7384949251
H,-0.7053510277,-2.7688246926,0.8106248876
H,-2.1918649536,-2.2865796213,-0.0226369216
C,-1.7406837976,-3.0145650272,3.3401722978
H,-0.65480503,-3.1084903747,3.2394886861
H,-2.1700507708,-4.0224824394,3.3769018714
H,-1.9486326661,-2.5096177329,4.2858275334
C,-1.0342880556,3.4837500896,-0.5479986768
H,-1.2412495854,3.0836690384,-1.5451634578
H,-0.3230508675,2.8138243868,-0.0549599838
H,-0.5704315719,4.4697458169,-0.6569351359
C,-3.3300196063,4.4970822966,-0.4634142014
H,-3.4164019741,4.217415924,-1.5181257589
H,-2.9774496114,5.5337063763,-0.4113529425
H,-4.3255105985,4.4419554241,-0.0179461806
C,-0.909582139,4.6665182578,2.1185872045
H,-0.8451647657,4.792539064,3.2039723036
H,-0.9718209568,5.660717899,1.6610952685
H,0.0086825466,4.1814604351,1.7809603006
C,-3.3855157392,4.4193651115,2.4548136891

H,-3.5597358848,5.4593315924,2.1592893807
H,-3.2407378684,4.3844801367,3.538814576
H,-4.2765323119,3.8317617128,2.2128268111

Product
Opt @ B3LYP/6-31G(d)/SDD in gas phase
SCF Done: E(RB3LYP) = -1209.69328872 A.U.
Zero-point correction= 0.537995 (Hartree/Particle)
Sum of electronic and thermal Free Energies= -1209.213107 A.U.

C,-4.5517763589,-1.3979786804,1.6893117097
H,-4.2347111366,-1.6696754061,2.6995320891
H,-5.1130453288,-0.4593057332,1.7365024824
H,-5.2233870527,-2.1872490747,1.3298353165
C,-3.3023845535,-1.247515467,0.7809327247
C,-2.4188904616,-0.1340550301,1.3840148814
C,-1.3430417113,-0.3561125286,2.1551706737
H,-0.7718295057,0.4768426975,2.5584261969
H,-1.0054139125,-1.351239496,2.4333852499
B,-2.8477224887,1.3439234732,1.1195515598
O,-4.1503107433,1.7287544604,0.9086297046
O,-1.9644290049,2.396771527,1.08681247
B,-2.5413572098,-2.6433195512,0.7669889105
O,-1.9859825669,-3.2187025153,-0.3475380527
O,-2.3911018333,-3.4171402561,1.8948978516
C,-2.7538206929,3.6176844442,1.0232741016
C,-4.1168860633,3.1014685447,0.4310992015
C,-1.6357051322,-4.5873622176,-0.0055072866
C,-1.4938921919,-4.5128261161,1.559048858
C,-5.3600432589,3.8242650724,0.9391158577
H,-6.2520047306,3.3825774894,0.4836177436
H,-5.3273818448,4.8858860831,0.668582395
H,-5.458435044,3.7411159532,2.0238070114
C,-4.1245294148,3.0296888275,-1.1008491751
H,-4.1635239266,4.0274617342,-1.551024298
H,-4.999324193,2.4593791075,-1.4242989511
H,-3.2410253422,2.5041129835,-1.4737083783
C,-2.8774819841,4.1368796386,2.4608326757
H,-3.4040576625,5.0962426023,2.500458704
H,-1.8739991072,4.2727953305,2.8757069643
H,-3.4112232137,3.4179828015,3.0904436426
C,-2.013379717,4.627450228,0.1522191983
H,-1.0737326331,4.9134881428,0.6356900806
H,-2.6162202985,5.5320500487,0.0122396382
H,-1.7757227903,4.2083925371,-0.8277830103
C,-0.0908650122,-4.0947851539,2.0156558729
H,-0.1213378442,-3.8578428175,3.0836191876
H,0.2461353793,-3.20337392,1.4780472686
H,0.6388630055,-4.8954938023,1.8573632533
C,-1.9465561982,-5.7601090582,2.3118168217
H,-1.8218596033,-5.6064321722,3.3883949947
H,-1.3454420396,-6.6279833941,2.0173524821
H,-2.9989933116,-5.9815331129,2.1228984451

8. Asymmetric synthesis of (2*R*, 3*R*)- and (2*R*, 3*S*)-brassinazole

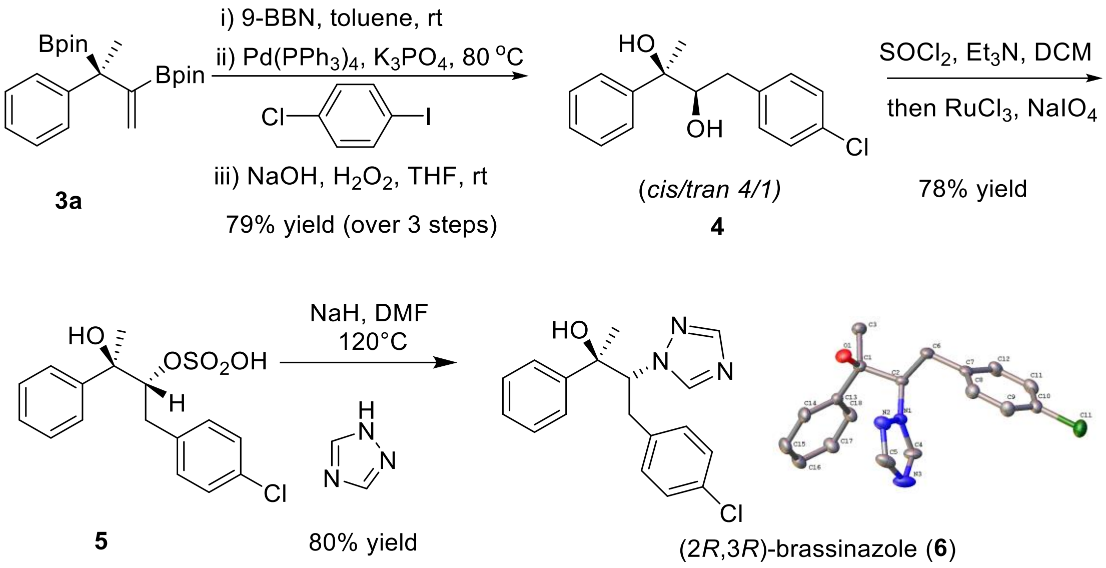

(2*R*, 3*R*)-1-(4-Chlorophenyl)-3-phenylbutane-2,3-diol (4): To a solution of **3a** (3.84 g, 10 mmol, 1.0 equiv) in toluene (20 mL) was added 9-BBN dimer (3.63 g, 15 mmol, 1.5 equiv) at rt and the mixture was stirred at rt for 10 h. To the mixture was added water (1.8 mL, 100 mmol, 10.0 equiv), $Pd(PPh_3)_4$ (577.8 mg, 0.5 mmol, 5 mol %), 1-chloro-4-iodobenzene (4.76 g, 20 mmol, 2.0 equiv), and K_3PO_4 (6.39 g, 30 mmol, 3.0 equiv). The resulting mixture was stirred at 80 °C under nitrogen for 12 h and then cooled to room temperature for next oxidation step. To the mixture at 0 °C was charged THF (50 mL), NaOH (3 M, 10 mL), and H_2O_2 (30% w/w, 10 mL). The resulting mixture was kept at 0 °C for 1 h, then allowed to warm to room temperature over 1 h, and kept stiring at rt for additional 8 h. The mixture was quenched with saturated aqueous sodium thiosulfate (10 mL) and transferred to a separatory funnel for extraction. The aqueous phase was extracted with EtOAc (20 mL × 3). The combined organic phase was washed with brine, dried over magnesium sulfate, and evaporated to dryness. The diasteromeric ratio **4/7** (4/1) was determined by collecting the ^{1}H NMR data of the crude product. The residue was purified by column chromatography (eluent: PE/EA 3/1) to afford compound (**4/7**, 2.19 g, 79%, 4/1) as white solid. **4**: ^{1}H NMR (500 MHz, acetone-d6) δ 7.66-7.59 (m, 2H), 7.37-7.30 (m, 2H), 7.28-7.20 (m, 3H), 7.18-7.12 (m, 2H), 4.16 (s,

1H), 3.91-3.83 (m, 2H), 2.87-2.82 (m, 1H), 2.29 (dd, *J* = 13.9, 10.1 Hz, 1H), 1.63 (s, 3H) ppm; ^{13}C NMR (126 MHz, acetone-d^6) δ 147.1, 140.6, 131.81, 131.75, 128.7, 128.4, 127.3, 127.1, 80.7, 76.7, 38.0, 25.4 ppm. EI-MS m/z: 276; HRMS (EI) m/z: calcd. for $C_{16}H_{17}ClO_2^+$: 276.0917, Found: 276.0923.

(2*R*, 3*R*)-1-(4-Chlorophenyl)-3-hydroxy-3-phenylbutan-2-yl hydrogen sulfate (5): To a mixture of **4/7** (138.4 mg, 0.5 mmol, 1.0 equiv), Et_3N (0.17 mL, 1.2 mmol, 2.4 equiv) in CH_2Cl_2 (2.0 mL) at 0 ^{o}C was added dropwise $SOCl_2$ (44 μL, 0.6 mmol, 1.2 equiv) over 5 min. The mixture was further stirred at 0 ^{o}C for 30 min and then concentrated to dryness. To the residue was added $NaIO_4$ (160.4 mg, 0.75 mmol, 1.5 equiv), $RuCl_3 \cdot 3H_2O$ (7mg, 5.0 mol %), and CH_3CN/H_2O (1mL/1mL). After stirred at rt for 30 min, the solution was quenched with saturated aqueous sodium thiosulfate (2 mL). Dichloromethane (10 mL) was added and the organic phase was separated, washed with brine, dried over sodium sulfate, concentrated, and purified by column chromatography (eluent: EA/MeOH 10/1) to afford compound **5** (138.5 mg, 78% yield, dr >20:1) as yellow solid. **5**: ^{1}H NMR (500 MHz, acetone-d6) δ 7.62-7.57 (m, 2H), 7.33-7.26 (m, 2H), 7.26-7.22 (m, 1H), 7.11-7.07 (m, 2H), 7.06-7.01 (m, 2H), 5.13 (s, 1H), 4.84 (dd, *J* = 6.2, 5.1 Hz, 1H), 3.50 (s, 1H), 2.70 (dd, *J* = 14.7, 6.2 Hz, 1H), 2.64 (dd, *J* = 14.7, 5.0 Hz, 1H), 1.66 (s, 3H) ppm; ^{13}C NMR (126 MHz, acetone-d6) δ 145.6, 138.8, 132.0, 131.6, 128.6, 128.4, 127.8, 127.7, 87.4, 77.2, 37.9, 23.3 ppm; ESI-MS m/z: 355.1 $[M-H]^-$; HRMS (ESI) m/z: calcd. for $C_{16}H_{16}ClNa_2O_5S^+$: 401.0197, Found: 401.0202 $[M+2Na-H]^+$.

(2*R*,3*R*)-4-(4-Chlorophenyl)-3-methyl-2-phenyl-3-(1H-1,2,4-triazol-1-yl)butan-2-ol (6): To a solution of NaH (320 mg, 8.0 mmol, 5.0 equiv, 60% content) in DMF (10 mL) at 0 ^{o}C was added 1,2,4-triazole (1.1g, 16.0 mmol, 10.0 equiv) under nitrogen. The mixture was allowed to warm to rt and stirred for 0.5 h. The mixture at 0 ^{o}C was added dropwise to a solution of **5** (540 mg, 1.6 mmol, 1.0 equiv) in DMF (10 mL). The resulting mixture was stirred at 120 ^{o}C for 12 h, then cooled to room temperature, and concentrated. To the residue was added EtOAc (50 mL) and water (20 mL). The organic

layer was washed with brine, dried over sodium sulfate, concentrated, and purified by column chromatography (eluent: PE/EA 3/1) to afford compound **6** (420 mg, 80 % yield) as yellow soild. **6**: M.p. 133-135 ºC. $[\alpha]_D^{20}$ = 71.4 (*c* 0.75, $CHCl_3$), 92% *ee* [determined by HPLC analysis using a Chiralcel OD-3 column; *n*-Hex/*i*-PrOH = 85:15, 1.0 mL/min, λ = 210 nm; t_R (major) = 11.07 min; t_R (minor) = 16.51 min]. ^{1}H NMR (500 MHz, $CDCl_3$) δ 7.80 (s, 1H), 7.71-7.67 (m, 1H), 7.49-7.45 (m, 2H), 7.26-7.21 (m, 2H), 7.18-7.12 (m, 3H), 7.01-6.96 (m, 2H), 5.02 (dd, *J* = 11.6, 3.4 Hz, 1H), 4.93 (s, 1H), 3.45 (dd, *J* = 14.2, 11.6 Hz, 1H), 3.35 (dd, *J* = 14.2, 3.3 Hz, 1H), 1.74 (s, 3H) ppm; ^{13}C NMR (126 MHz, $CDCl_3$) δ 151.7, 147.1, 145.7, 137.9, 132.5, 131.4, 129.0 ,128.7, 127.6, 125.9, 76.8, 70.6, 35.1, 26.7 ppm. IR (neat) ν 3272, 2981, 1497, 1277, 1133, 1087, 1012, 812, 761, 695, 666 cm^{-1}; ESI-MS m/z: 328.3 $[M+H]^+$; HRMS (ESI) m/z: calcd. for $C_{18}H_{19}ClN_3O^+$: 328.1211, Found: 328.1212 $[M+H]^+$.

Crystal structural data of product 6

A single crystal of product **6** was obtained by recrystallization from CH_2Cl_2/*n*-hexane. Its X-ray diffractional data and the refinement were shown in Table S7. The absolute configuration of **6** was determined to be (2*R*, 3*R*) (Figure *S4*).

Table S7. Crystal data and structure refinement for 6.

Identification code	mo_dm16701_0m	
Empirical formula	C18 H18 Cl N3 O	
Formula weight	327.80	
Temperature	130 K	
Wavelength	0.71073 Å	
Crystal system	Monoclinic	
Space group	P 1 21 1	
Unit cell dimensions	a = 11.6963(15) Å	α= 90°.
	b = 7.8375(10) Å	β= 96.443(2)°.
	c = 18.213(2) Å	γ = 90°.
Volume	1659.0(4) Å^3	
Z	4	
Density (calculated)	1.312 Mg/m^3	
Absorption coefficient	0.238 mm^{-1}	
F(000)	688	
Crystal size	0.15 x 0.1 x 0.05 mm^3	

Theta range for data collection	1.125 to 30.618°.
Index ranges	-16<=h<=16, -11<=k<=11, -26<=l<=25
Reflections collected	16974
Independent reflections	9757 [R(int) = 0.0321]
Completeness to theta = 26.000°	100.0 %
Absorption correction	Semi-empirical from equivalents
Max. and min. transmission	0.7461 and 0.6819
Refinement method	Full-matrix least-squares on F^2
Data / restraints / parameters	9757 / 1 / 419
Goodness-of-fit on F^2	1.014
Final R indices [I>2sigma(I)]	R1 = 0.0464, wR2 = 0.0871
R indices (all data)	R1 = 0.0693, wR2 = 0.0972
Absolute structure parameter	0.00(3)
Extinction coefficient	n/a
Largest diff. peak and hole	0.240 and -0.302 e.Å^{-3}

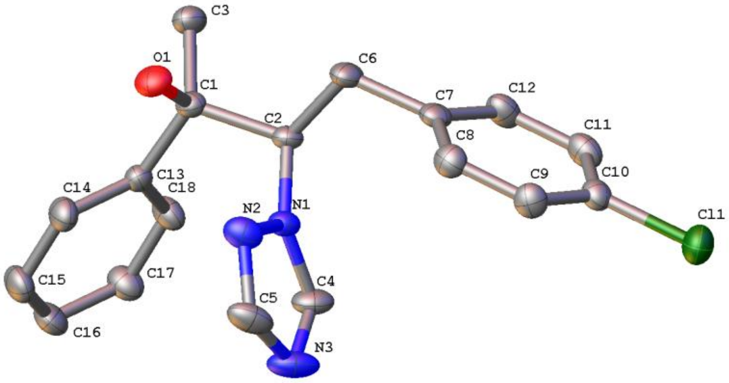

Figure S4. X-ray crystal structure of (2*R*, 3*R*)-**6**

The CIF file of **6** can be obtained from the Cambridge Crystallographic Data Centre using deposition numbers 1517578. Copies of the data can be obtained, free of charge, on application to the CCDC, 12 Union Road, Cambridge CB2 1EZ, UK [fax: +44 (1223) 336 033; e-mail: deposit@ccdc.cam.ac.uk].

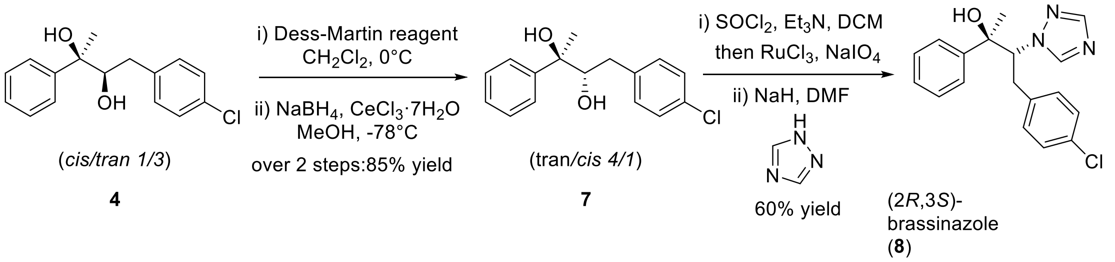

(2*R*, 3*S*)-1-(4-Chlorophenyl)-3-phenylbutane-2,3-diol（7）: To a solution of **4** (276.8 mg, 1.0 mmol, 1.0 equiv) in DCM (5.0 mL) at 0 ^{o}C was added Dess-Martin periodinane (636.2 mg, 1.5 mmol, 1.5 equiv) under nitrogen. The mixture was allowed to warm to rt and stirred for 3 h. The mixture was then quenched with saturated $NaHCO_3$ solution (5 mL) and extracted with dichloromethane (20 mL). The organic layer was separated, washed sequentially with sodium thiosulfate (20 mL) and brine (20 mL), dried over sodium sulfate, concentrated under vacuum, and purified by column chromatography (eluent: PE/EA 3/1) to afford crude (*R*)-1-(4-chlorophenyl)-3-hydroxy-3-phenylbutan-2-one.

To a solution of (*R*)-1-(4-chlorophenyl)-3-hydroxy-3-phenylbutan-2-one (55 mg, 0.2 mmol, 1.0 equiv) and $CeCl_3 \cdot 7H_2O$ (89.4 mg, 0.24 mmol, 1.2 equiv) in MeOH (2 mL) at -78 ^{o}C was added $NaBH_4$ (15 mg, 0.4 mmol, 2.0 equiv) under nitrogen. The mixture was stirred at -78 ^{o}C for 5 h, then allowed to warm to 0 ^{o}C, and quenched with saturated NH_4Cl solution (5 mL). EtOAc (10 mL) was added and the organic phase was separated, washed with brine, dried over sodium sulfate, concentrated, and purified by column chromatography (eluent: PE/EA 3/1) to afford compound **7/4** (4/1, 56 mg, 85% yield for over two steps) as white solid. **7**: ^{1}H NMR (500 MHz, acetone-d^{6}) δ 7.60-7.54 (m, 2H), 7.38-7.32 (m, 2H), 7.26-7.18 (m, 3H), 7.13-7.08 (m, 2H), 4.01 (s, 1H), 3.88 (q, J = 6.4 Hz, 1H), 3.81 (d, J = 6.9 Hz, 1H), 2.51 (d, J = 6.2 Hz, 2H), 1.60 (s, 3H) ppm; ^{13}C NMR (126 MHz, acetone-d^{6}) δ 147.8 140.7, 131.8, 131.7, 128.71, 128.68, 127.1, 126.3, 79.9, 76.9, 38.0, 27.5 ppm. EI-MS m/z: 276; HRMS (EI) m/z: calcd. for $C_{16}H_{17}ClO_2^+$: 276.0917, Found: 276.0923.

(2*R*,3*S*)-4-(4-Chlorophenyl)-3-methyl-2-phenyl-3-(1H-1,2,4-triazol-1-yl)butan-2-ol (8) To a mixture of **7** (138.4 mg, 0.5 mmol, 1.0 equiv), Et_3N (0.17 mL, 1.2 mmol, 2.4 equiv) in CH_2Cl_2 (2.0 mL) at 0 ^{o}C was added dropwise $SOCl_2$ (44 μL, 0.6 mmol, 1.2 equiv) over 5 min. The mixture was further stirred at 0 ^{o}C for 30 min and then concentrated to dryness. To the residue was added $NaIO_4$ (160.4 mg, 0.75 mmol, 1.5 equiv), $RuCl_3 \cdot 3H_2O$ (7mg, 5.0 mol%), and CH_3CN/H_2O (1mL/1mL). After stirred at rt for 30 min, the solution was quenched with saturated aqueous sodium thiosulfate (2

mL). Dichloromethane (10 mL) was added and the organic phase was separated, washed with brine, dried over sodium sulfate, concentrated, and purified by column chromatography (eluent: EA/MeOH 10/1) to afford compound (2*R*,3*S*)- 1-(4-Chlorophenyl)-3-hydroxy-3-phenylbutan-2-yl hydrogen sulfate.

To a solution of NaH (9.0 mg, 0.23 mmol, 5.0 equiv, 60% content) in DMF(1.0 mL) at 0 ^{o}C was added 1,2,4-triazole (31 mg, 0.45 mmol, 10.0 equiv) under nitrogen. The mixture was allowed to warm to rt and stirred for 0.5 h. Then the mixture at 0 ^{o}C was added dropwise to a solution of (2*R*,3*S*)- 1-(4-Chlorophenyl)-3-hydroxy-3-phenylbutan-2-yl hydrogen sulfate (16 mg, 0.045 mmol, 1.0 equiv) in DMF (1 mL). The resulting mixture was stirred at 120 ^{o}C for 12 h, then cooled to room temperature, and concentrated. To the residue was added EtOAc (5 mL) and water (2 mL). The organic layer was washed with brine, dried over sodium sulfate, concentrated, and purified by column chromatography (eluent: PE/EA 3/1) to afford compound **8** (8.8 mg, 60% yield) as yellow soild. **8**: M.p. 175-178 ^{o}C. $[\alpha]_D^{20}$ = -8.7 (*c* 0.175, $CHCl_3$). 91% *ee* [determined by HPLC analysis using a Chiralcel ID-3 column; *n*-Hex/*i*-PrOH = 90:10, 1.0 mL/min, λ = 210 nm; t_R (minor) = 5.70 min; t_R (major) = 6.78 min]. ^{1}H NMR (500 MHz, $CDCl_3$) δ 7.74 (s, 1H), 7.27-7.23 (m, 2H), 7.22-7.17 (m, 2H), 7.17 (s, 1H), 7.15-7.12 (m, 1H), 7.12-7.09 (m, 2H), 6.80-6.73 (m, 2H), 4.83 (s, 1H), 4.48 (dd, *J* = 11.7, 3.0 Hz, 1H), 3.46 (dd, *J* = 14.2, 11.6 Hz, 1H), 3.28 (dd, *J* = 14.2, 3.0 Hz, 1H), 1.72 (s, 3H). ppm; ^{13}C NMR (126 MHz, $CDCl_3$) δ 151.4, 145.1, 144.1, 135.7, 132.6, 129.8, 128.6, 128.2, 127.1, 124.0, 76.3, 70.7, 34.4, 26.4 ppm. IR (neat) ν 3332, 1509, 1491, 1447, 1277, 1141, 1095, 763, 698, 679 cm^{-1}; ESI-MS m/z: 328.1 $[M+H]^+$; HRMS (ESI) m/z: calcd. for $C_{18}H_{19}ClN_3O^+$: 328.1211, Found: 328.1210 $[M+H]^+$.

9. Nonlinear effect study

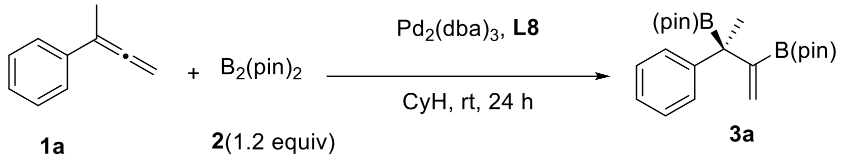

To a flame-dried Schlenk tube was added $Pd_2(dba)_3$ (1 mol %), **L8** (2.5 mol%, mixture of **L8** and *ent*-**L8**), and cyclohexane (2 mL). The resulting mixture was stirred at rt under nitrogen for 0.5 h. Substrate **1a** (0.2 mmol, 1.0 equiv) and B_2pin_2 (0.24 mmol, 1.2 equiv) was added in one portion and the resulting mixture was stirred at rt for 24 h, then quenched with saturated NH_4Cl solution (2 mL), and extracted with EtOAc (2 mL). The organic layer was separated, washed with brine, dried over sodium sulfate, and concentrated. The crude product was purified by flash chromatography on silica gel and the enantiomeric excess was determined by HPLC using a chiralcel IC-3 column.

Entry	1	2	3	4	5	6
ee of **L8**	0	32%	46%	61%	76%	>99%
ee of **3a**	0	29%	49%	63%	78%	94%

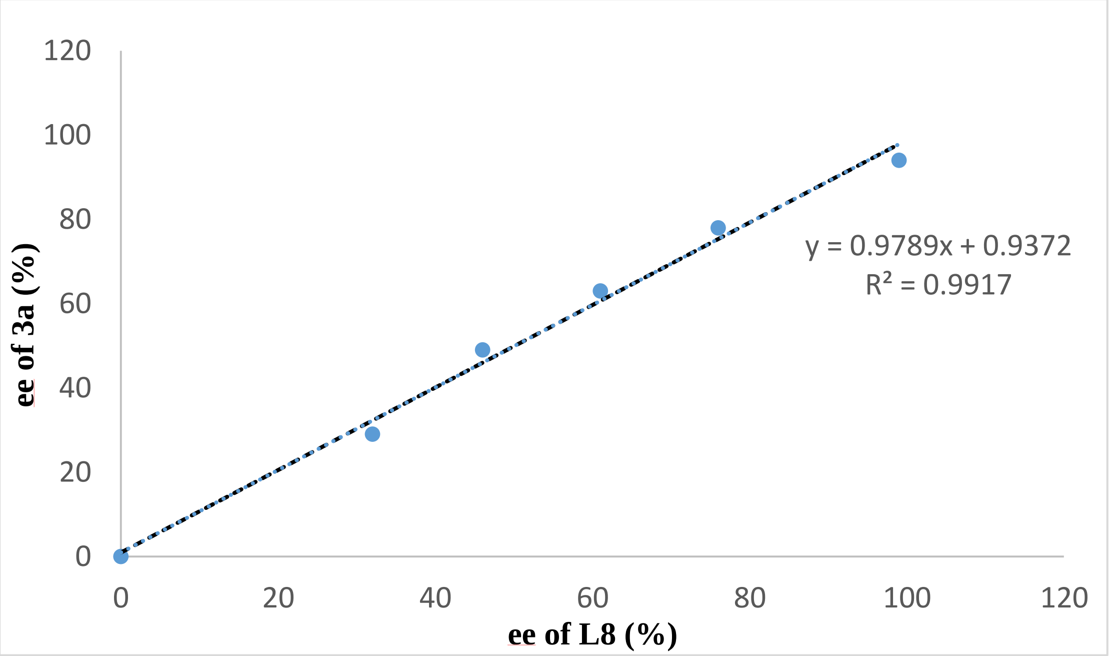

10. References

[1] a) X. Wang, Z. Han, Z. Wang, K. L. Ding, *Angew. Chem., Int. Ed.* **2012**, *51*, 936; b) X. Wang, Z. Wang, K. L. Ding, *Adv. Synth. Catal.* **2013**, *355*, 2900.

[2] a) W. Tang, A. G. Capacci, X. D. Wei, W. J. Li, A. White, N. D. Patel, J. Savoie, J. J. Gao, S. Rodriguez, B. Qu, N. Haddad, B. Z. Lu, D. Krishnamurthy, N. K. Yee, C. H. Senanayake, *Angew. Chem. Int. Ed*. **2010**, 49, 5879-5883; b) W. Tang, N. D. Patel, G. Q. Xu, X. B. Xu, J. Savoie, S. L. Ma, M. H. Hao, S. Keshipeddy, A. G. Capacci, X. D. Wei, Y. D. Zhang, J. J. Gao, W. J. Li, S. Rodriguez, B. Z. Lu, N. K. Yee, C. H. Senanayake, *Org. Lett*. **2012**, 14, 2258-2261.

[3] a) W. Tang, S. Keshipeddy, Y. D. Zhang, X. D. Wei, J. Savoie, N. D. Patel, N. K. Yee, C. H. Senanayake, *Org. Lett*. **2011**, 13, 1366-1369; b) Q. Zhao, C. X. Li, C. H. Senanayake, W. Tang, *Chem. Eur. J*. **2013**, 19, 2261-2265.

[4] G. Xu, W. Fu, G. Liu, C. H. Senanayake, W. Tang, *J. Am. Chem. Soc*. **2013**, 136, 570-573.

[5] a) S. Oda, B. Sam, M. J. Krische, *Angew. Chem. Int. Ed.* **2015**, 54, 8525; b) Y. Tani, T. Fujihara, J. Terao, Y. Tsuji, *J. Am. Chem. Soc*. **2014**, 136, 17706; c) M. S. Baird, A. V. Nizovtsev, I. G. Bolesov, *Tetrahedron*. **2002**, 58, 1581; d) S. Yamazaki, Y. Yamamoto, Y. Fukushima, M. Takebayashi, T. Ukai, Y. Mikata, *J. Org. Chem*, **2010**, 75, 5216; e) X. D. Yang, Y. She, Y. Chong, H. C. Zhai, H. Zhu, B. H. Chen, G. S. Huang, R. L. Yan; *Adv. Synth. Catal.* **2016**, *358*, 3130; f) M. L. Conner; M. K. Brown, *Tetrahedron*. **2016**, 72, 3759; g) H. Jang, B. Jung, A. H. Hoveyda, *Org. Lett.* **2014**, 16, 4658; h) T. Sawano, K. Ou, T. Nishimura, T. Hayashi, *J. Org. Chem*, **2013**, 78, 8986; i) Q. Li, J. Liao, Y. Huang, R. Chiang, H. Gau, *Org. Biomol. Chem*, **2014**, 12, 7634; j) S. Ma, A. Zhang, *J. Org. Chem*, **2002**, *67*, 2287; k) S. Ma, A. Zhang, *J. Org. Chem*, **1998**, *63*, 9601; l) A. Köpfer, B. Breit, *Angew. Chem. Int. Ed*. **2015**, *54*, 6913-6917; m) S. A. Fleming; R. L. Liu; J. T. Redd, *Tetrahedron Lett*. **2005**, *46*, 8095; n) M. C. P. Pinazzi; J. P. Villette; A. Pleurdeau, *Eur. Polym. J*, **1997**, *9*, 1211.

[6] See Supporting Information for computational details.

[7] (a) C. Y. Legault, Y. Garcia, C. A. Merlic, K. N. Houk, *J. Am. Chem. Soc.* **2007**, *129*,

12664. (b) A. E. Hayden, K. N. Houk, *J. Am. Chem. Soc.* **2009**, *131*, 4084.

[8] H. E. Burks, S. Liu, S, J. P. Morken, *J. Am. Chem. Soc.* **2007**, *129*, 8766.

[9] (a) A. D. Becke, *J. Chem. Phys.* **1993**, *98*, 1372. (b) C. Lee, W. Yang, R. G. Parr, *Phys. Rev. B* **1988**, *37*, 785. (c) A. D. Becke, *Phys. Rev. A* **1988**, *38*, 3098. (d) P. J. Stephens, F. J. Devlin, C. F. Chabalowski, M. J. Frisch, *J. Phys. Chem* **1994**, *98*, 11623.

[10] S. Grimme, J. Antony, S. Ehrlich, H. Krieg, *J. Chem. Phys.* **2010**, *132*, 154104.

[11] A. Bergner, M. Dolg, W. Küchle, H. Stoll, H. Preuß, *Mol. Phys.* **1993**, *80*, 1431.

[12] M. J. Frisch, G. W. T., H. B. Schlegel, G. E. Scuseria, M. A. Robb, J. R. Cheeseman, G. Scalmani, V. Barone, B. Mennucci, G. A. Petersson, H. Nakatsuji, M. Caricato, X. Li, H. P. Hratchian, A. F. Izmaylov, J. Bloino, G. Zheng, J. L. Sonnenberg, M. Hada, M. Ehara, K. Toyota, R. Fukuda, J. Hasegawa, M. Ishida, T. Nakajima, Y. Honda, O. Kitao, H. Nakai, T. Vreven, J. A. Montgomery, Jr., J. E. Peralta, F. Ogliaro, M. Bearpark, J. J. Heyd, E. Brothers, K. N. Kudin, V. N. Staroverov, R. Kobayashi, J. Normand, K. Raghavachari, A. Rendell, J. C. Burant, S. S. Iyengar, J. Tomasi, M. Cossi, N. Rega, J. M. Millam, M. Klene, J. E. Knox, J. B. Cross, V. Bakken, C. Adamo, J. Jaramillo, R. Gomperts, R. E. Stratmann, O. Yazyev, A. J. Austin, R. Cammi, C. Pomelli, J. W. Ochterski, R. L. Martin, K. Morokuma, V. G. Zakrzewski, G. A. Voth, P. Salvador, J. J. Dannenberg, S. Dapprich, A. D. Daniels, Ö. Farkas, J. B. Foresman, J. V. Ortiz, J. Cioslowski, and D. J. Fox. *Gaussian 09*, revision D.01; Gaussian, Inc. Wallingford, CT: **2009**.

[13] CYLview, 1.0b; Legault, C. Y., Université de Sherbrooke, **2009** (http://www.cylview.org).

[14] The PyMOL Molecular Graphics System, Version 1.8 Schrödinger, LLC.

[15] S. Grimme, J. Antony, S. Ehrlich, H. Krieg, *J. Chem. Phys.* **2010**, *132*, 154104.

11. NMR and HPLC spectra

NMR spectra of 1a

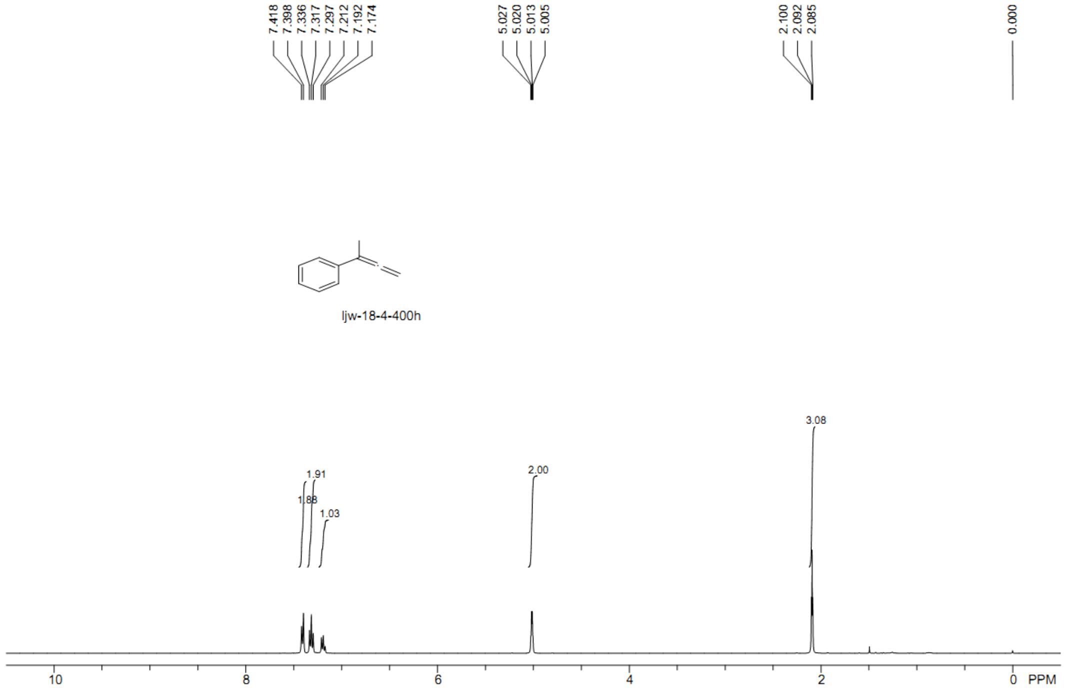

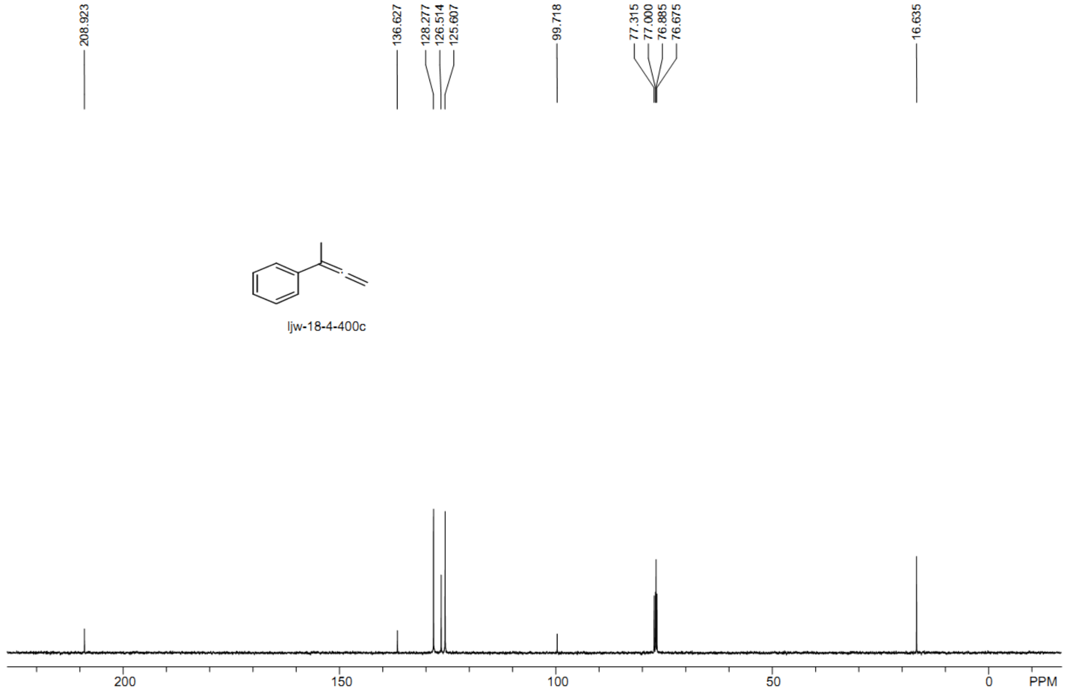

NMR spectra of 1b

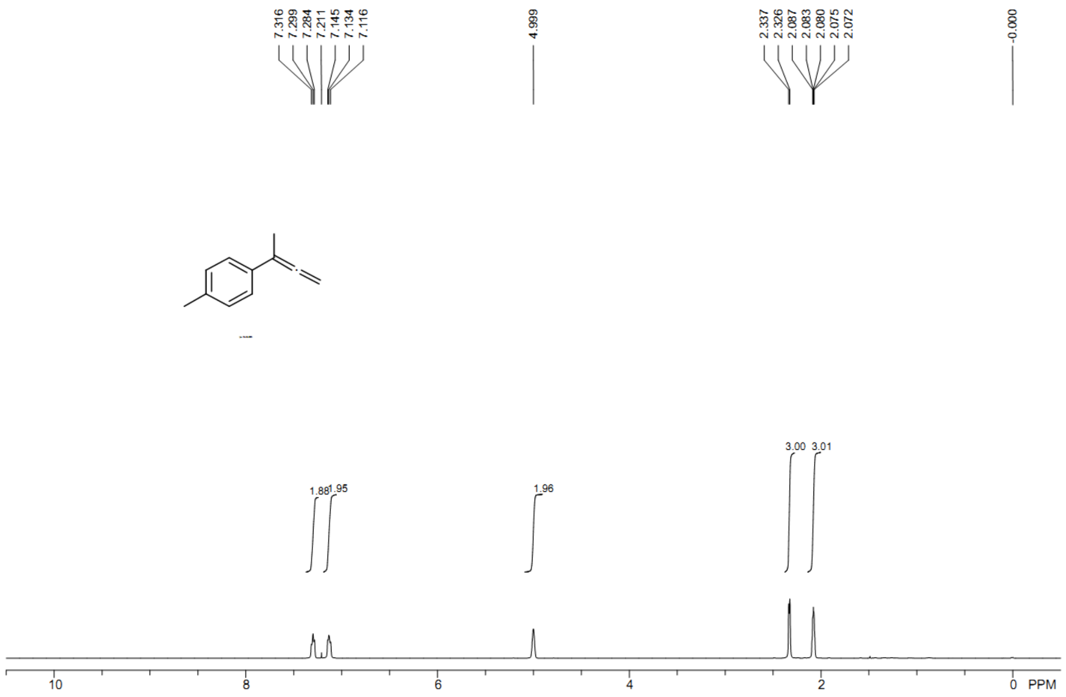

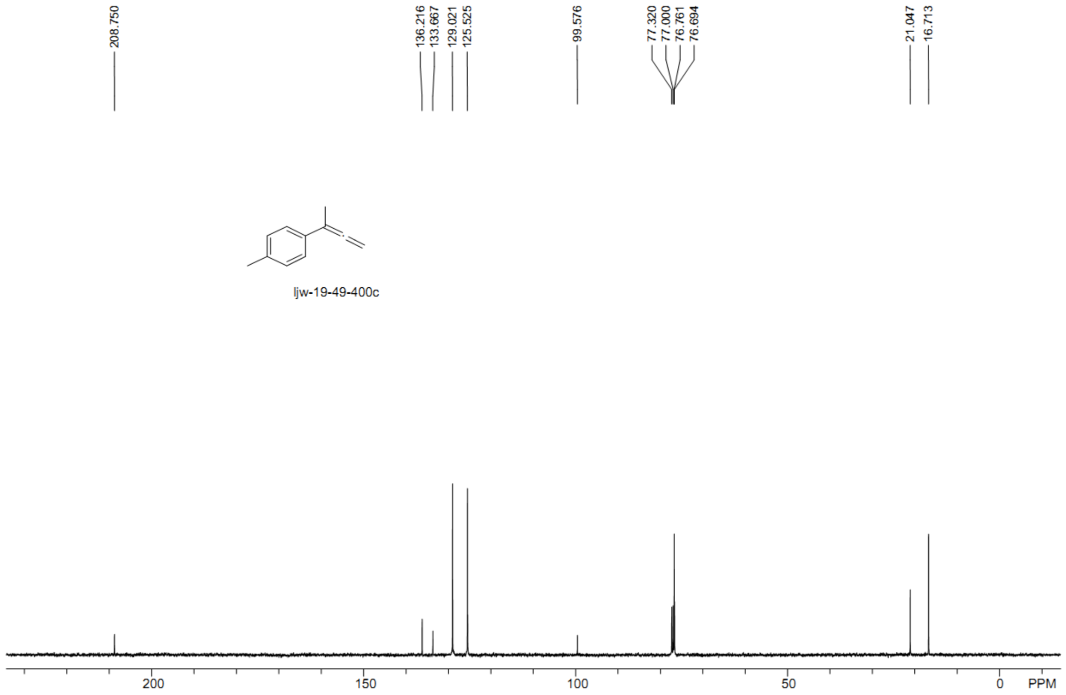

NMR spectra of 1c

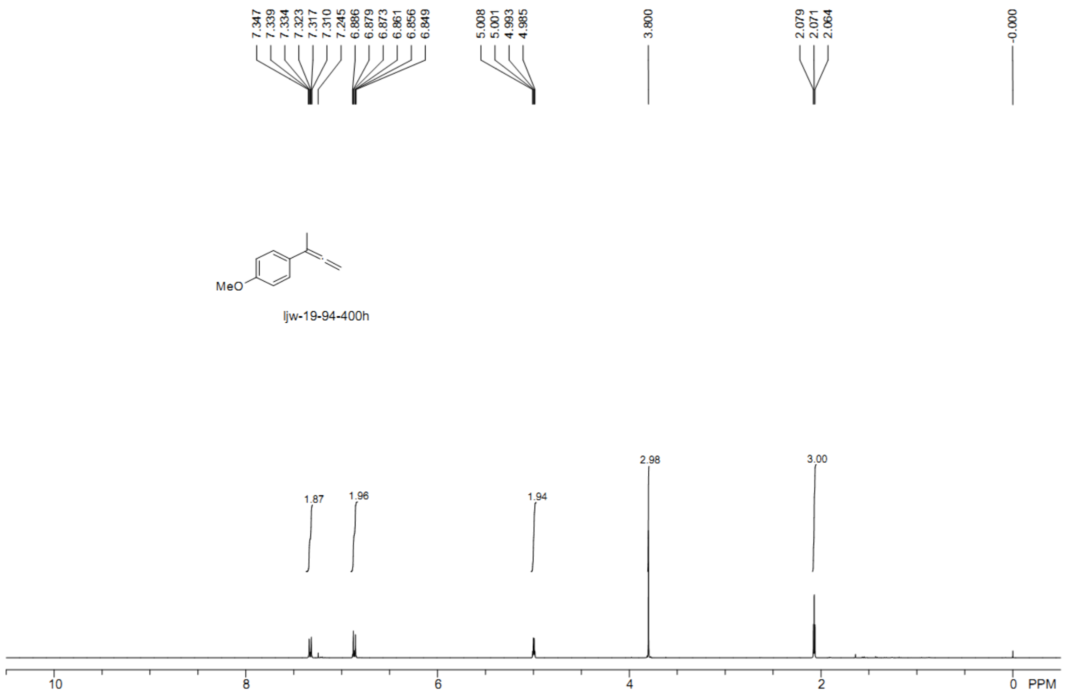

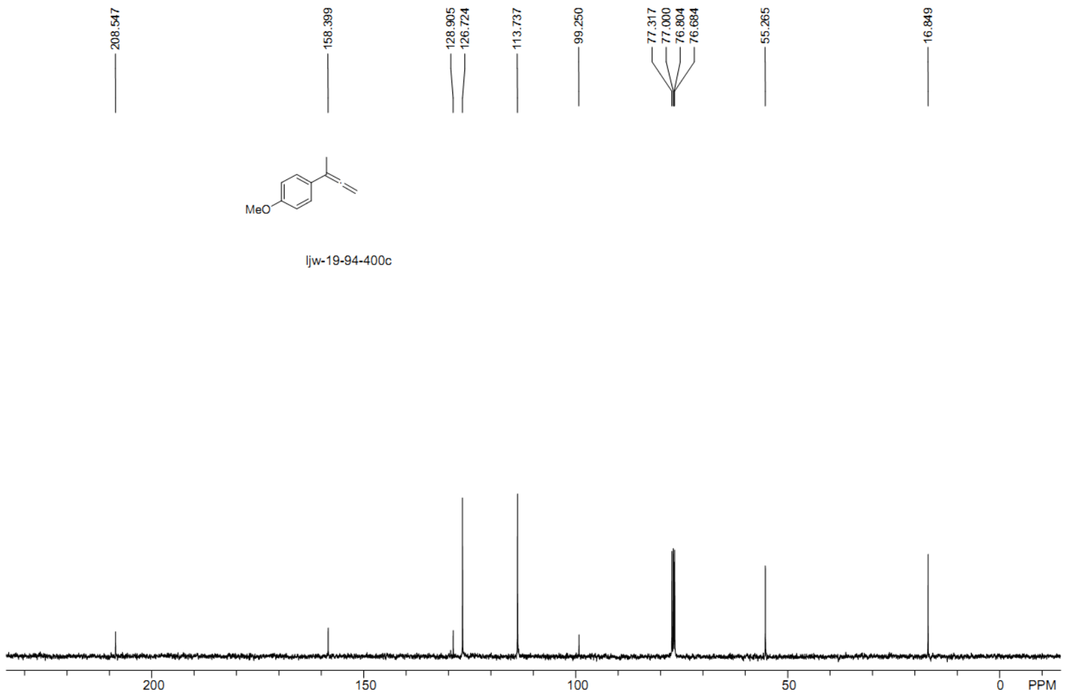

NMR spectra of 1d

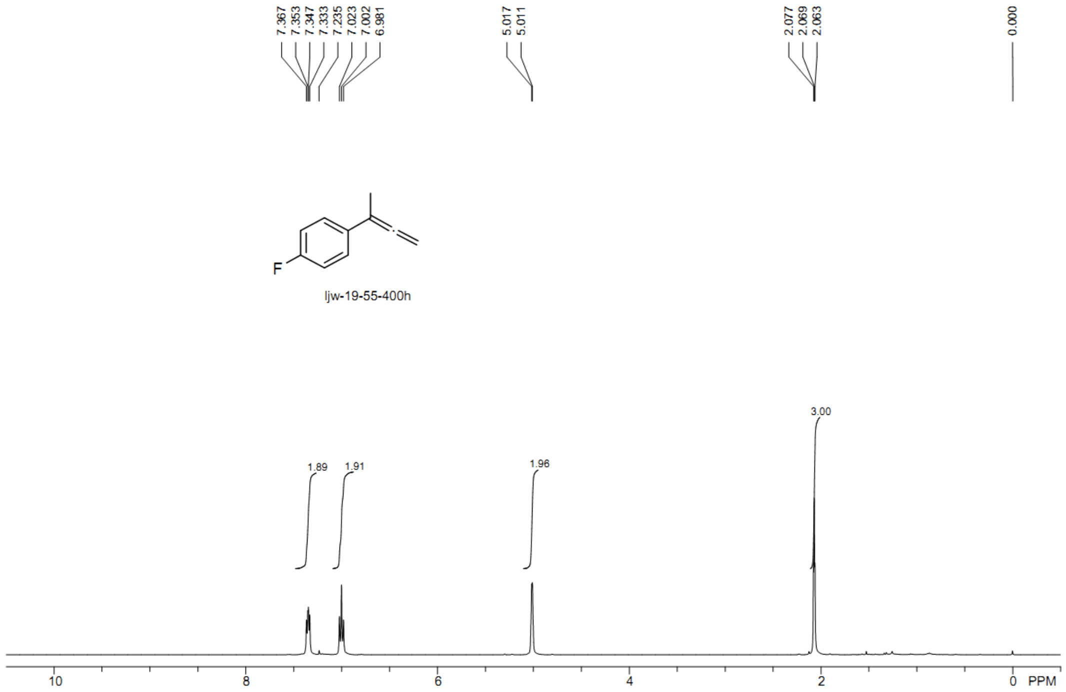

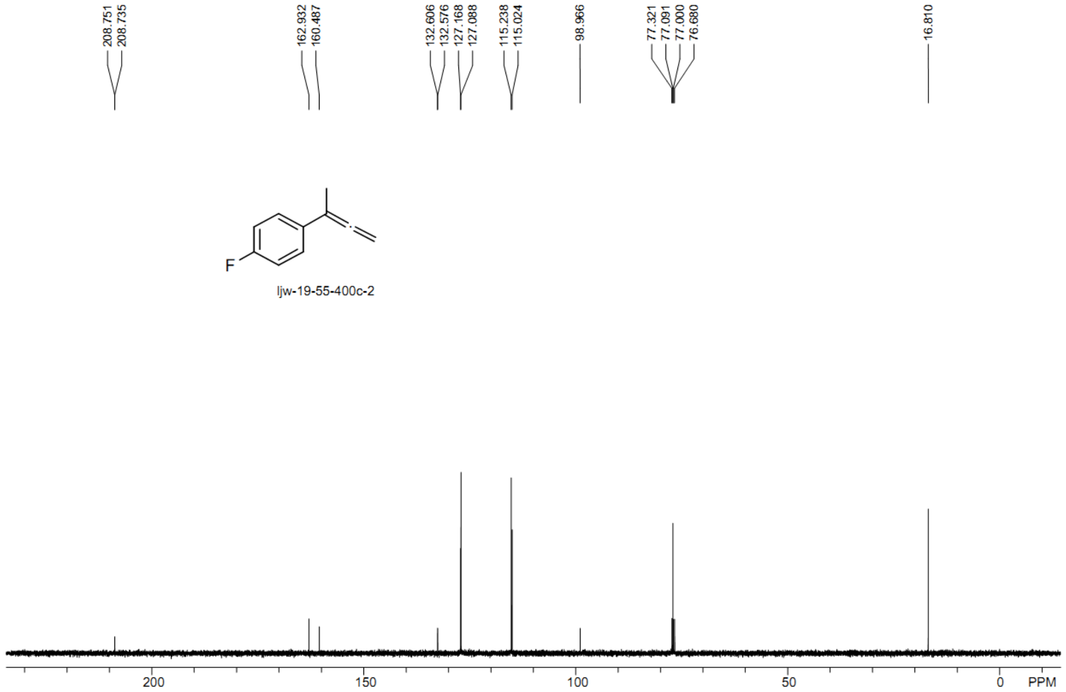

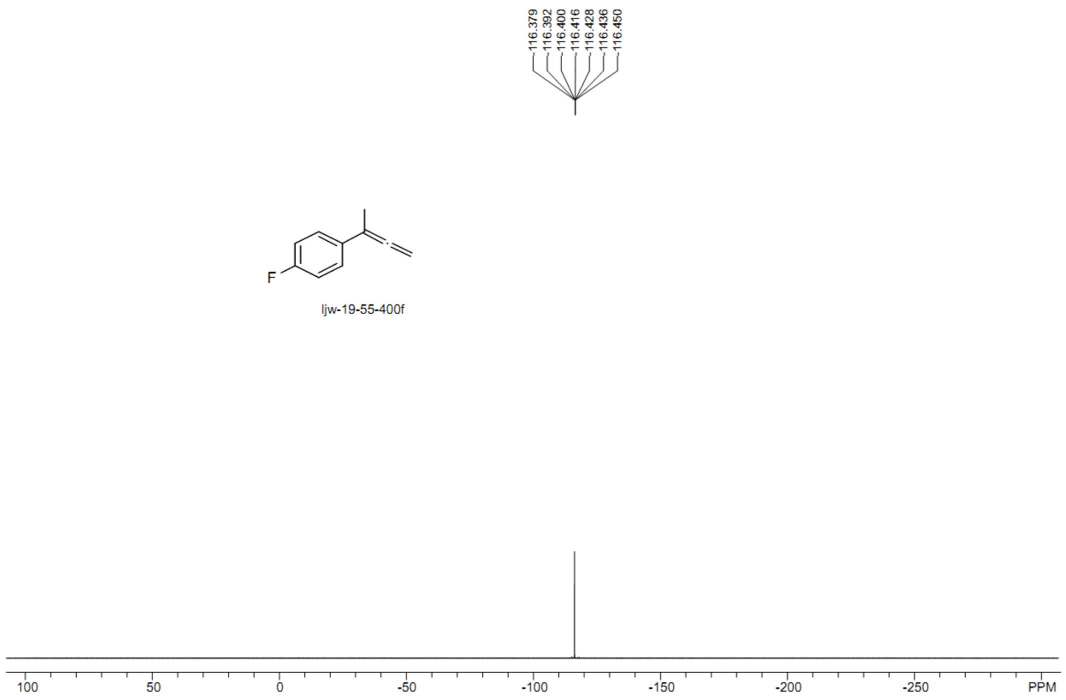

NMR spectra of 1e

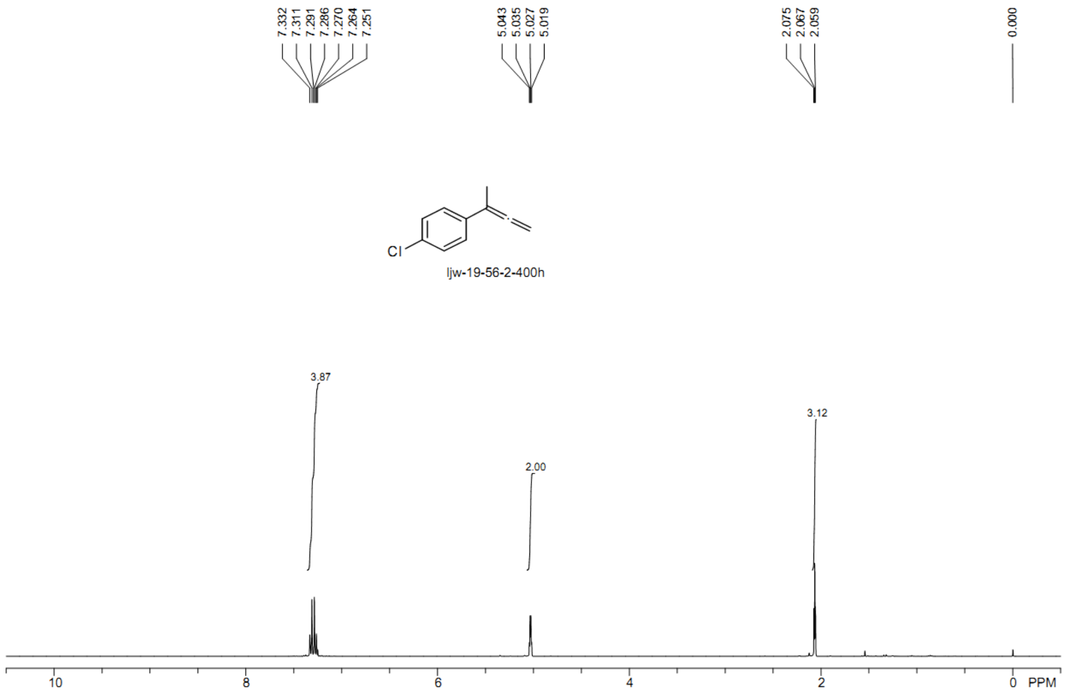

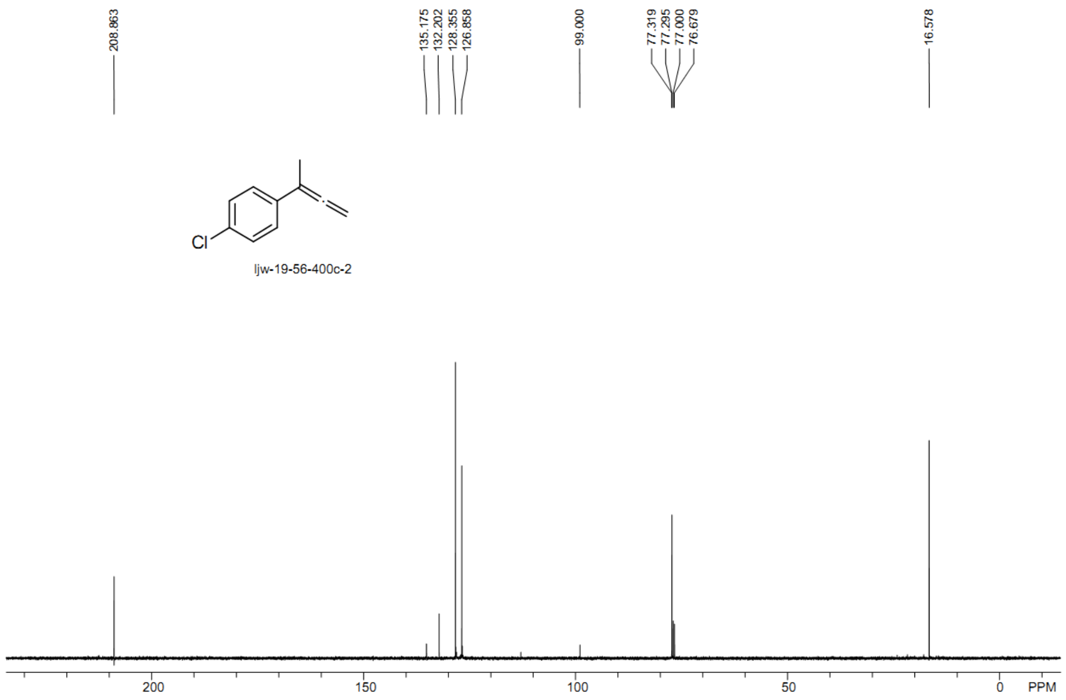

NMR spectra of 1f

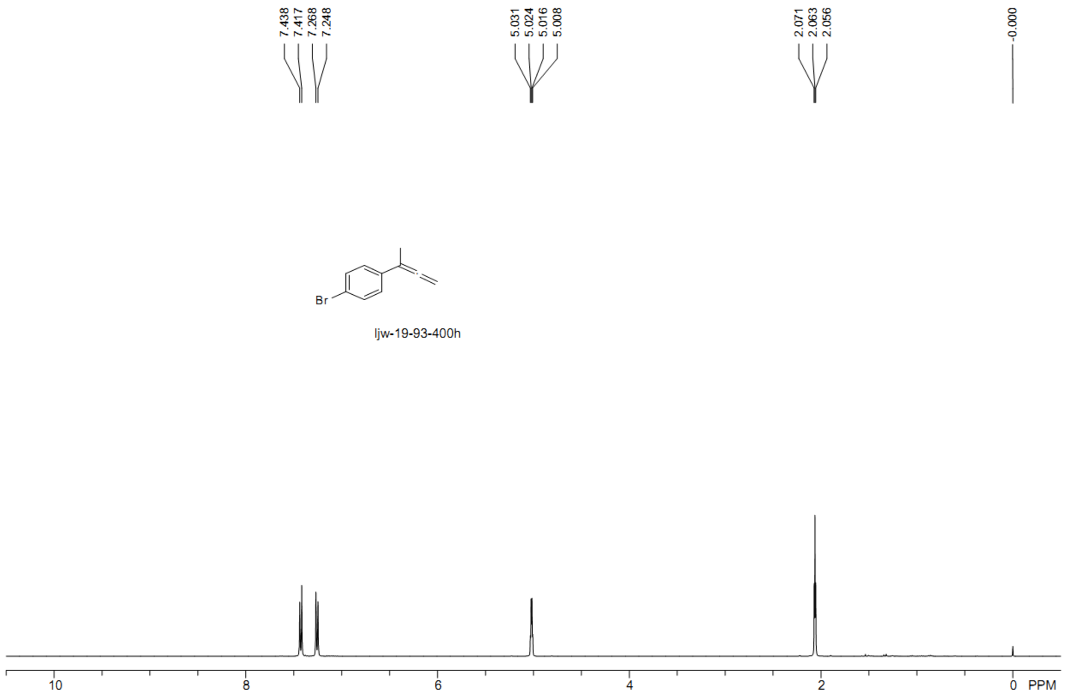

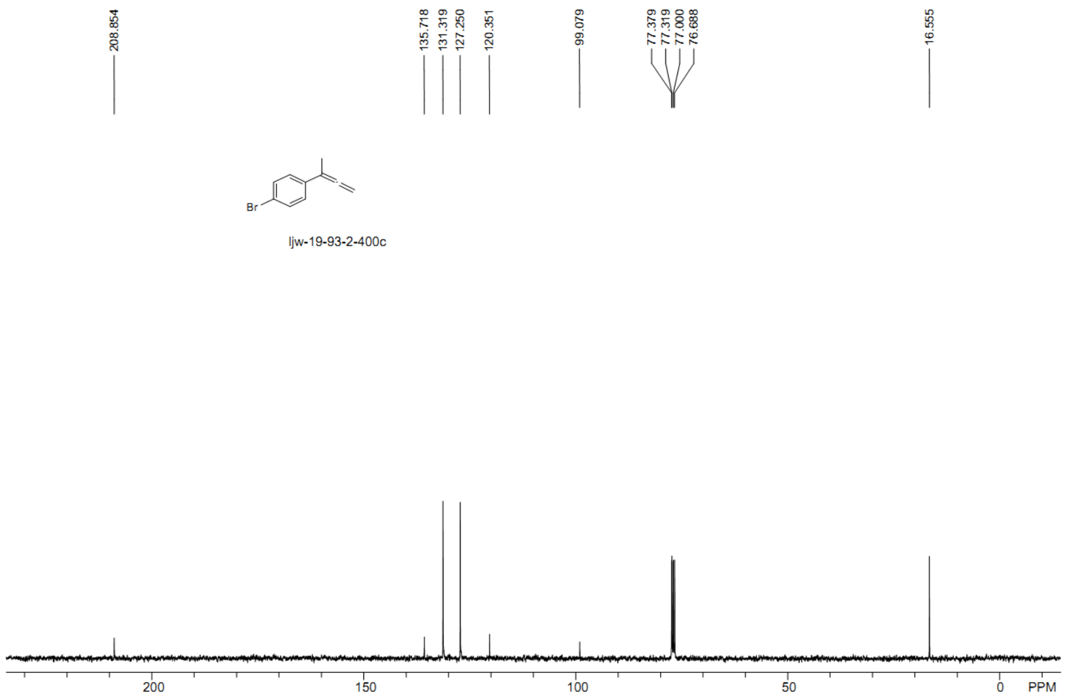

NMR spectra of 1g

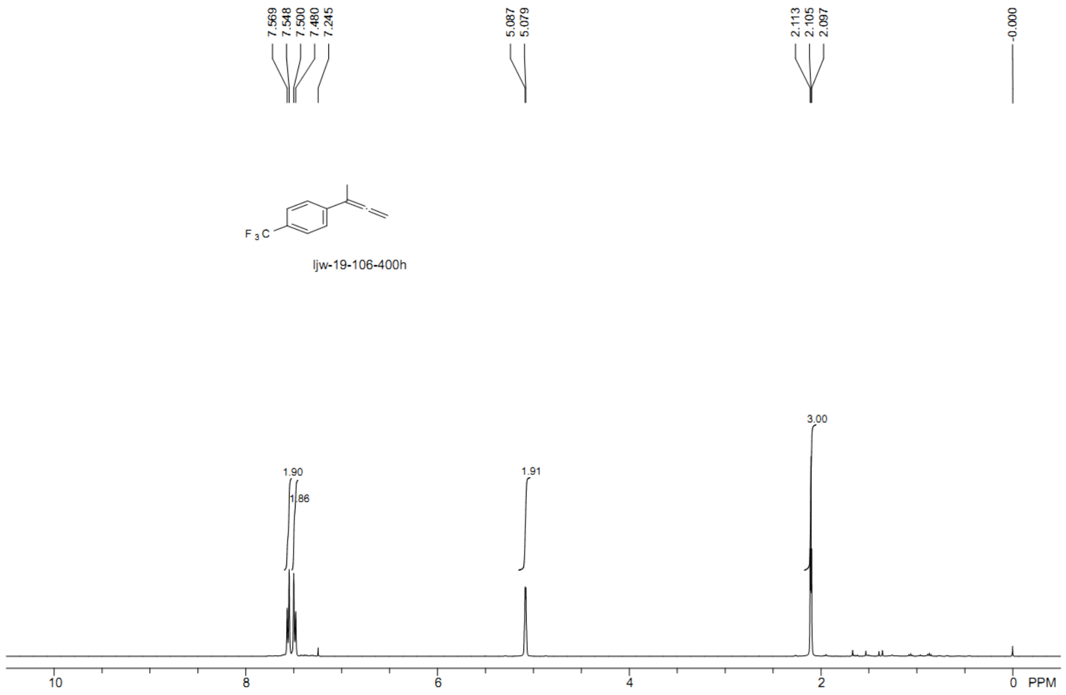

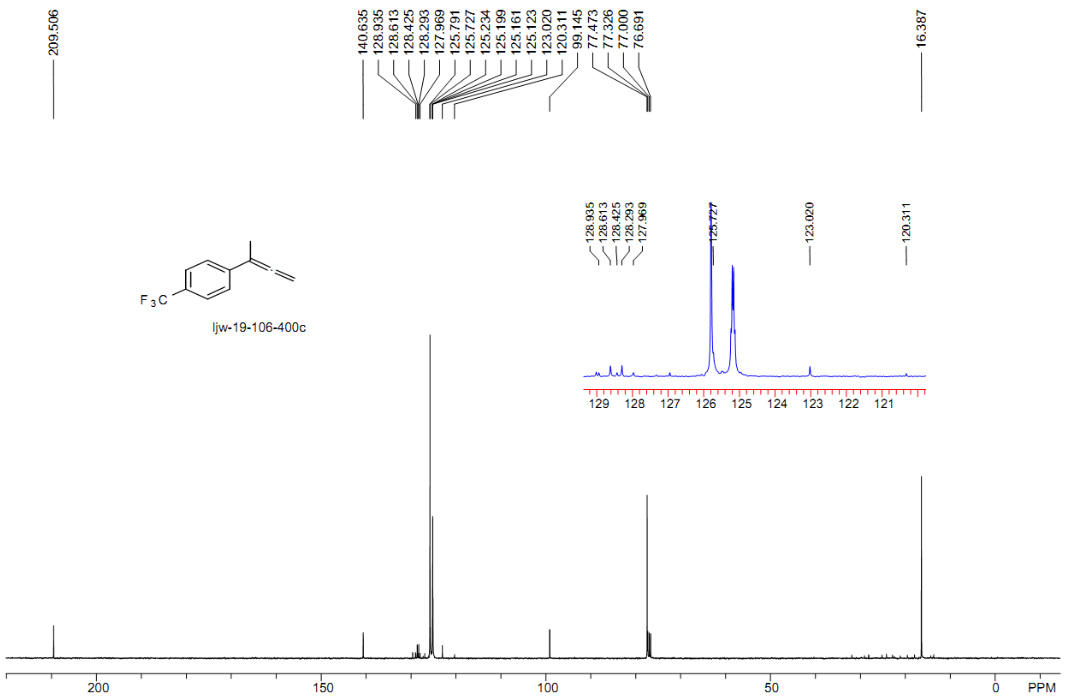
209.506
140.635
128.935
128.613
128.425
128.293
127.969
125.791
125.727
125.234
125.199
125.161
125.123
123.020
120.311
99.145
77.473
77.326
77.000
76.691
16.387
F3C
ljw-19-106-400c
200
150
100
50
0
PPM

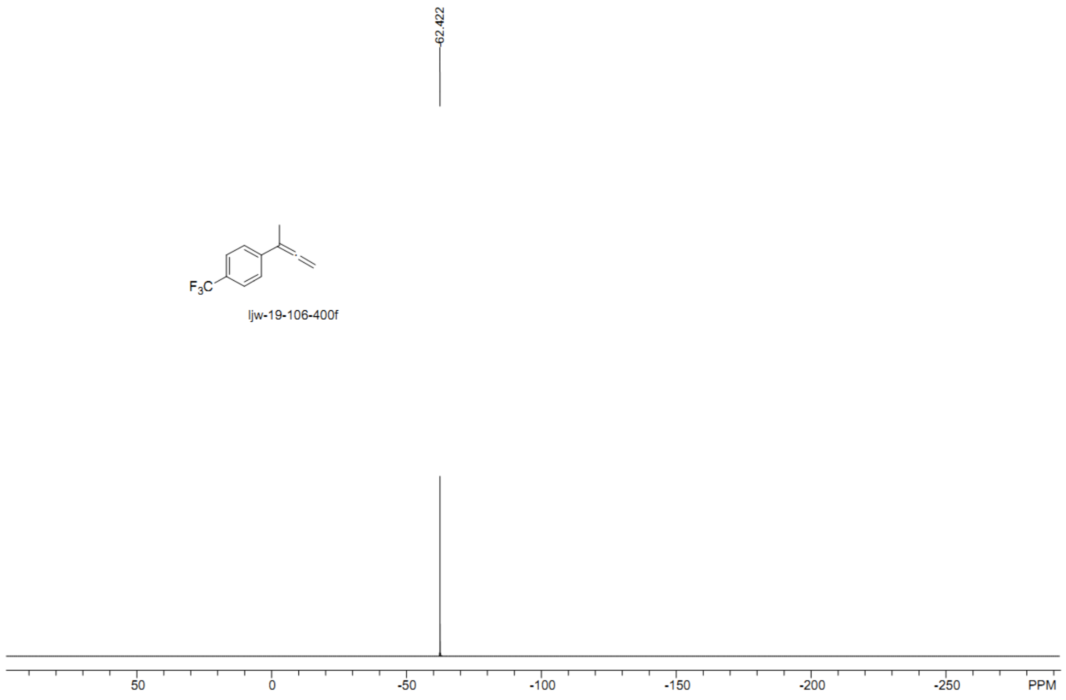
-62.422
F3C
ljw-19-106-400f
50
0
-50
-100
-150
-200
-250
PPM

NMR spectra of 1h

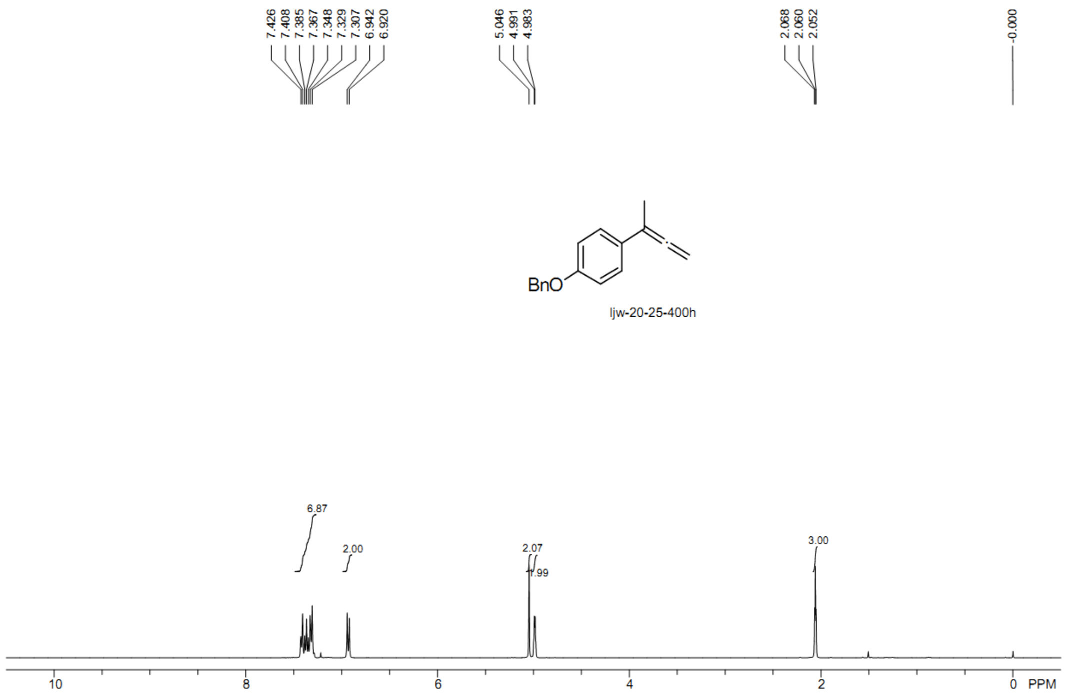

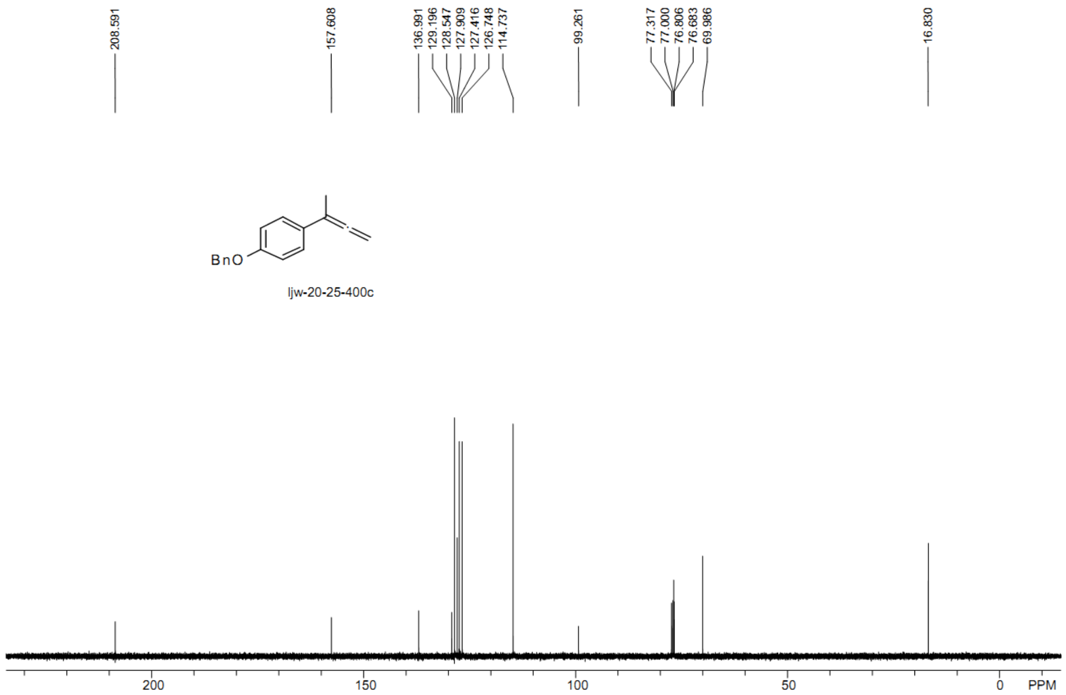

NMR spectra of 1i

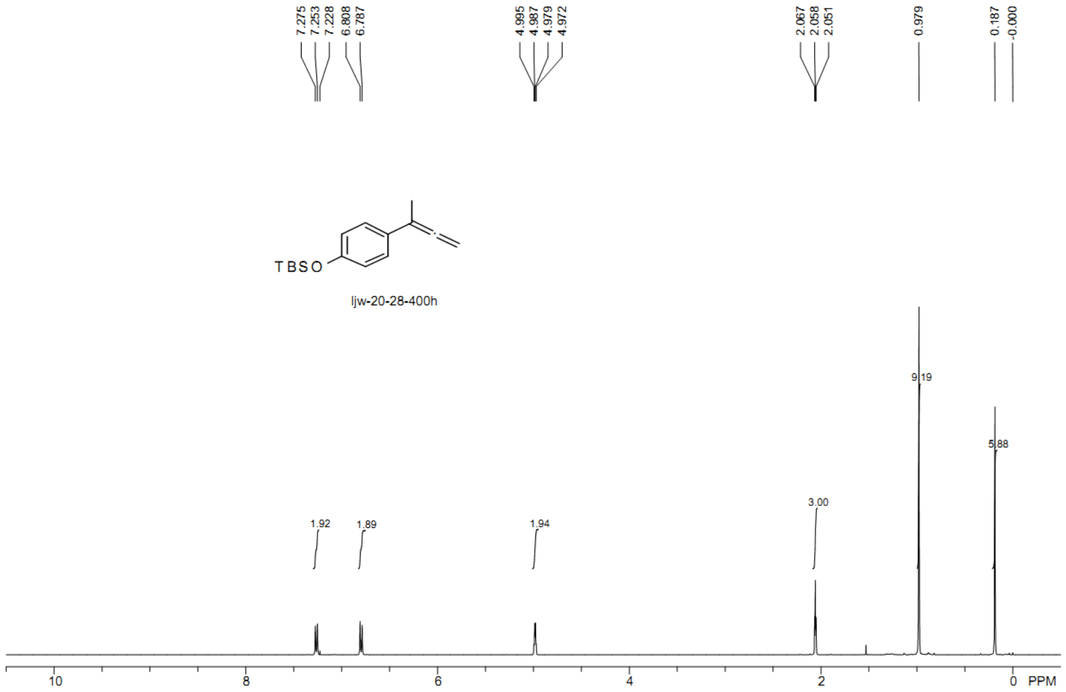

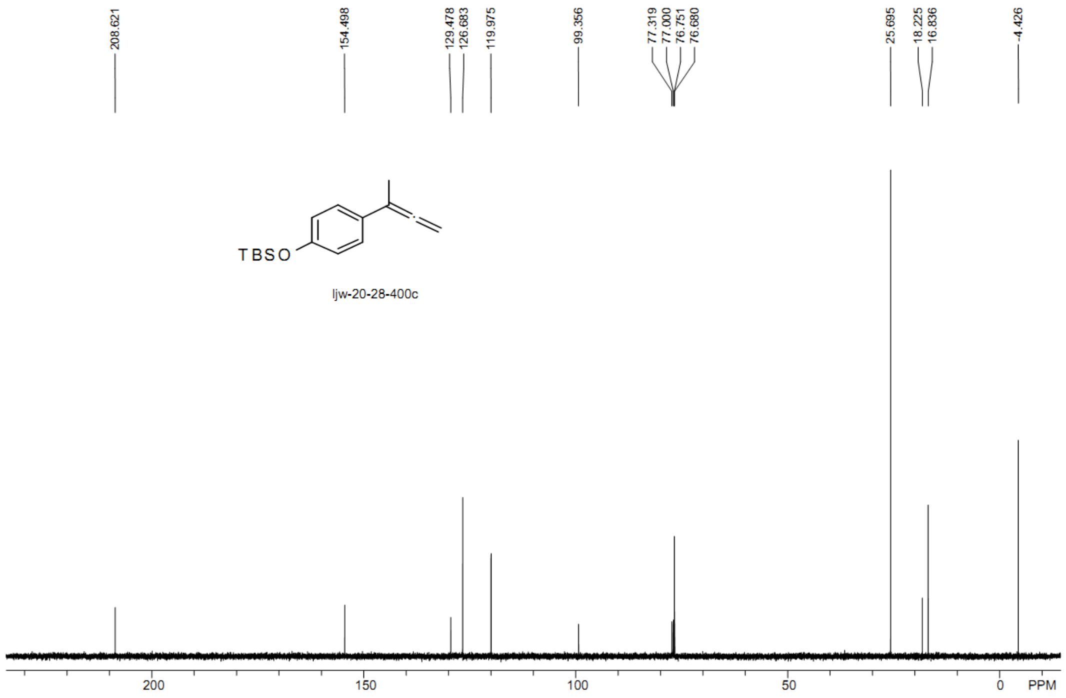

NMR spectra of 1j

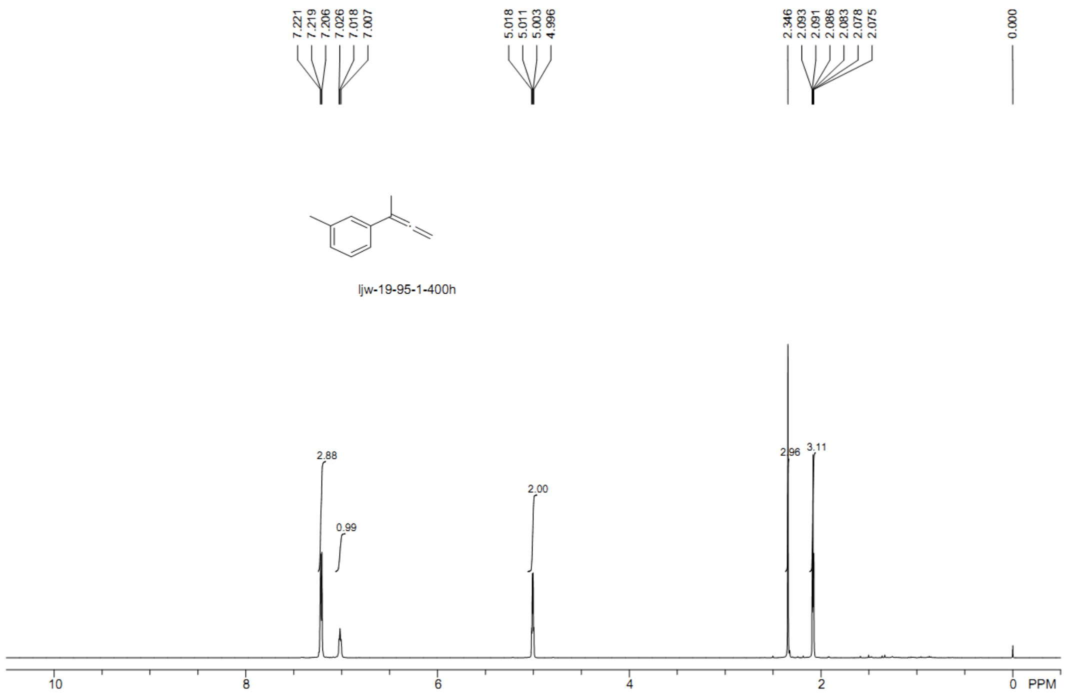

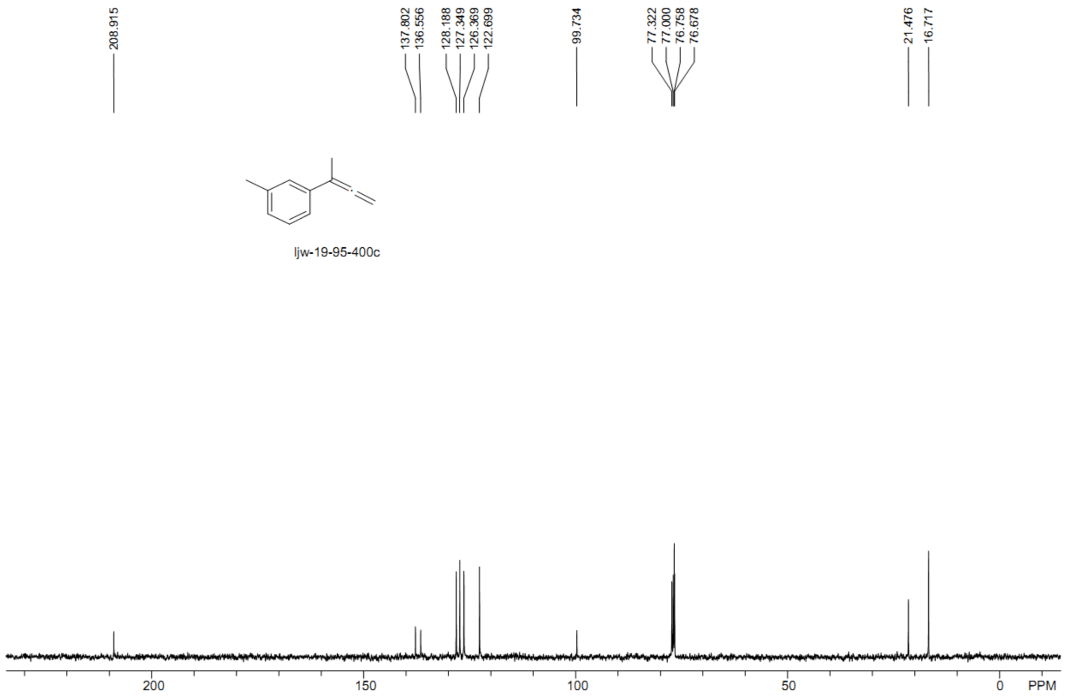

NMR spectra of 1k

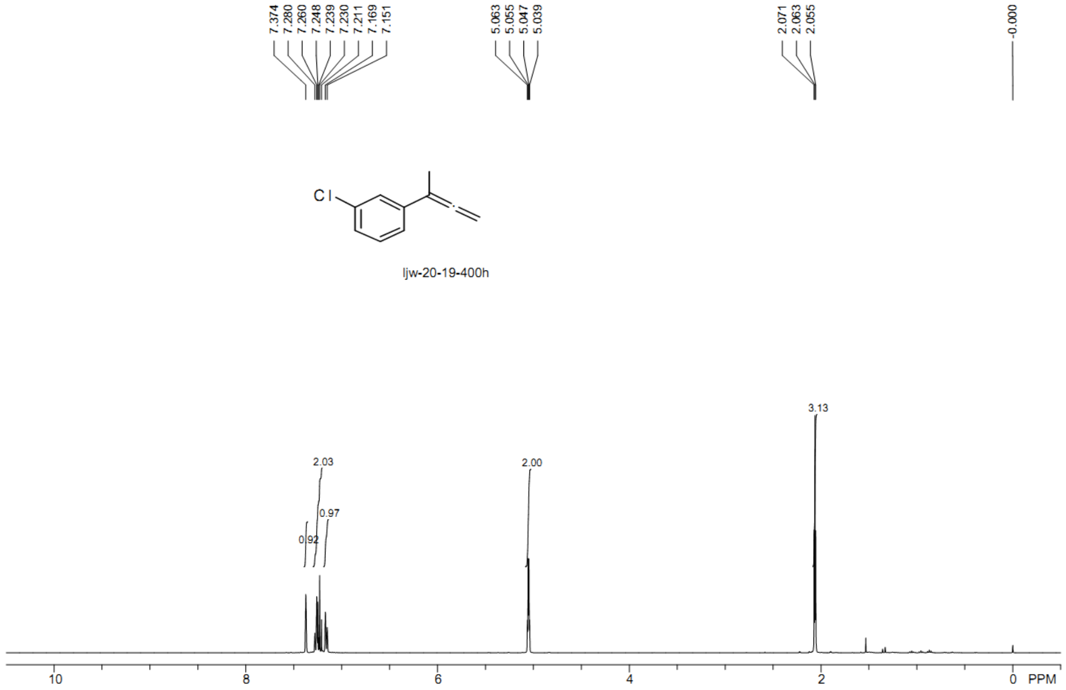

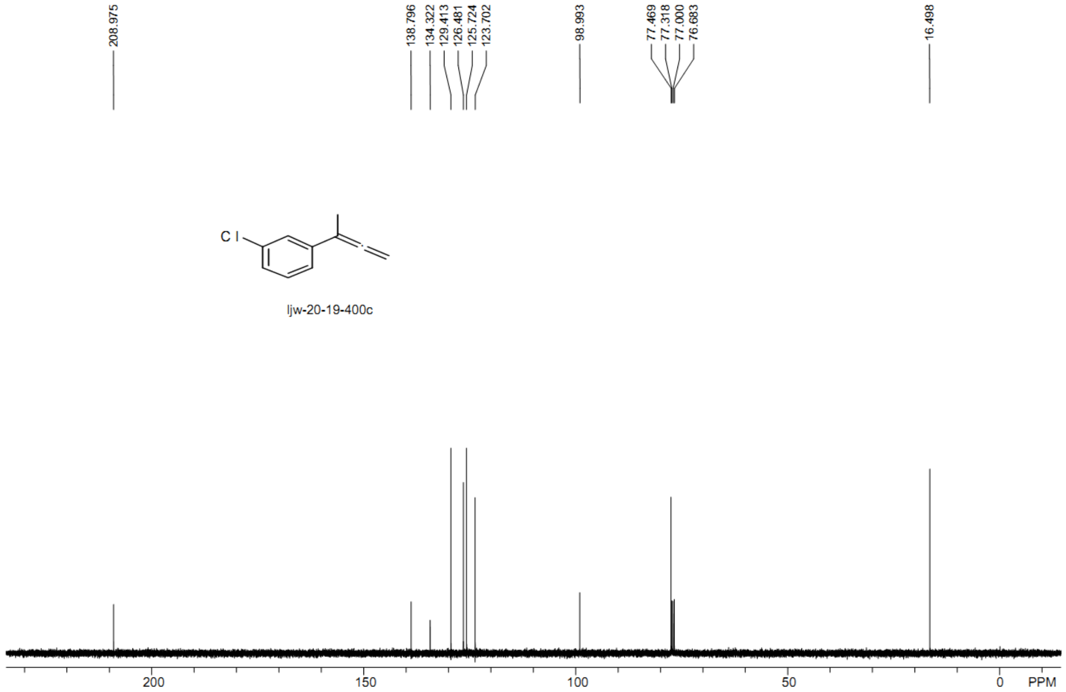

NMR spectra of 1l

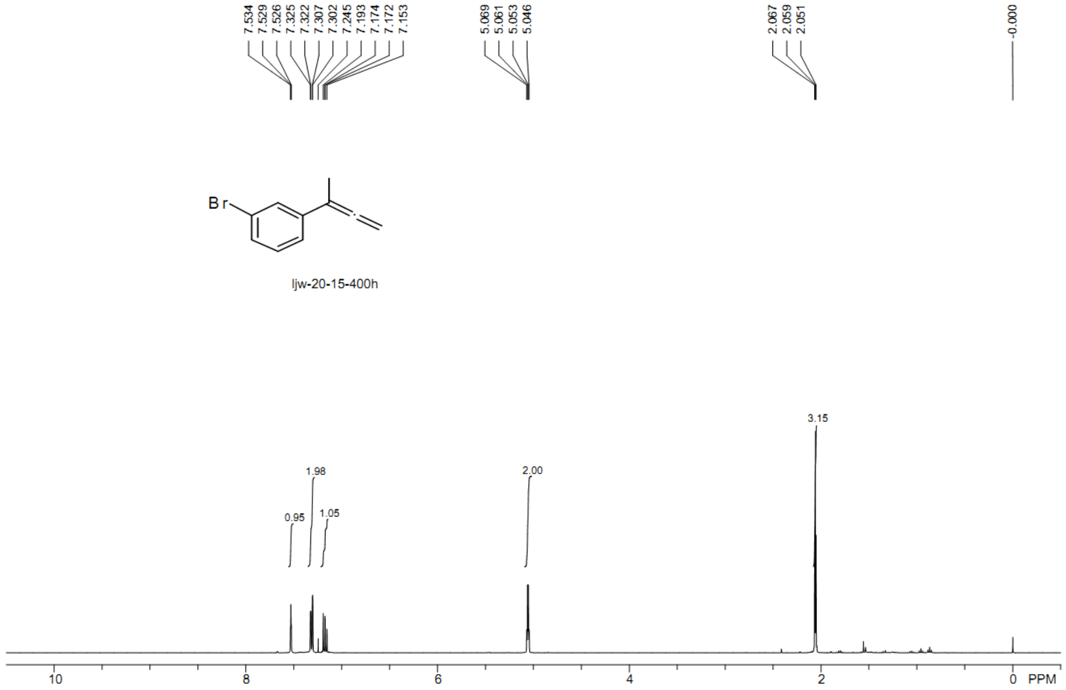

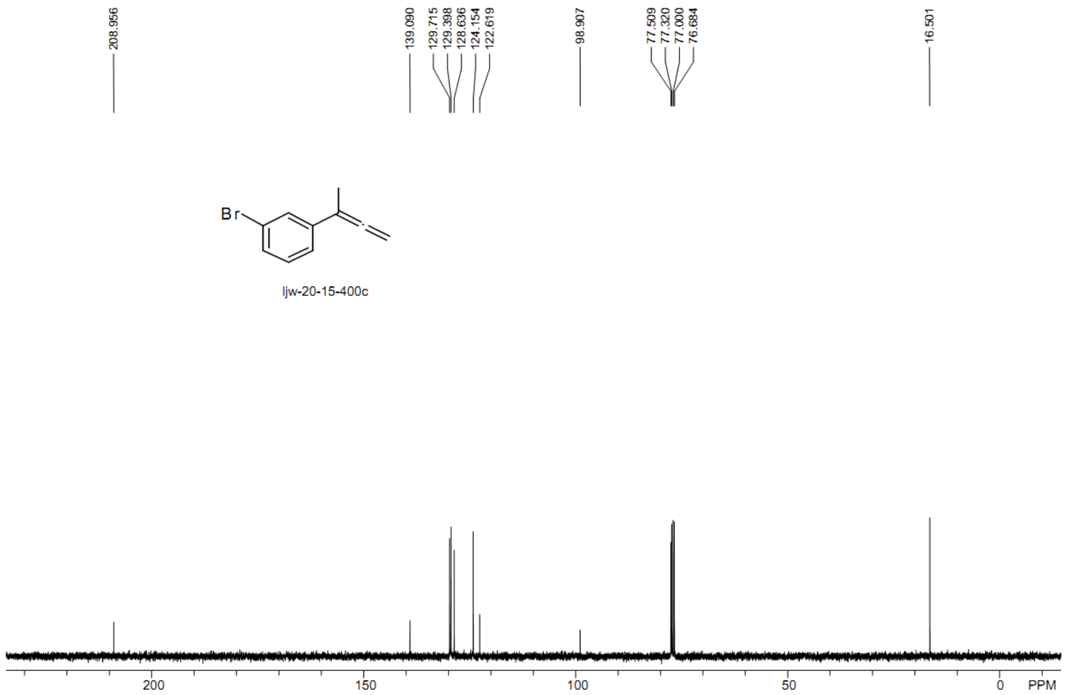

NMR spectra of 1m

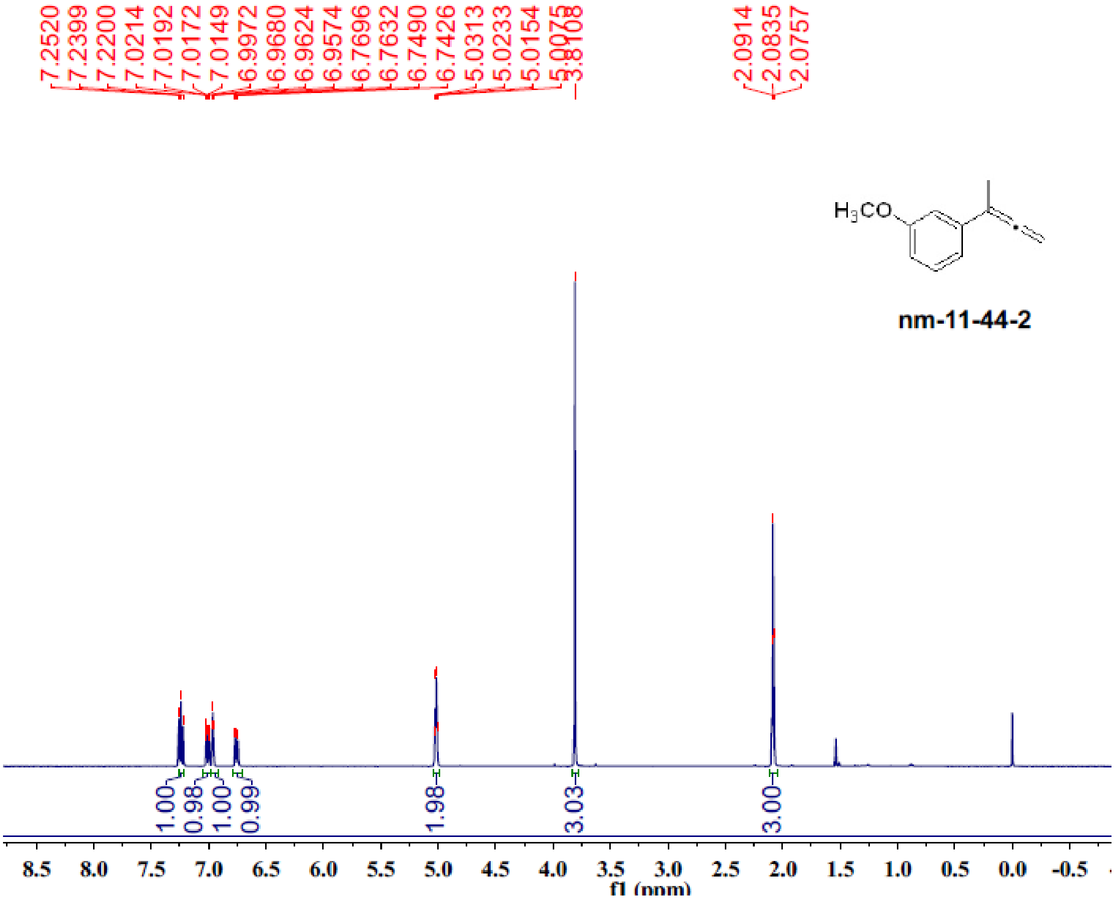

NMR spectra of 1n

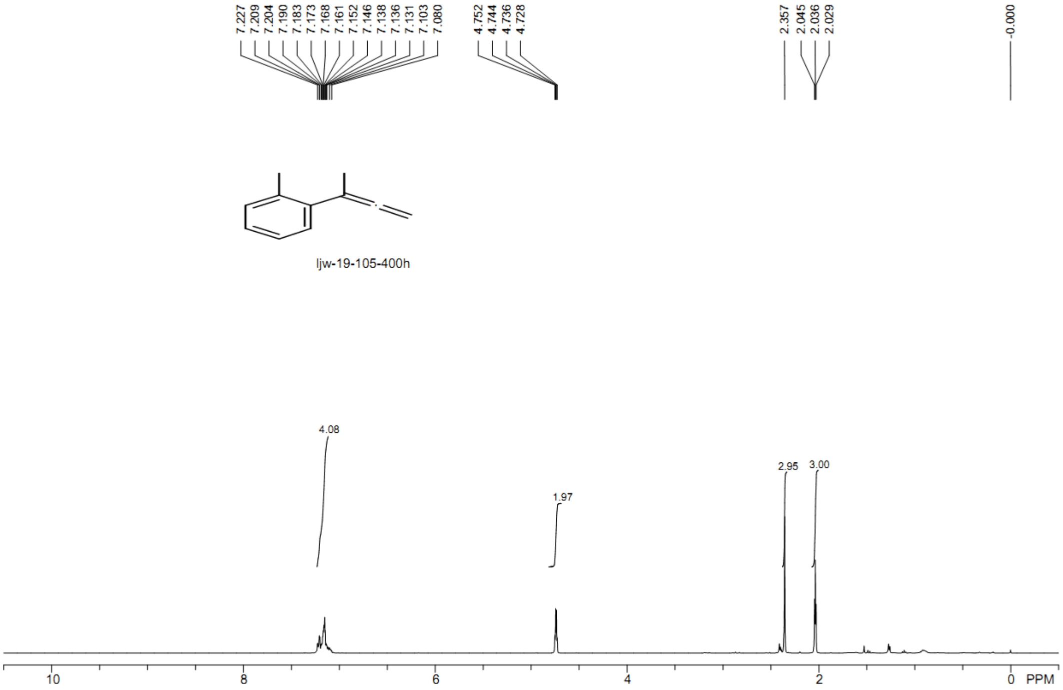

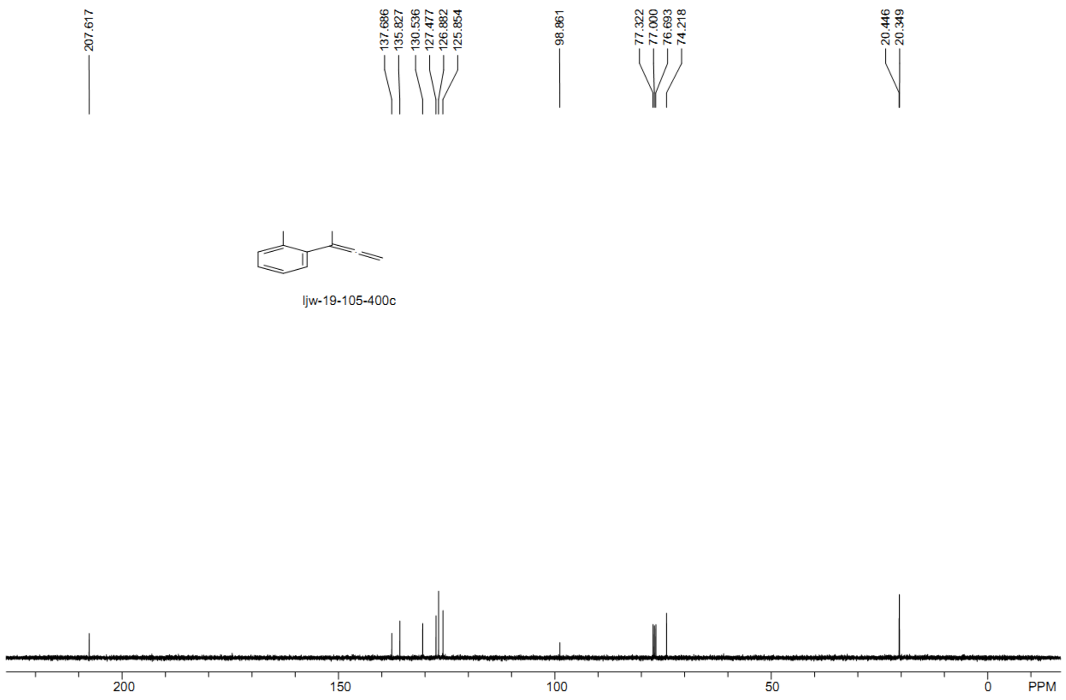

NMR spectra of 1o

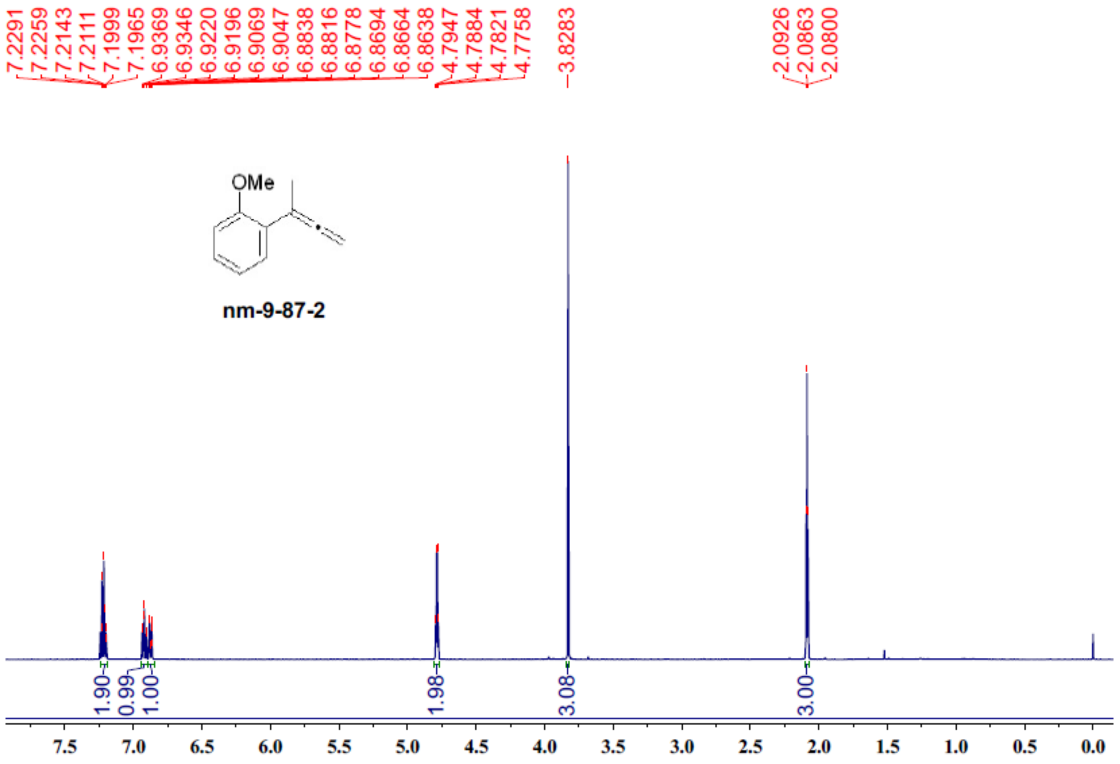

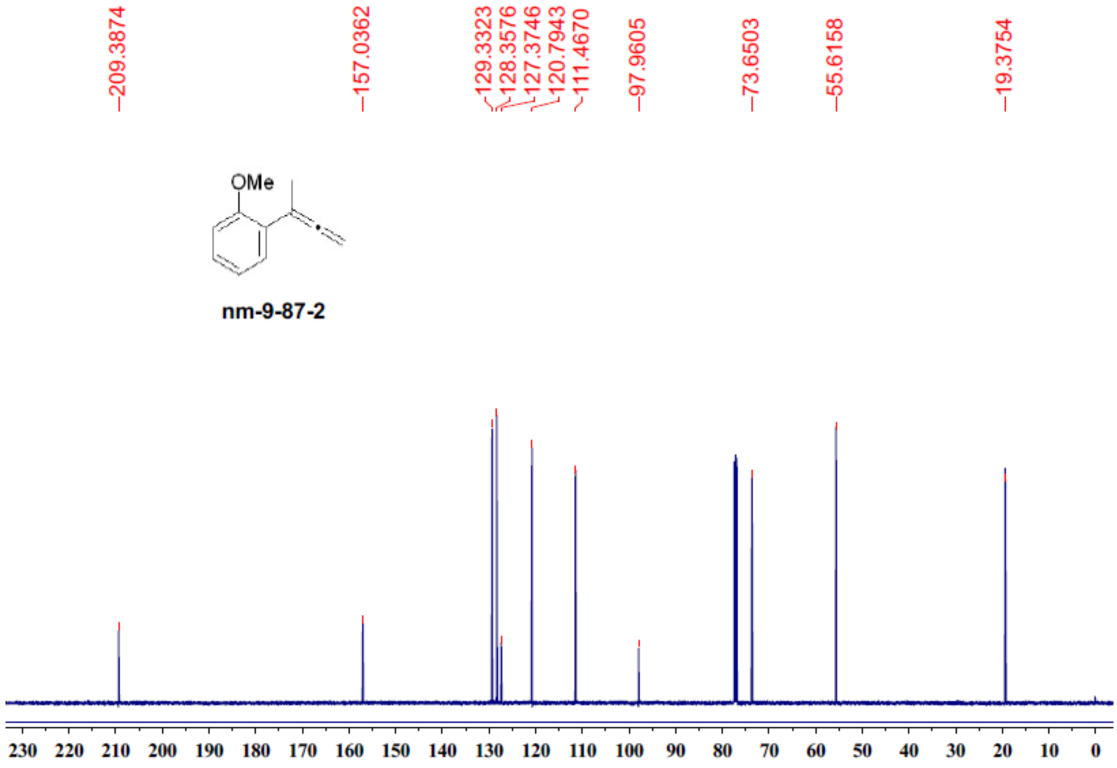

NMR spectra of 1p

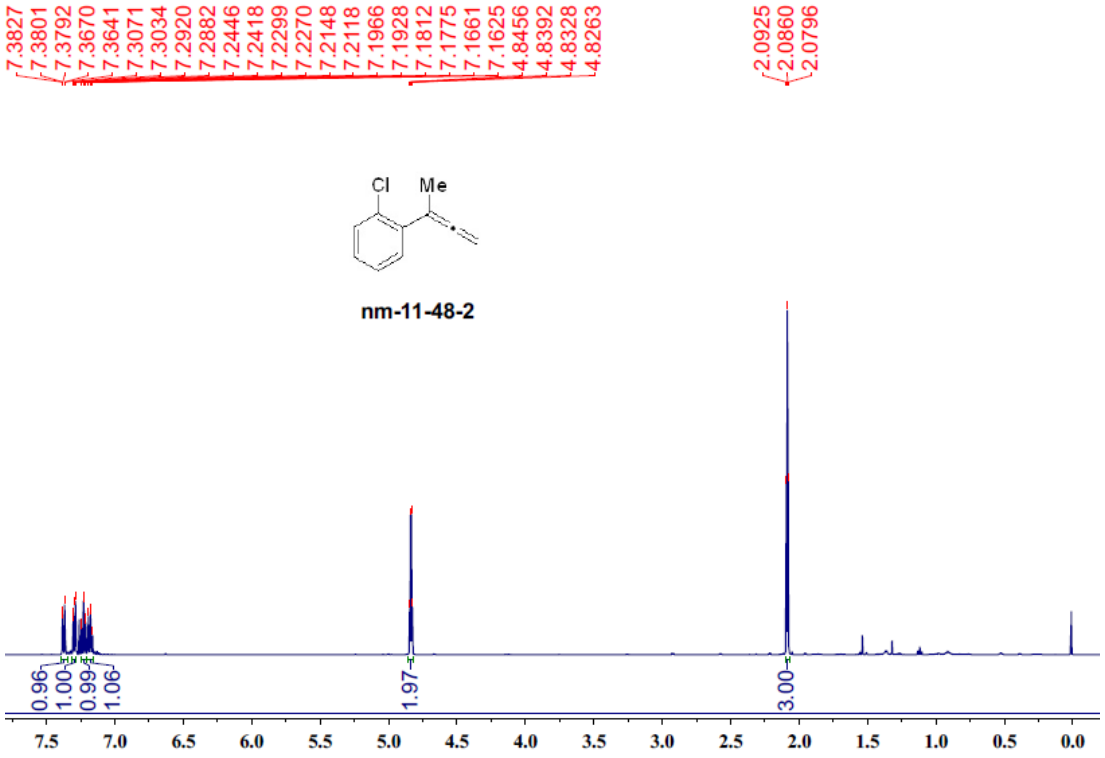

NMR spectra of 1q

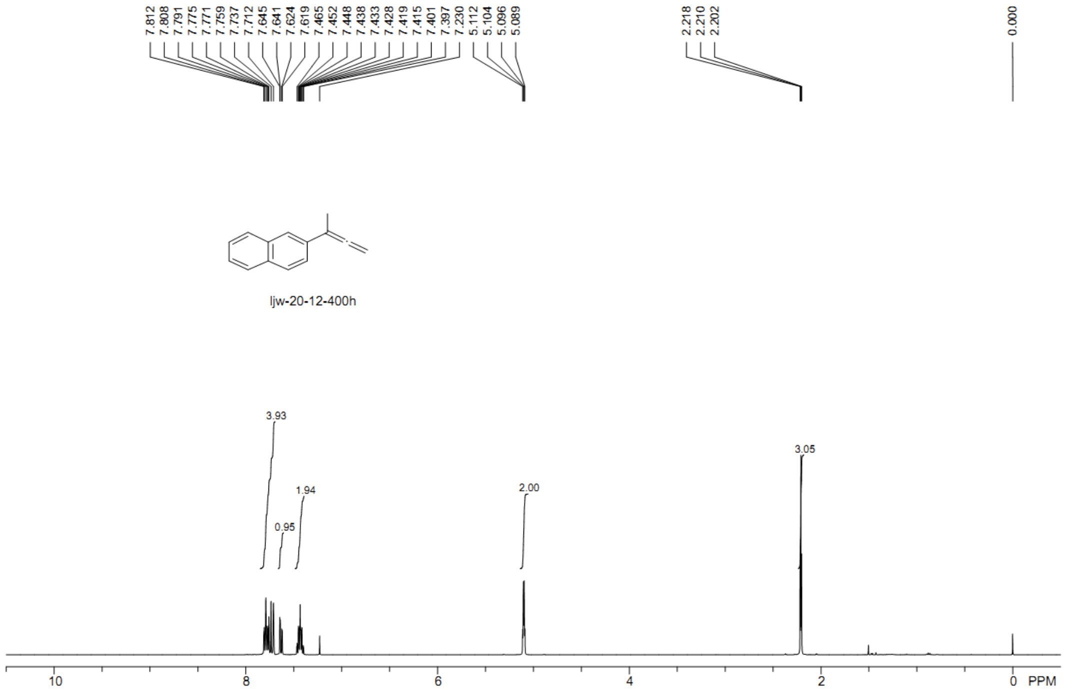

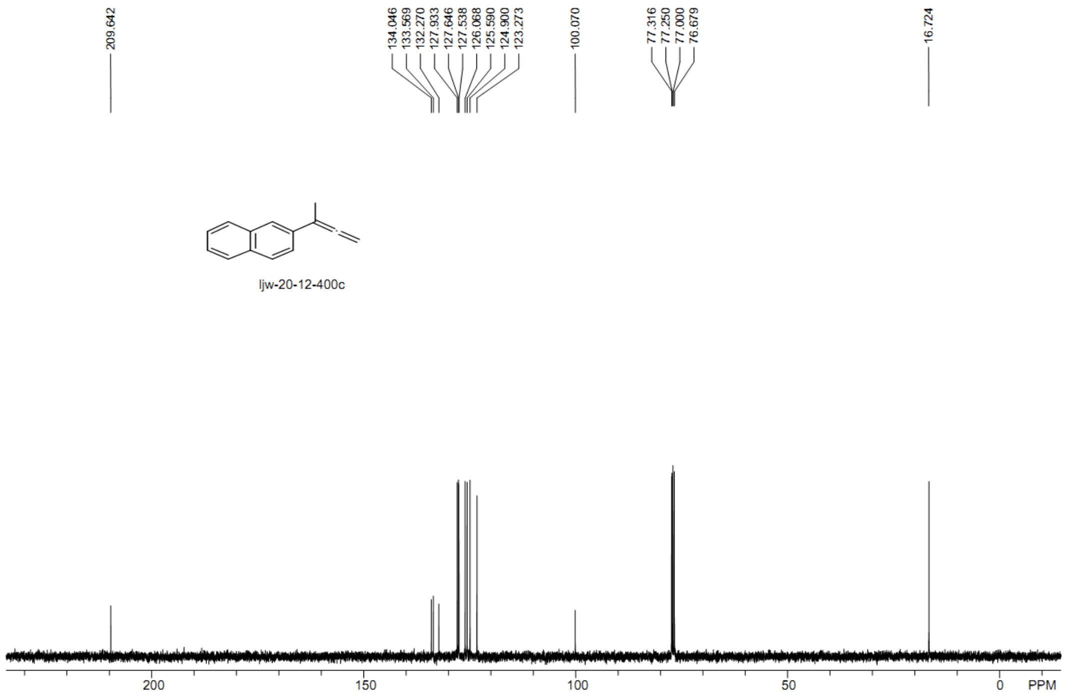

NMR spectra of 1r

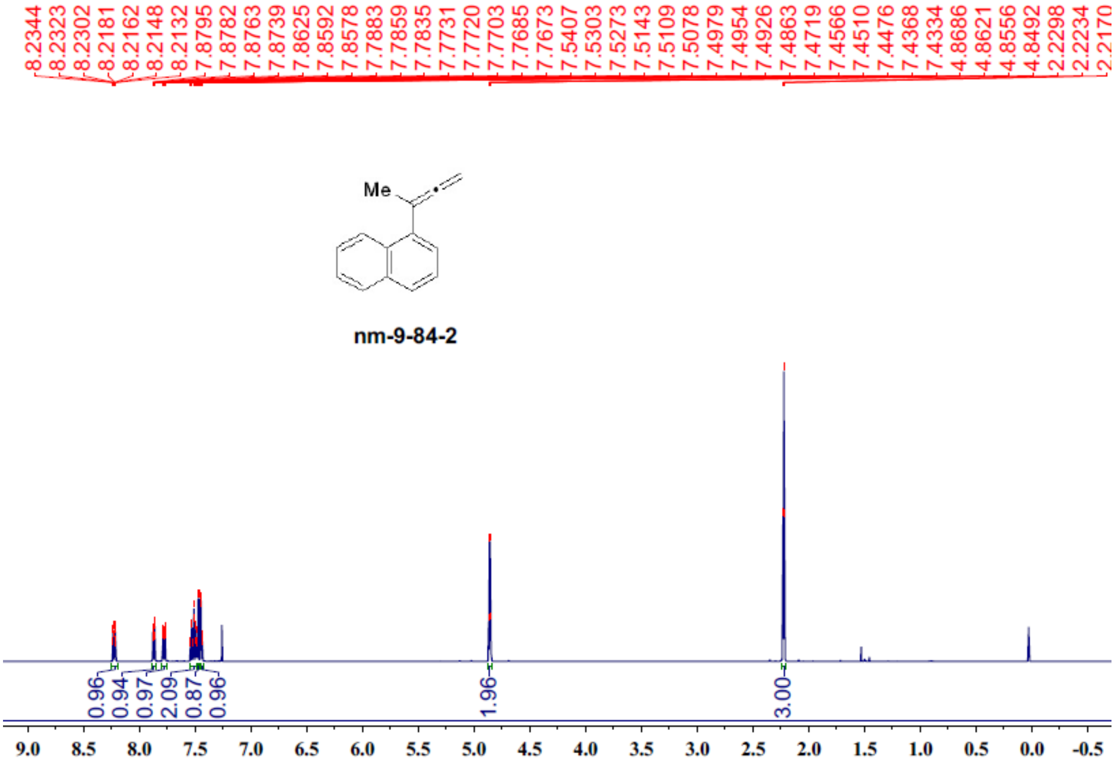

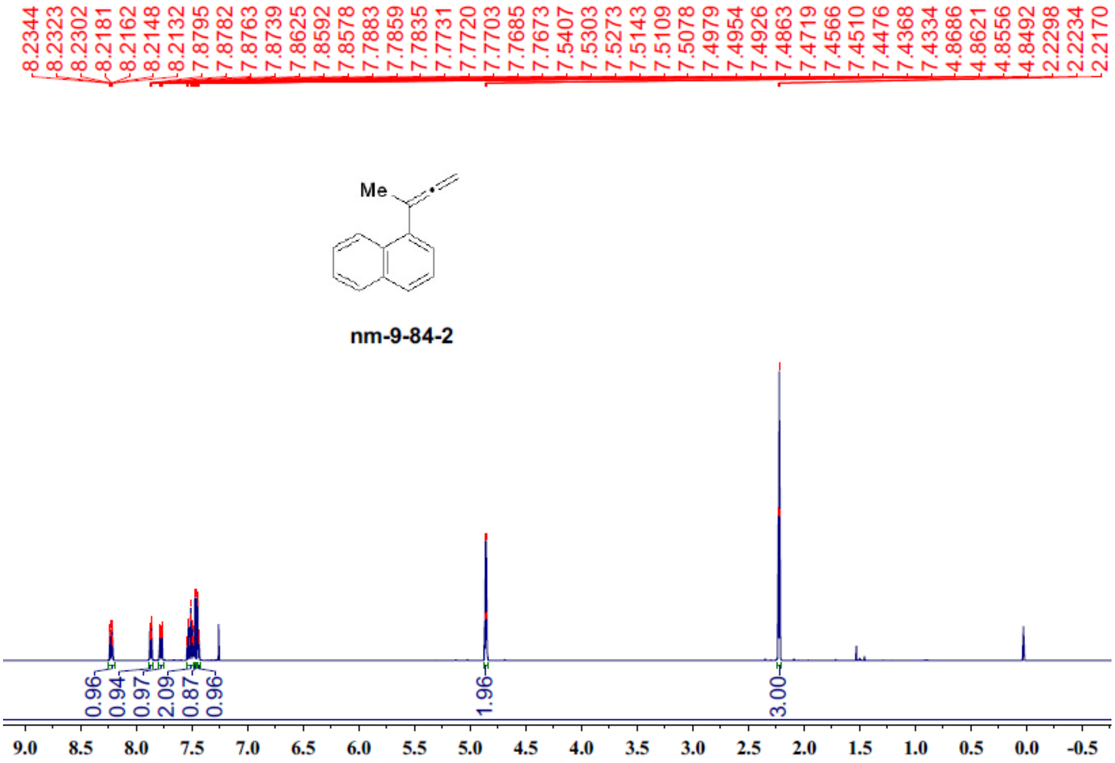

NMR spectra of 1s

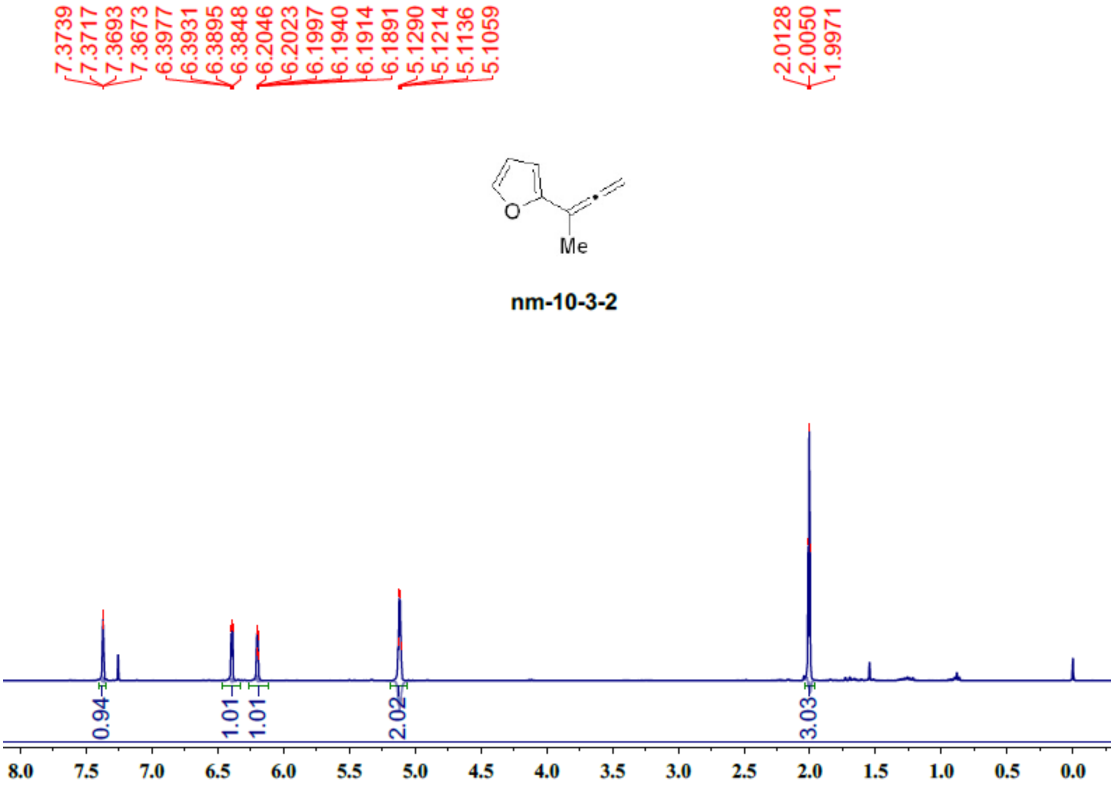

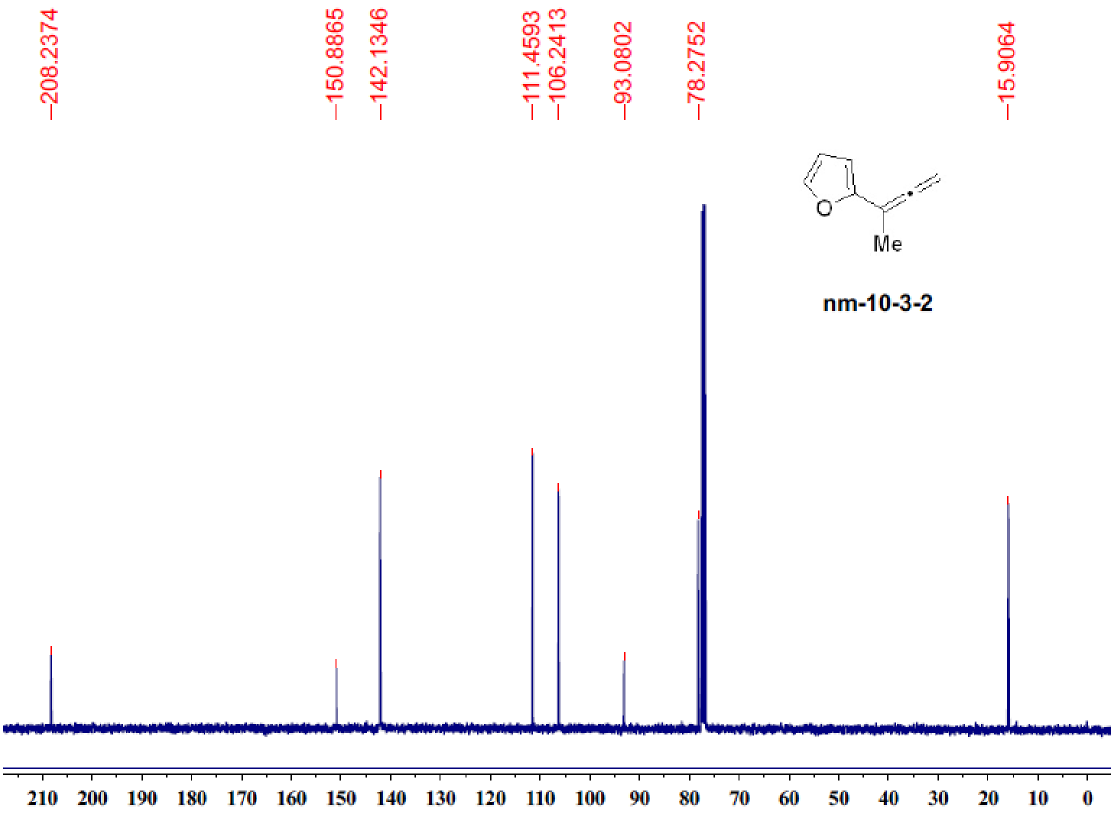

NMR spectra of 1t

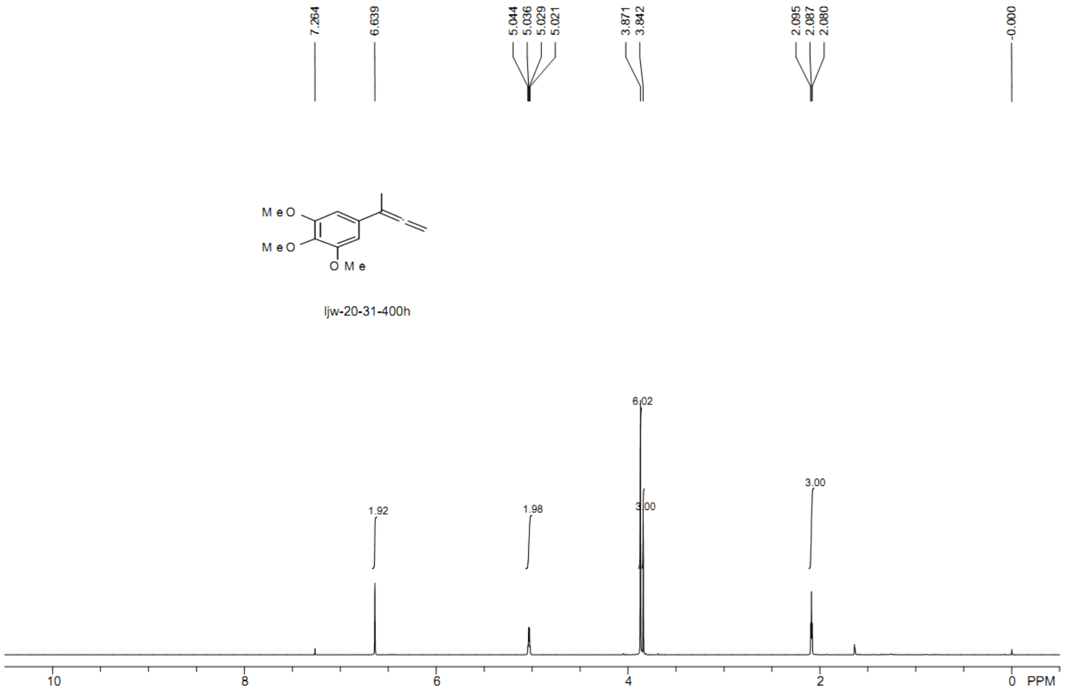

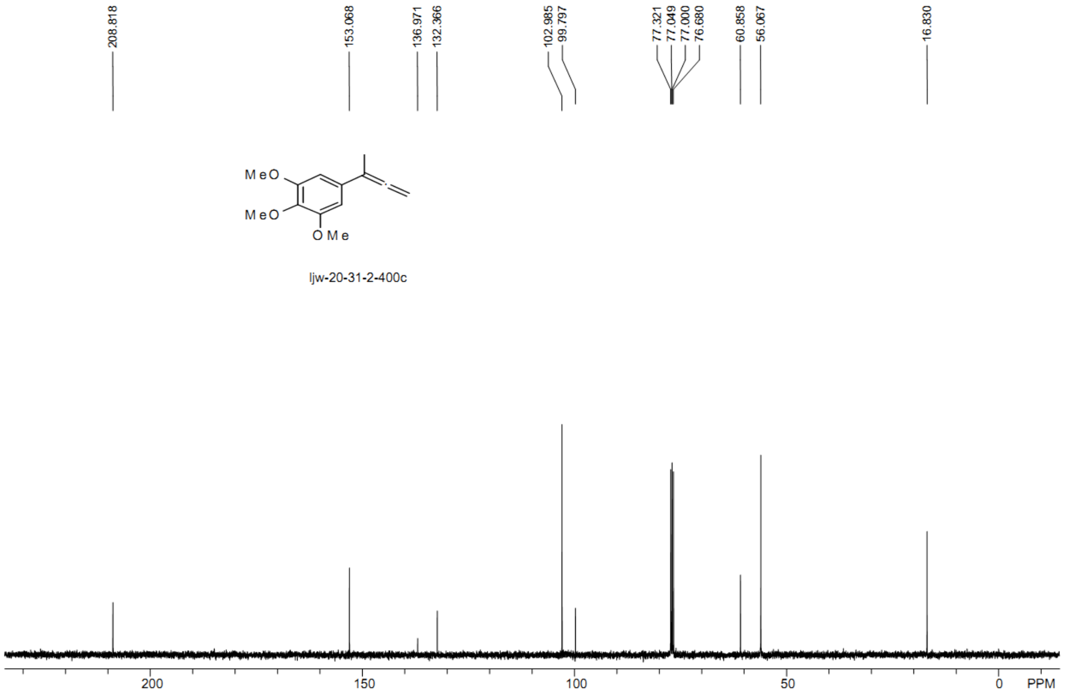

NMR spectra of 1u

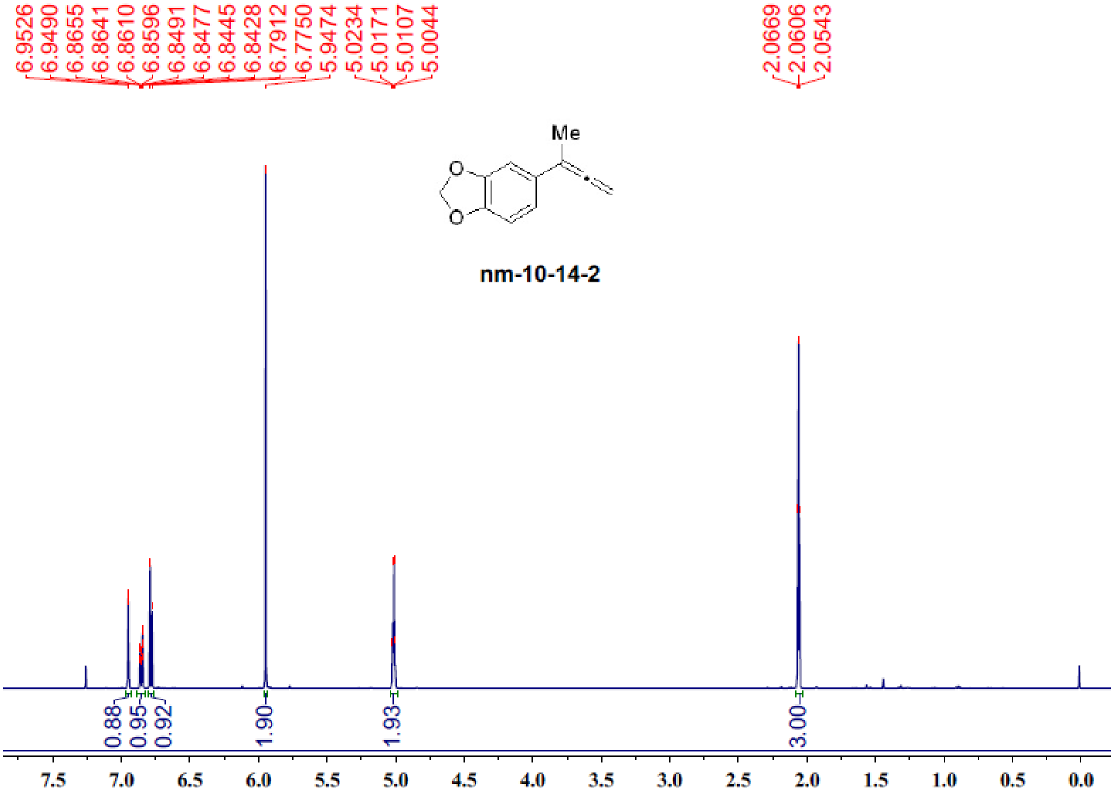

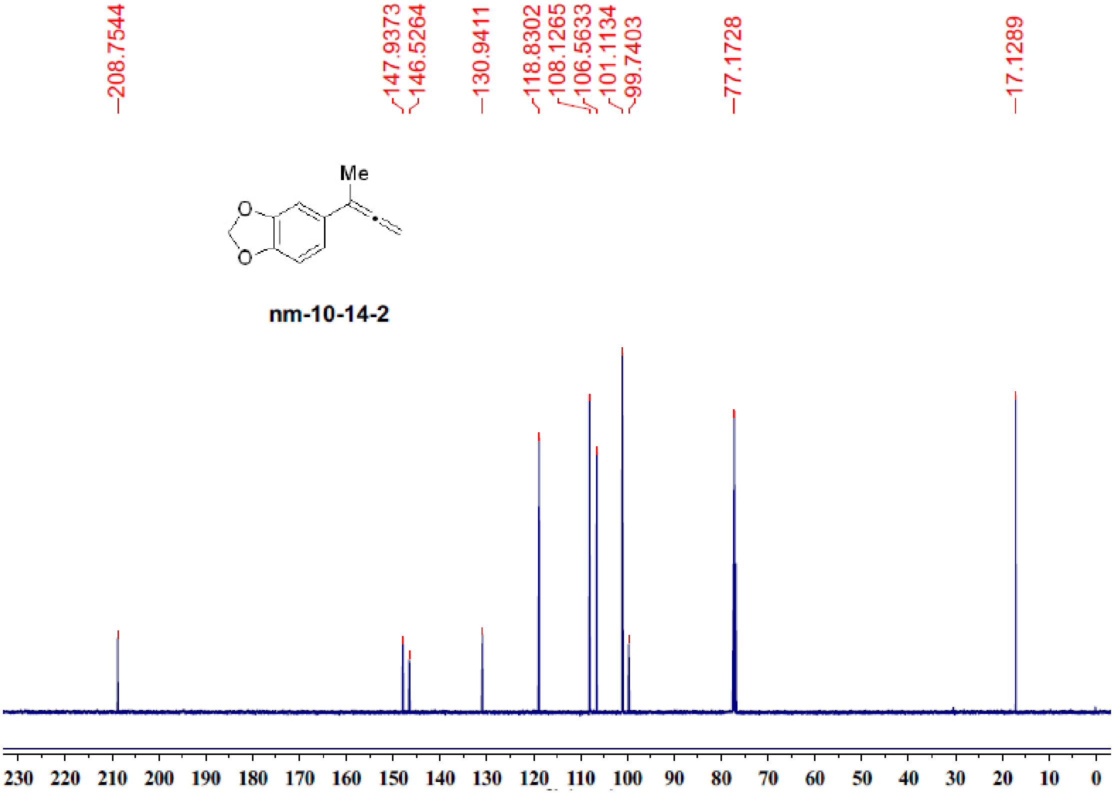

NMR spectra of 1v

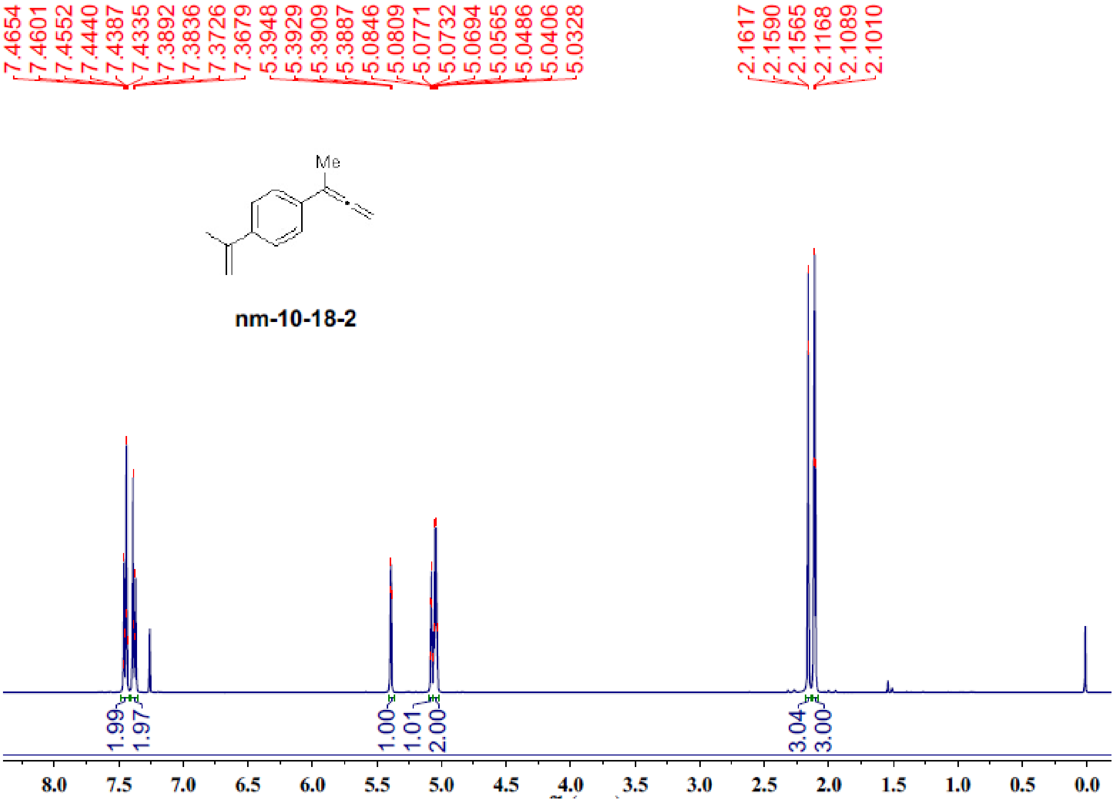

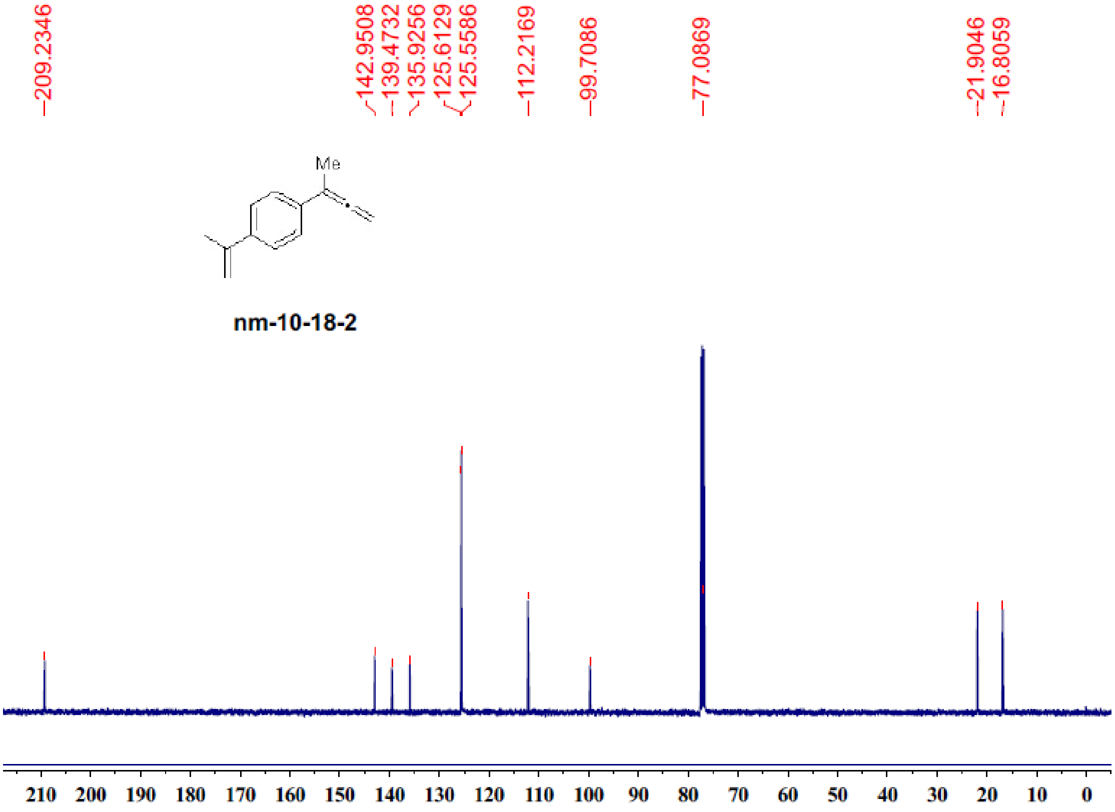

NMR spectra of 1w

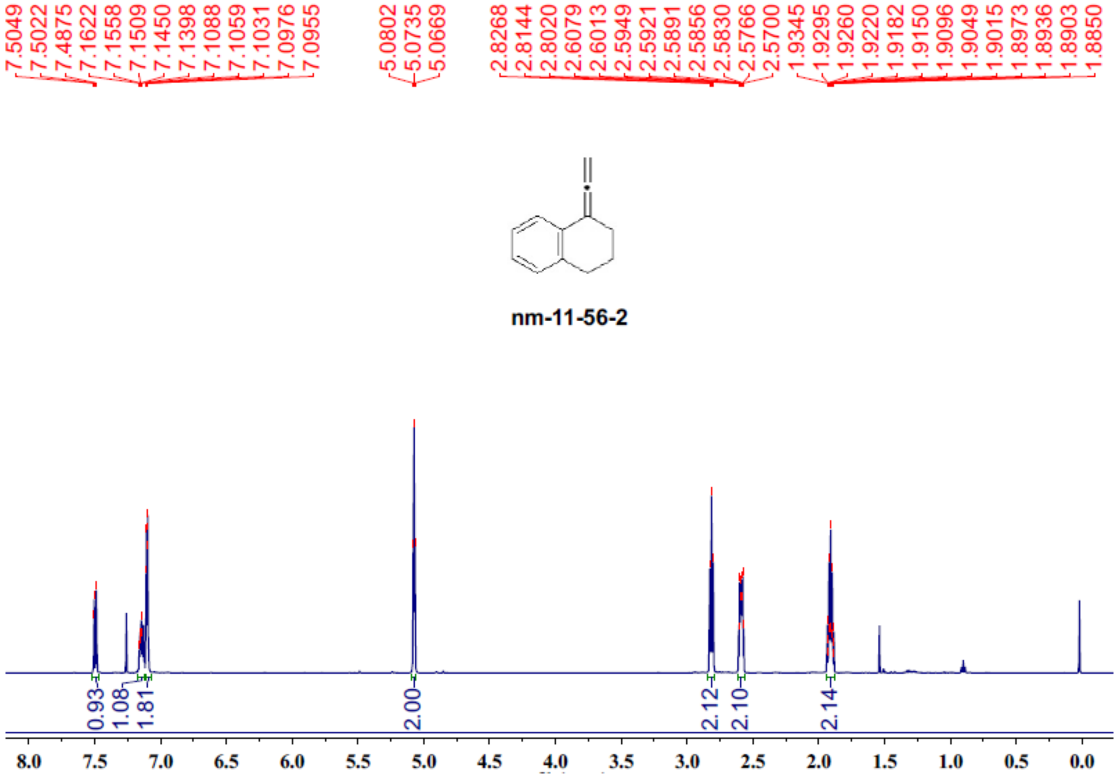

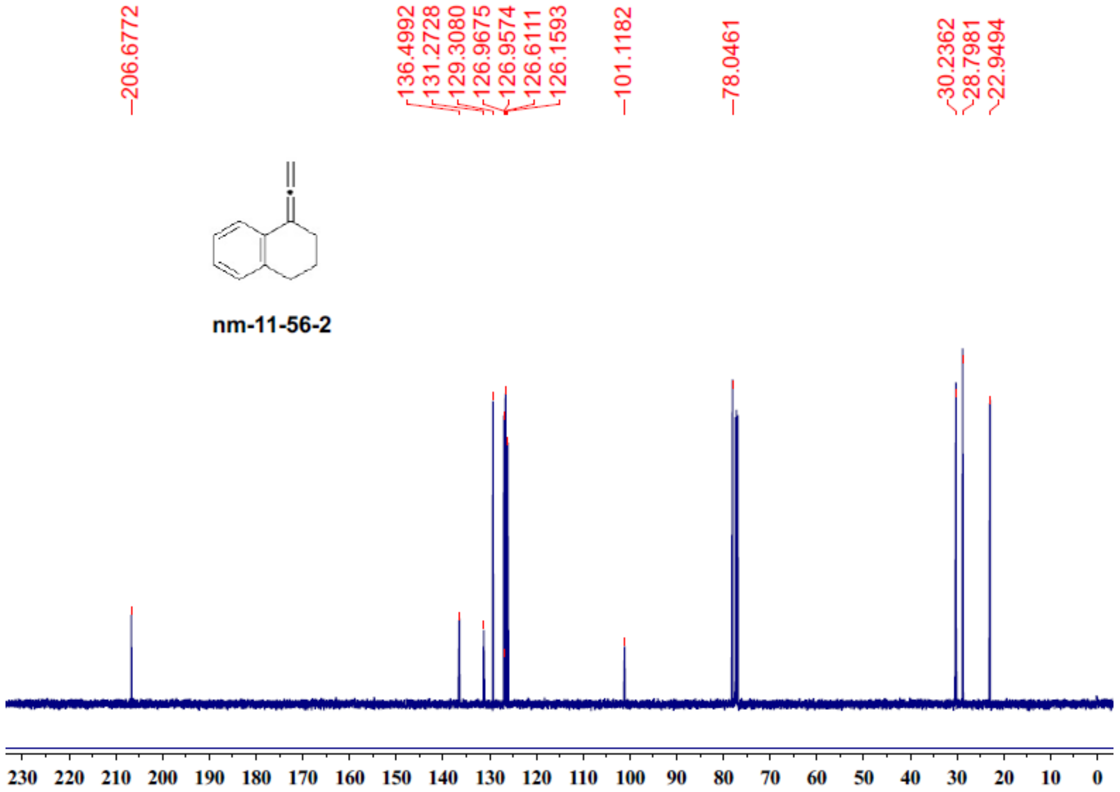

NMR spectra of 1x

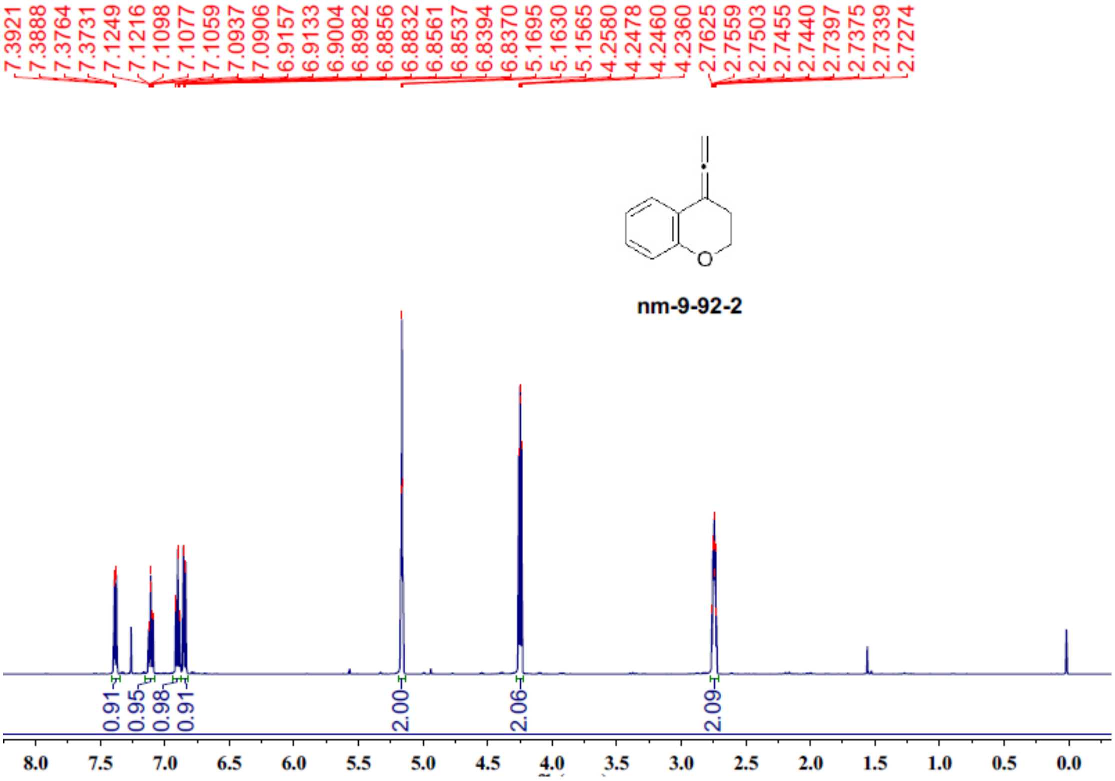

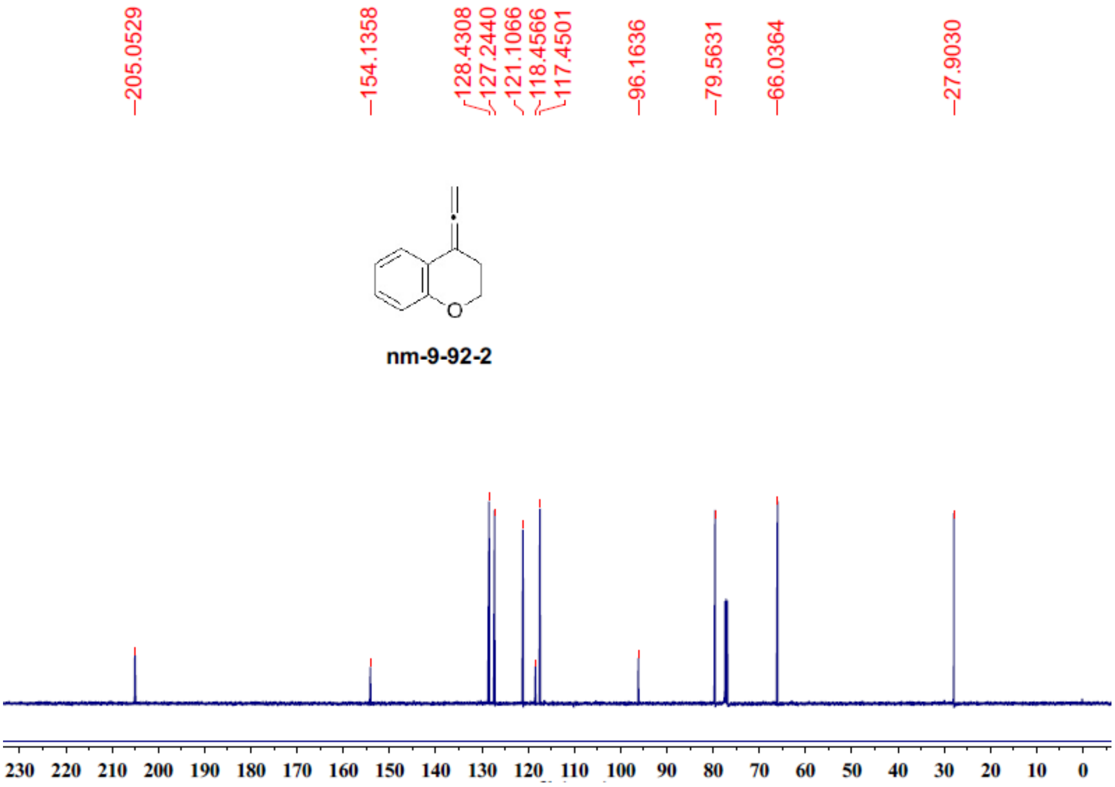

NMR spectra of 1y

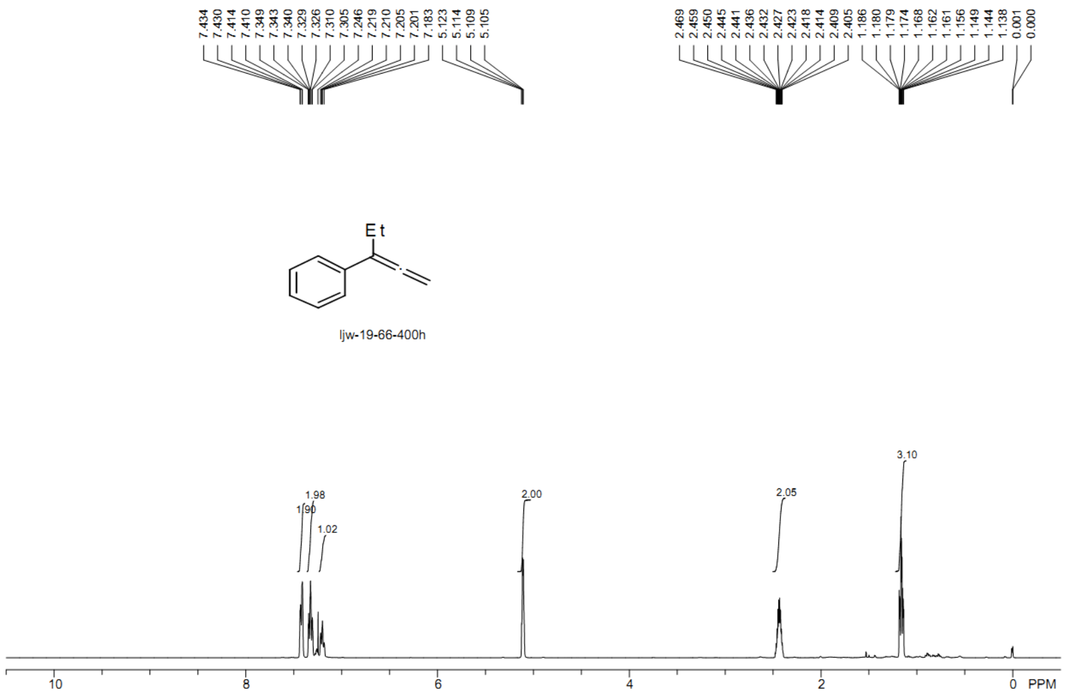

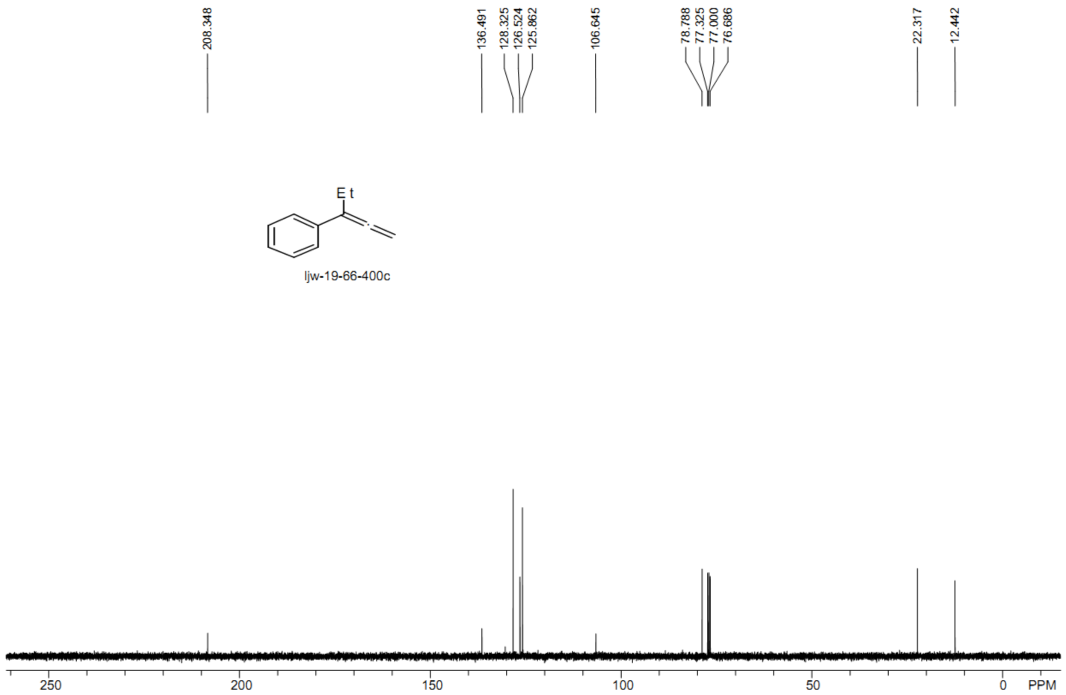

NMR spectra of 1z

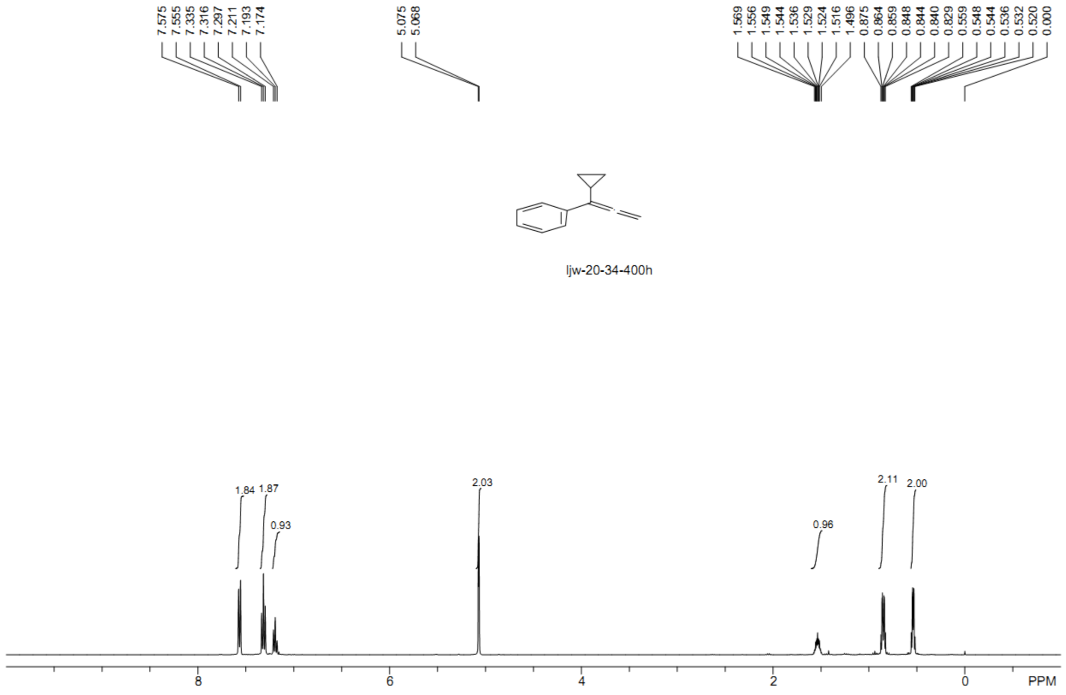

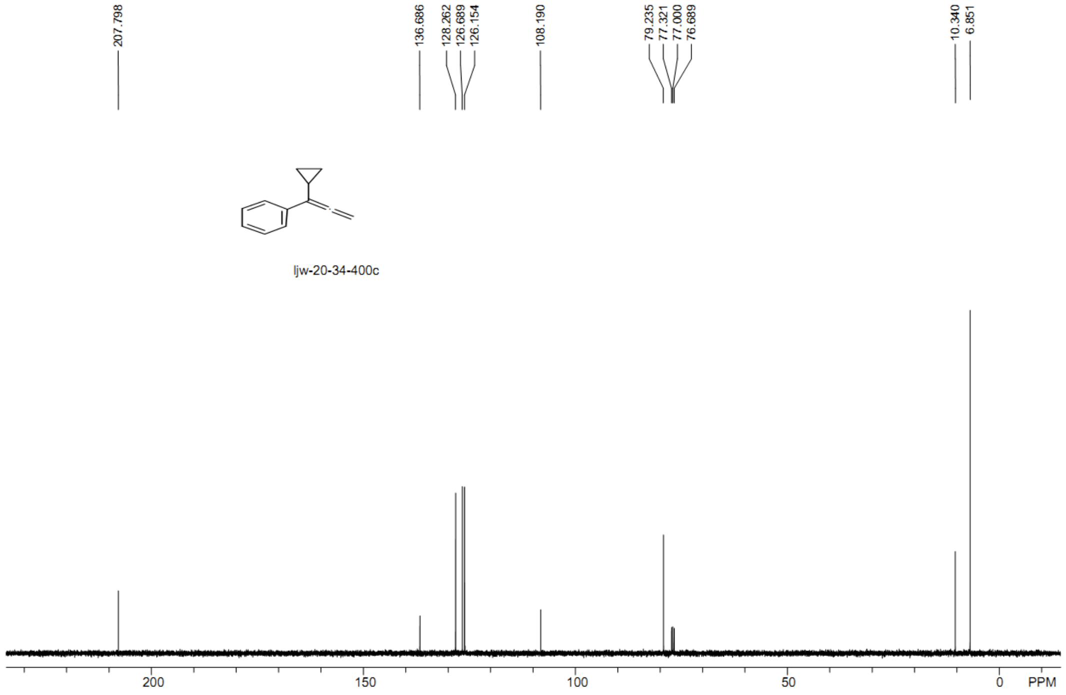

NMR spectra of 1aa

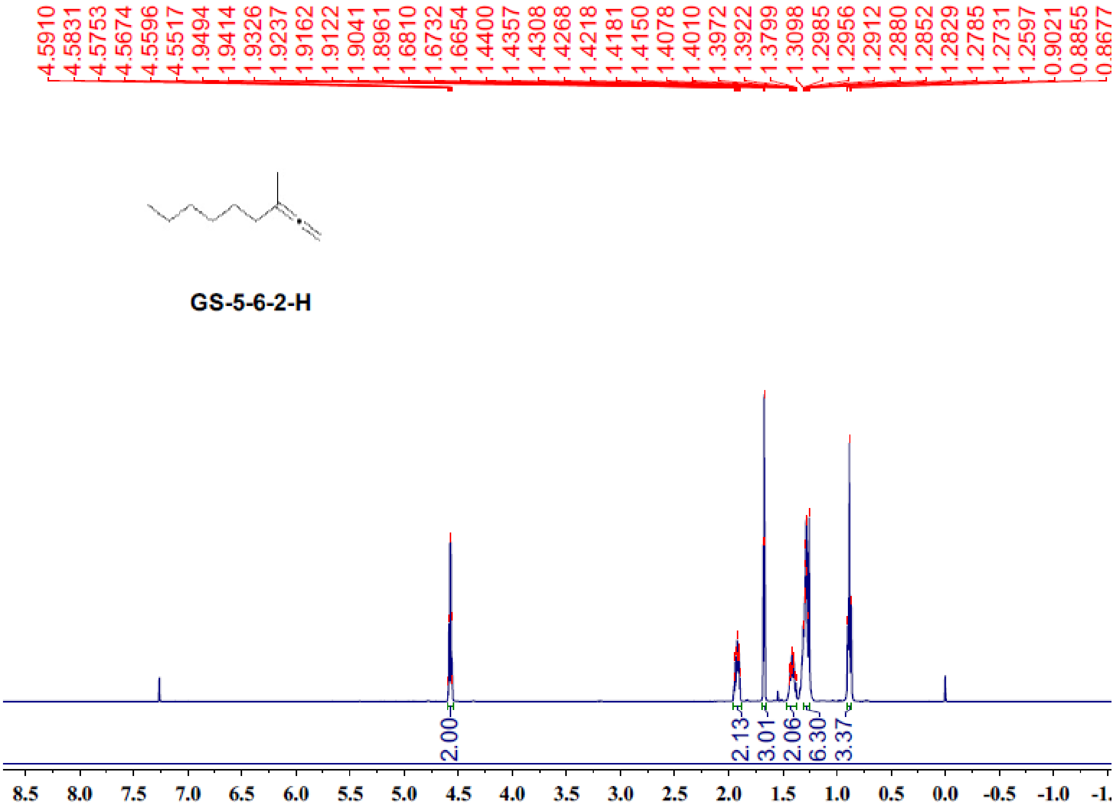

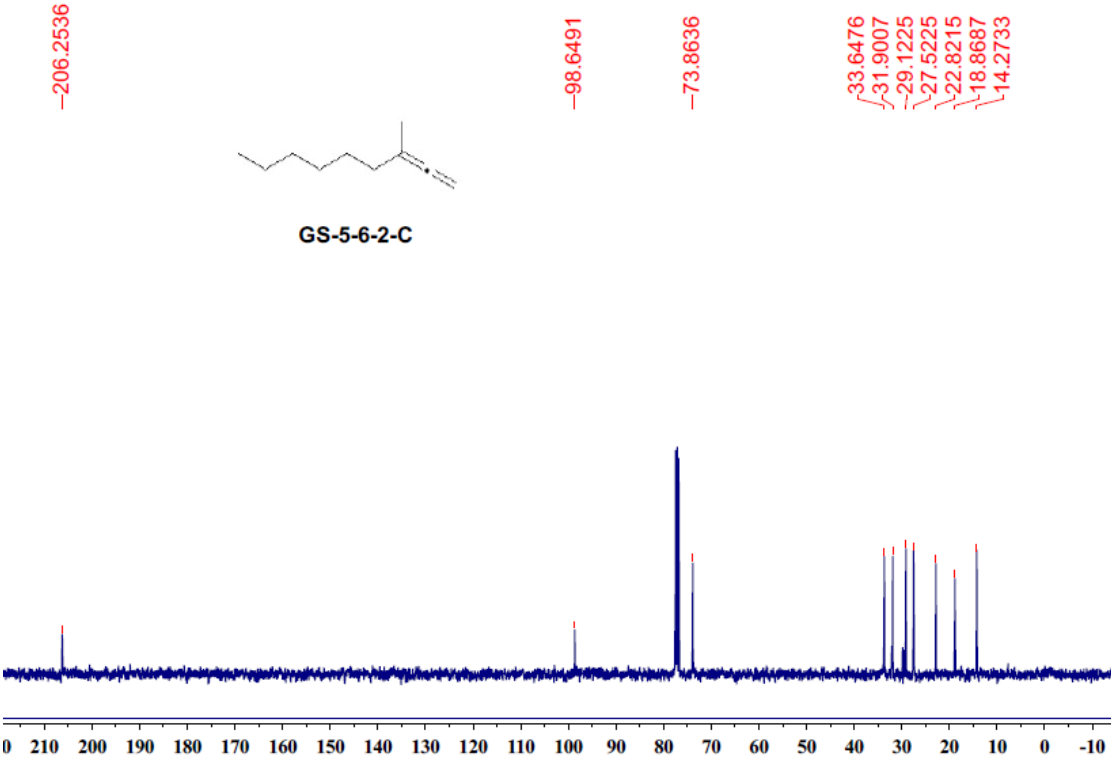

NMR spectra of 1ab

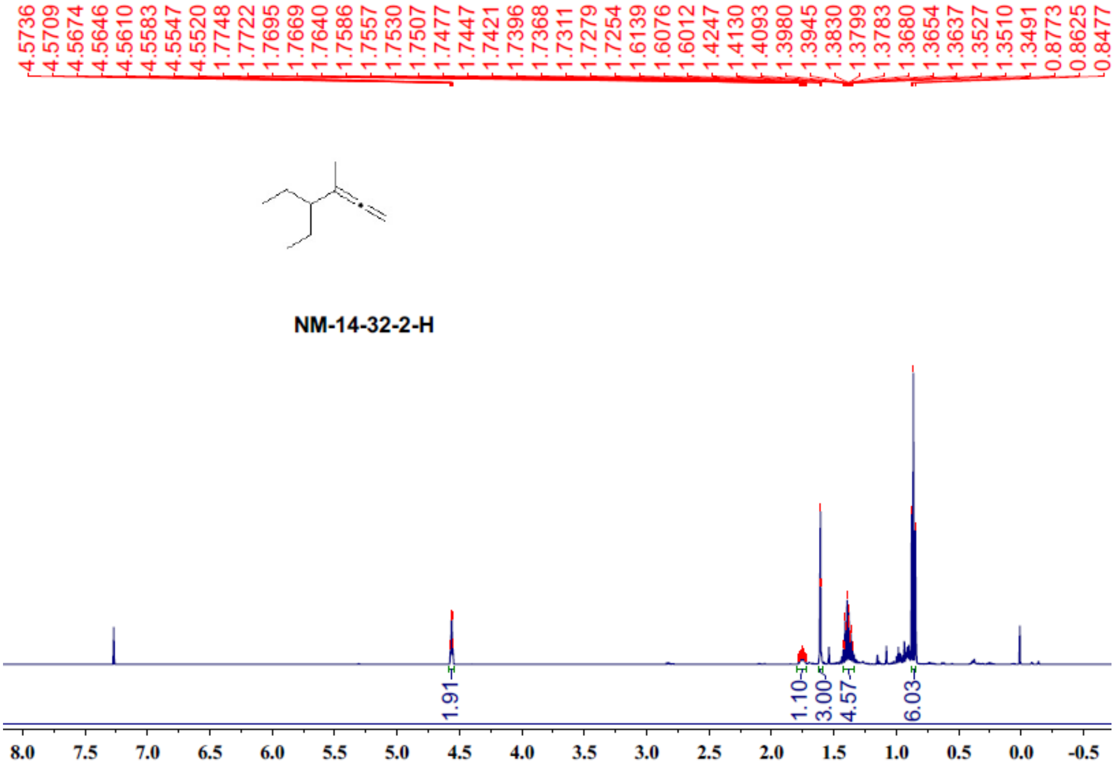

NMR spectra of 1ac

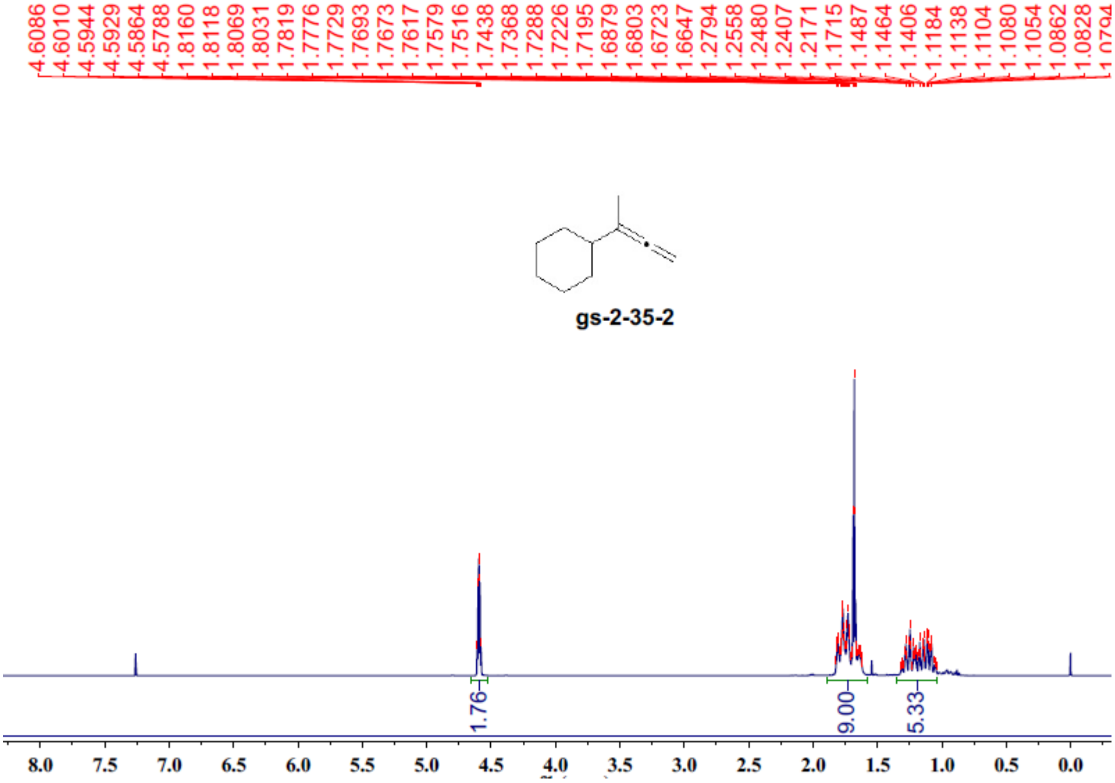

NMR spectra of 1ad

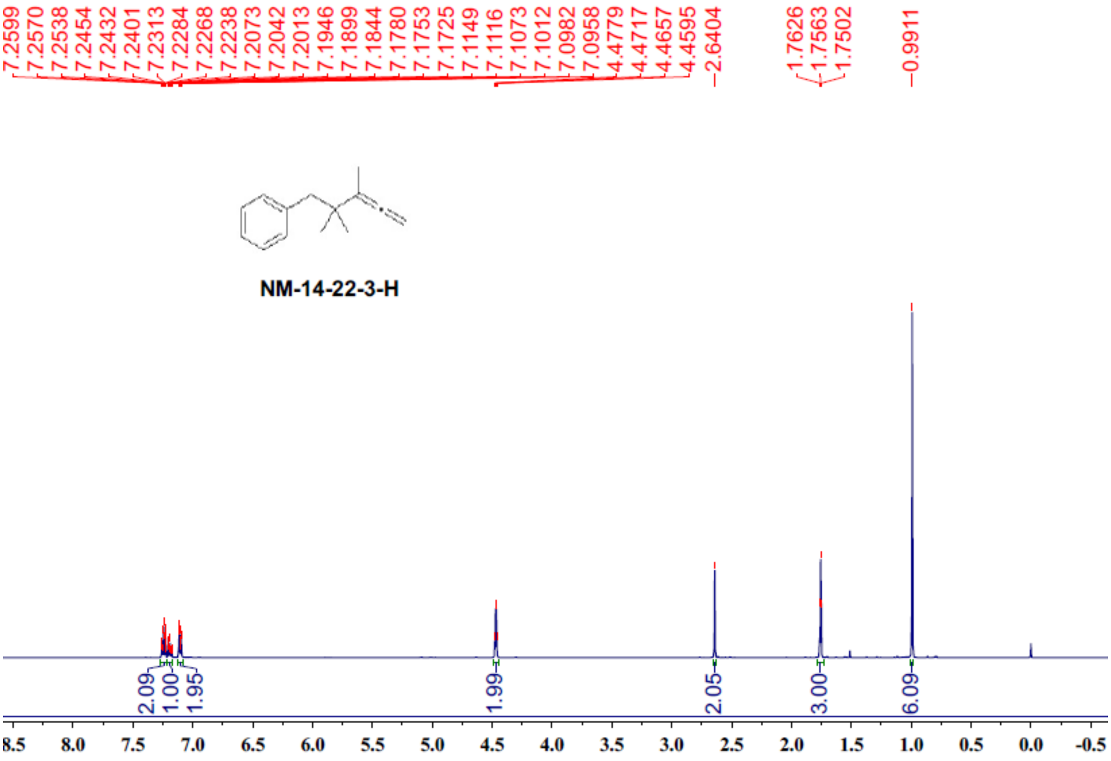

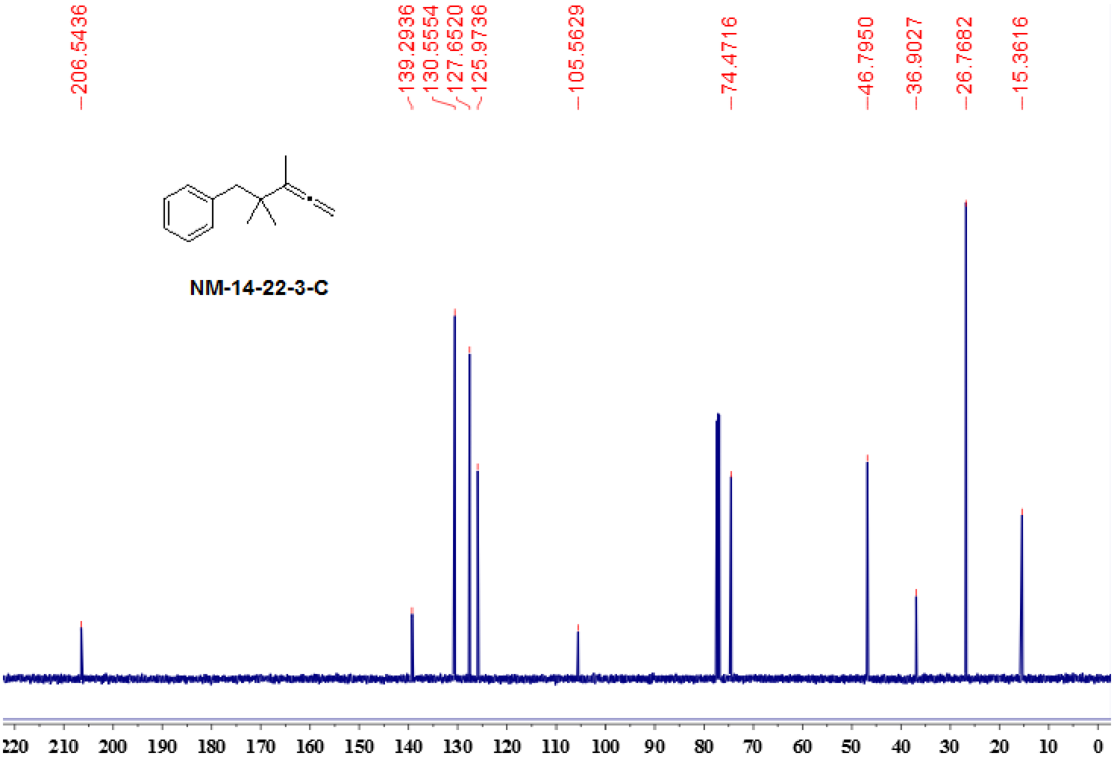

NMR spectra of 1ae

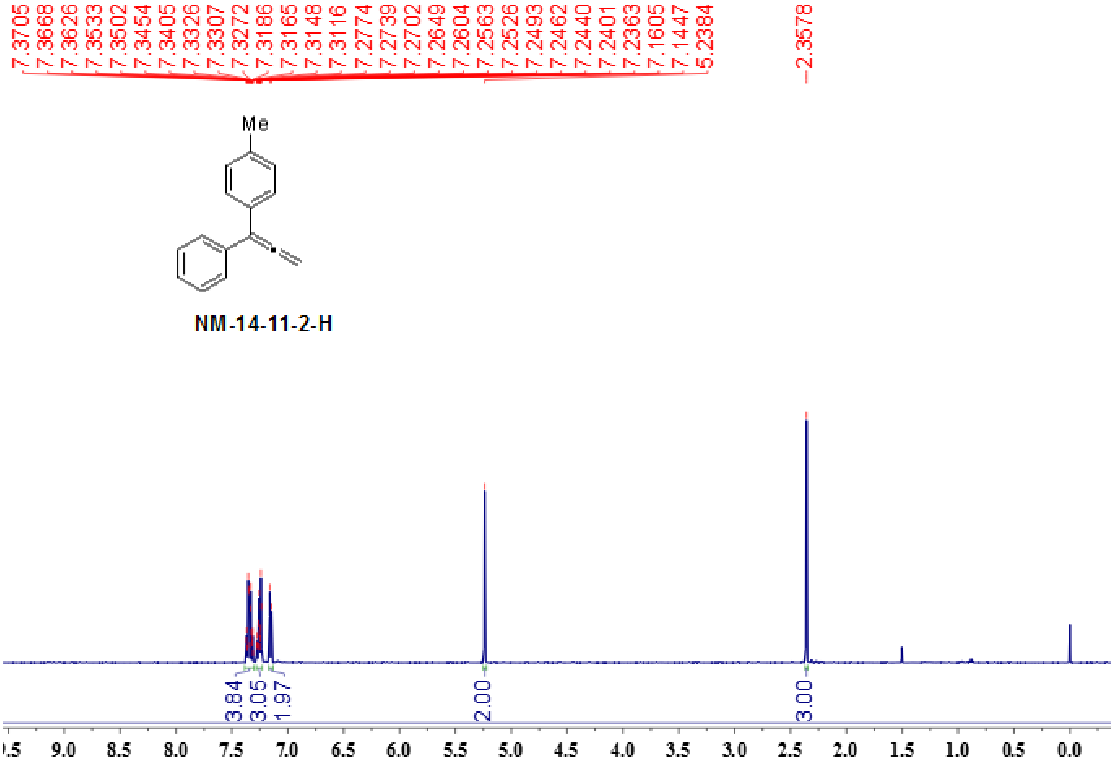

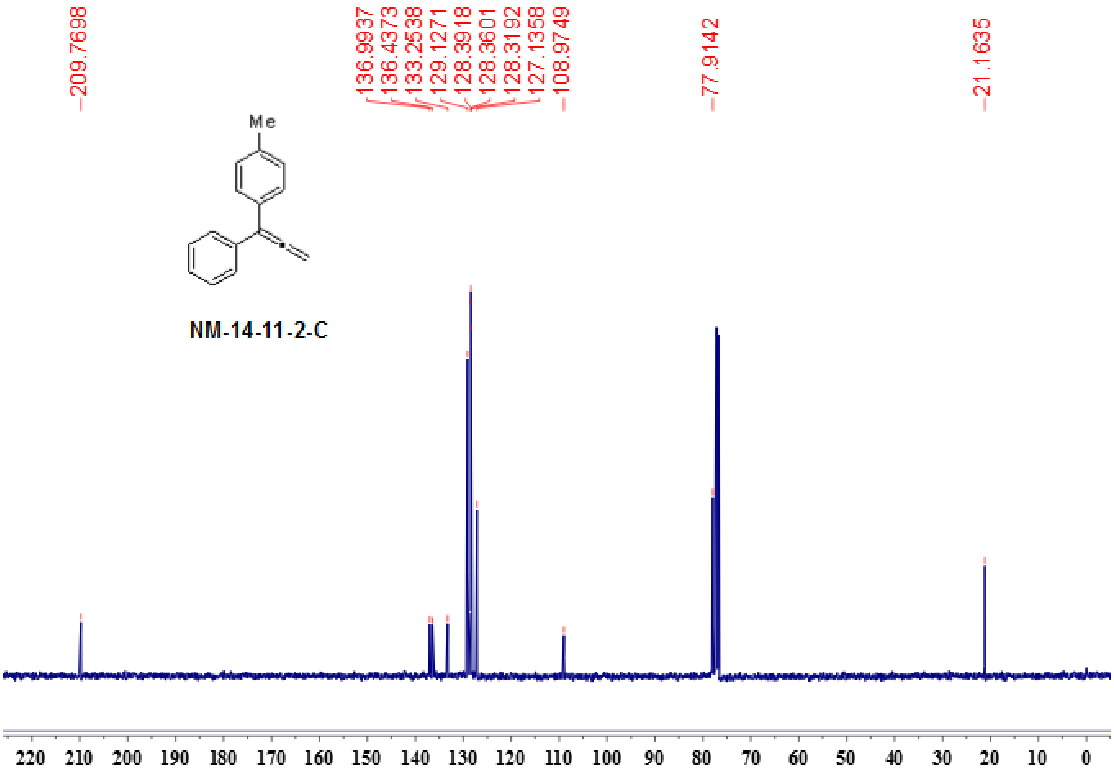

NMR spectra of 1af

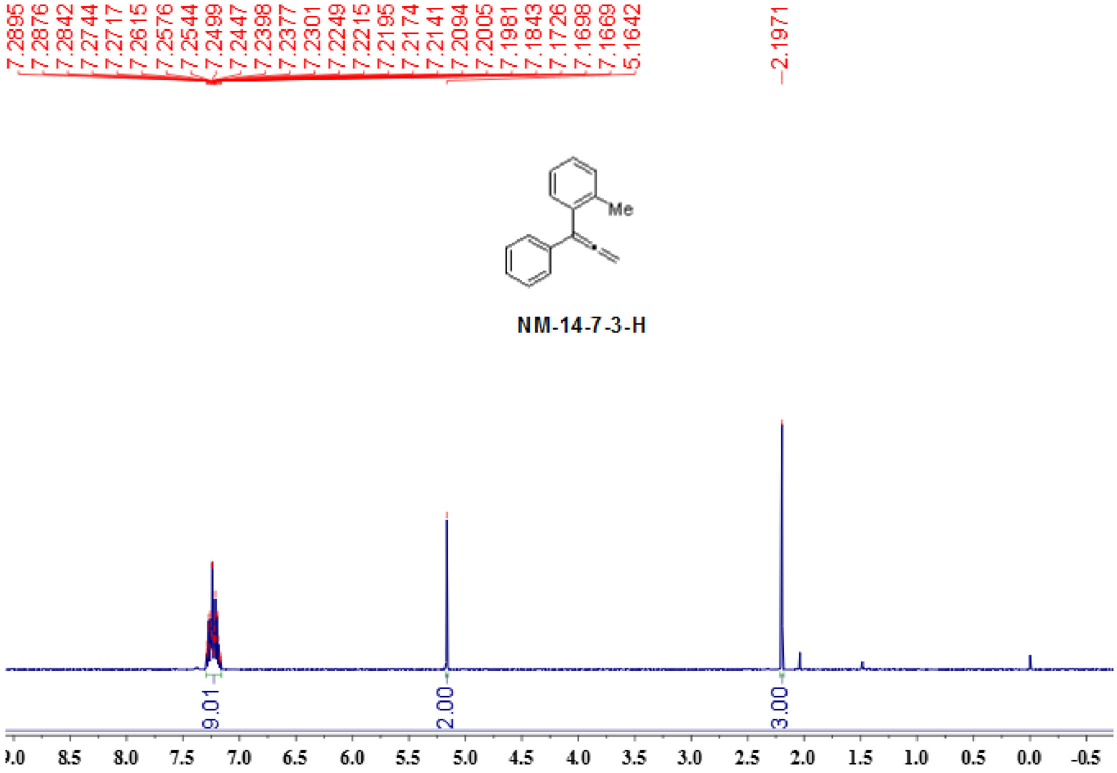

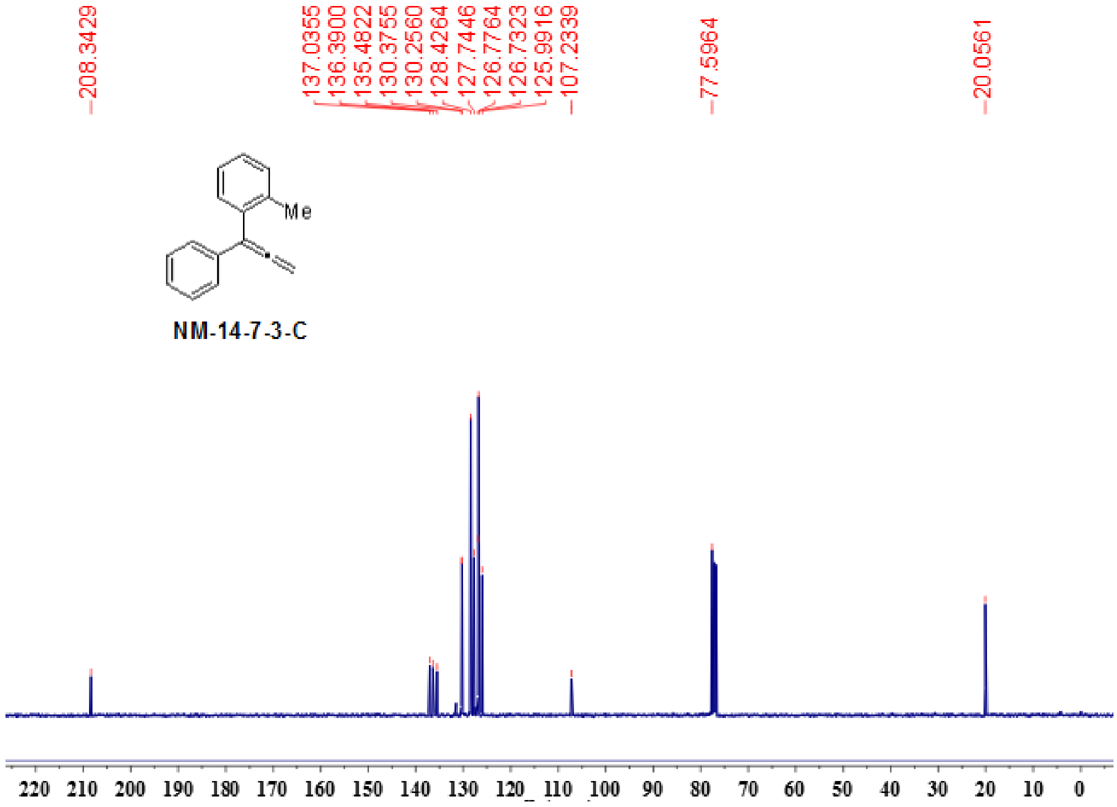

NMR spectra of 3a

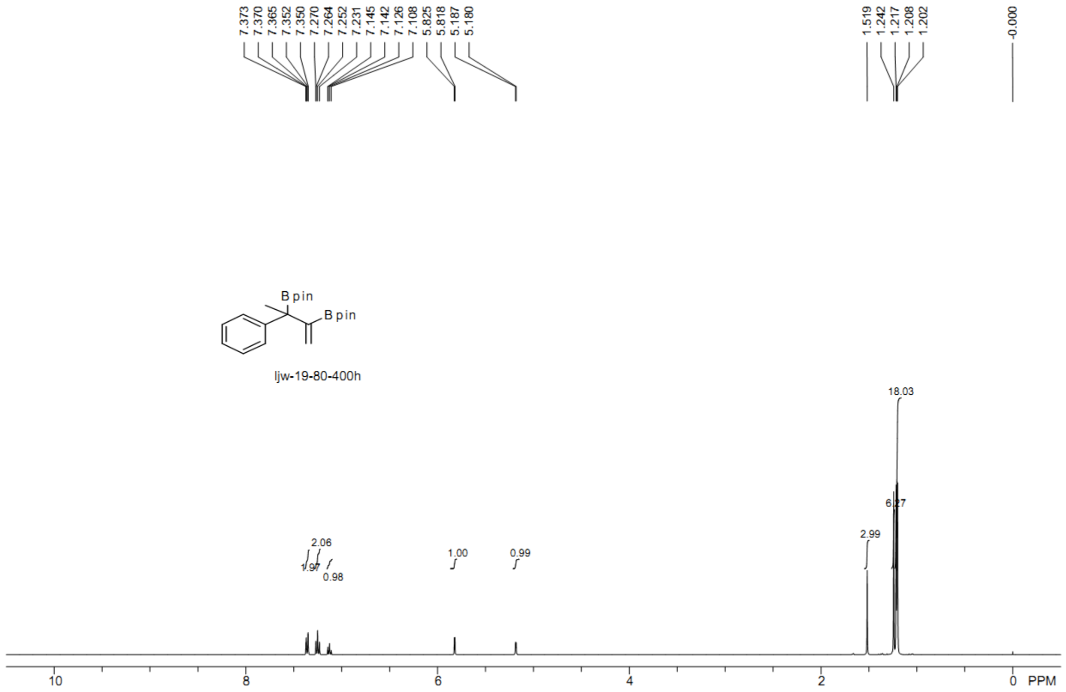

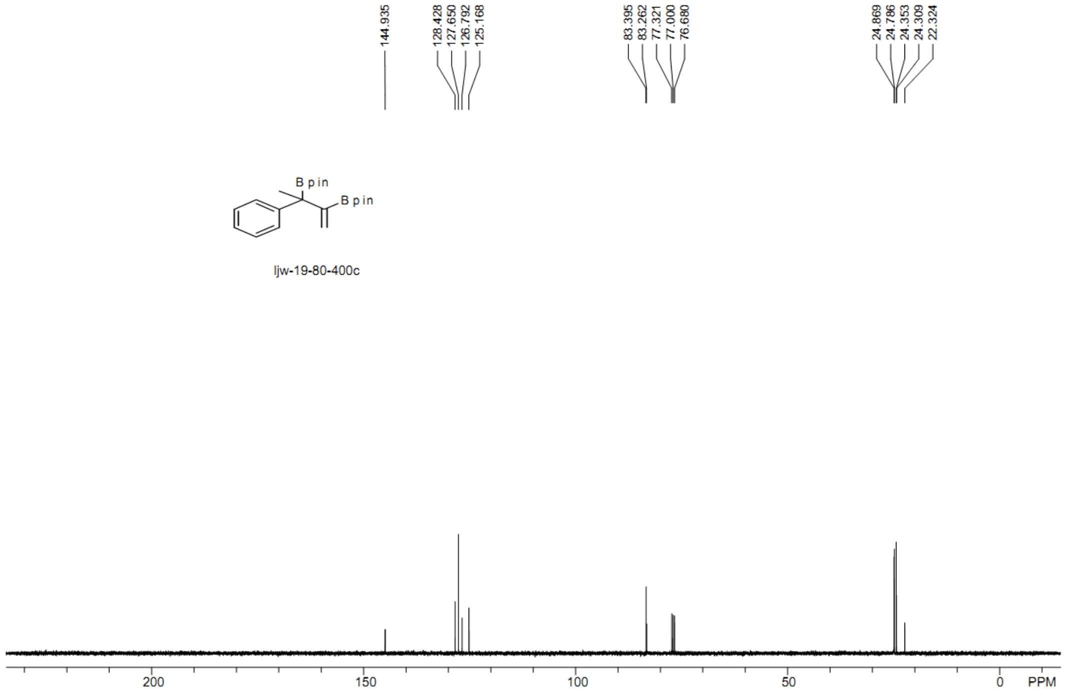

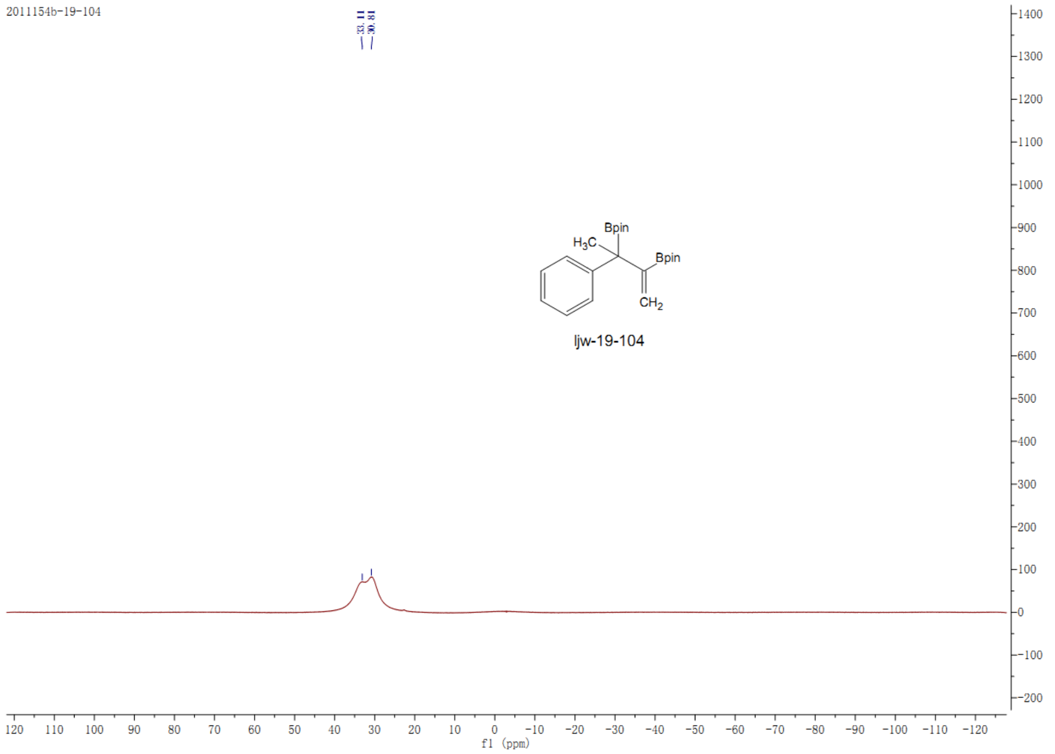

NMR spectra of 3b

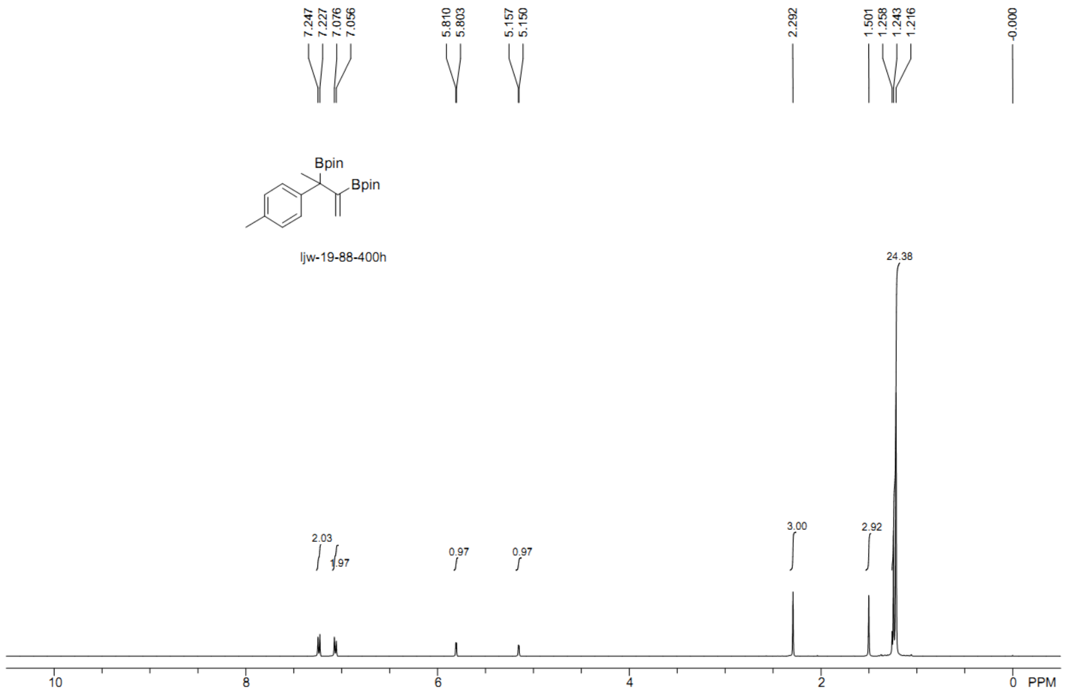

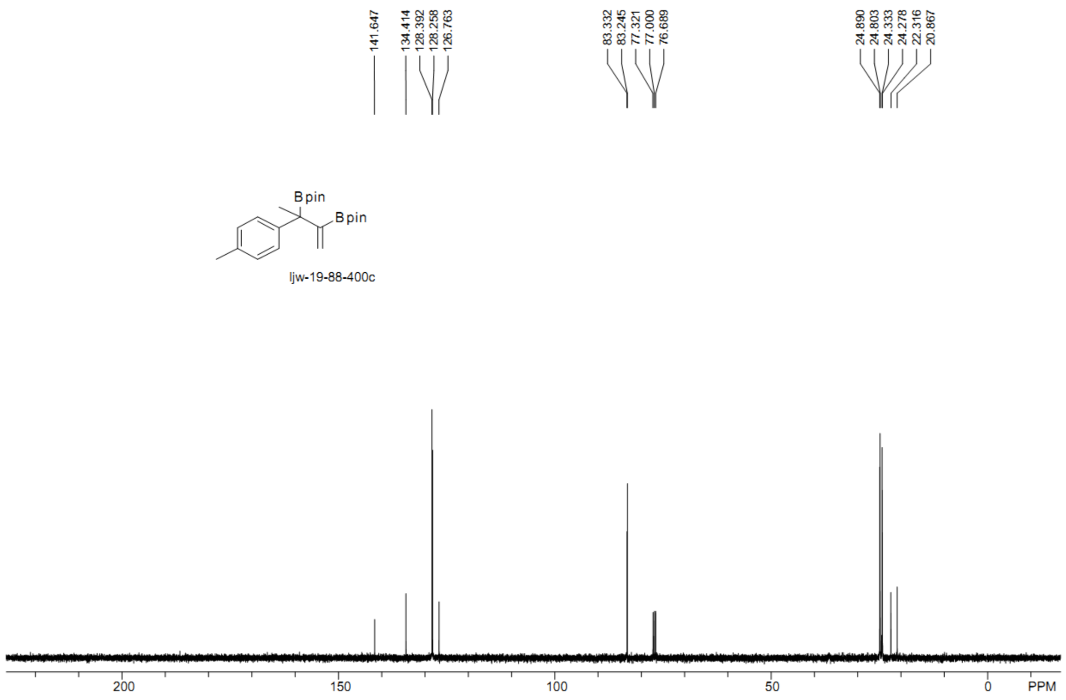
141.647
134.414
128.392
128.258
126.763
83.332
83.245
77.321
77.000
76.689
24.890
24.803
24.333
24.278
22.316
20.867
Bpin
Bpin
ljw-19-88-400c
200
150
100
50
0
PPM

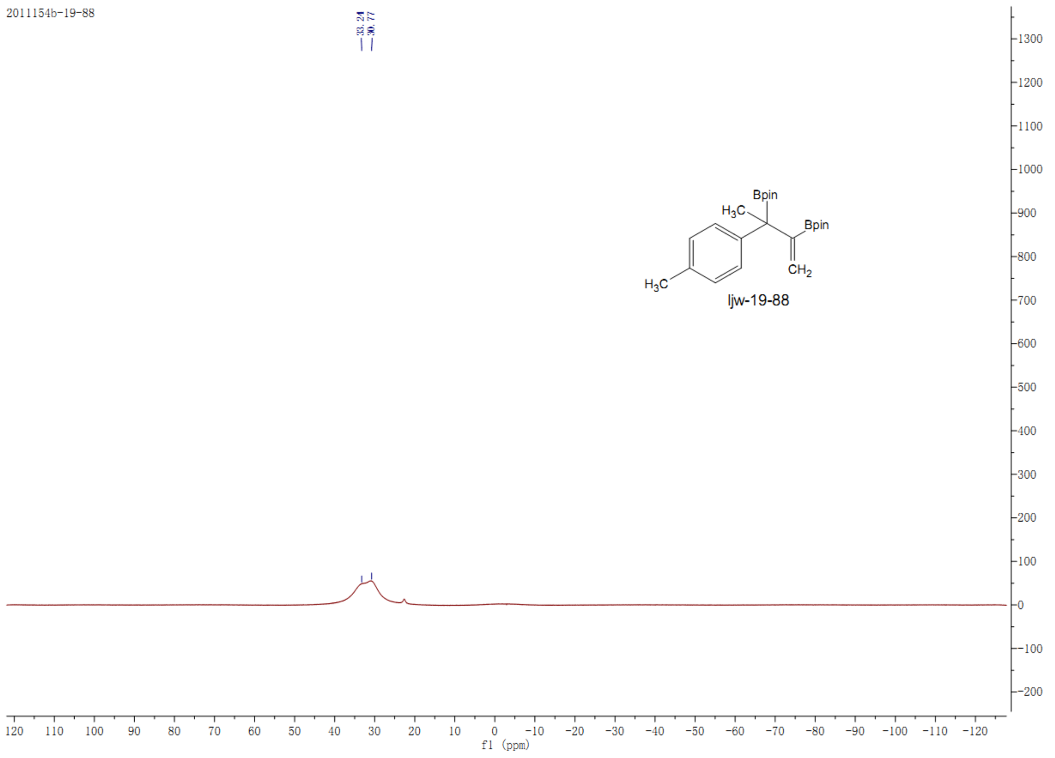
2011154b-19-88
33.24
30.77
Bpin
H_3C
Bpin
CH_2
H_3C
ljw-19-88
f1 (ppm)

NMR spectra of 3c

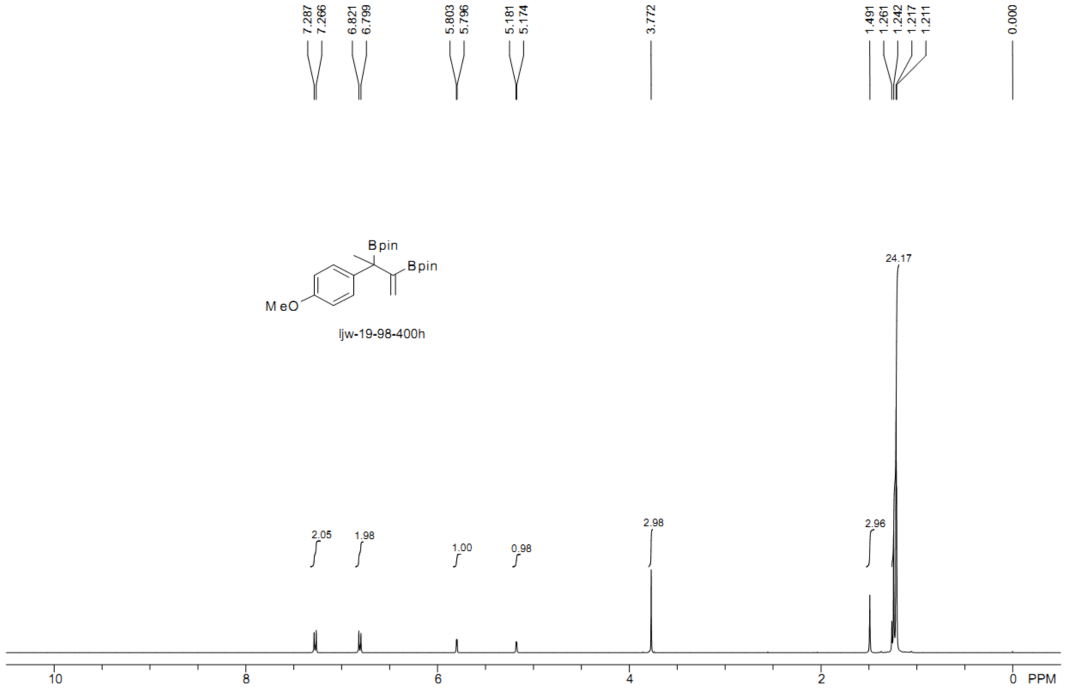

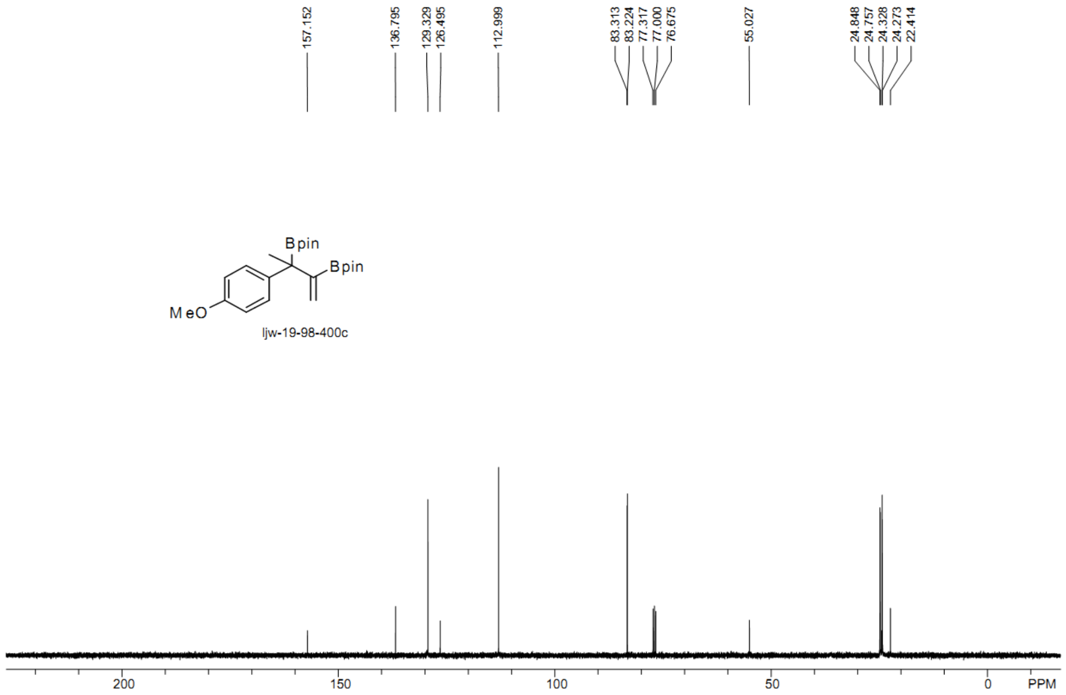

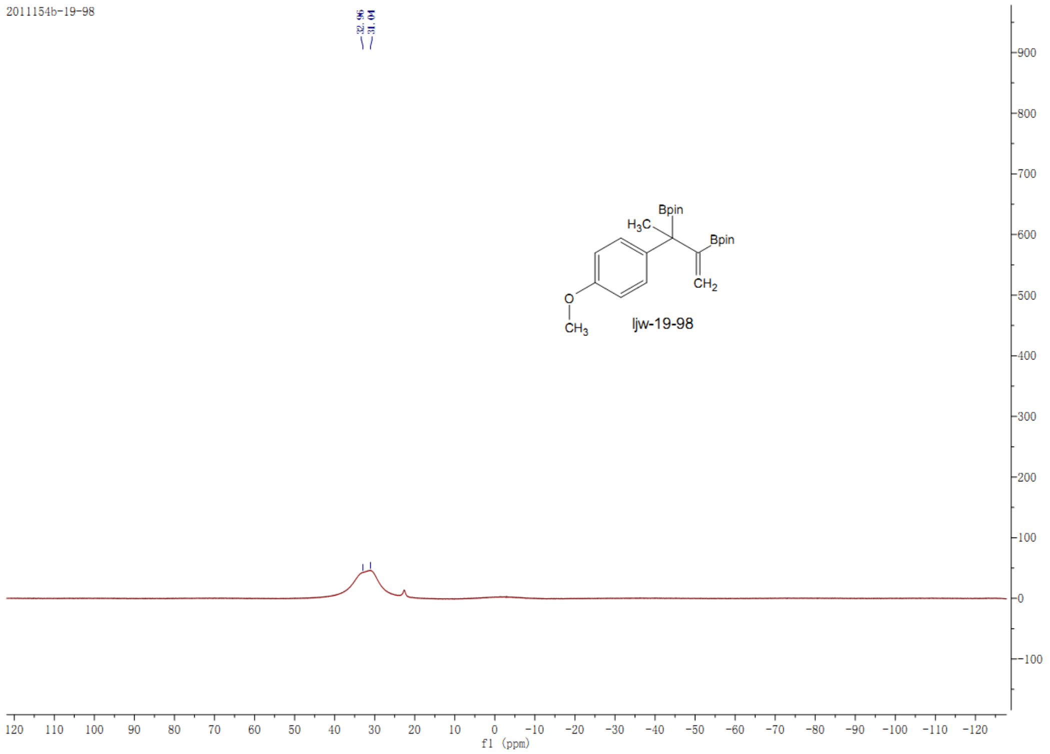

NMR spectra of 3d

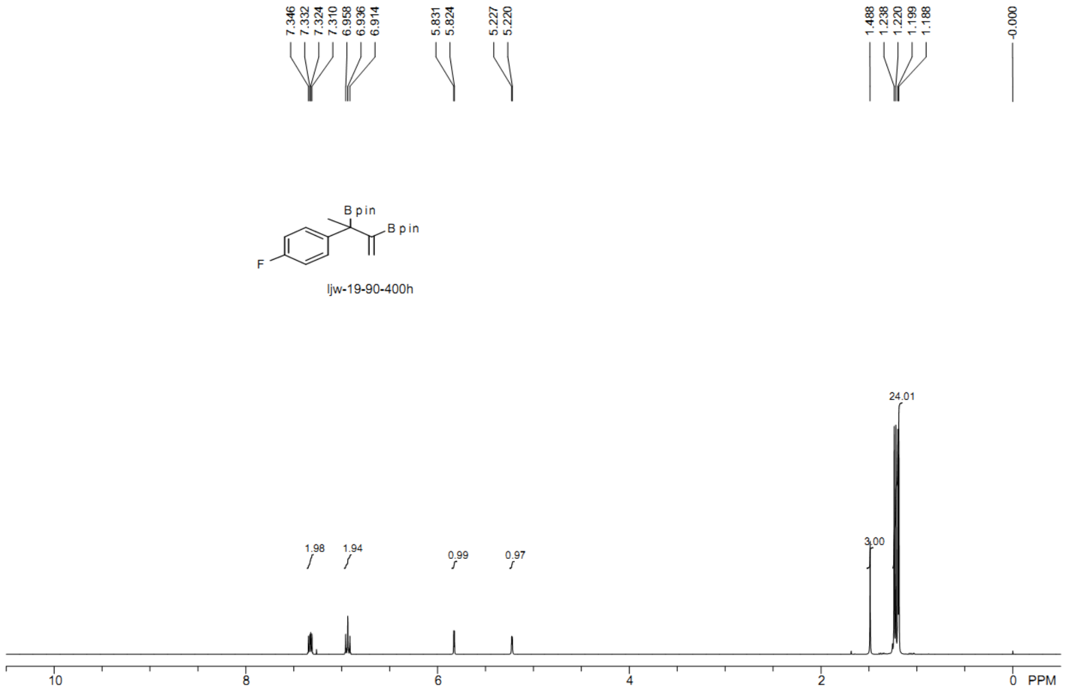

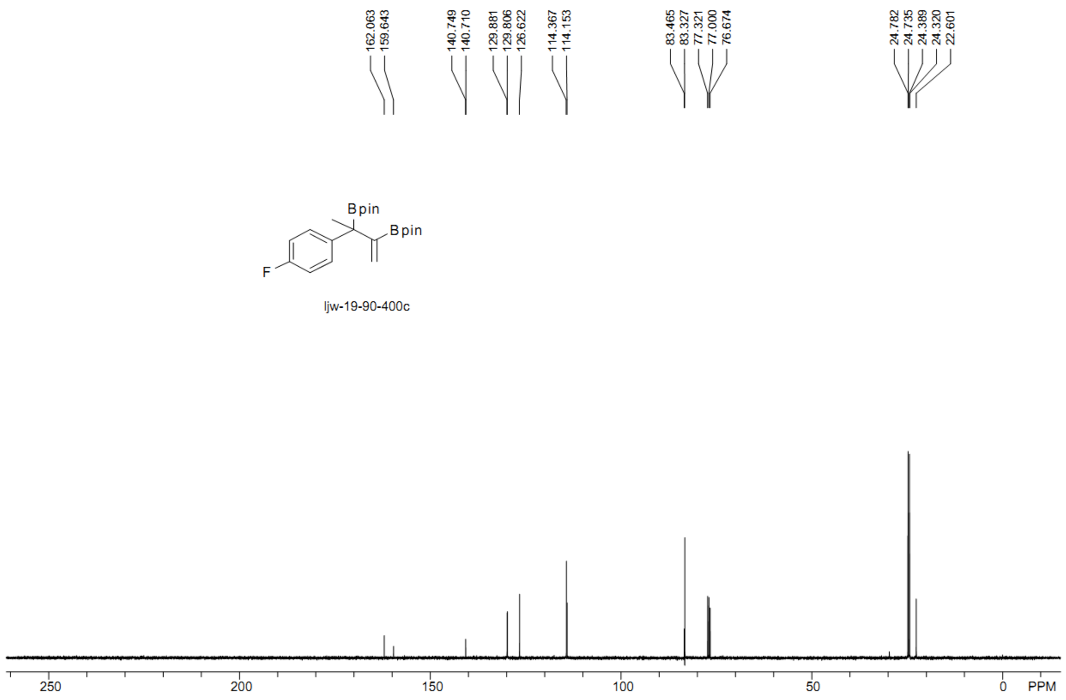
162.063
159.643
140.749
140.710
129.881
129.806
126.622
114.367
114.153
83.465
83.327
77.321
77.000
76.674
24.782
24.735
24.389
24.320
22.601
Bpin
Bpin
F
ljw-19-90-400c
250
200
150
100
50
0
PPM

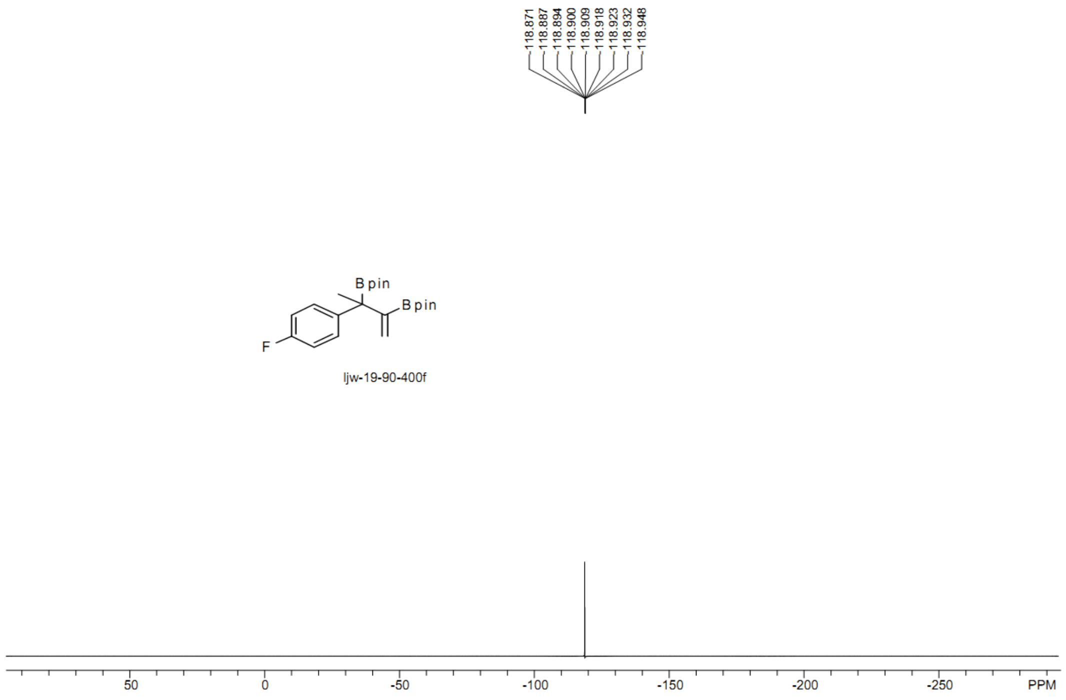
118.871
118.887
118.894
118.900
118.909
118.918
118.923
118.932
118.948
Bpin
Bpin
F
ljw-19-90-400f
50
0
-50
-100
-150
-200
-250
PPM

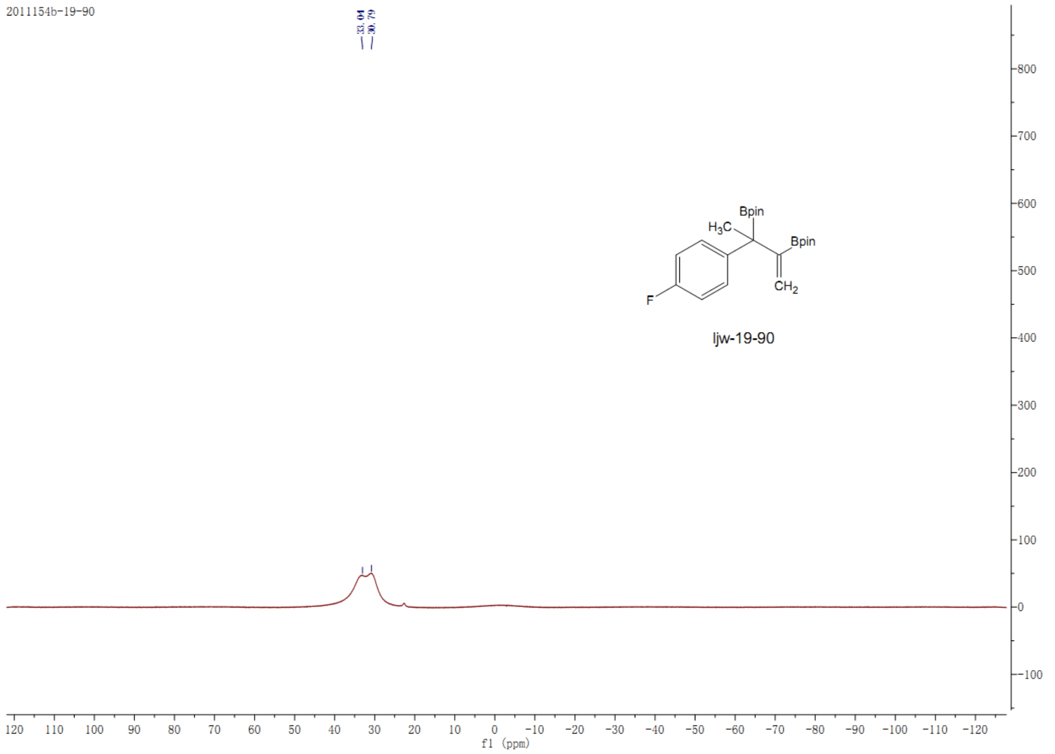

NMR spectra of 3e

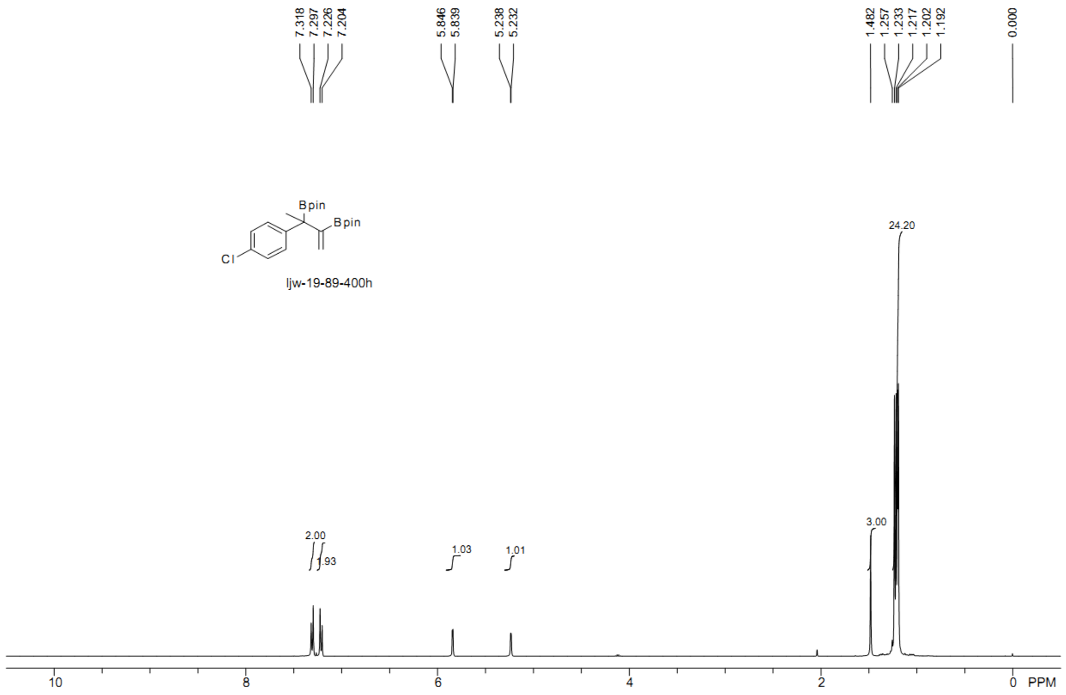

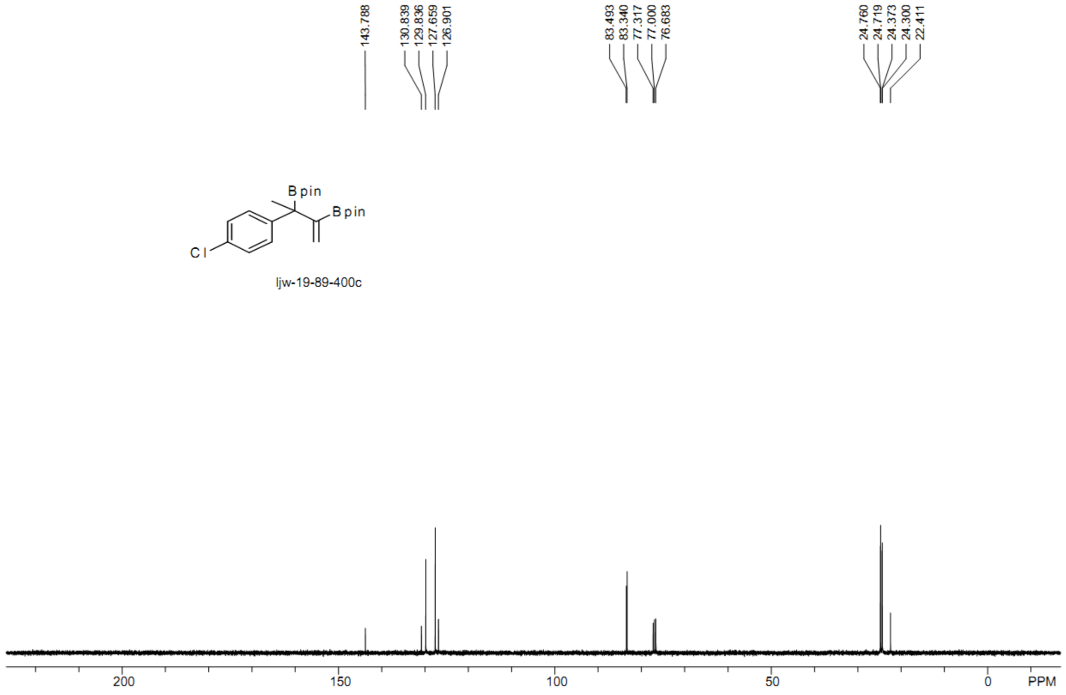

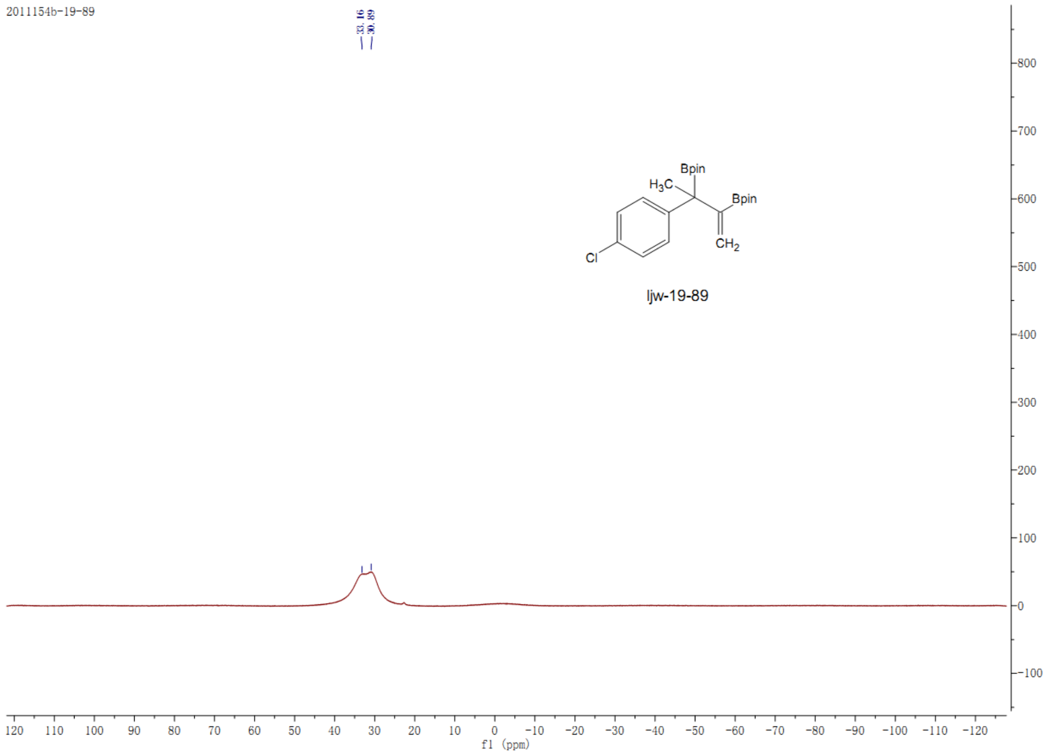

NMR spectra of 3f

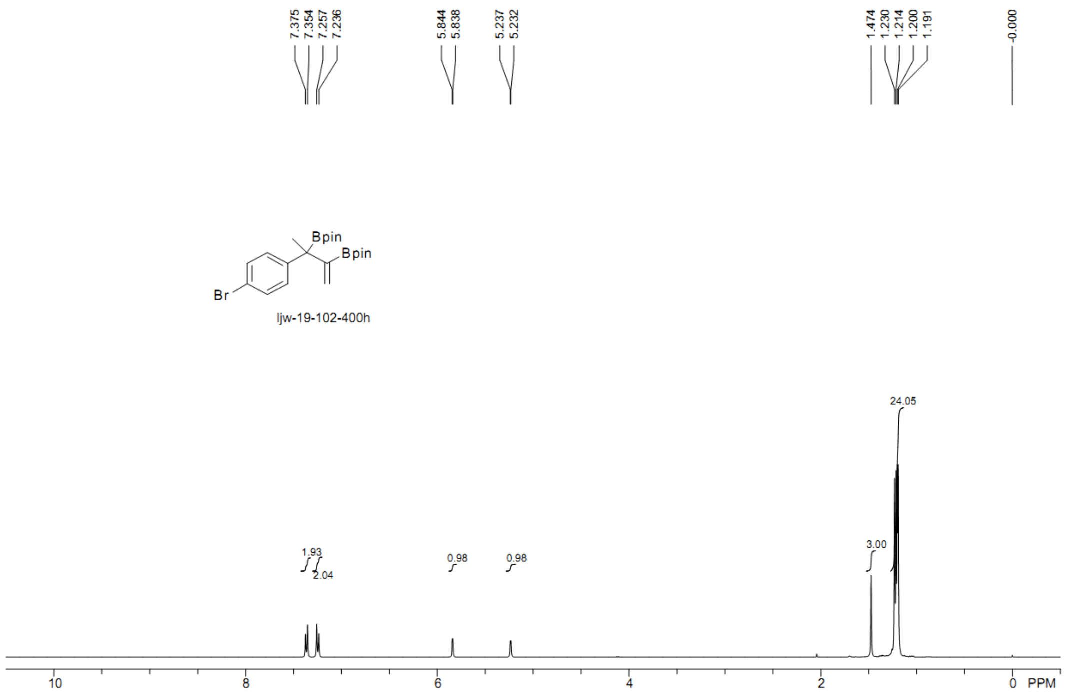

7.375
7.354
7.257
7.236
5.844
5.838
5.237
5.232
1.474
1.230
1.214
1.200
1.191
-0.000
Bpin
Bpin
Br
ljw-19-102-400h
1.93
2.04
0.98
0.98
3.00
24.05
10
8
6
4
2
0
PPM

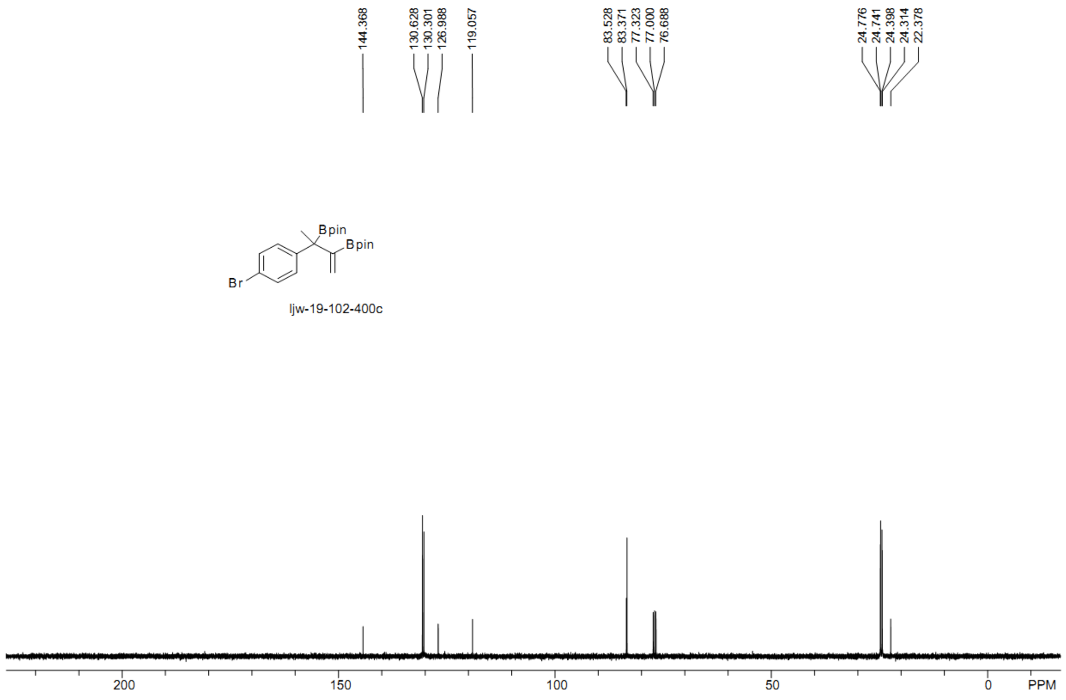

144.368
130.628
130.301
126.988
119.057
83.528
83.371
77.323
77.000
76.688
24.776
24.741
24.398
24.314
22.378
Bpin
Bpin
Br
ljw-19-102-400c
200
150
100
50
0
PPM

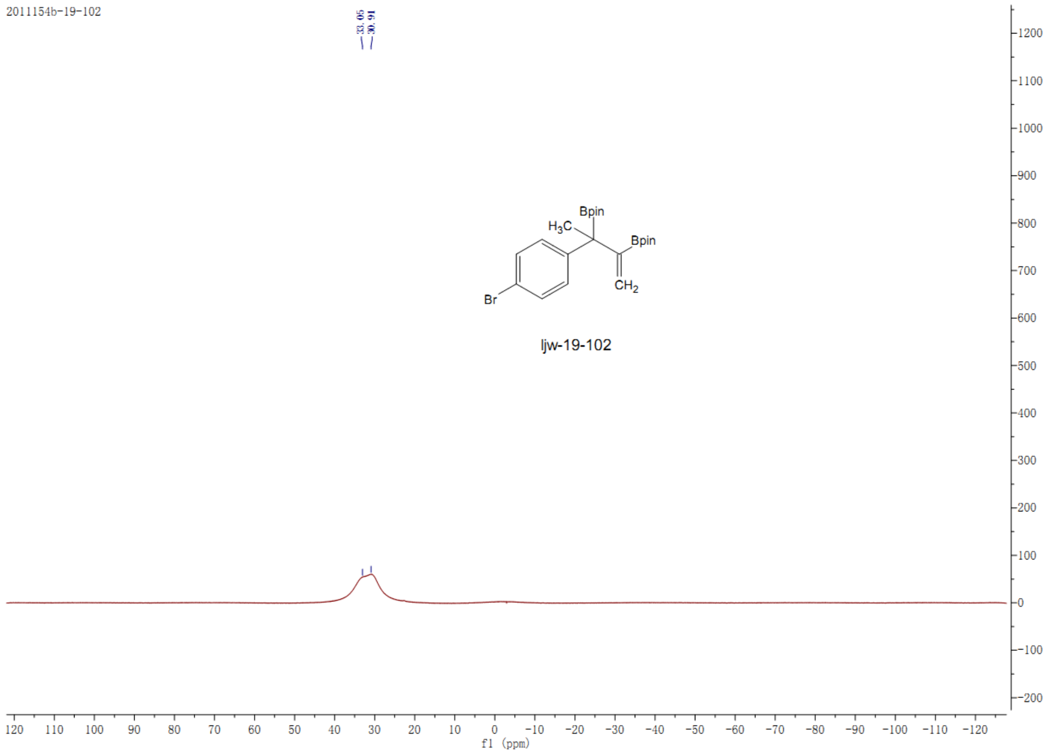

NMR spectra of 3g

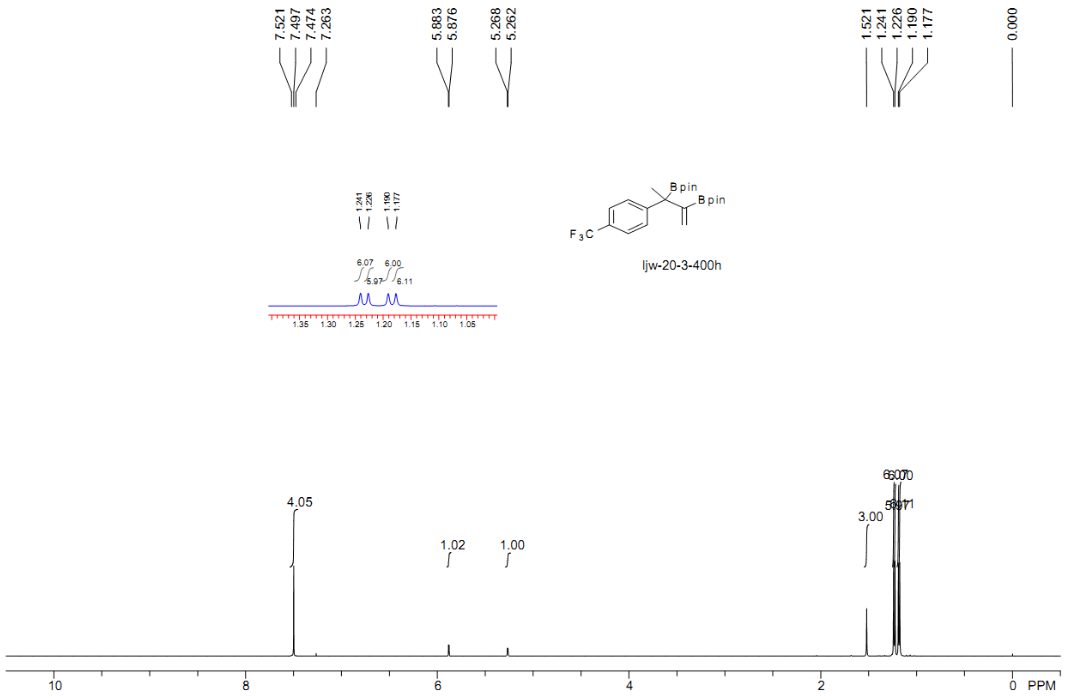

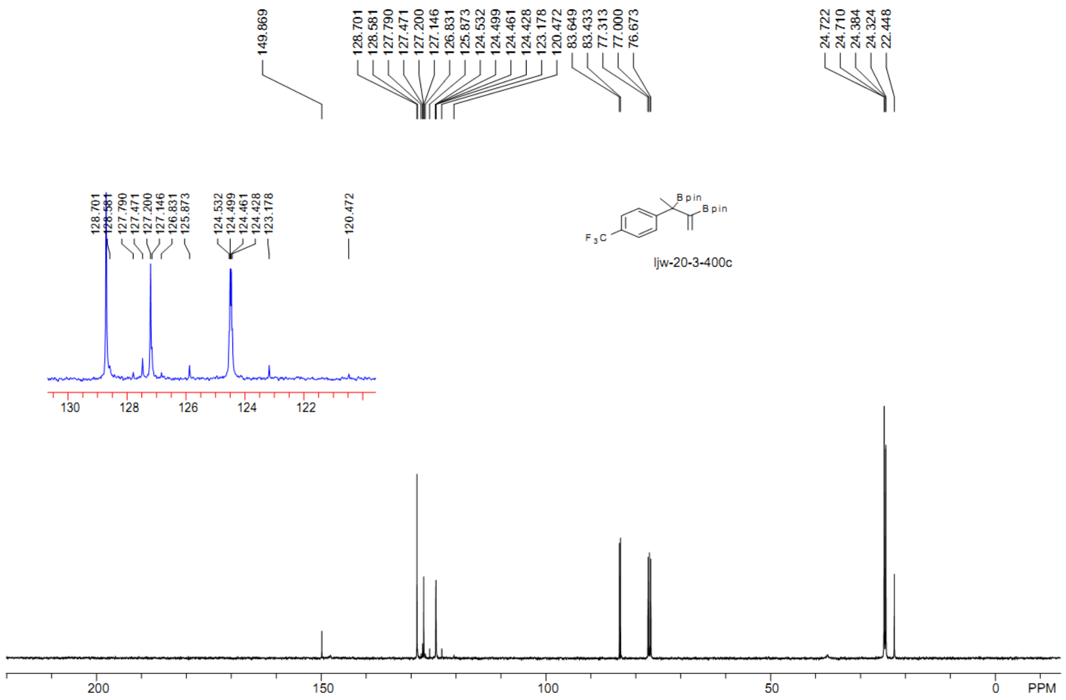
149.869
128.701
128.581
127.790
127.471
127.200
127.146
126.831
125.873
124.532
124.499
124.461
124.428
123.178
120.472
83.649
83.433
77.313
77.000
76.673
24.722
24.710
24.384
24.324
22.448
Bpin
Bpin
F3C
ljw-20-3-400c
130
128
126
124
122
200
150
100
50
0
PPM

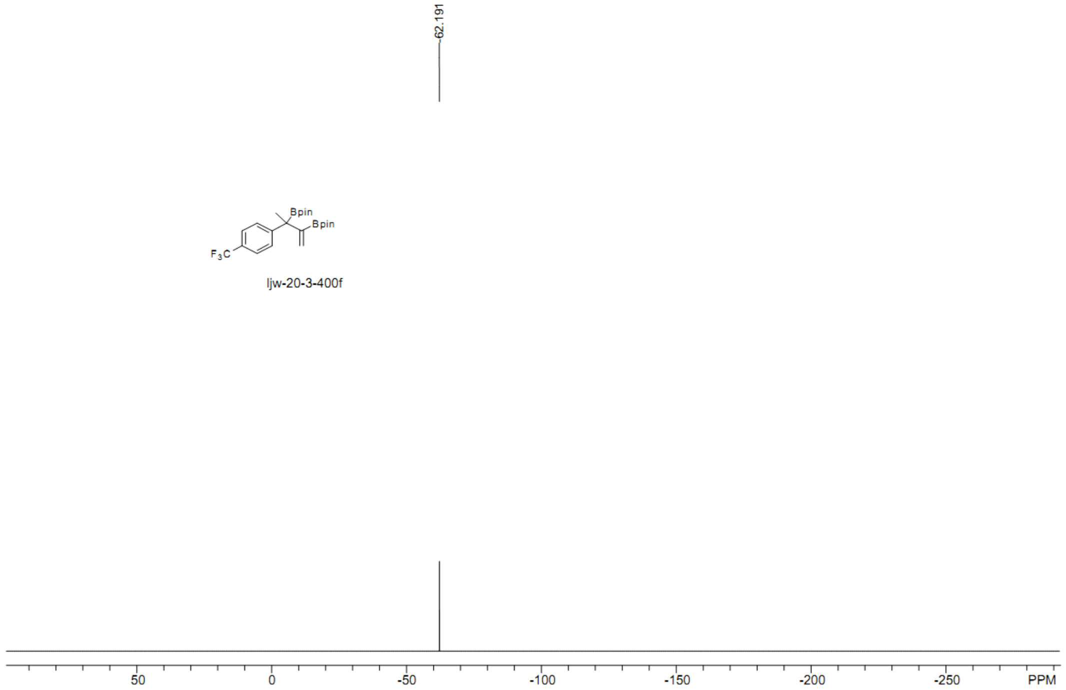
-62.191
Bpin
Bpin
F3C
ljw-20-3-400f
50
0
-50
-100
-150
-200
-250
PPM

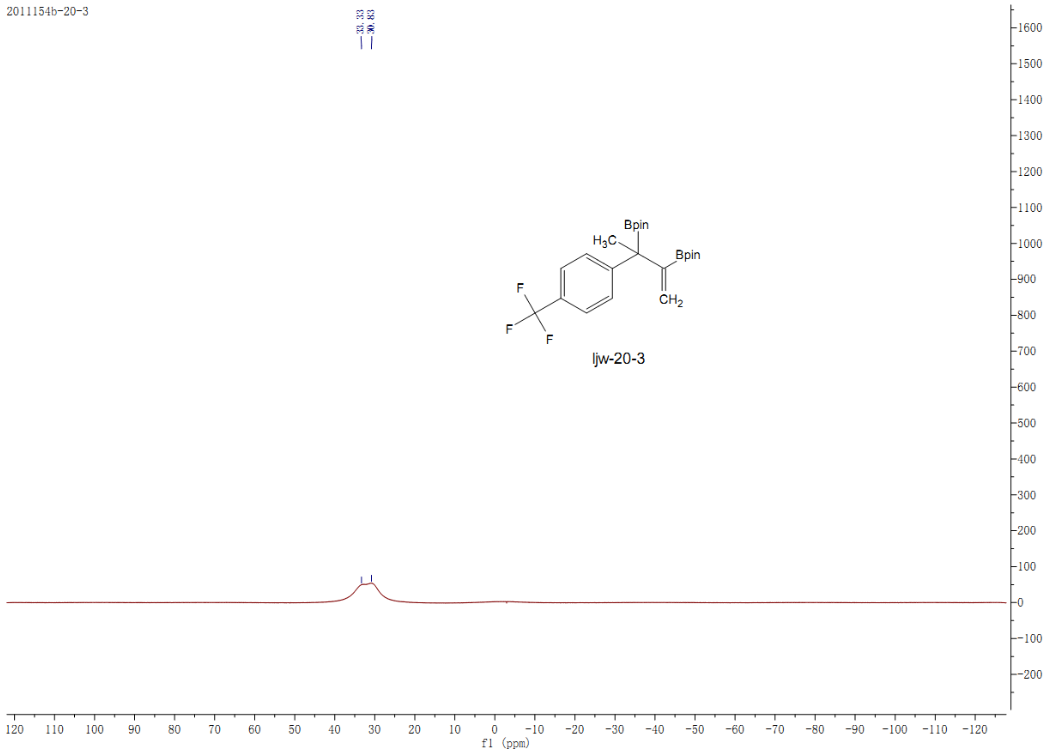

NMR spectra of 3h

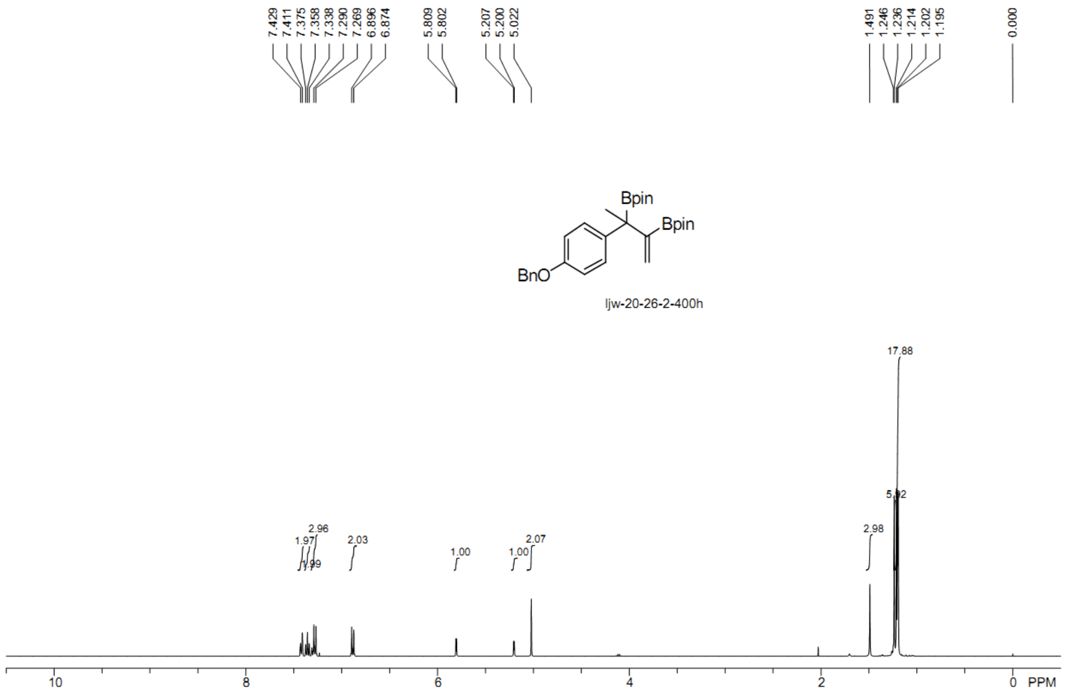

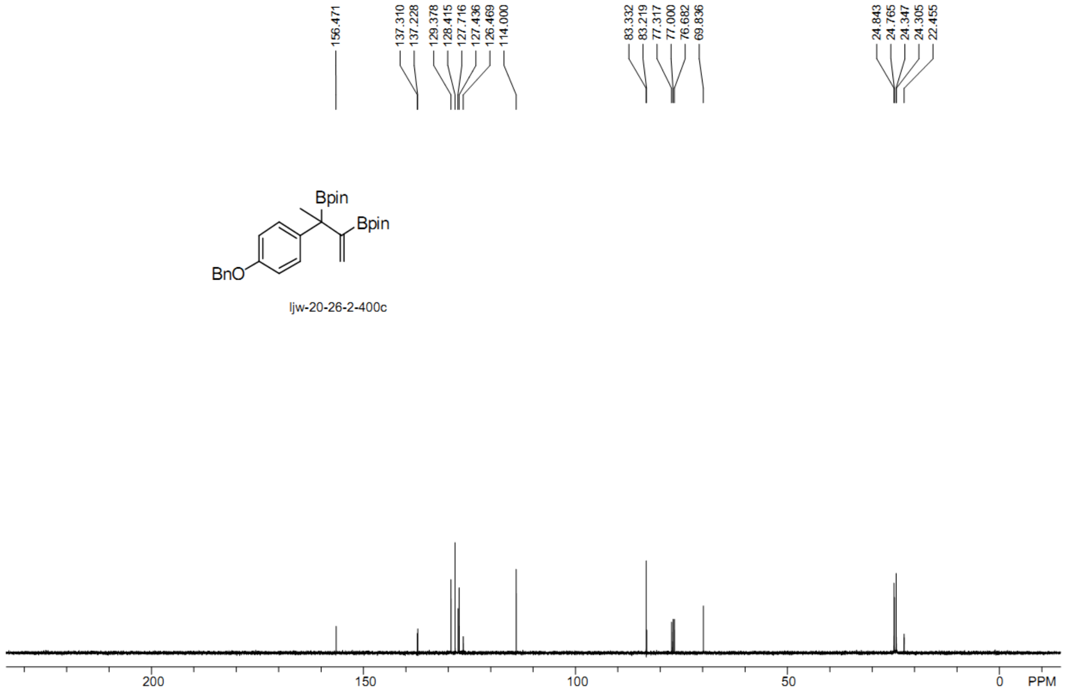
156.471
137.310
137.228
129.378
128.415
127.716
127.436
126.469
114.000
83.332
83.219
77.317
77.000
76.682
69.836
24.843
24.765
24.347
24.305
22.455
Bpin
Bpin
BnO
ljw-20-26-2-400c
200
150
100
50
0
PPM

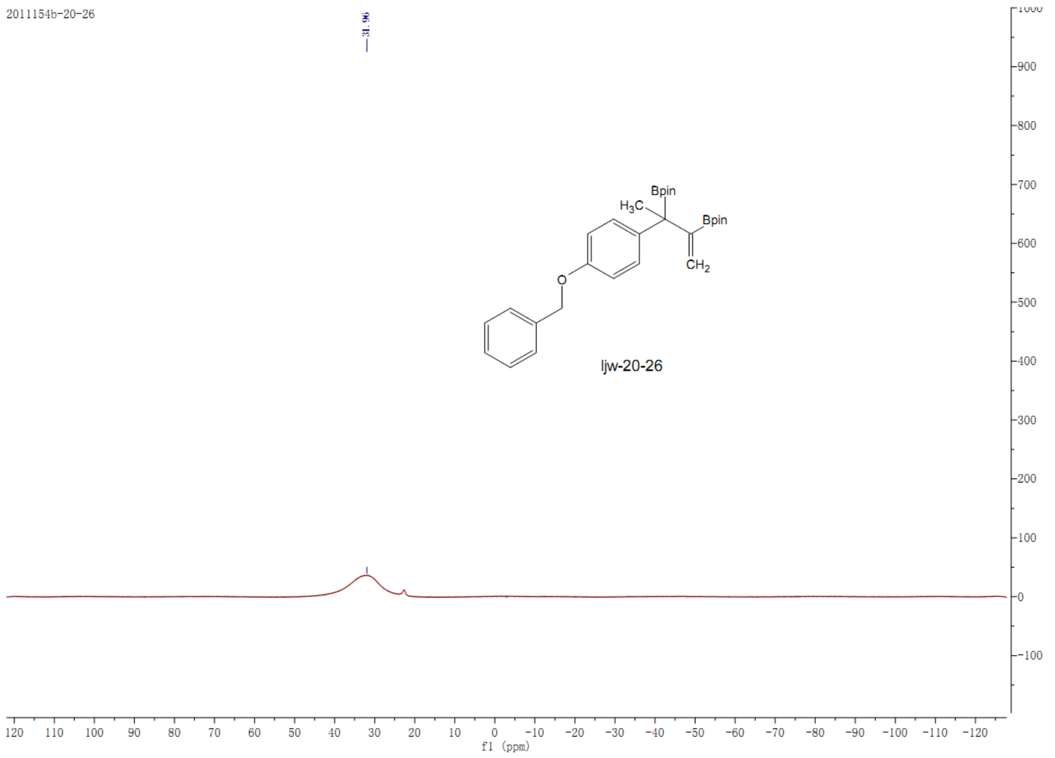
2011154b-20-26
31.96
Bpin
H_3C
Bpin
O
CH_2
ljw-20-26
f1 (ppm)

NMR spectra of 3i

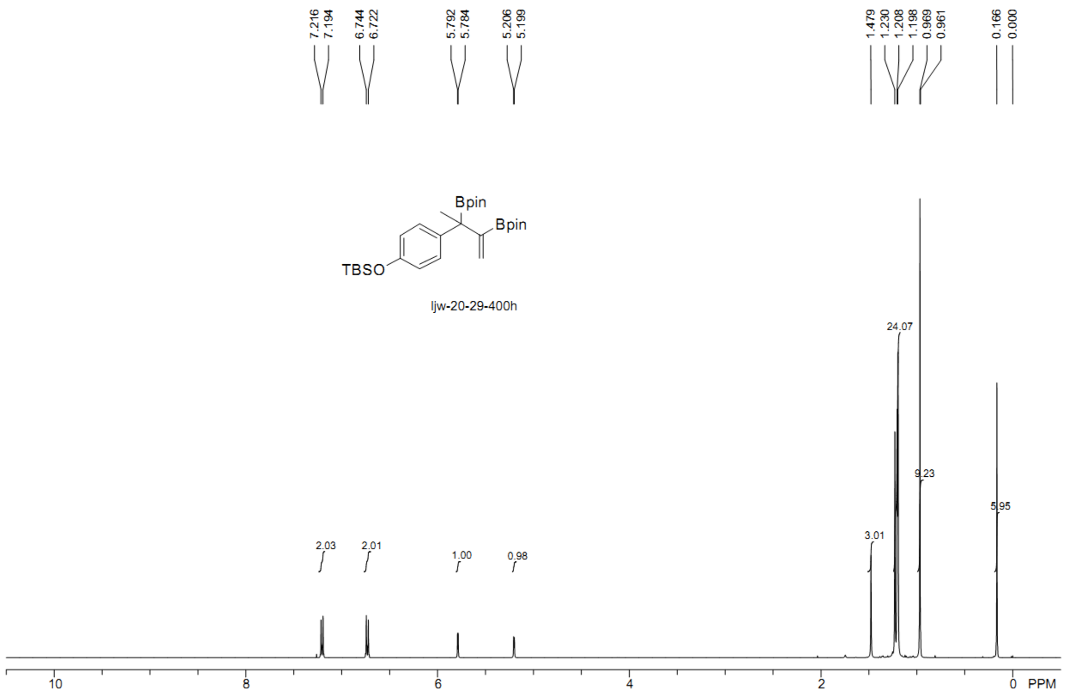

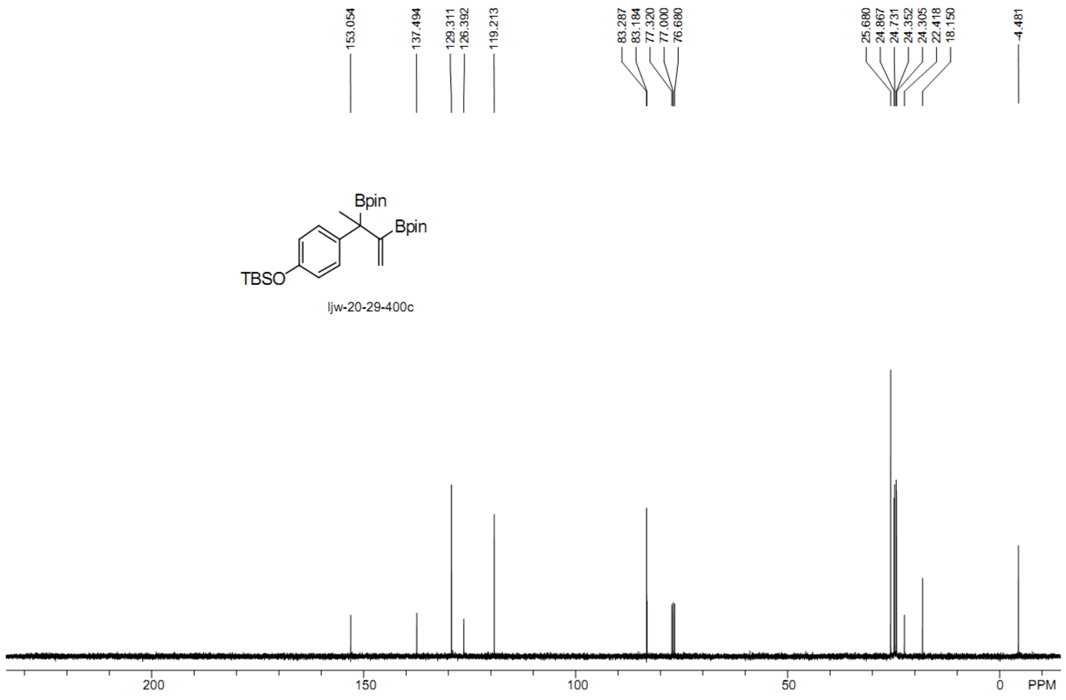

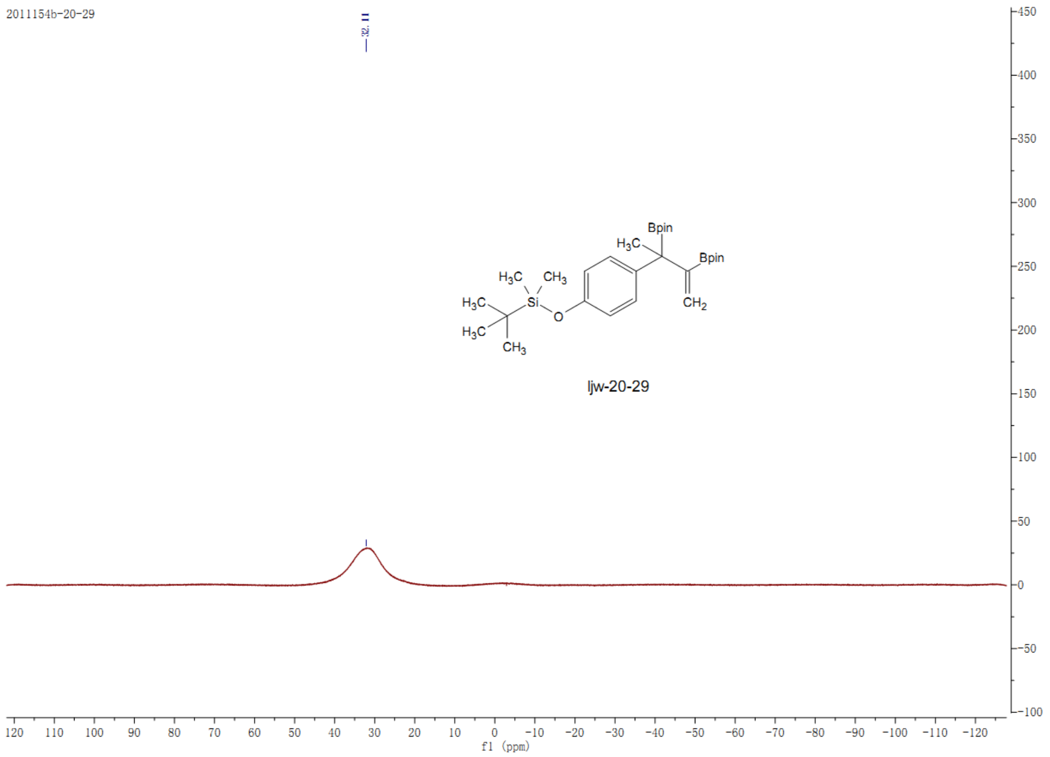

NMR spectra of 3j

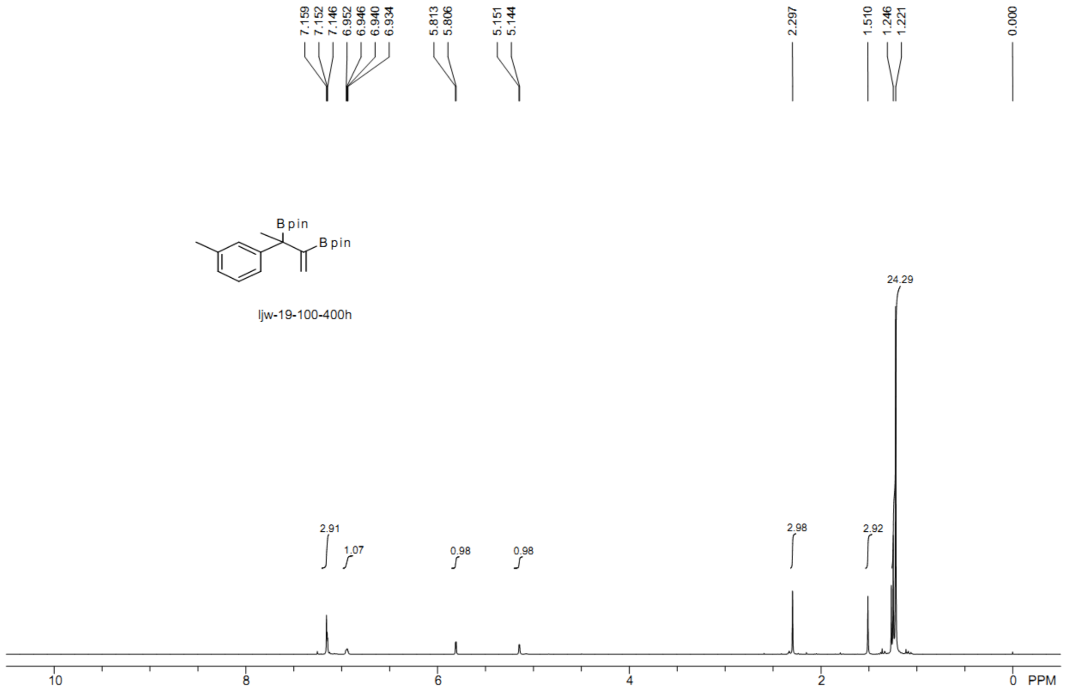

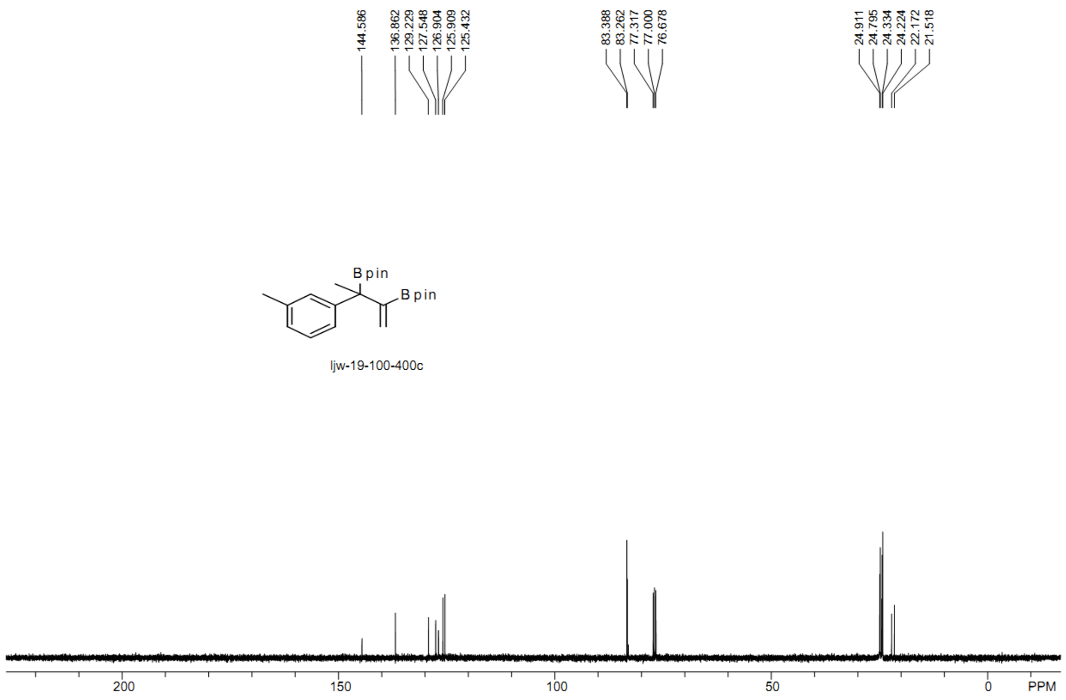
144.586
136.862
129.229
127.548
126.904
125.909
125.432
83.388
83.262
77.317
77.000
76.678
24.911
24.795
24.334
24.224
22.172
21.518
Bpin
Bpin
ljw-19-100-400c
200
150
100
50
0
PPM

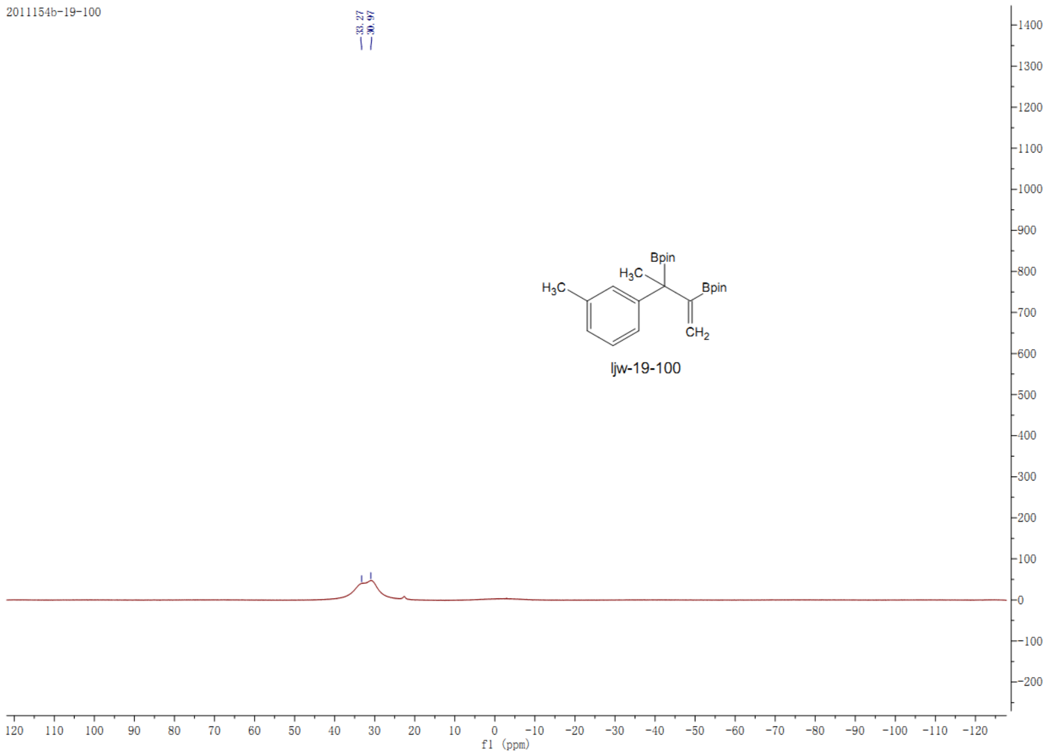
2011154b-19-100
33.27
30.97
Bpin
H3C
H3C
Bpin
CH2
ljw-19-100
f1 (ppm)

NMR spectra of 3k

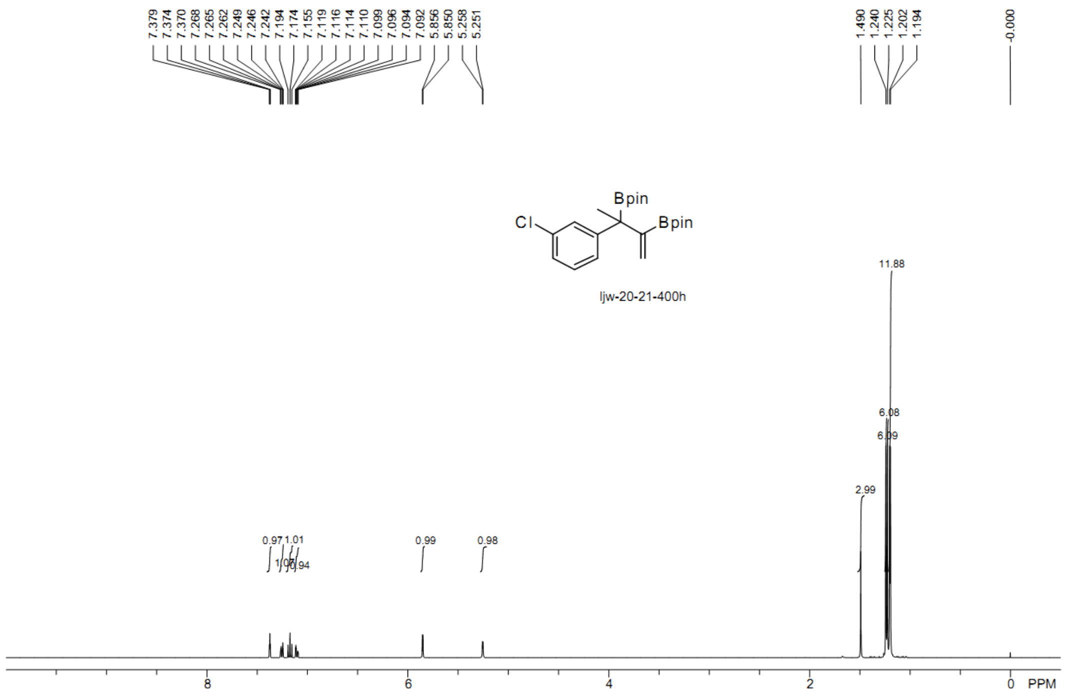

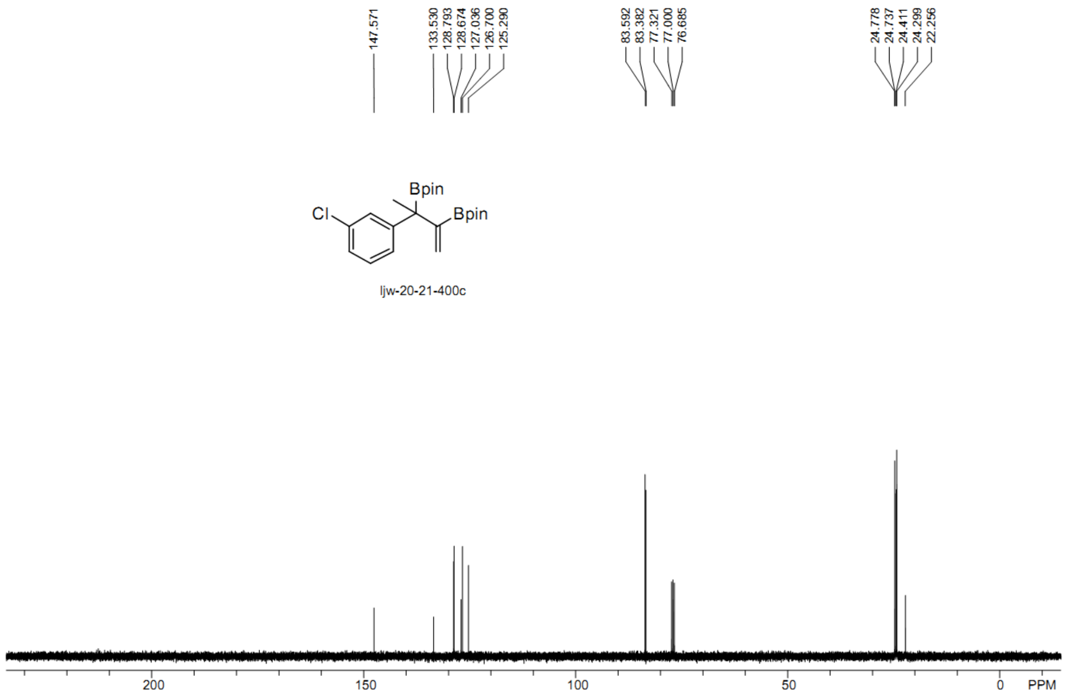

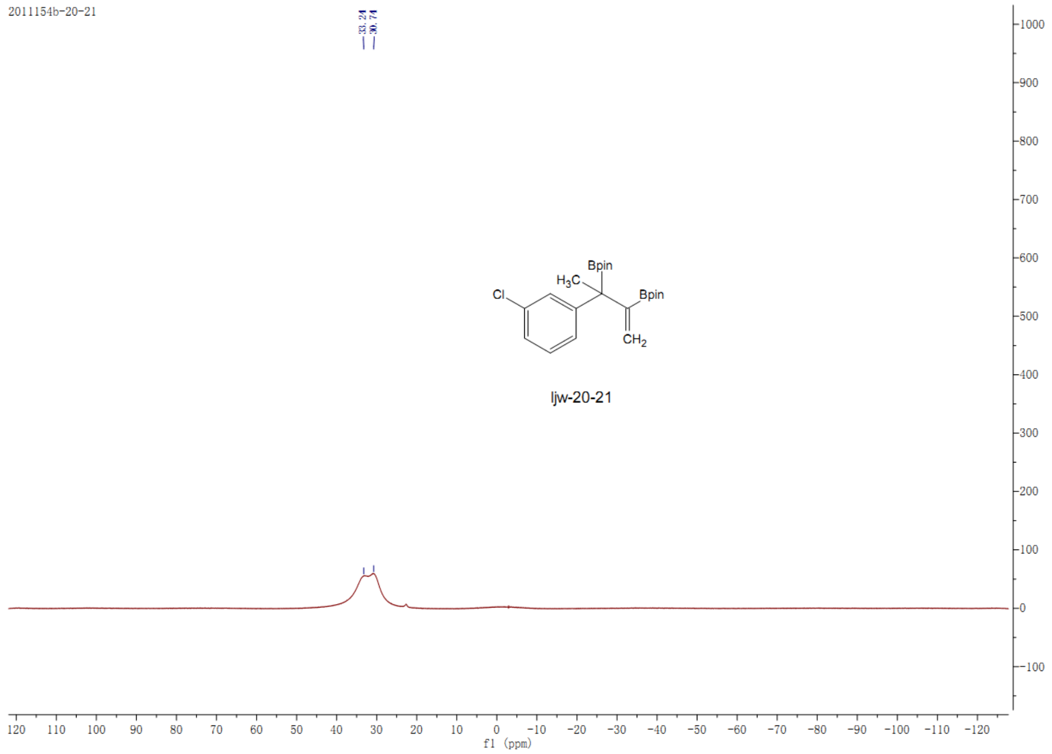

NMR spectra of 3l

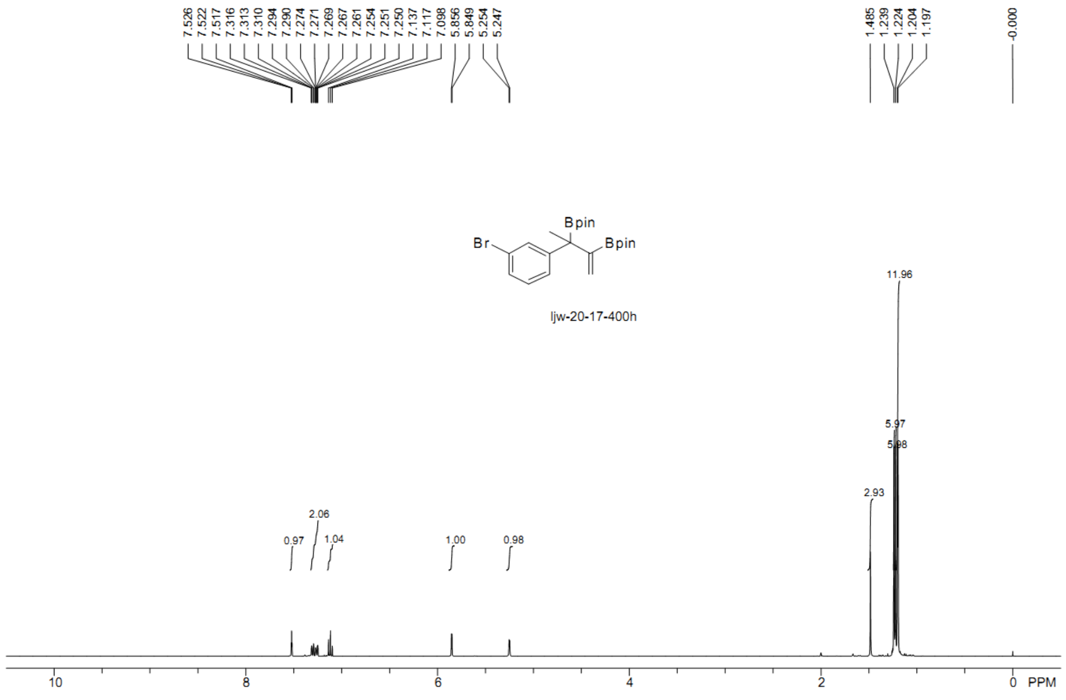

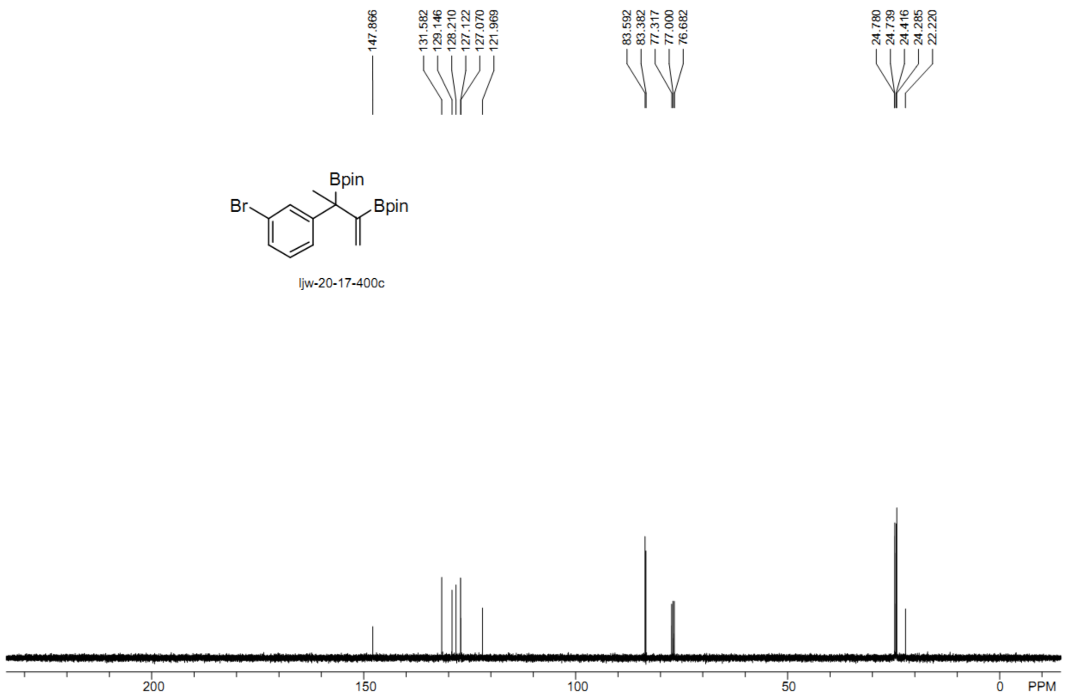
147.866
131.582
129.146
128.210
127.122
127.070
121.969
83.592
83.382
77.317
77.000
76.682
24.780
24.739
24.416
24.285
22.220
Bpin
Br
Bpin
ljw-20-17-400c
200
150
100
50
0
PPM

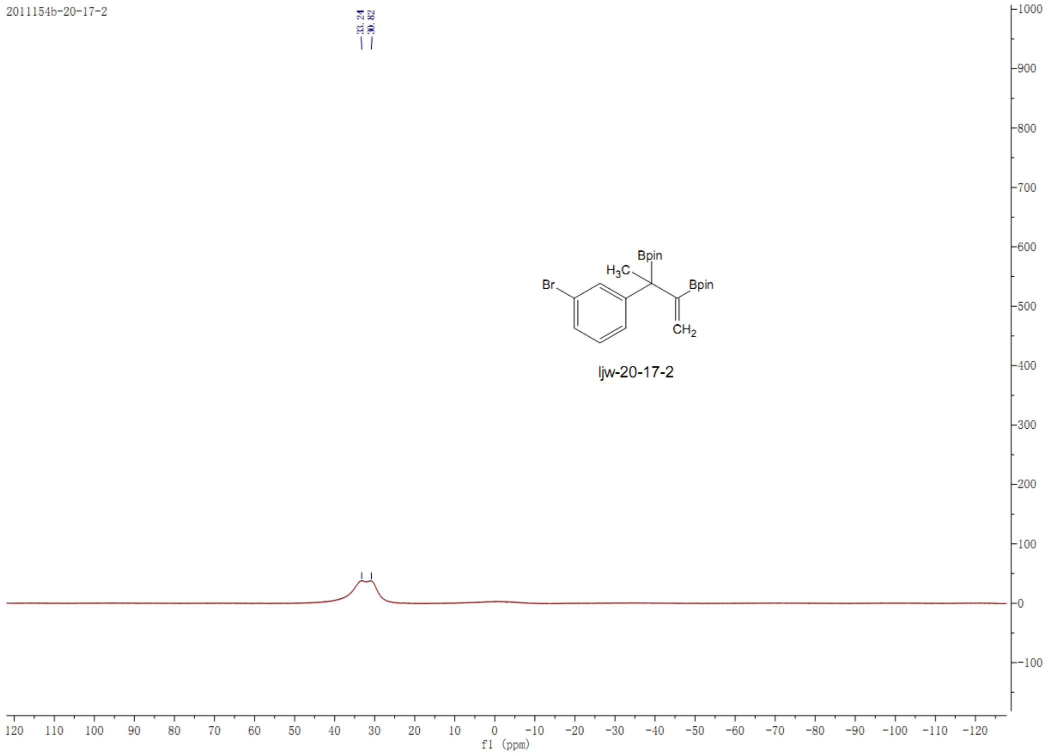
2011154b-20-17-2
33.24
30.82
Bpin
H_3C
Br
Bpin
CH_2
ljw-20-17-2
f1 (ppm)

NMR spectra of 3m

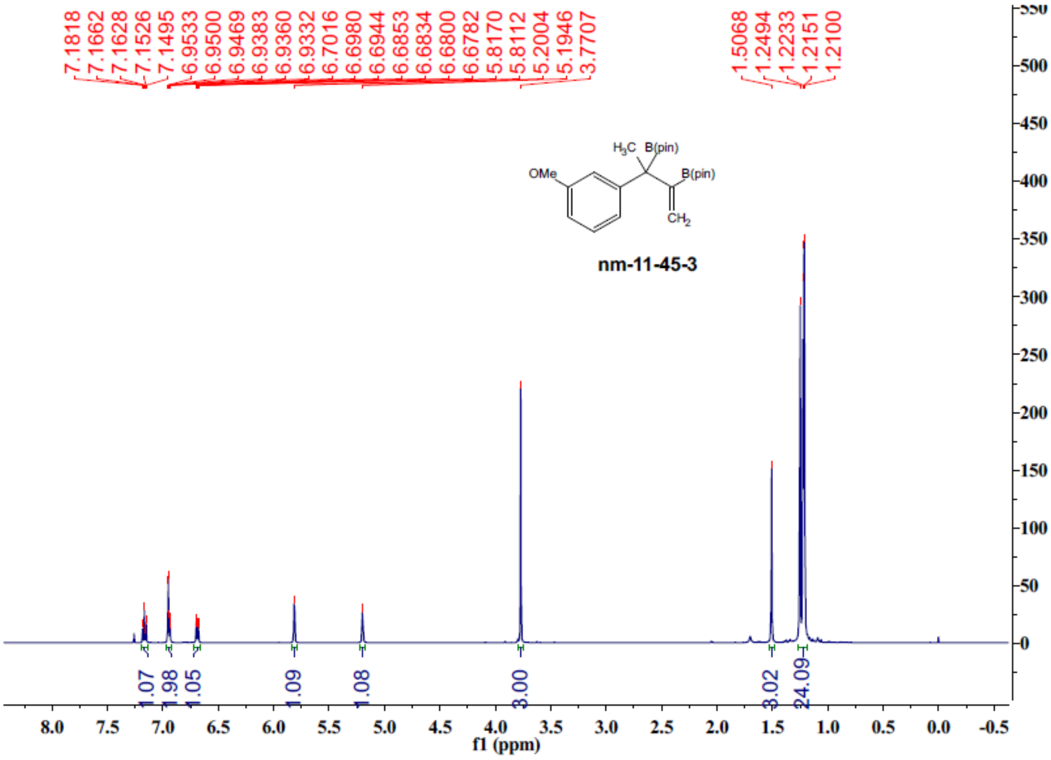

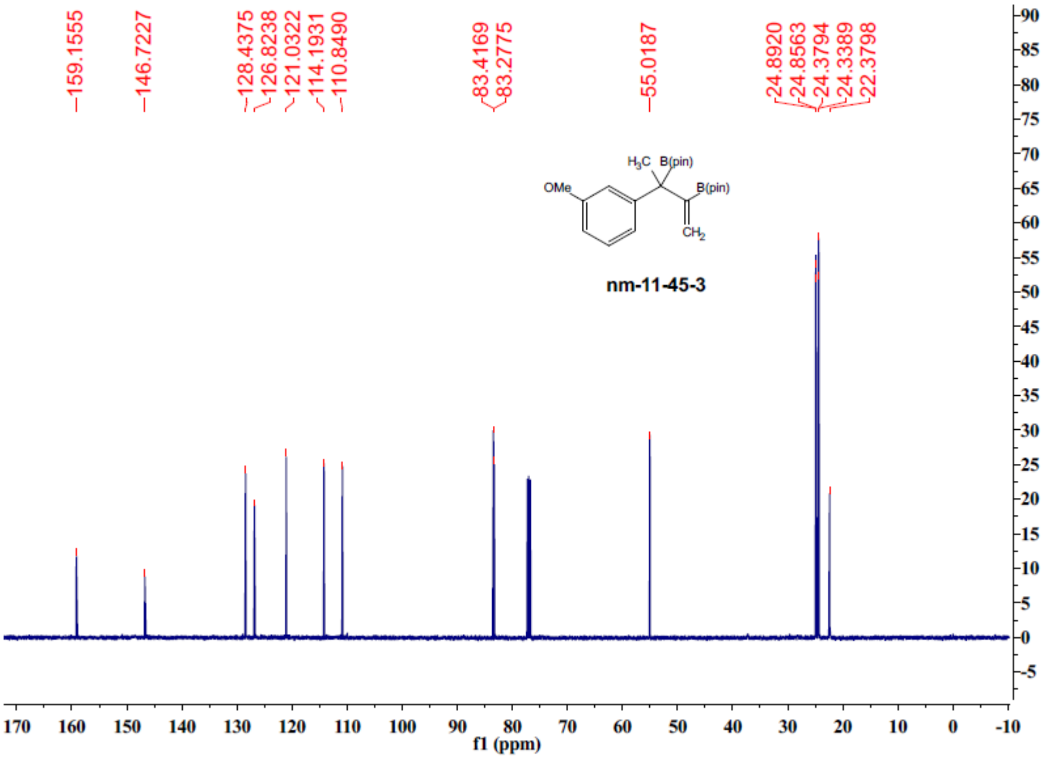

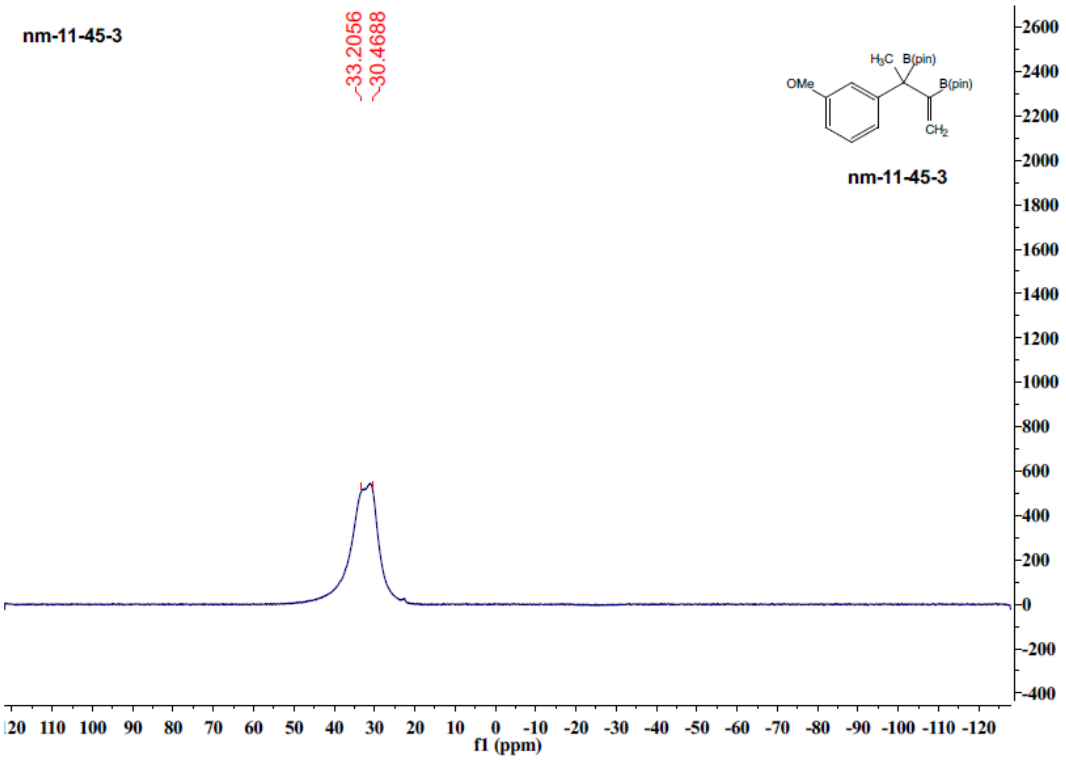

NMR spectra of 3n

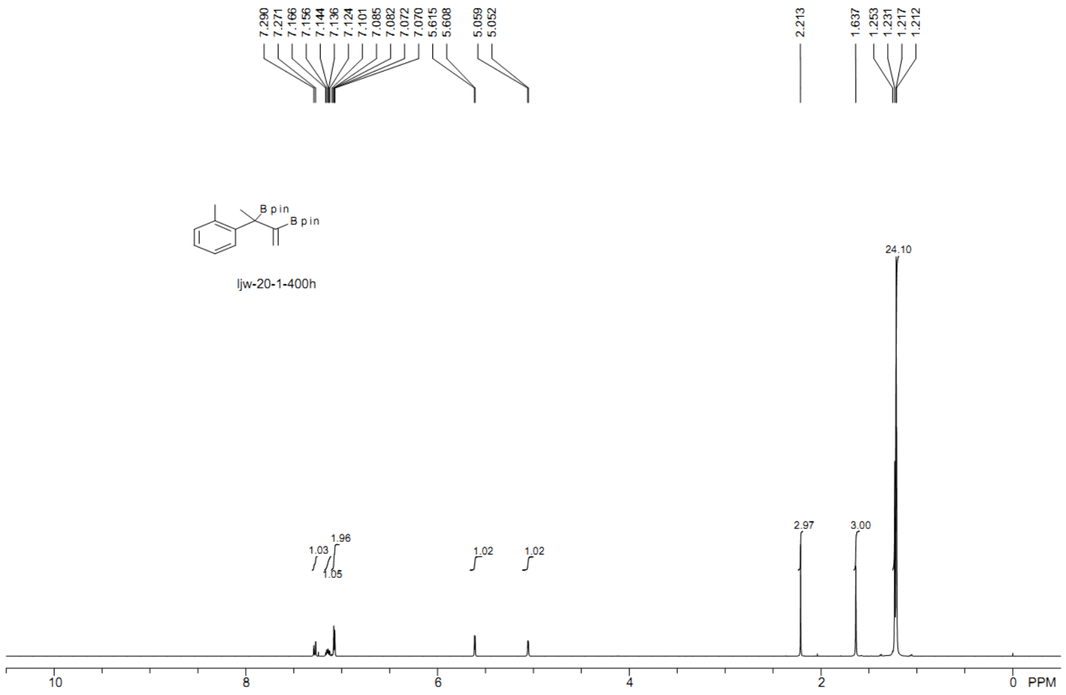

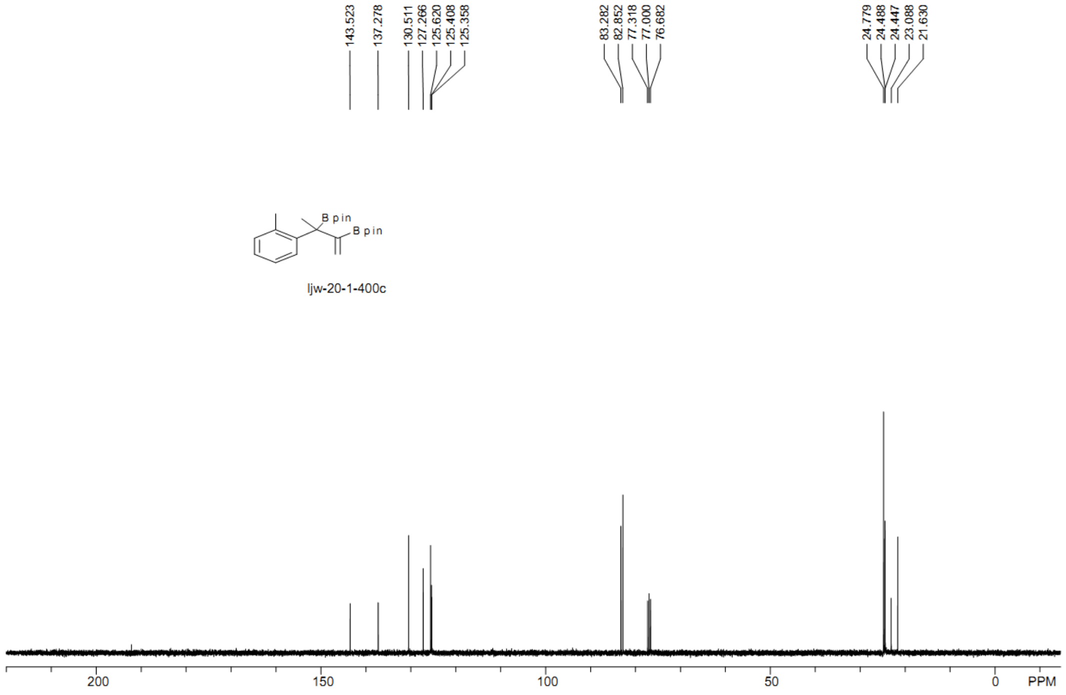
143.523
137.278
130.511
127.266
125.620
125.408
125.358
83.282
82.852
77.318
77.000
76.682
24.779
24.488
24.447
23.088
21.630
Bpin
Bpin
ljw-20-1-400c
200
150
100
50
0
PPM

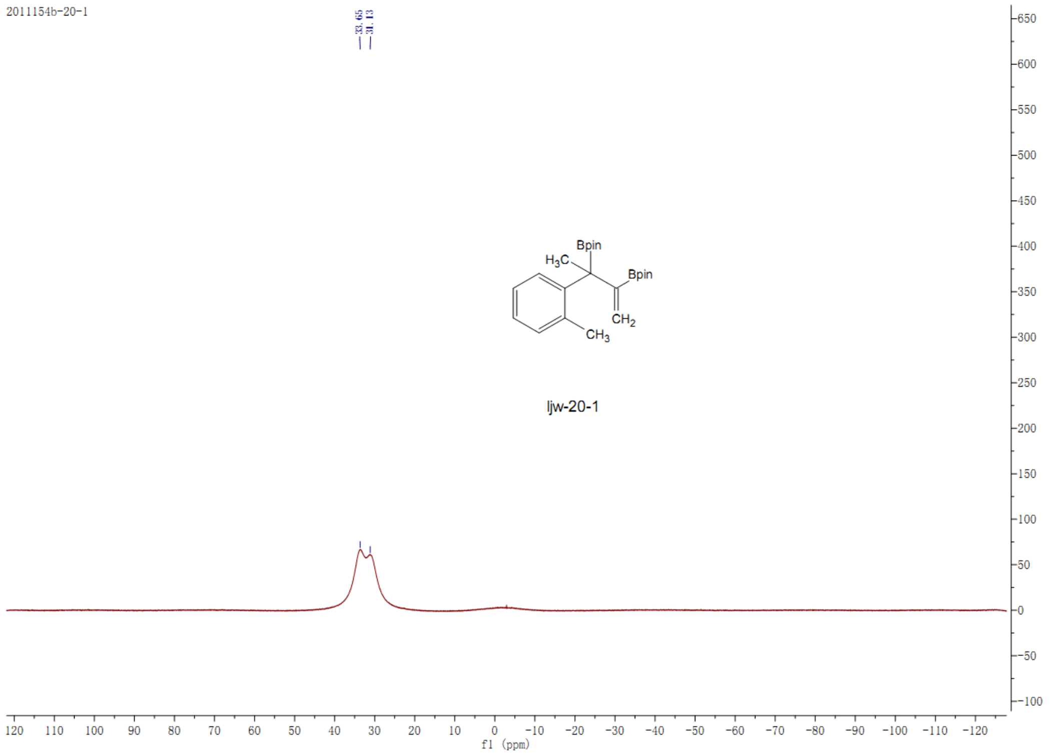
2011154b-20-1
33.65
31.13
Bpin
H_3C
Bpin
CH_2
CH_3
ljw-20-1
f1 (ppm)

NMR spectra of 3o

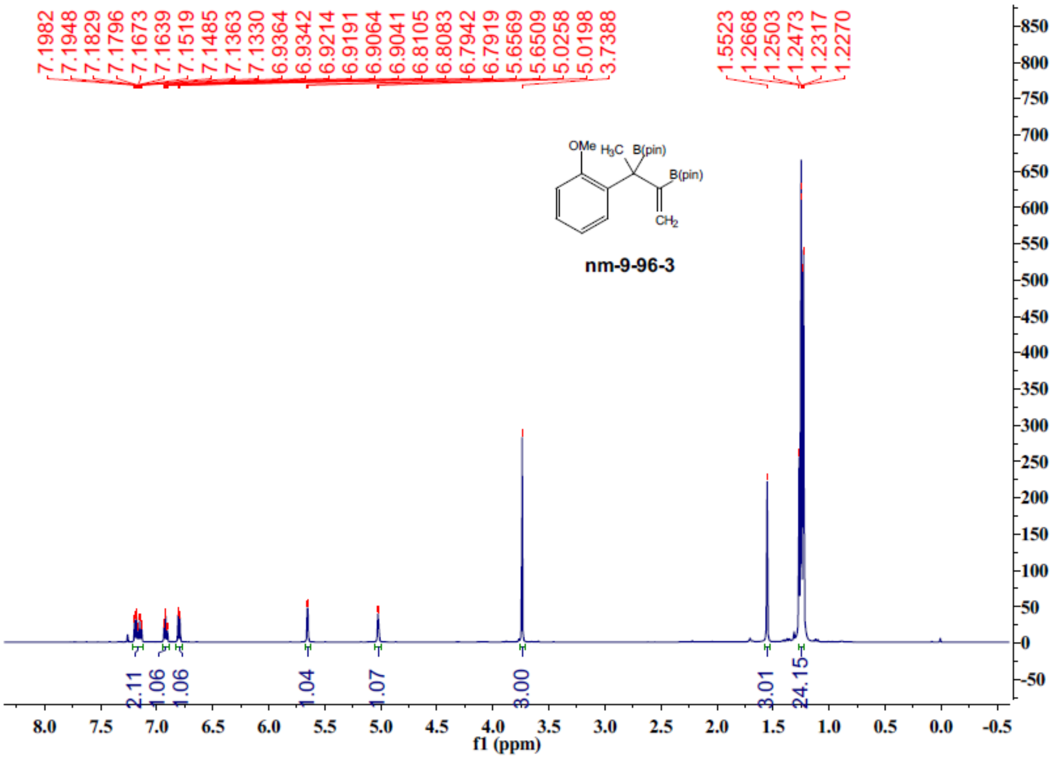

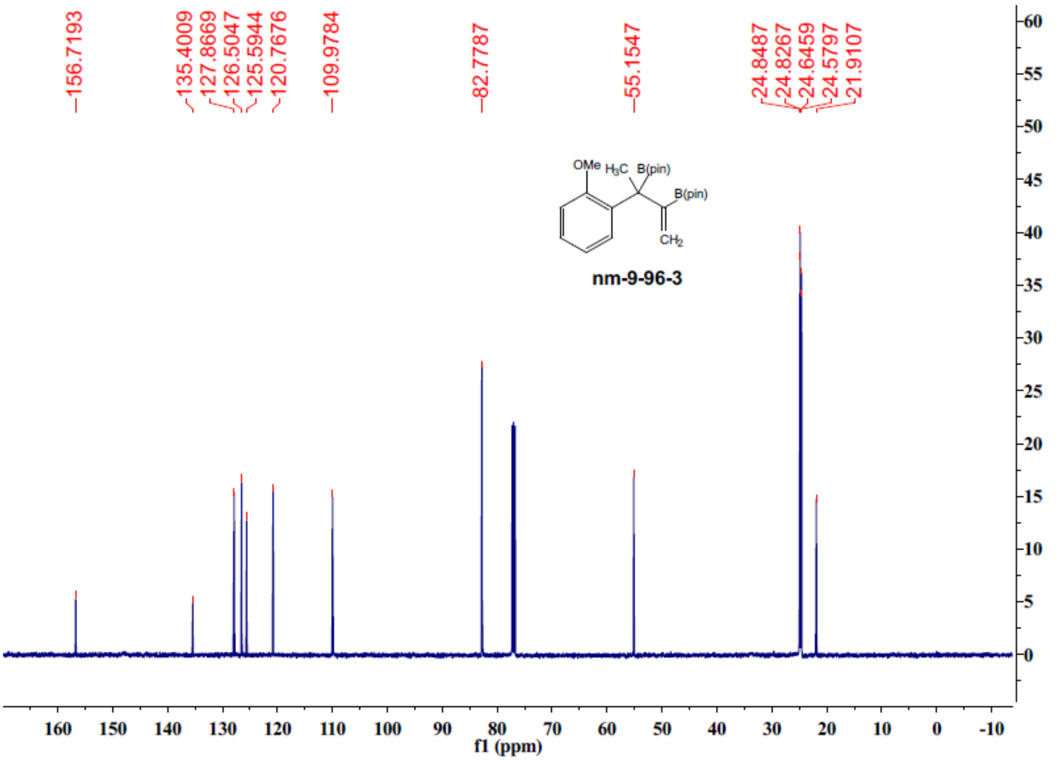

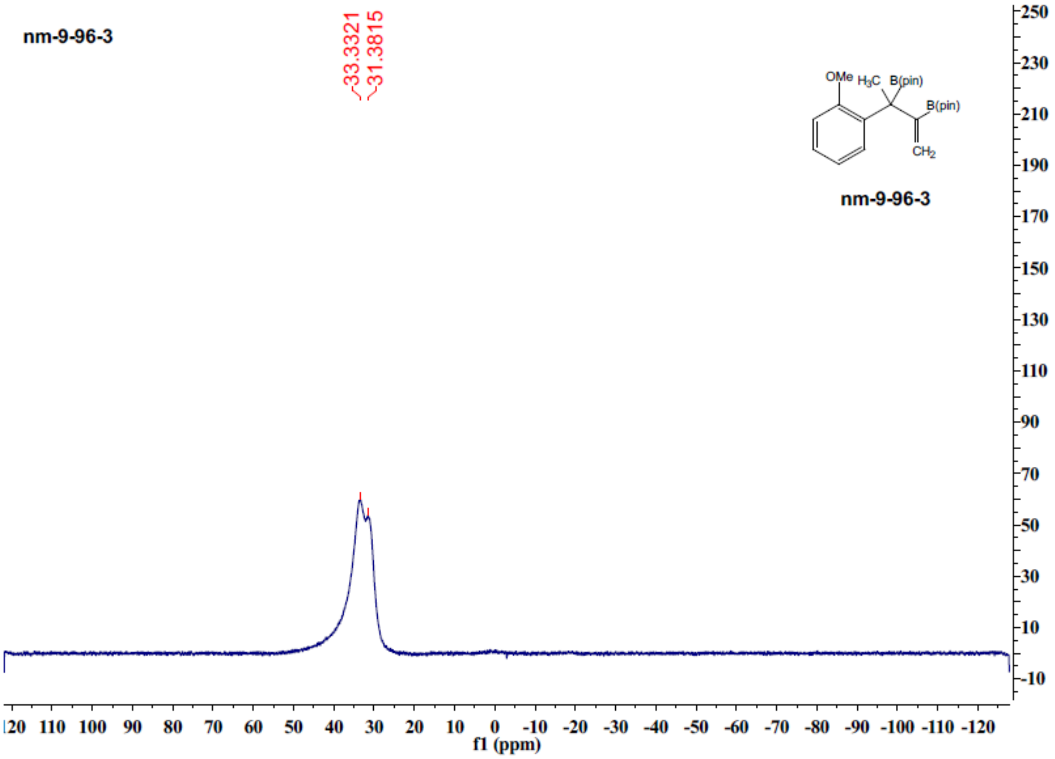

NMR spectra of 3p

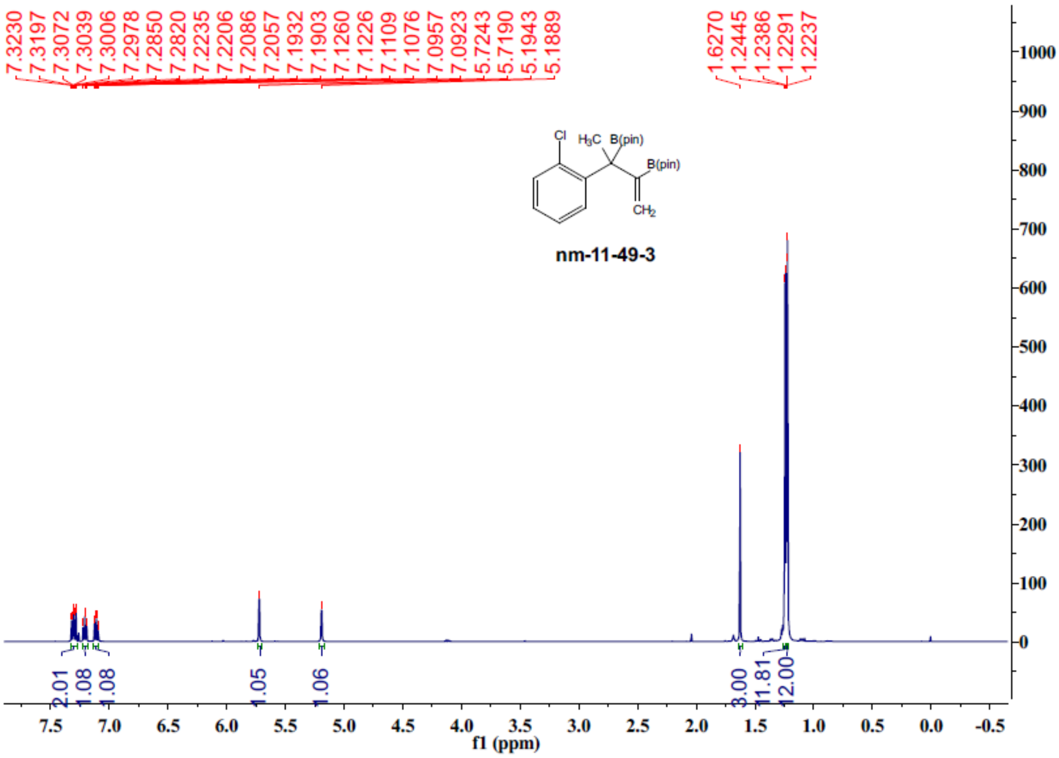

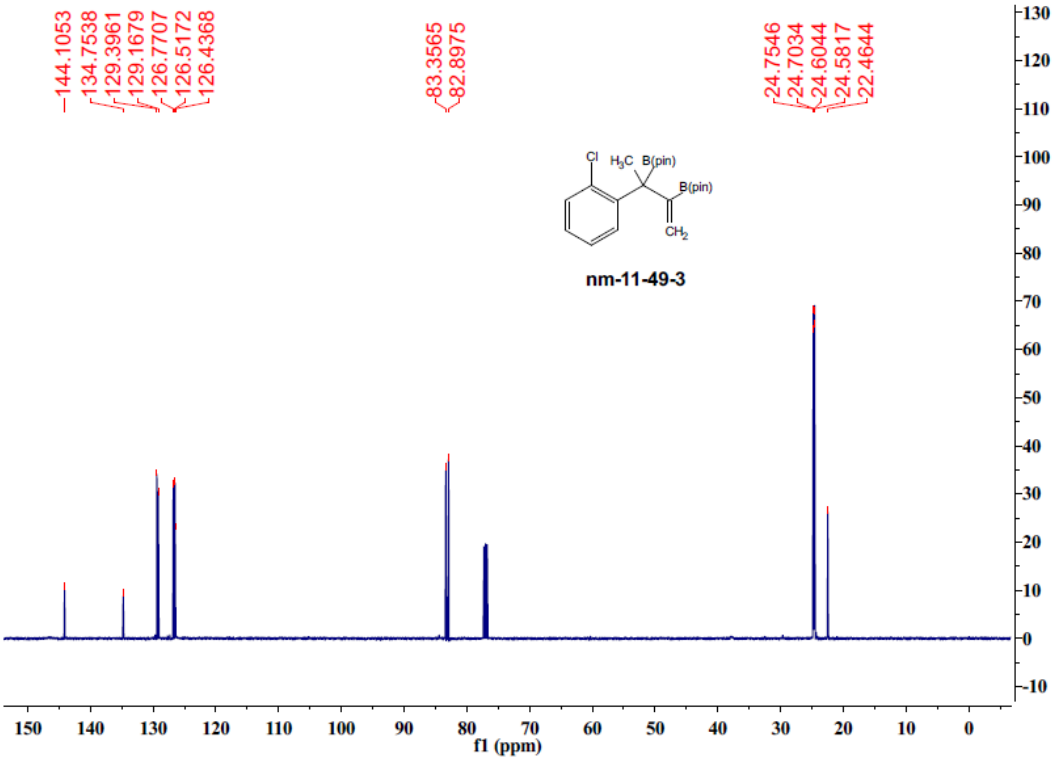

144.1053
134.7538
129.3961
129.1679
126.7707
126.5172
126.4368
83.3565
82.8975
24.7546
24.7034
24.6044
24.5817
22.4644
Cl
H3C
B(pin)
B(pin)
CH2
nm-11-49-3
f1 (ppm)

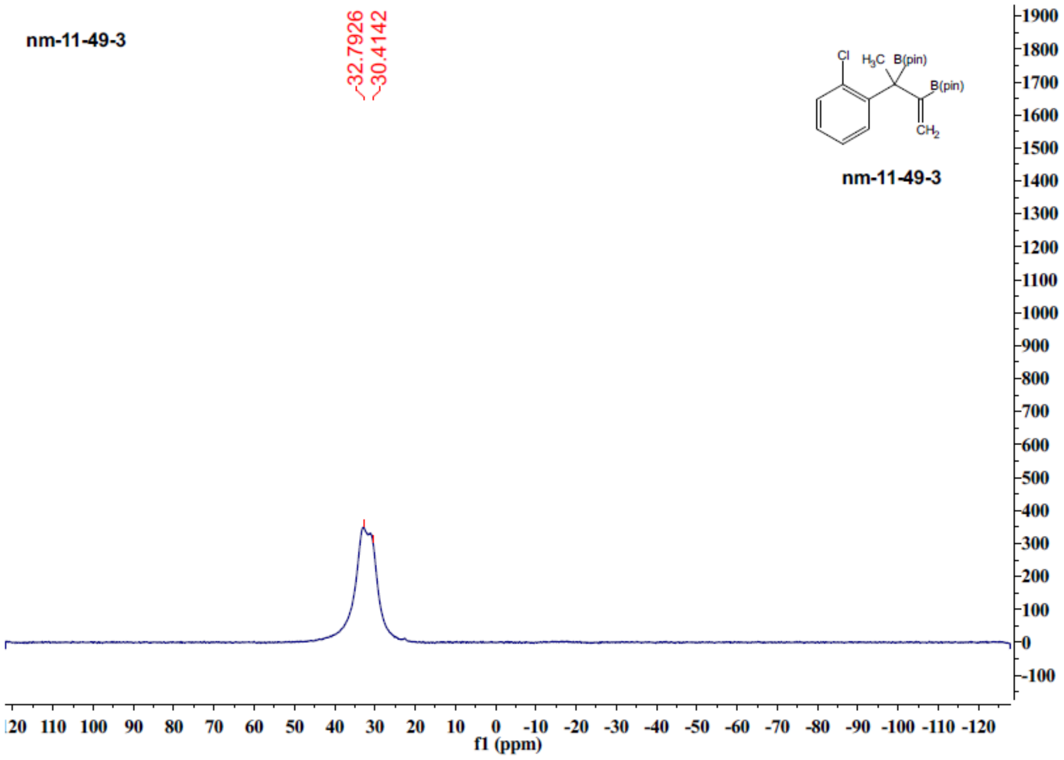

nm-11-49-3
32.7926
30.4142
Cl
H3C
B(pin)
B(pin)
CH2
nm-11-49-3
f1 (ppm)

NMR spectra of 3q

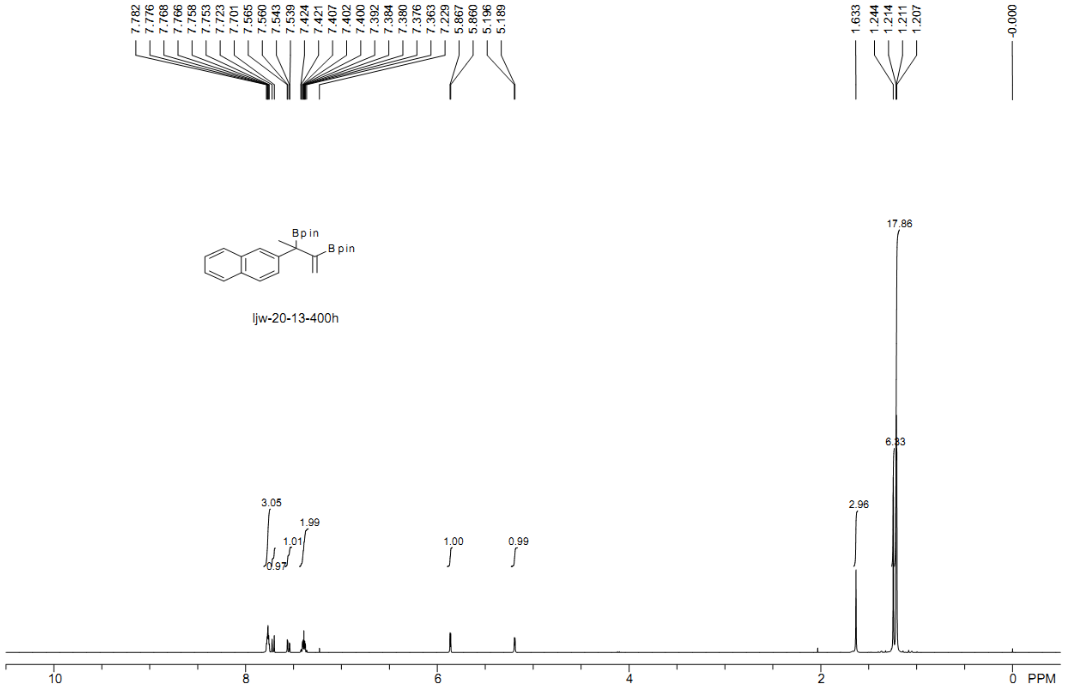

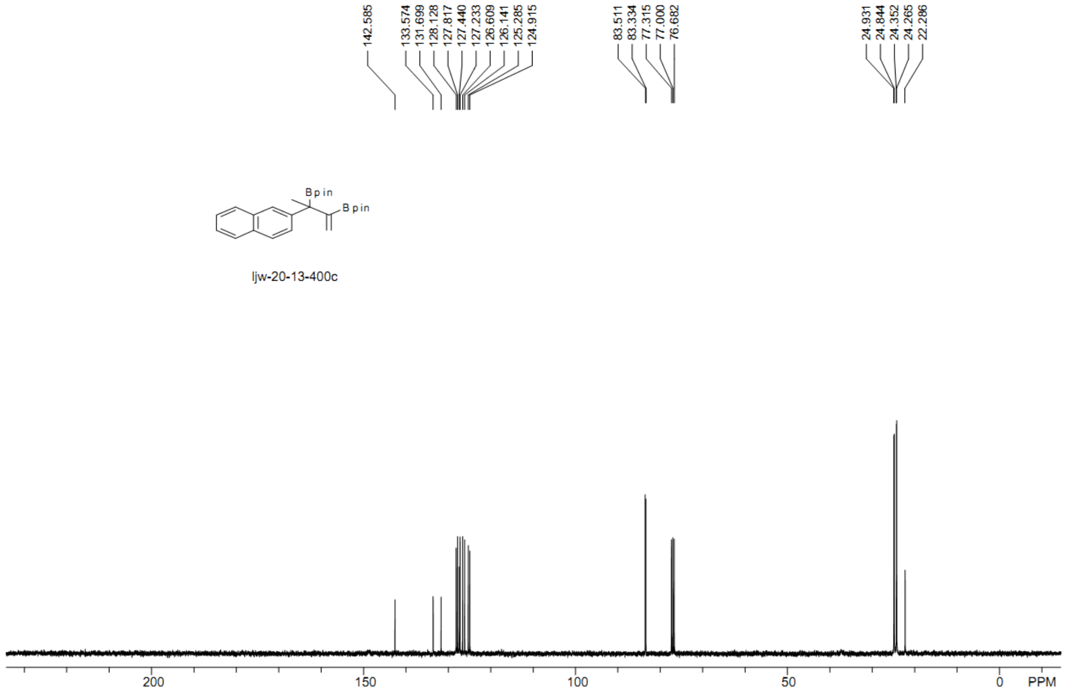

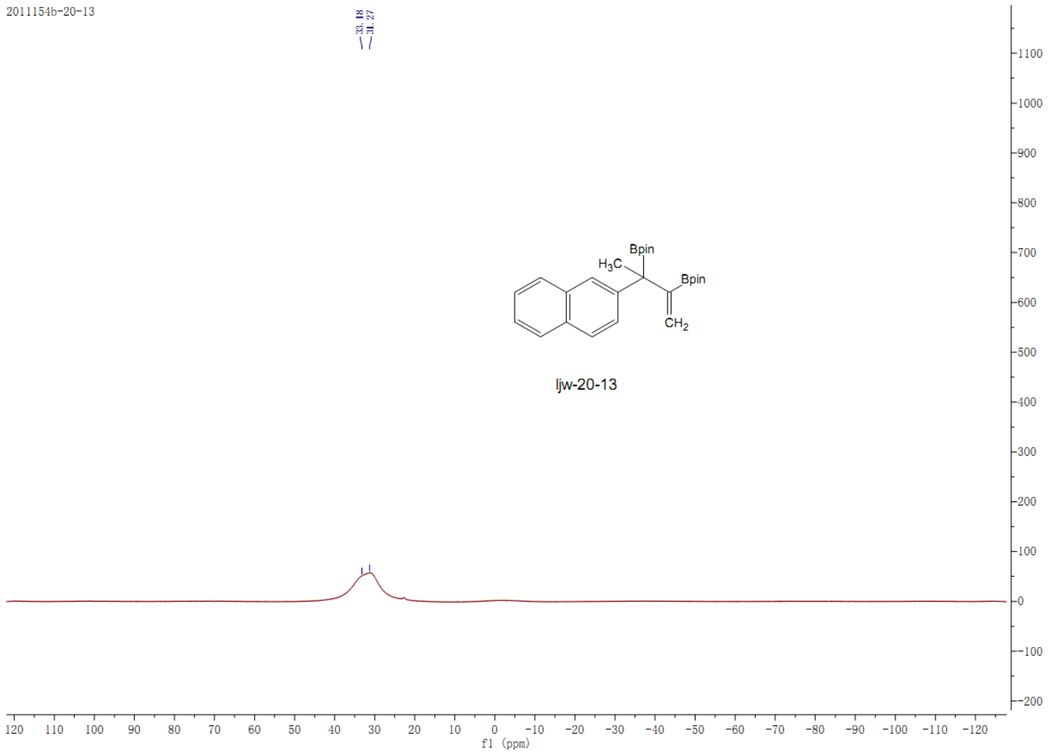

NMR spectra of 3r

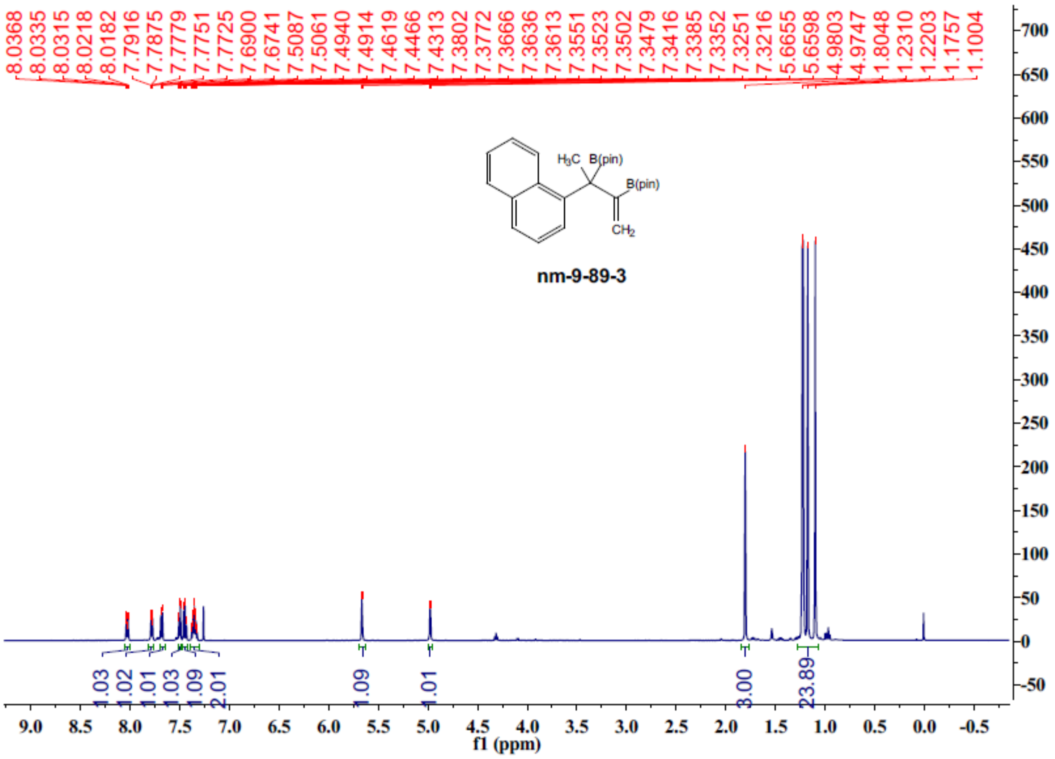

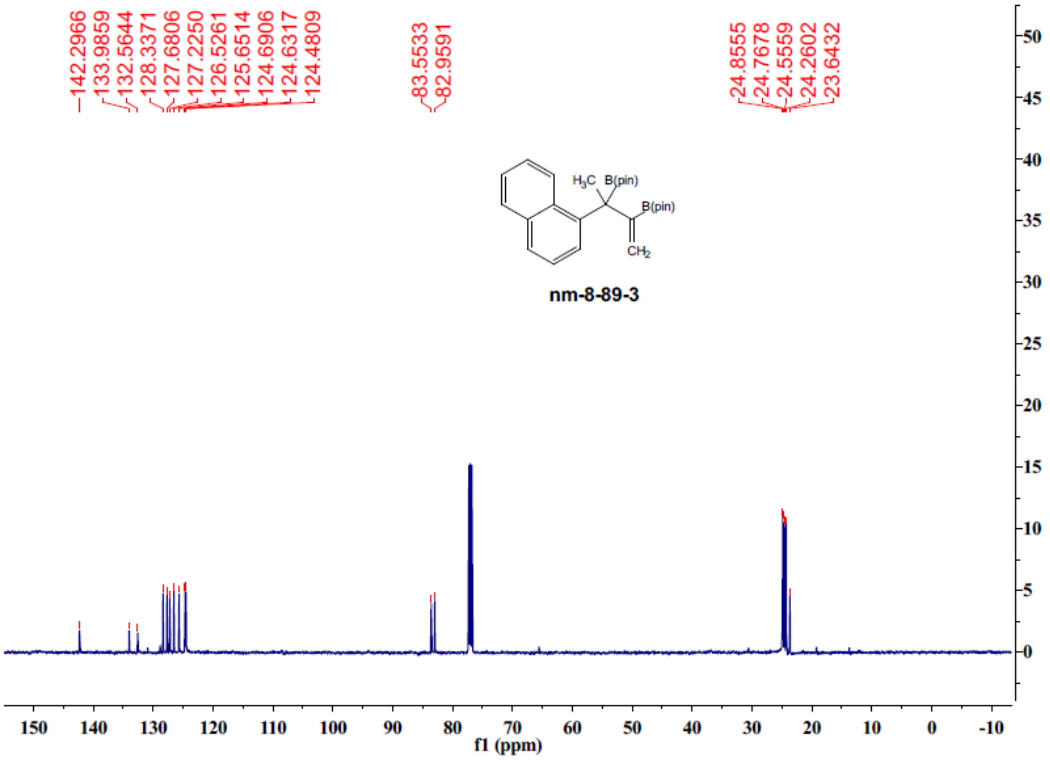
142.2966
133.9859
132.5644
128.3371
127.6806
127.2250
126.5261
125.6514
124.6906
124.6317
124.4809
83.5533
82.9591
24.8555
24.7678
24.5559
24.2602
23.6432
H3C B(pin)
B(pin)
CH2
nm-8-89-3
f1 (ppm)

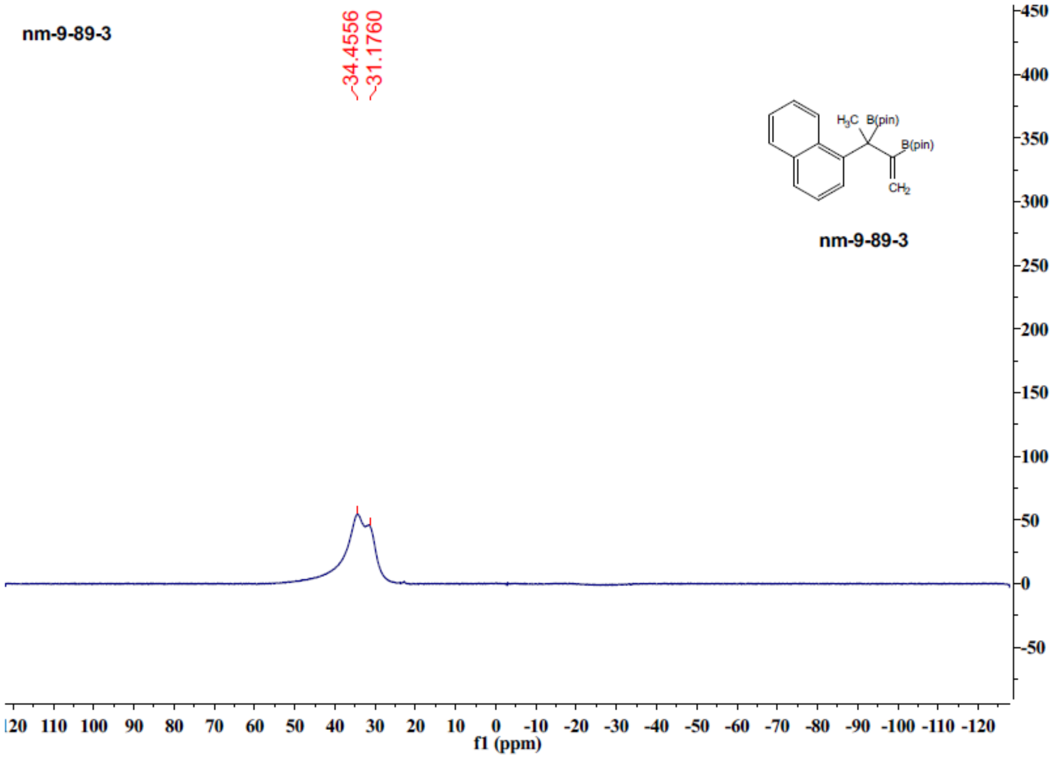
nm-9-89-3
34.4556
31.1760
H3C B(pin)
B(pin)
CH2
nm-9-89-3
f1 (ppm)

NMR spectra of 3s

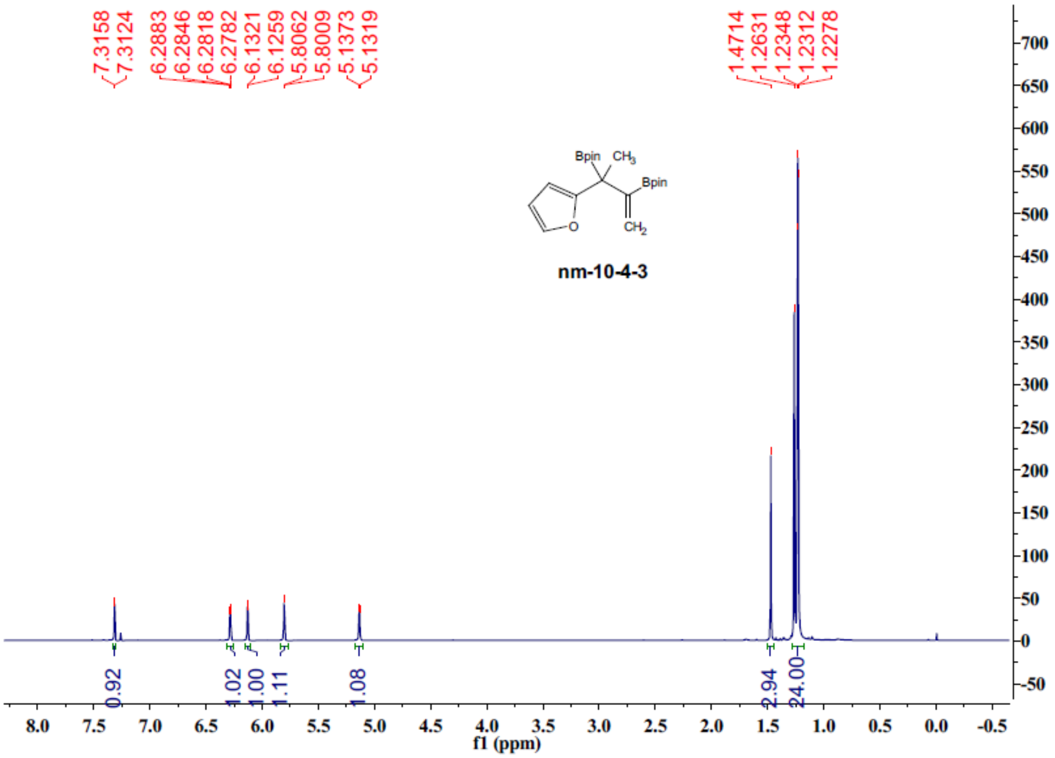

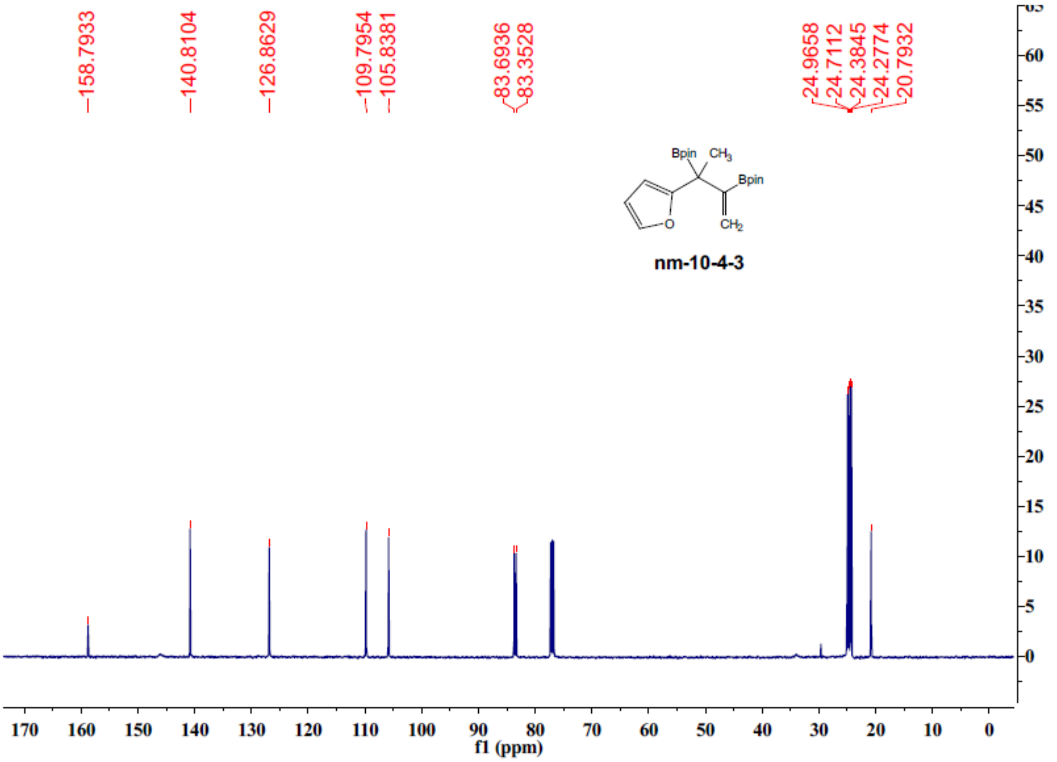

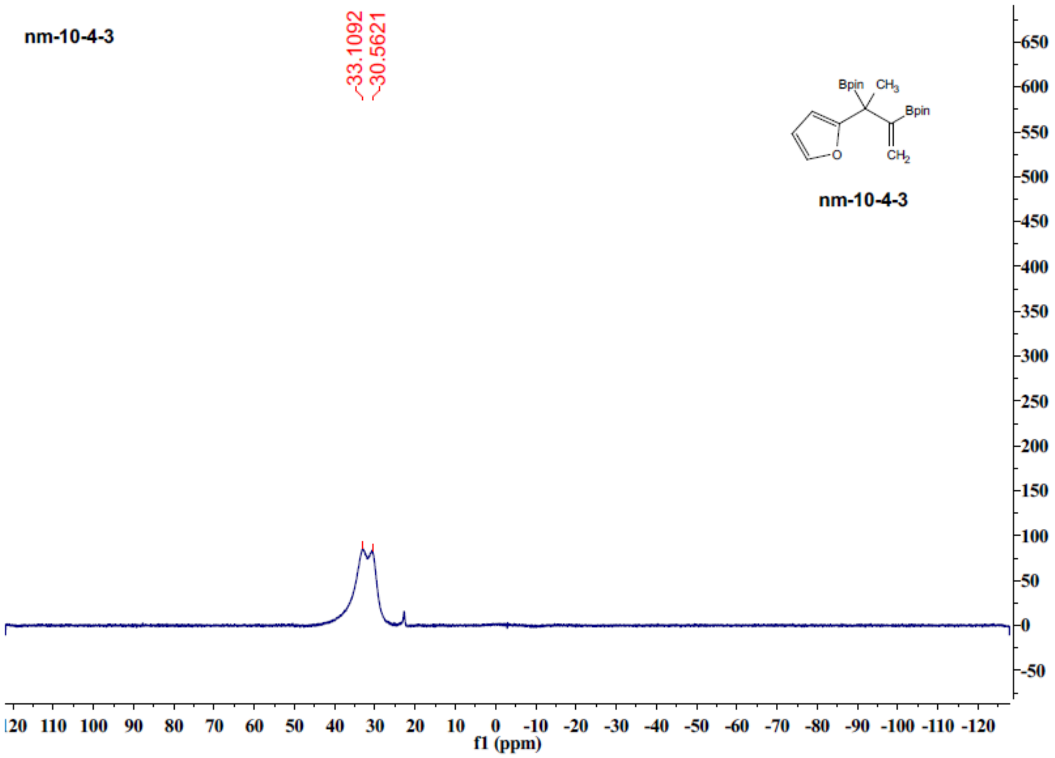

NMR spectra of 3t

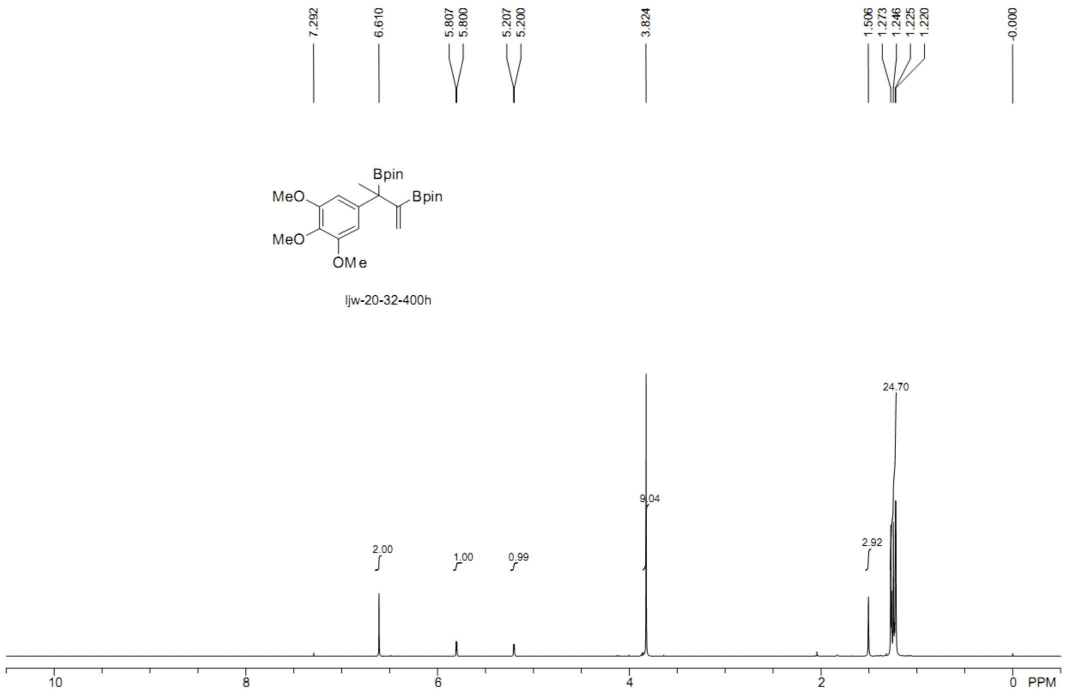

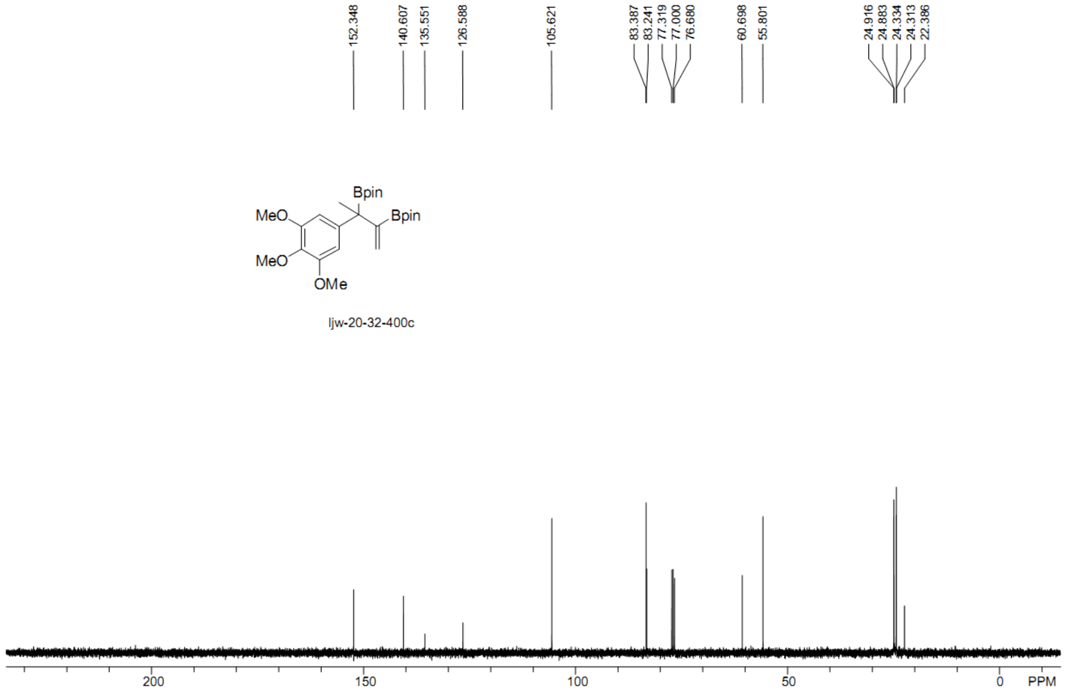
152.348
140.607
135.551
126.588
105.621
83.387
83.241
77.319
77.000
76.680
60.698
55.801
24.916
24.883
24.334
24.313
22.386
Bpin
MeO
Bpin
MeO
OMe
ljw-20-32-400c
200
150
100
50
0
PPM

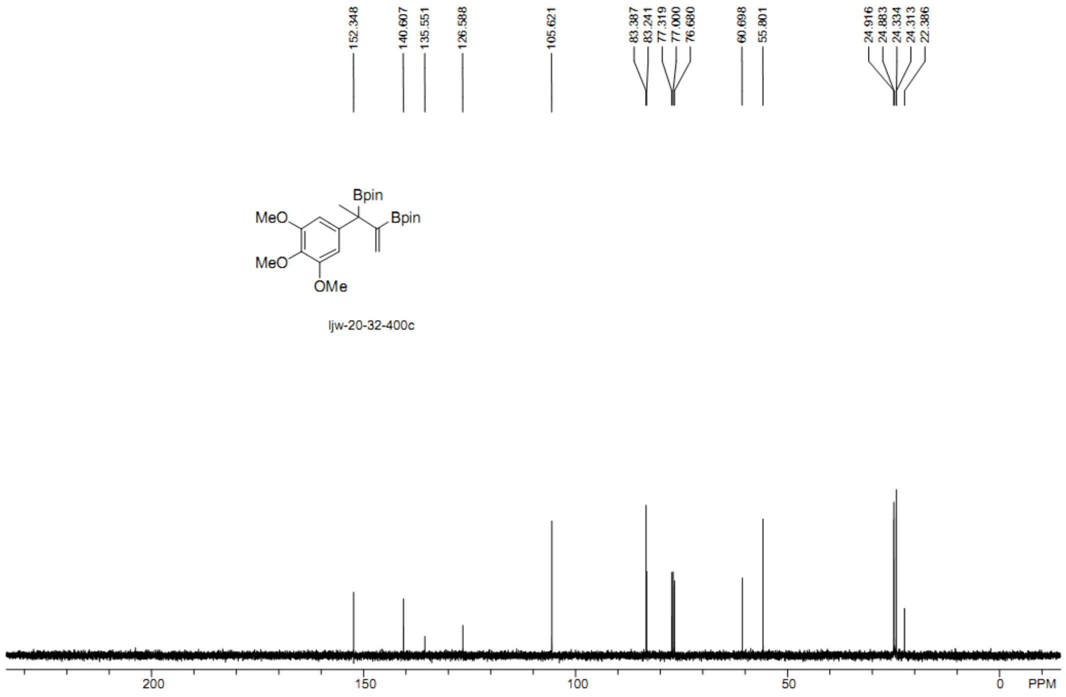
152.348
140.607
135.551
126.588
105.621
83.387
83.241
77.319
77.000
76.680
60.698
55.801
24.916
24.883
24.334
24.313
22.386
Bpin
MeO
Bpin
MeO
OMe
ljw-20-32-400c
200
150
100
50
0
PPM

NMR spectra of 3u

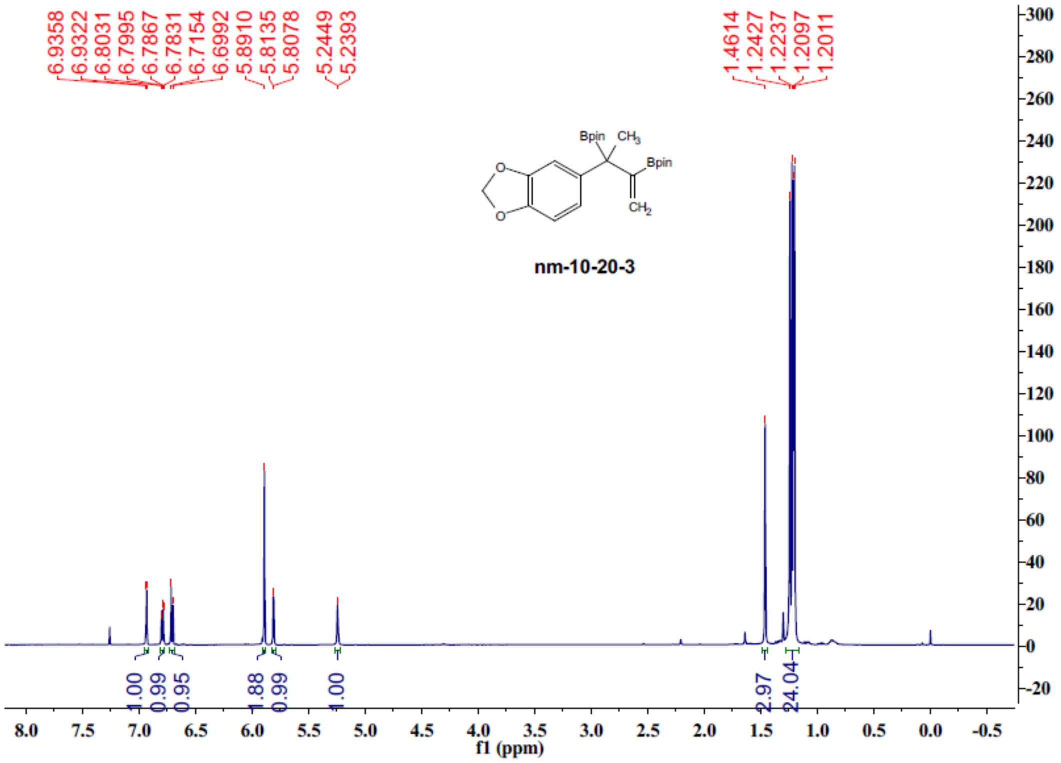

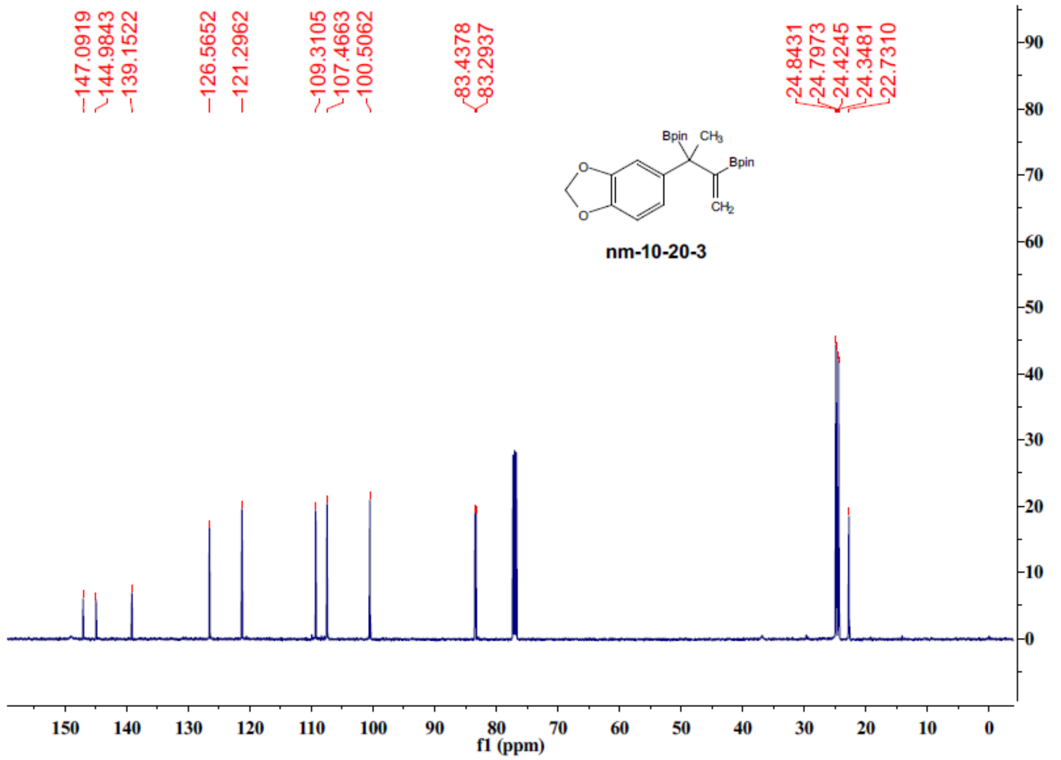

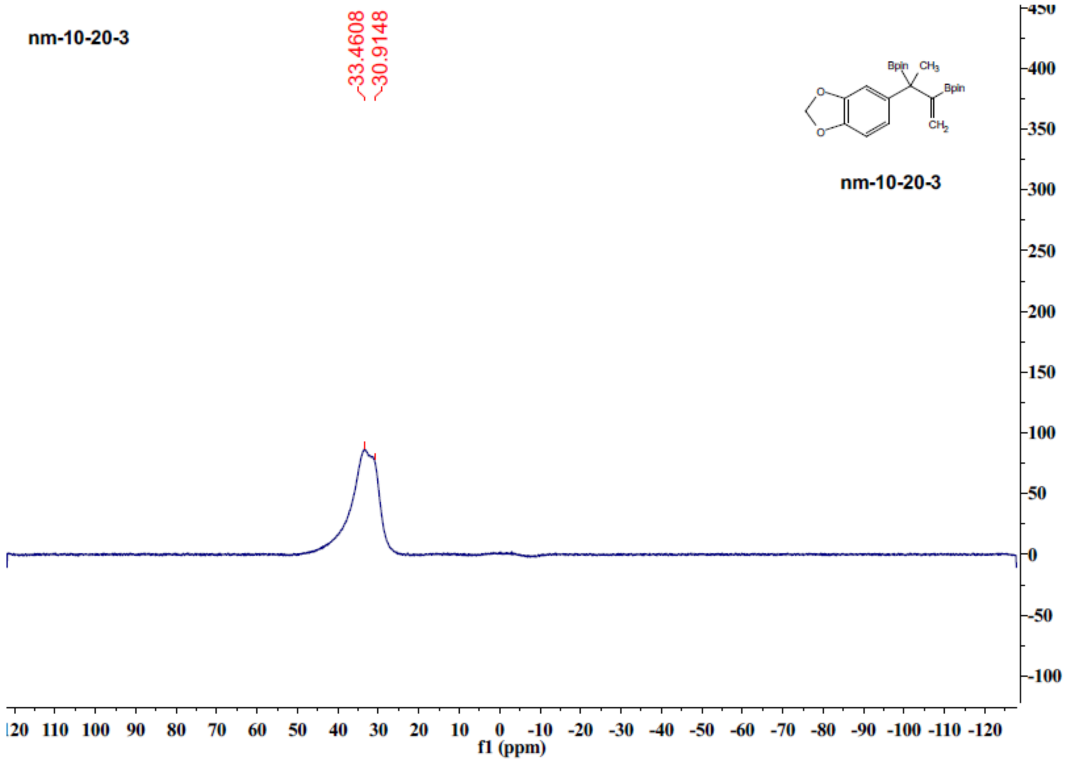

NMR spectra of 3v

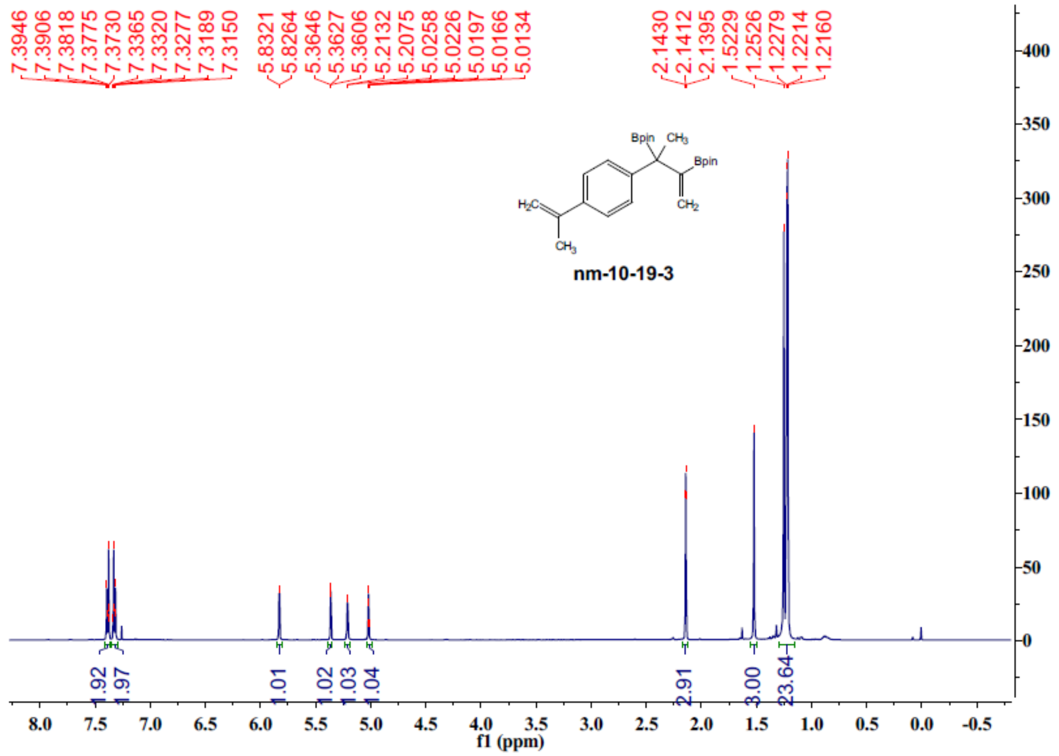

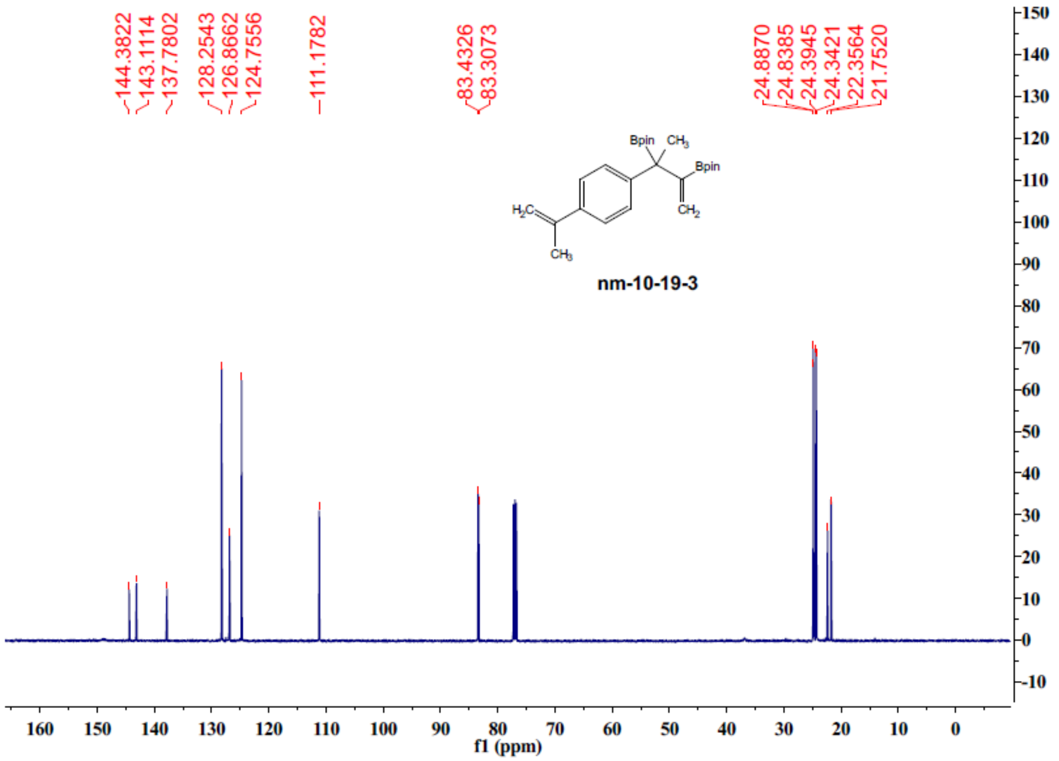

144.3822
143.1114
137.7802
128.2543
126.8662
124.7556
111.1782
83.4326
83.3073
24.8870
24.8385
24.3945
24.3421
22.3564
21.7520
Bpin
CH3
Bpin
H2C
CH2
CH3
nm-10-19-3
f1 (ppm)

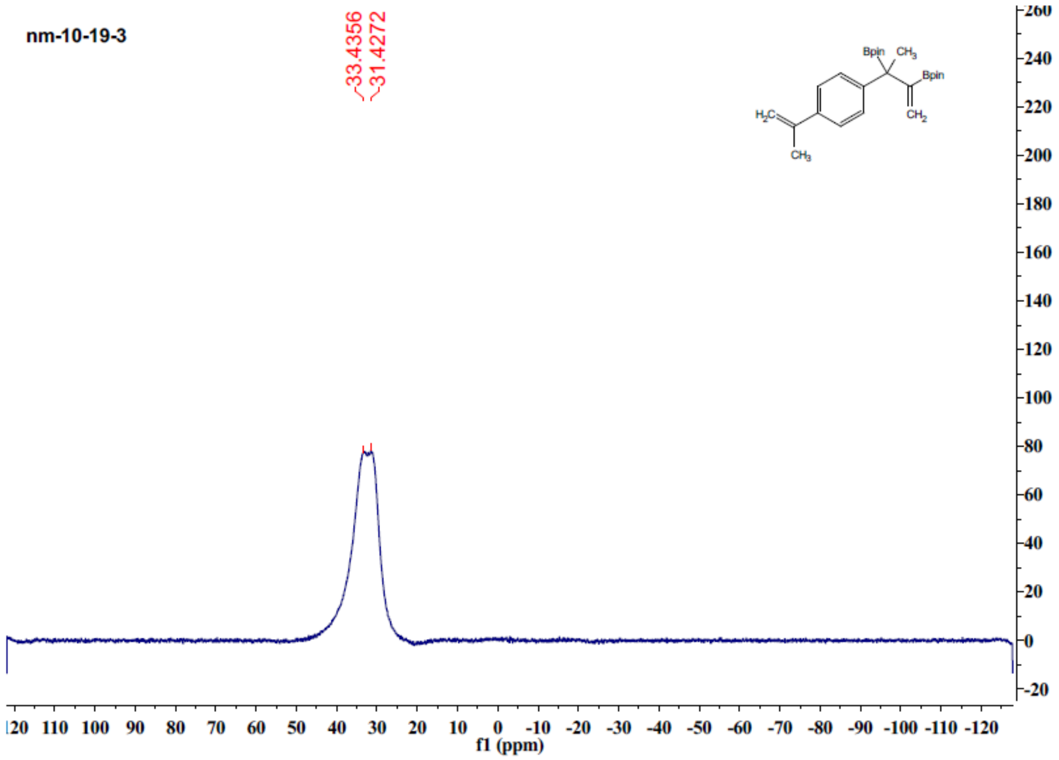

nm-10-19-3
33.4356
31.4272
Bpin
CH3
Bpin
H2C
CH2
CH3
f1 (ppm)

NMR spectra of 3w

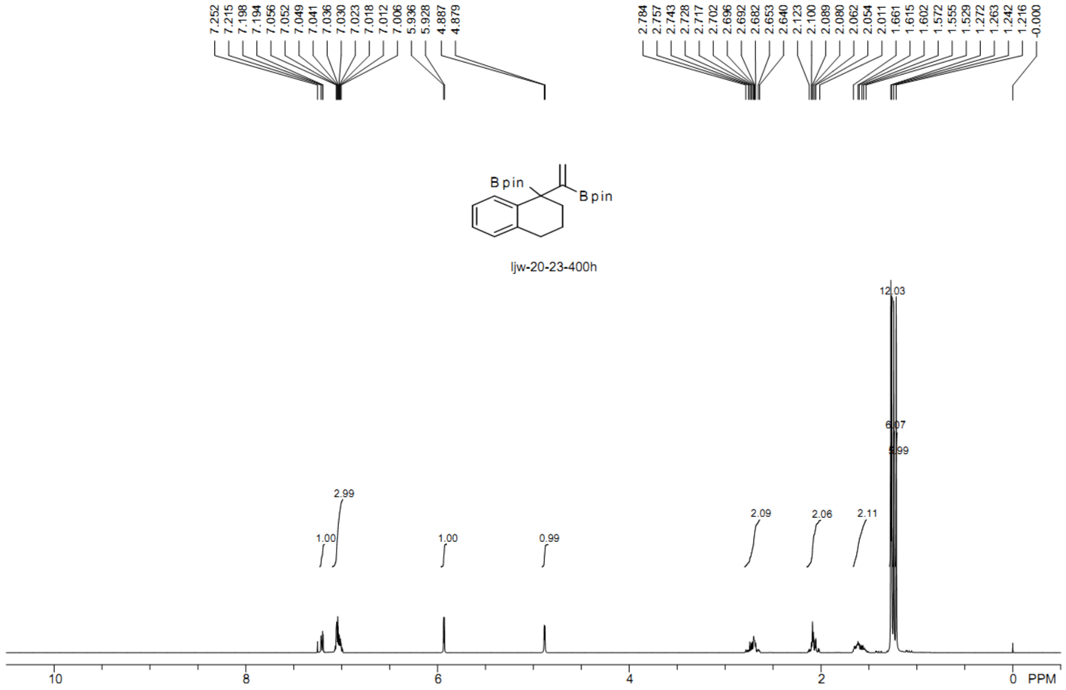

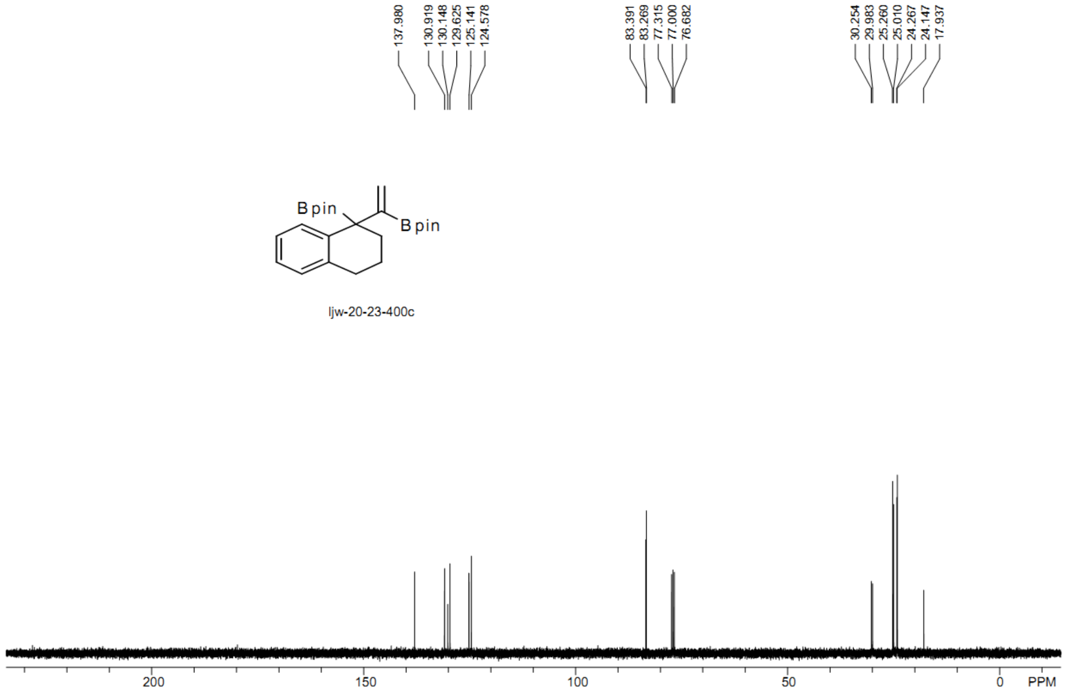

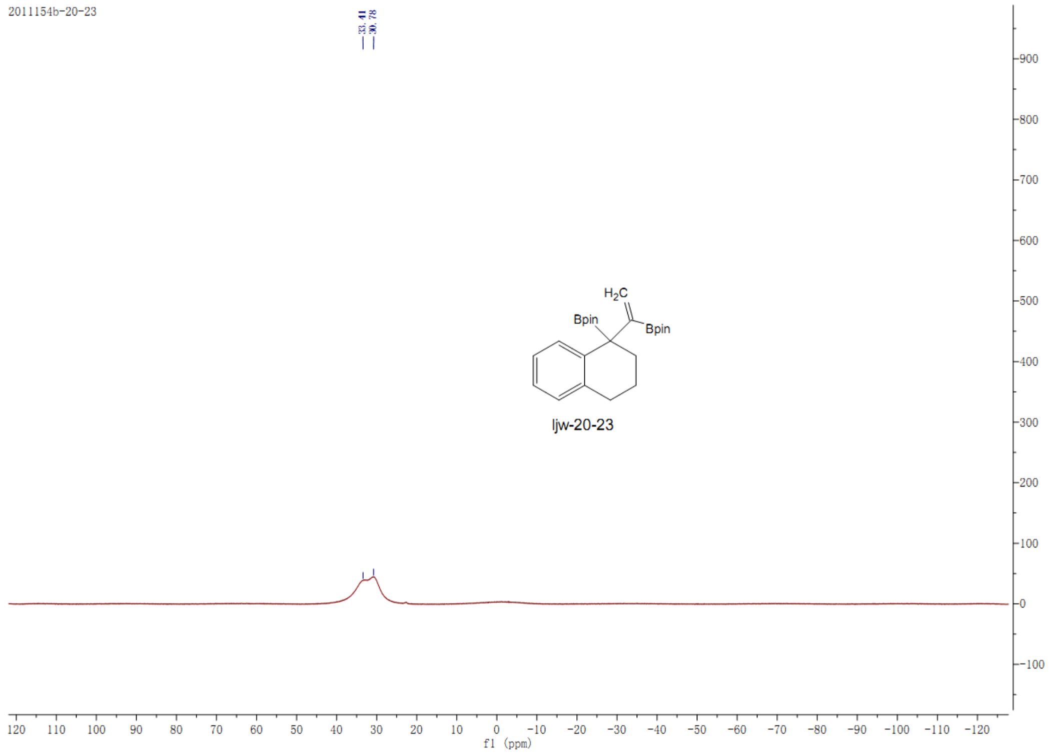

NMR spectra of 3x

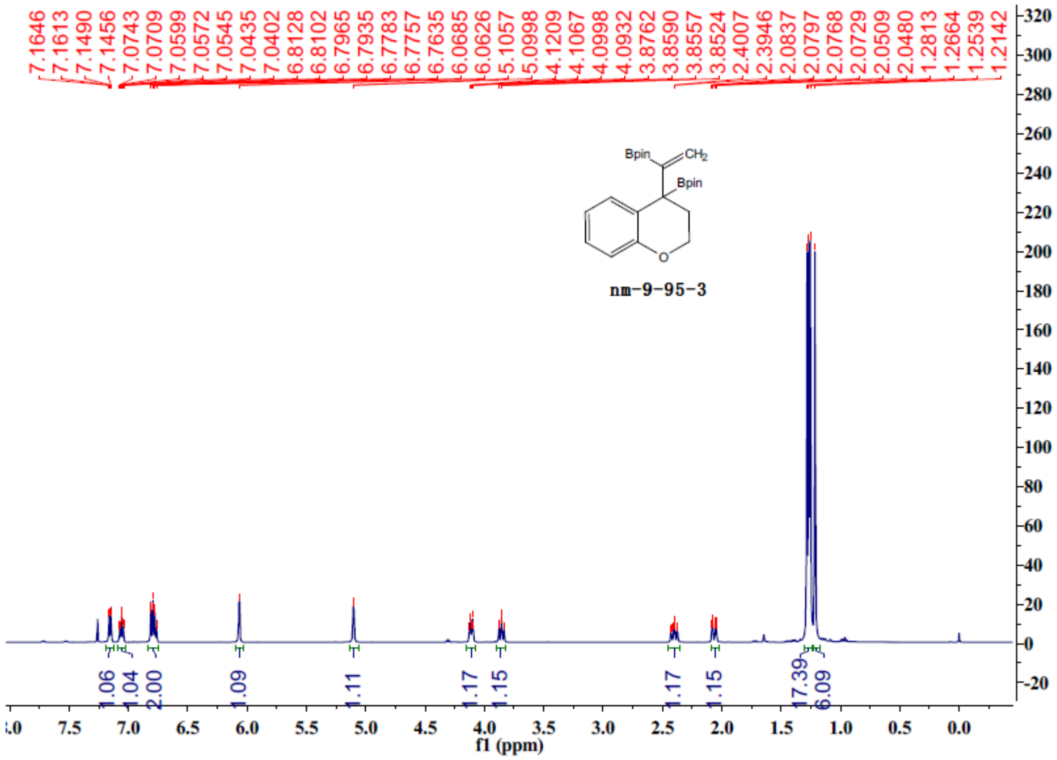

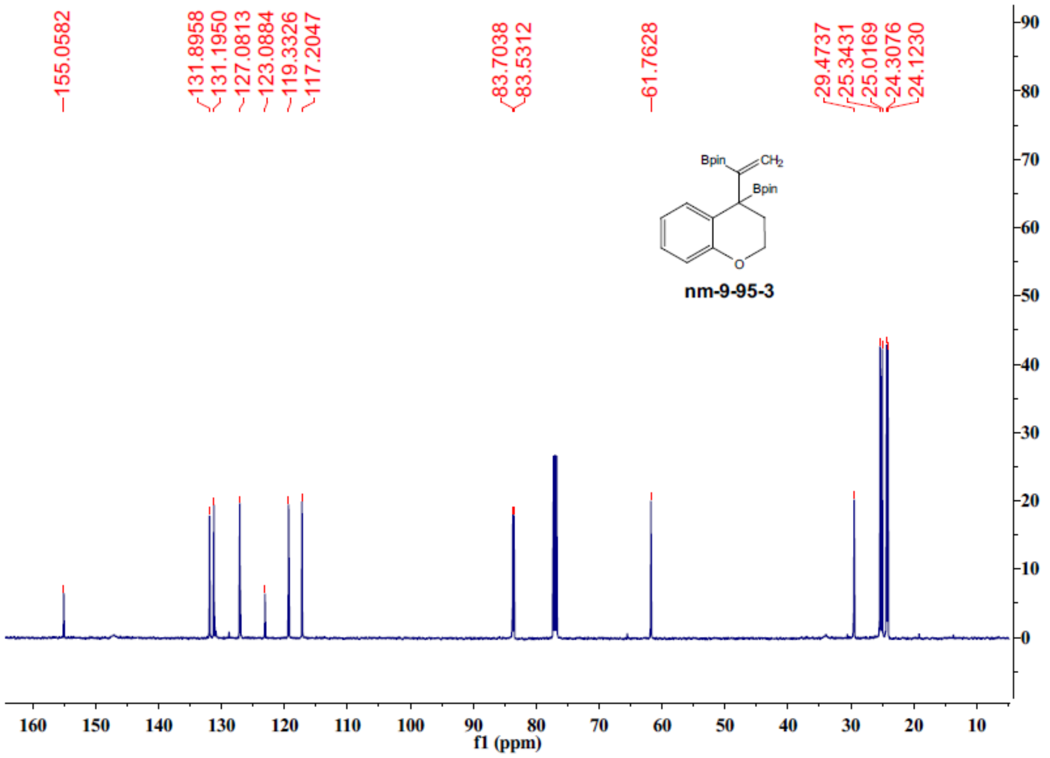
155.0582
131.8958
131.1950
127.0813
123.0884
119.3326
117.2047
83.7038
83.5312
61.7628
29.4737
25.3431
25.0169
24.3076
24.1230
Bpin
CH2
Bpin
O
nm-9-95-3
f1 (ppm)

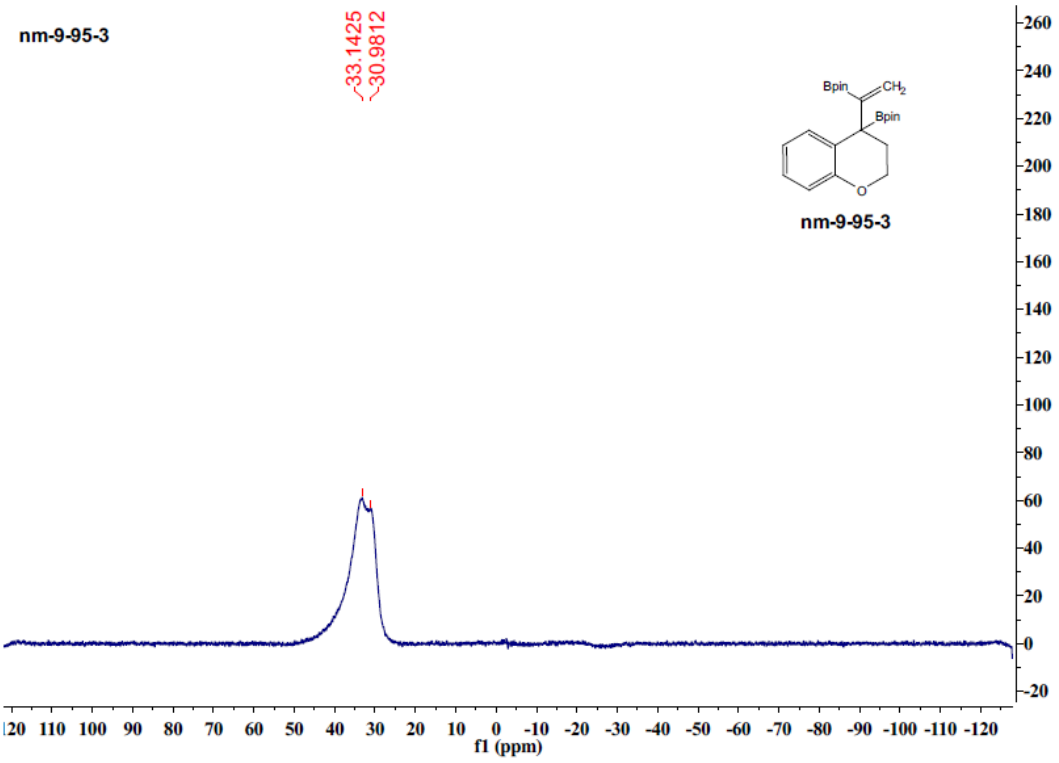
nm-9-95-3
33.1425
30.9812
Bpin
CH2
Bpin
O
nm-9-95-3
f1 (ppm)

NMR spectra of 3y

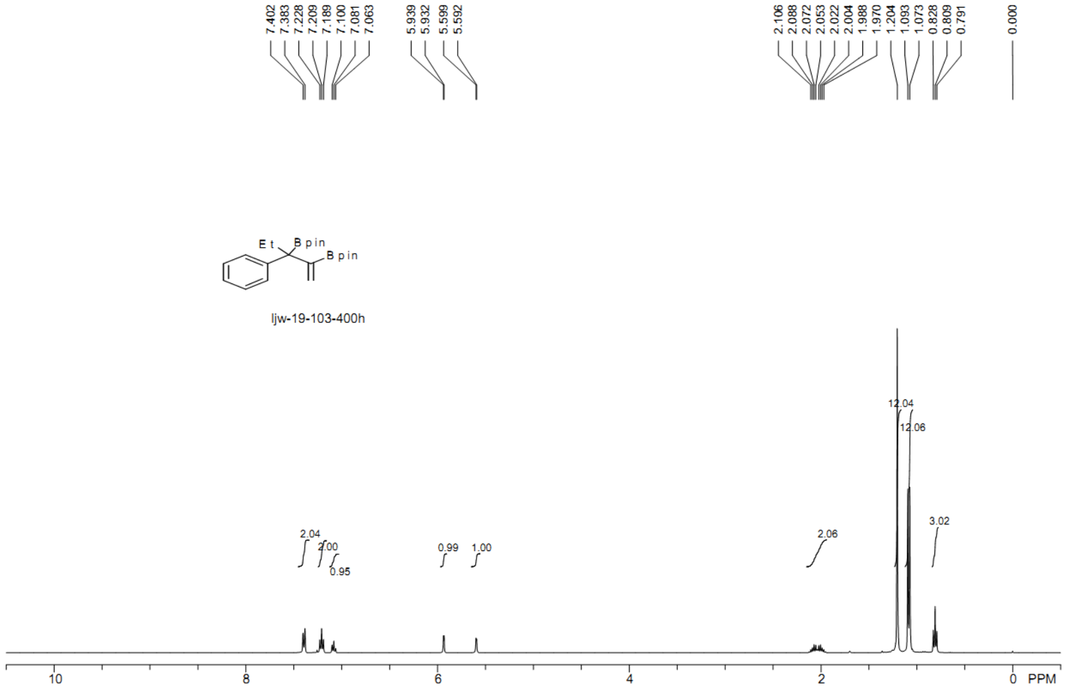

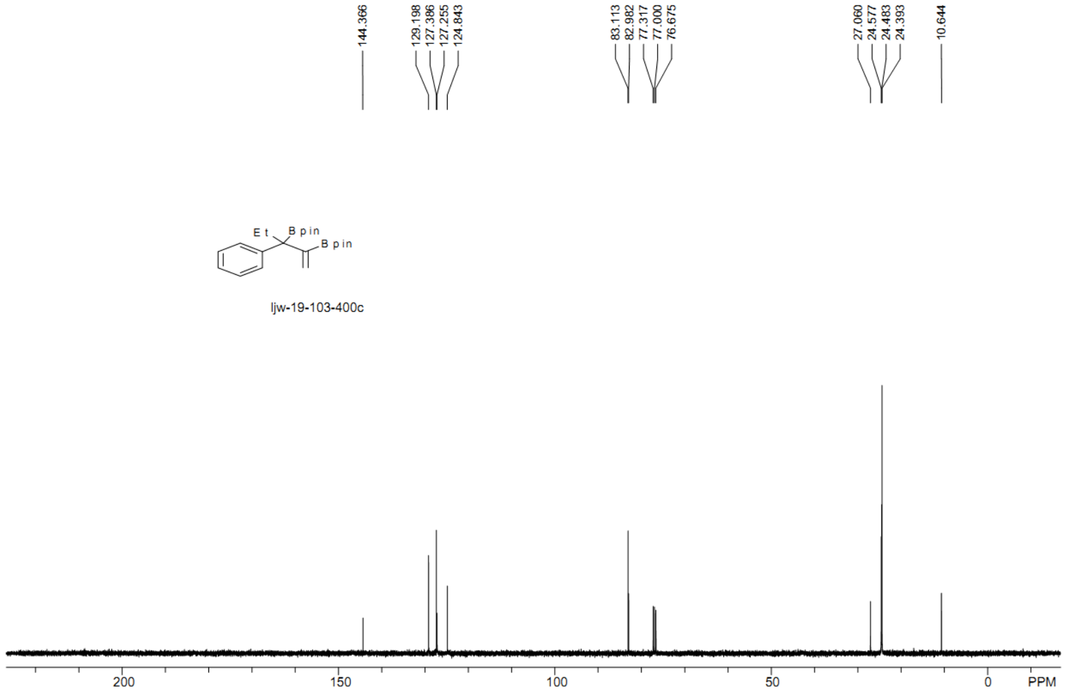

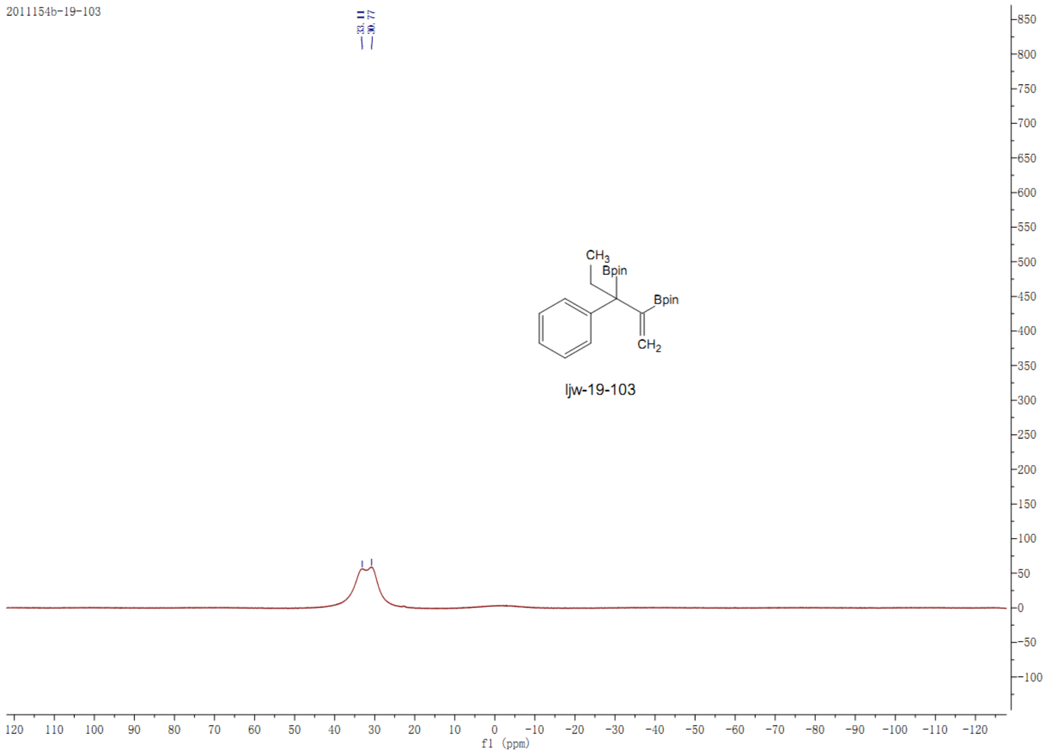

NMR spectra of 3z

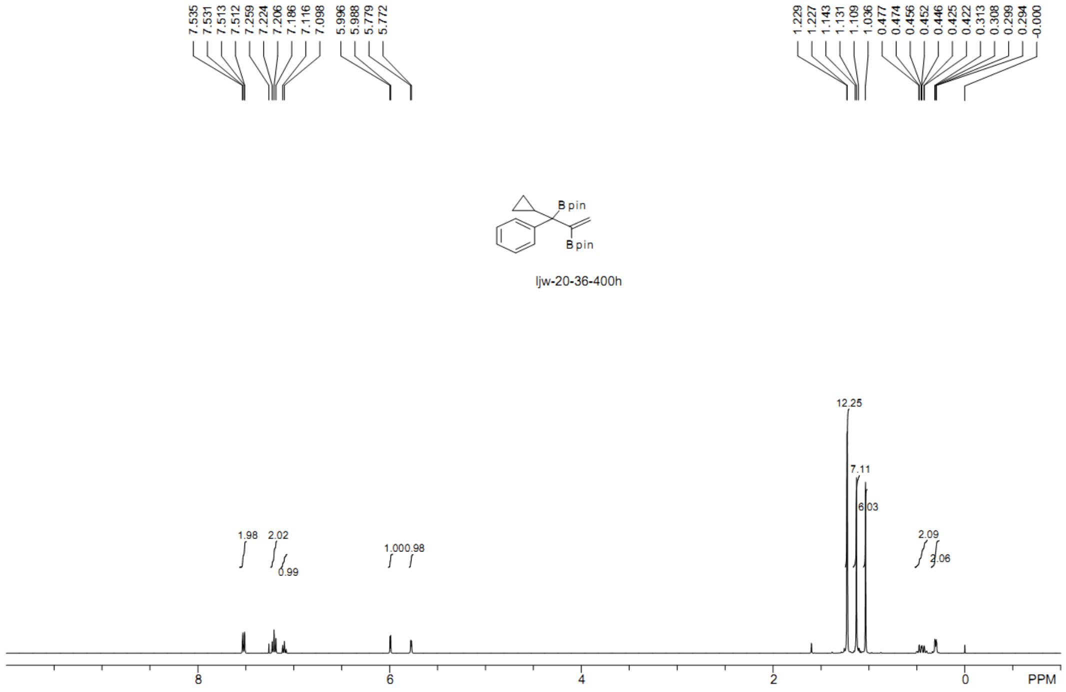

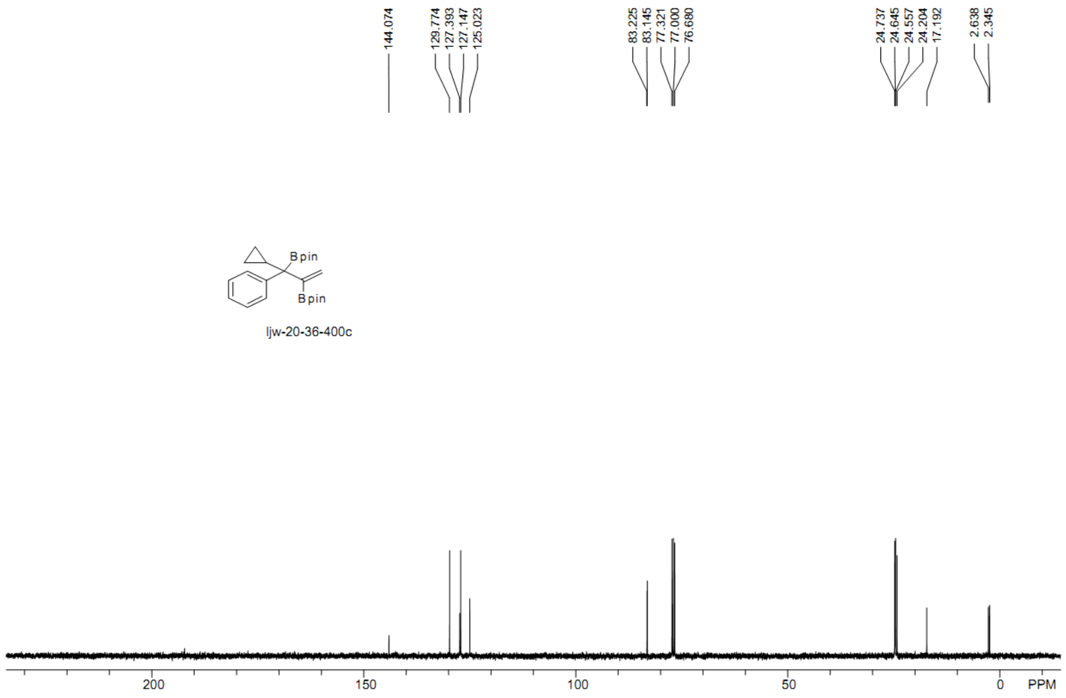
144.074
129.774
127.393
127.147
125.023
83.225
83.145
77.321
77.000
76.680
24.737
24.645
24.557
24.204
17.192
2.638
2.345
Bpin
Bpin
ljw-20-36-400c
200
150
100
50
0
PPM

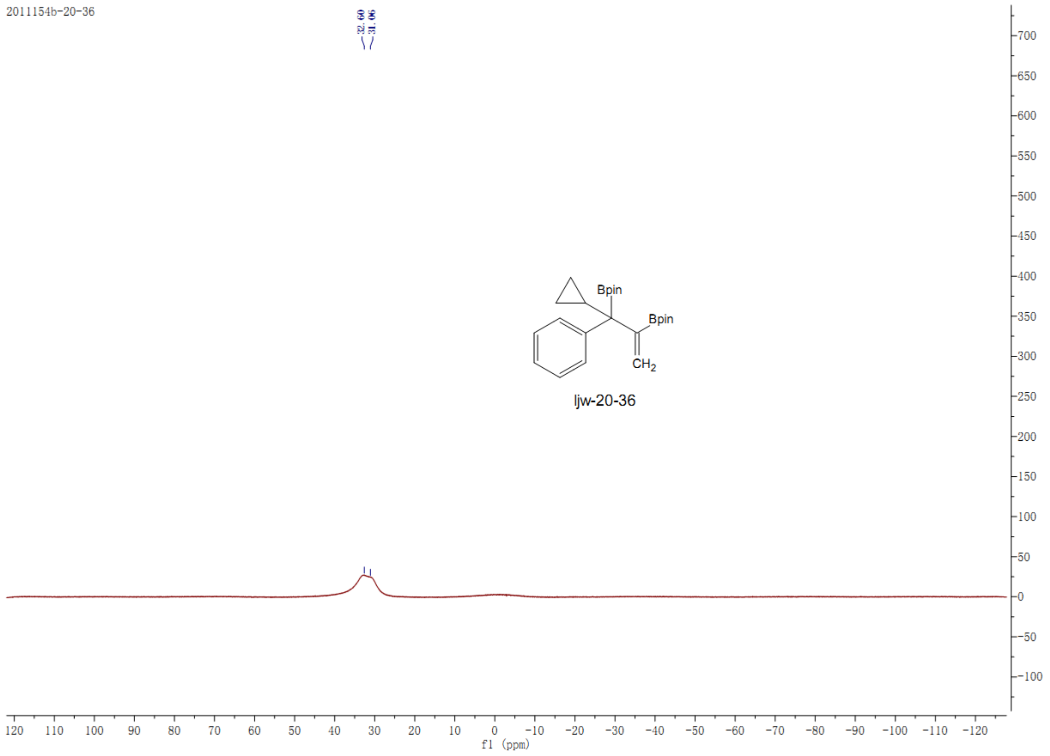
2011154b-20-36
Bpin
Bpin
CH2
ljw-20-36
f1 (ppm)

NMR spectra of 3aa

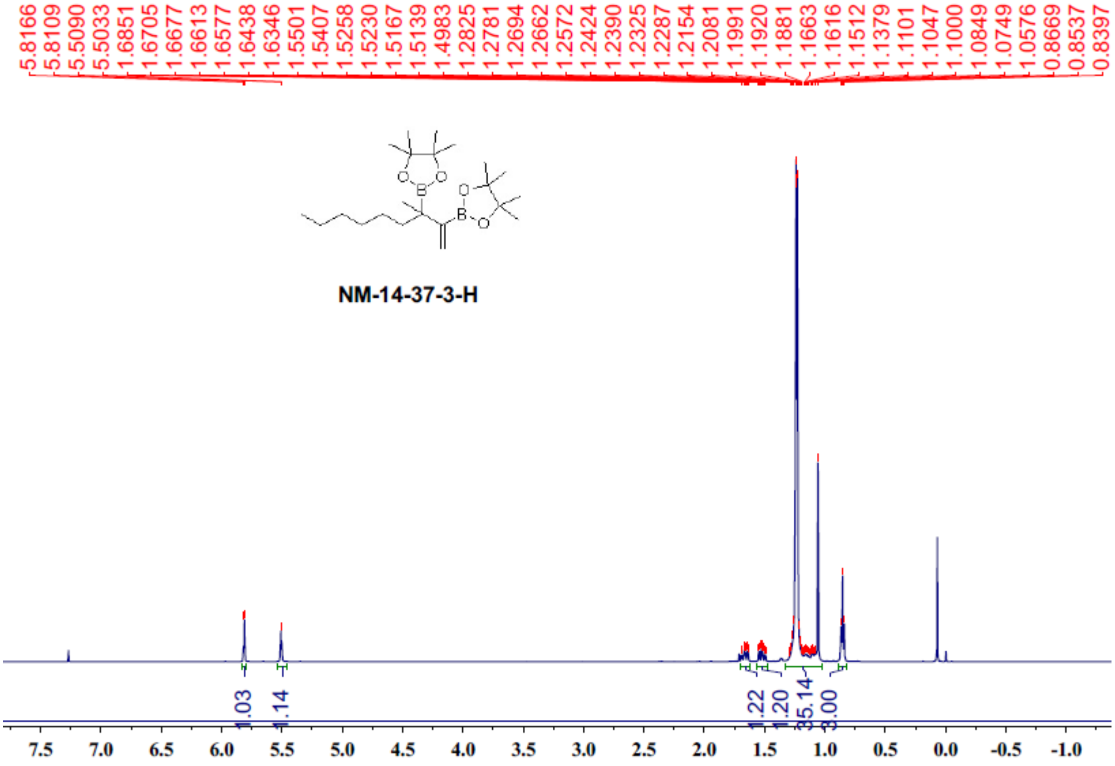

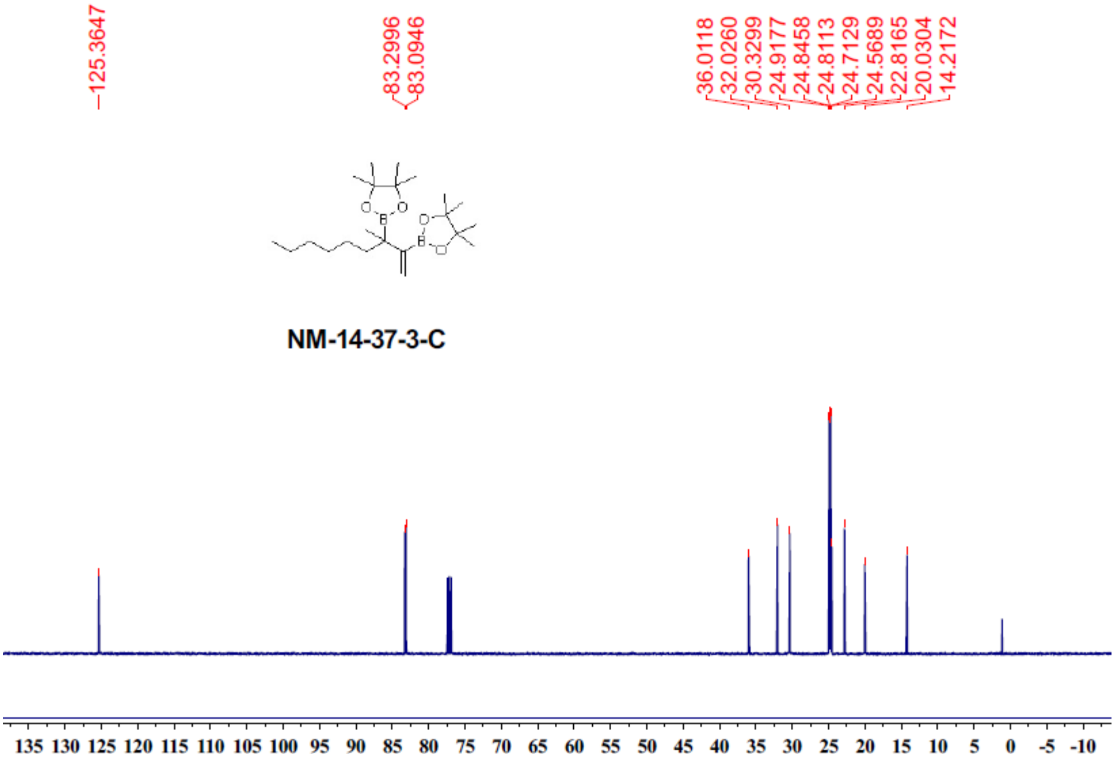

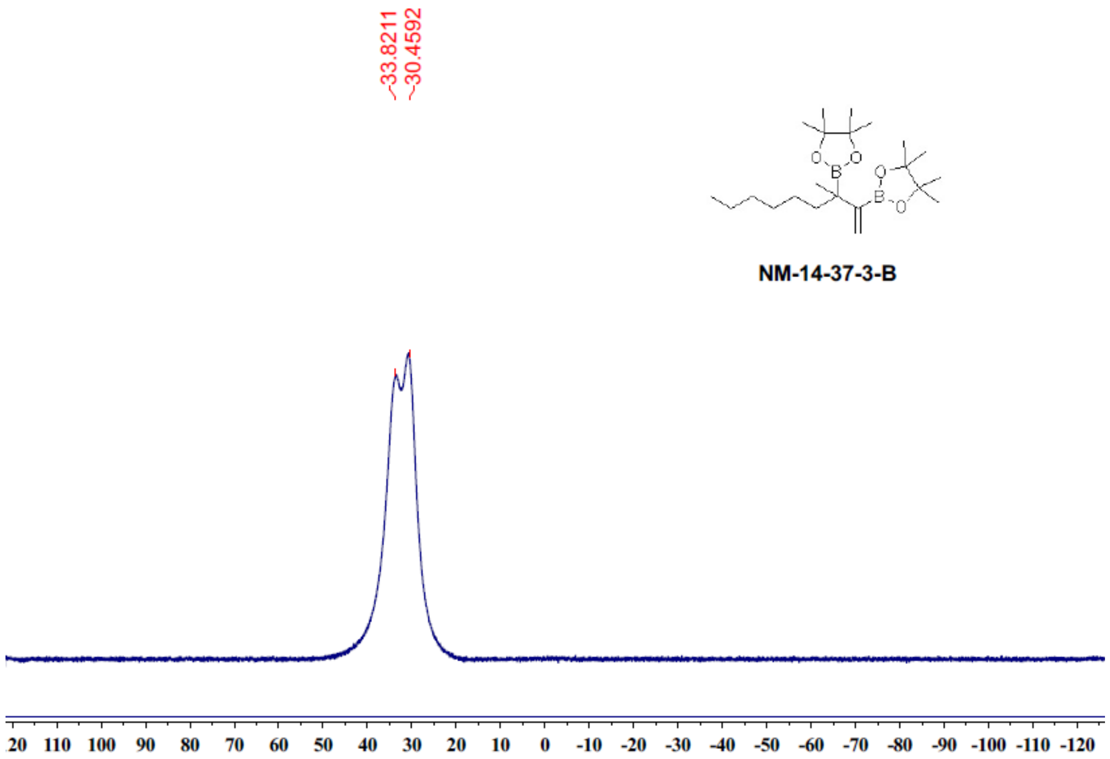

NMR spectra of 3ab

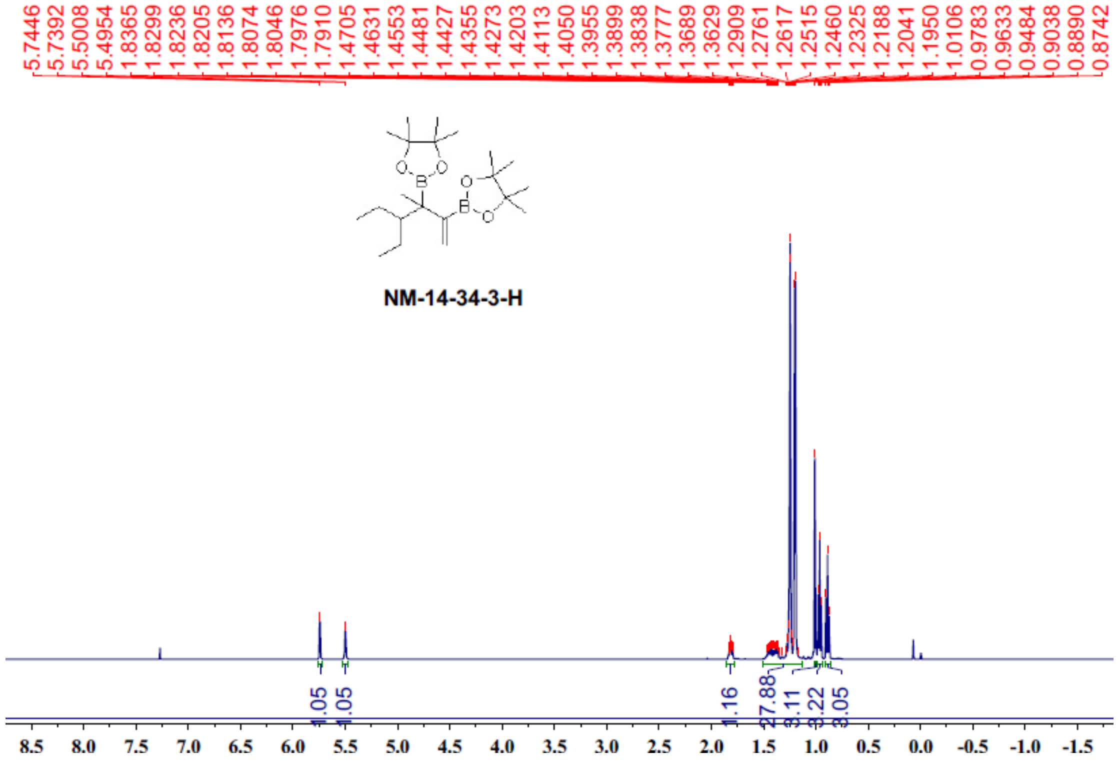

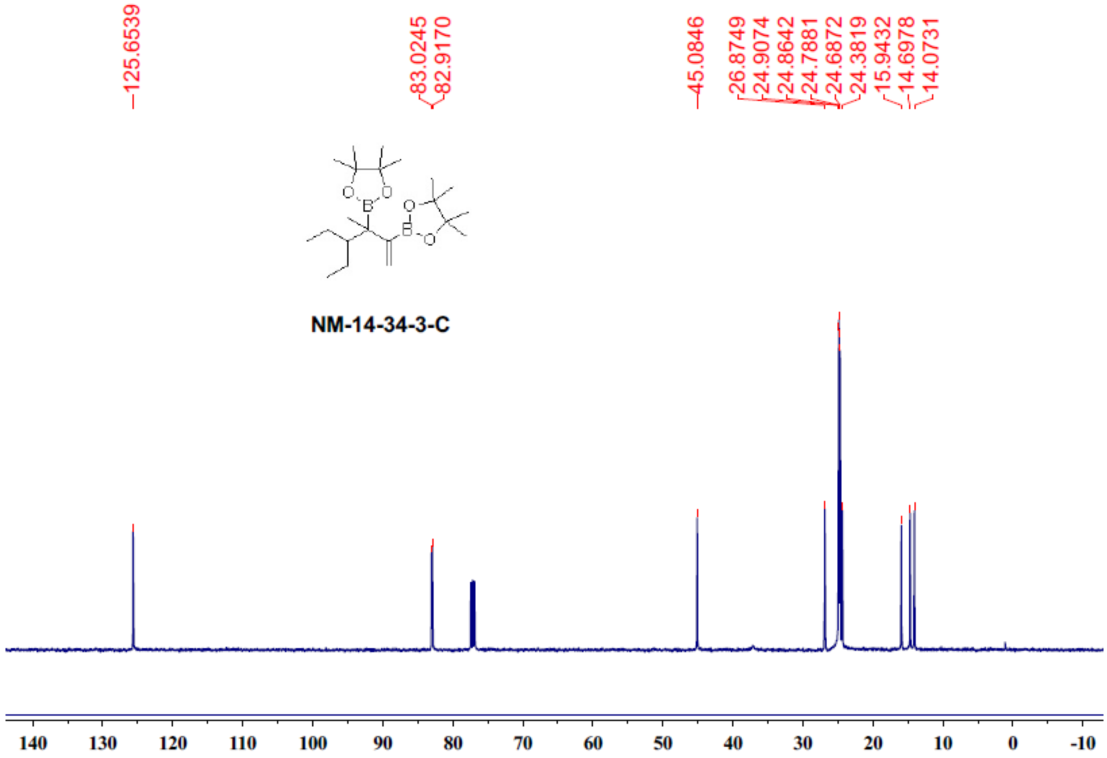

125.6539
83.0245
82.9170
45.0846
26.8749
24.9074
24.8642
24.7881
24.6872
24.3819
15.9432
14.6978
14.0731
NM-14-34-3-C
140 130 120 110 100 90 80 70 60 50 40 30 20 10 0 -10

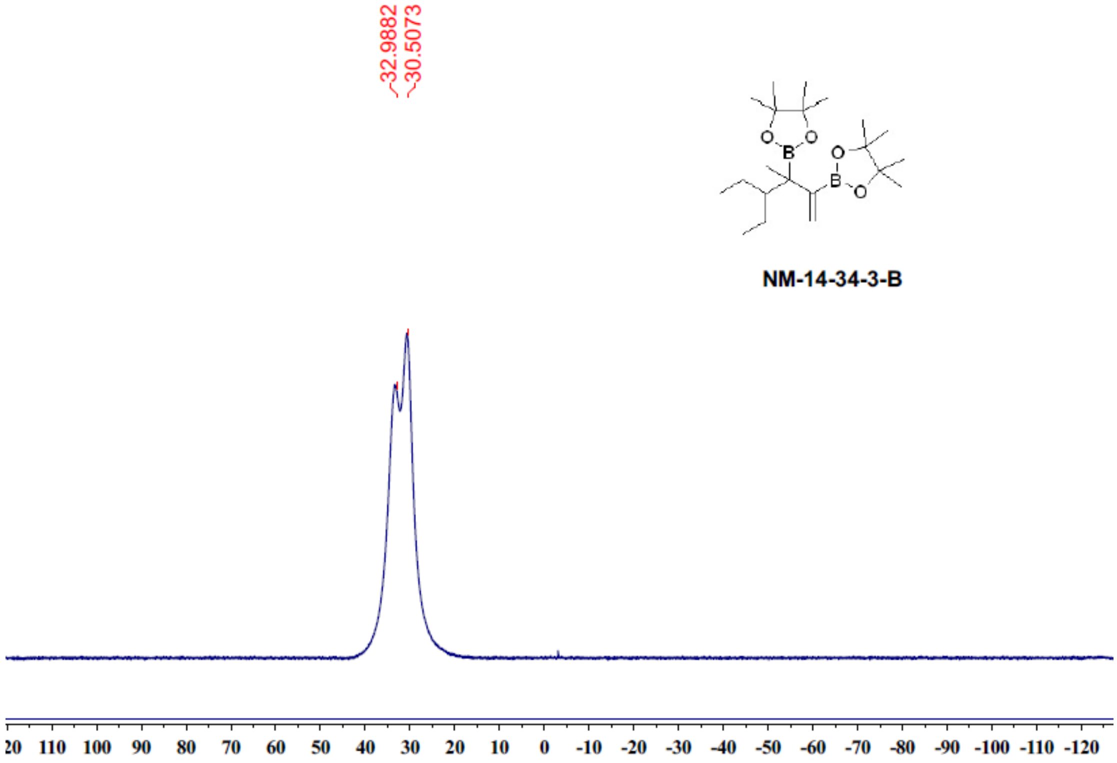

32.9882
30.5073
NM-14-34-3-B
20 110 100 90 80 70 60 50 40 30 20 10 0 -10 -20 -30 -40 -50 -60 -70 -80 -90 -100 -110 -120

NMR spectra of 3ac

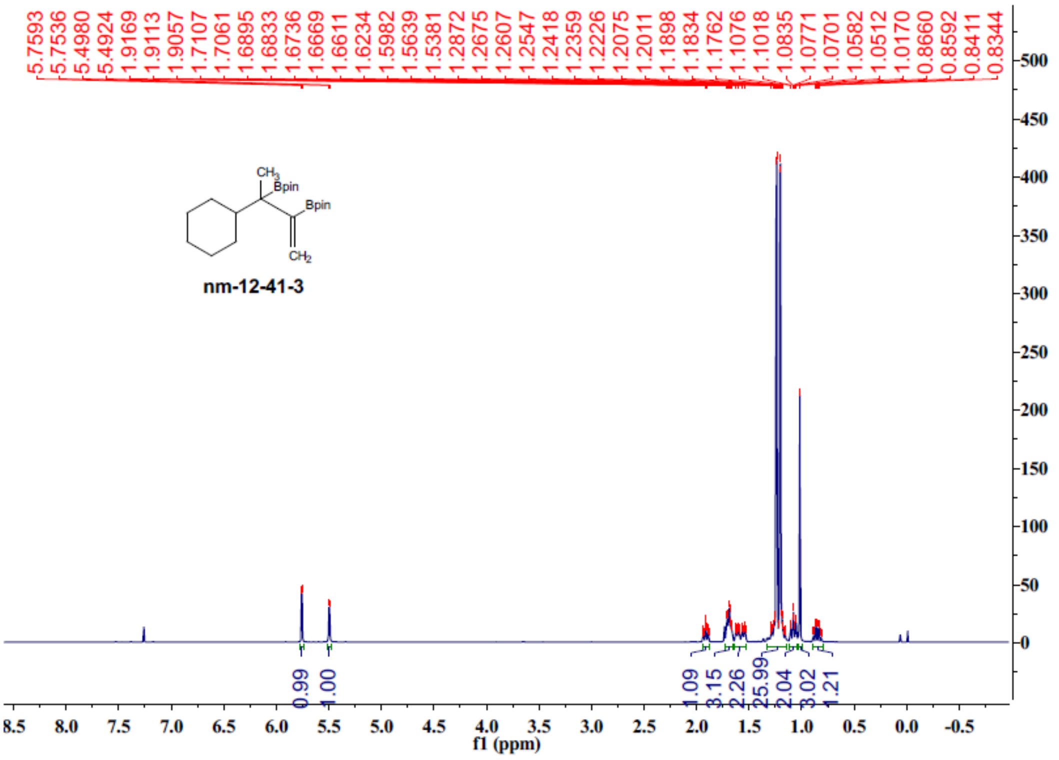

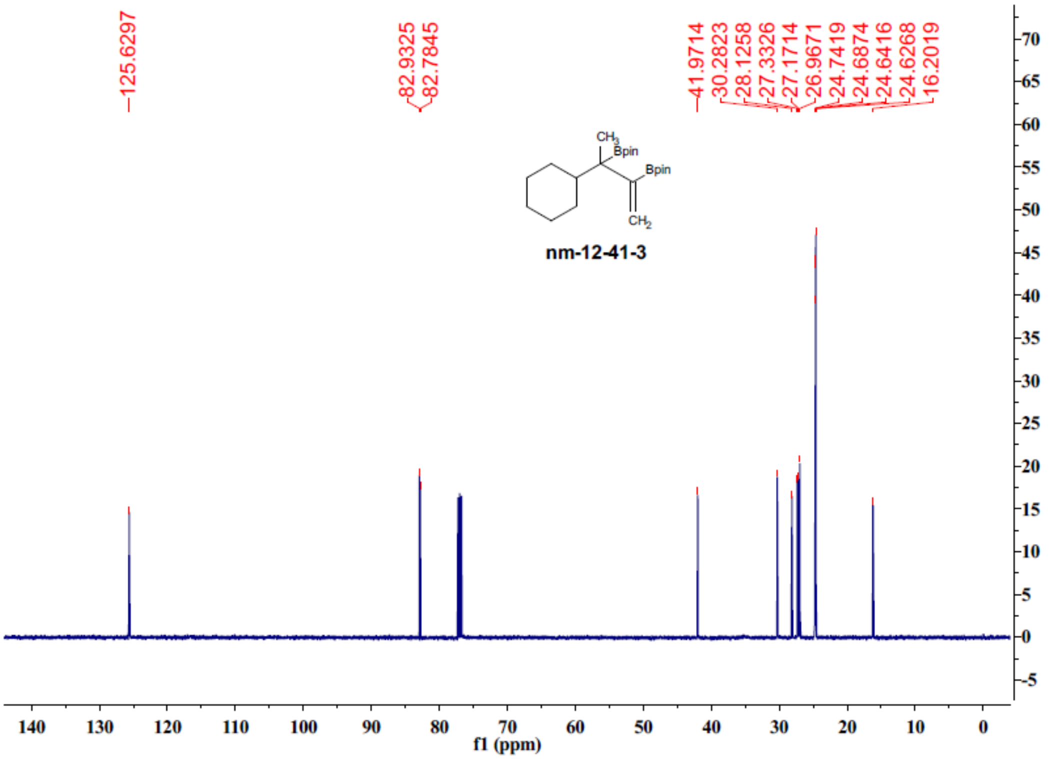

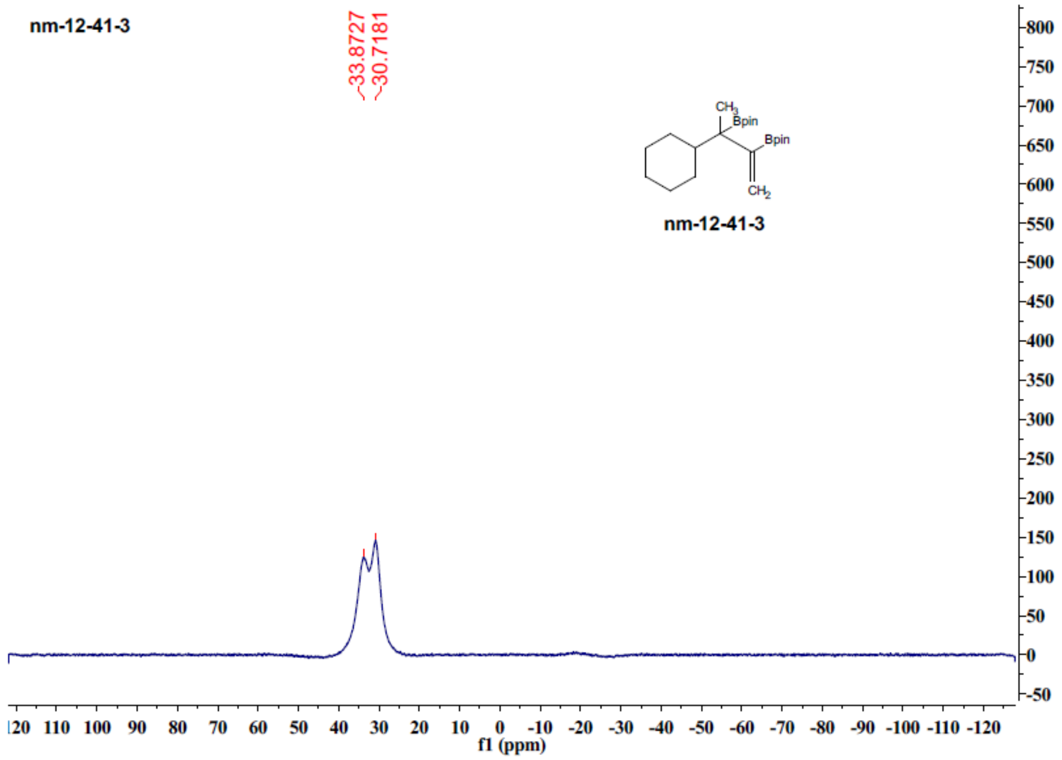

NMR spectra of 3ad

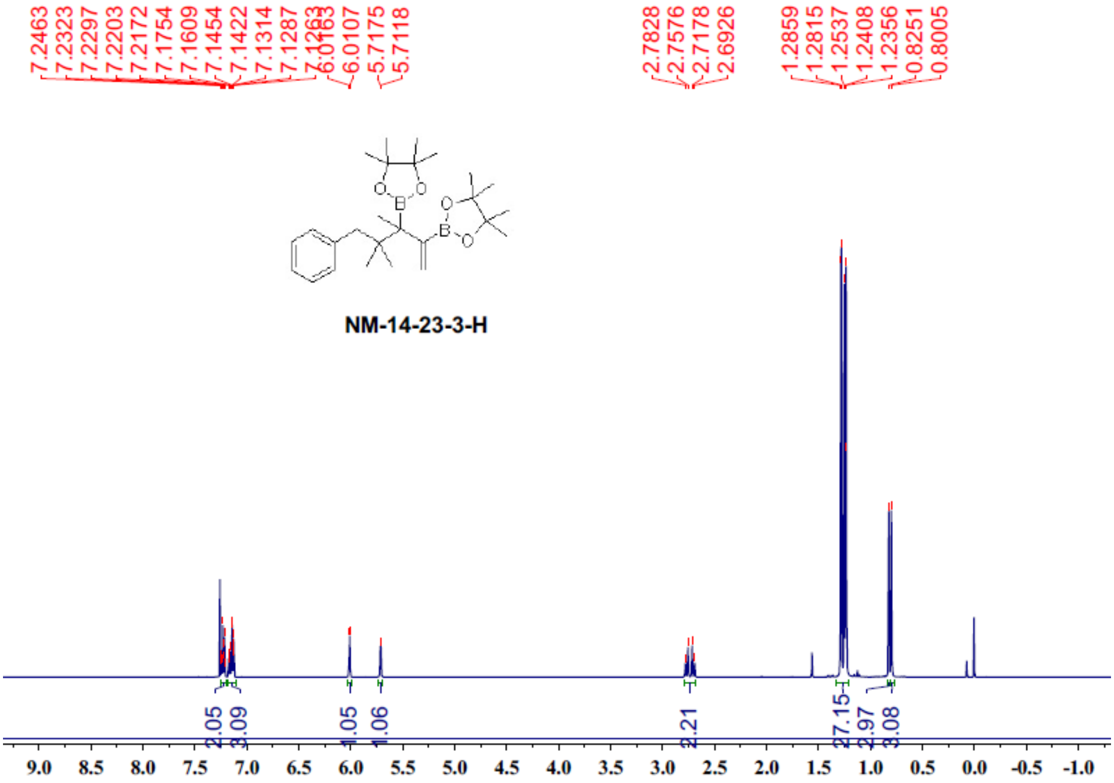

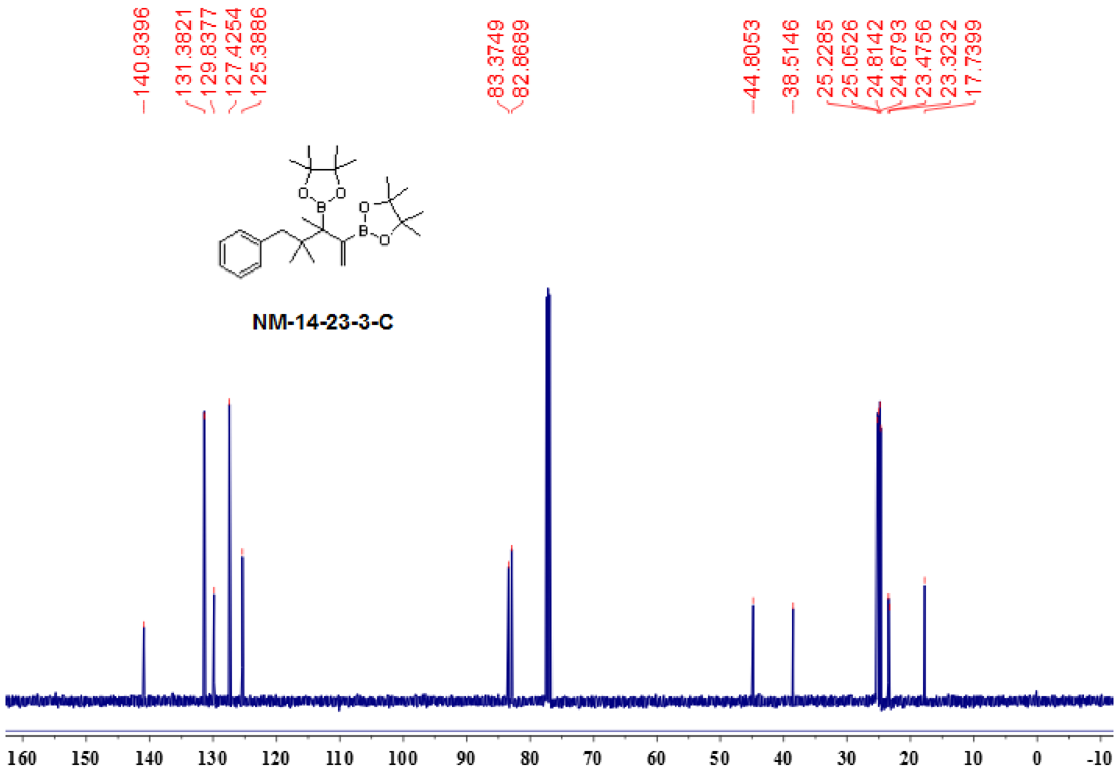
140.9396
131.3821
129.8377
127.4254
125.3886
83.3749
82.8689
44.8053
38.5146
25.2285
25.0526
24.8142
24.6793
23.4756
23.3232
17.7399
NM-14-23-3-C
160 150 140 130 120 110 100 90 80 70 60 50 40 30 20 10 0 -10

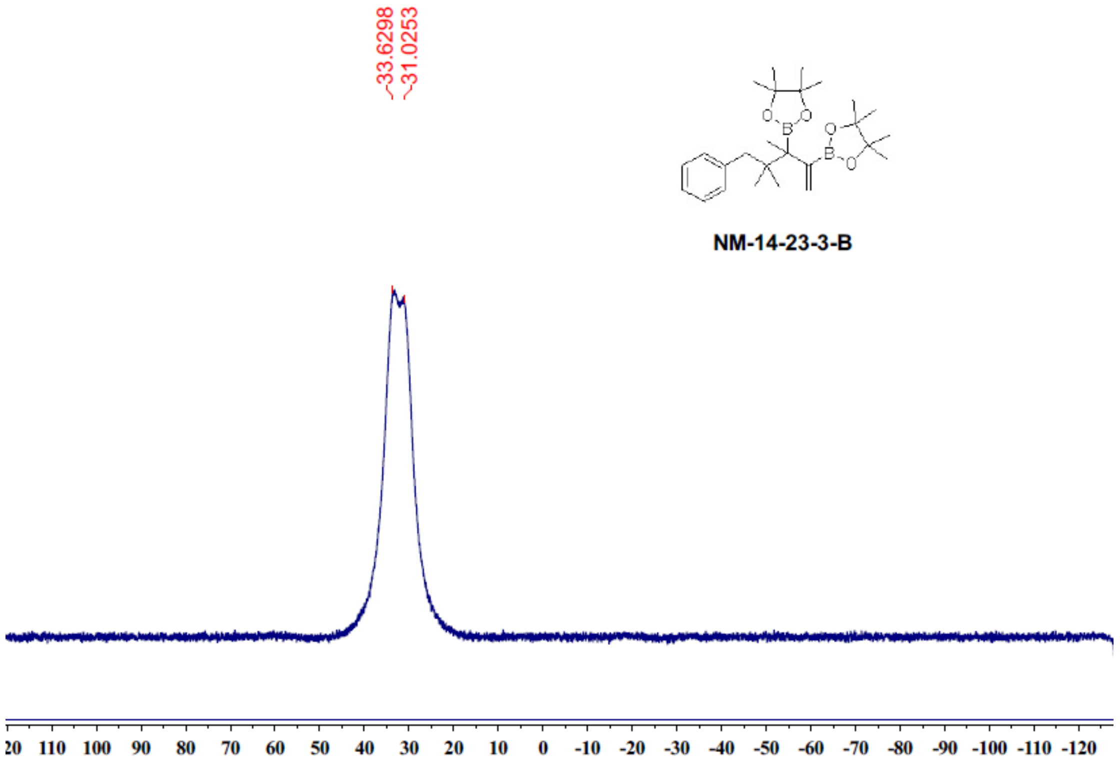
33.6298
31.0253
NM-14-23-3-B
20 110 100 90 80 70 60 50 40 30 20 10 0 -10 -20 -30 -40 -50 -60 -70 -80 -90 -100 -110 -120

NMR spectra of 3ae

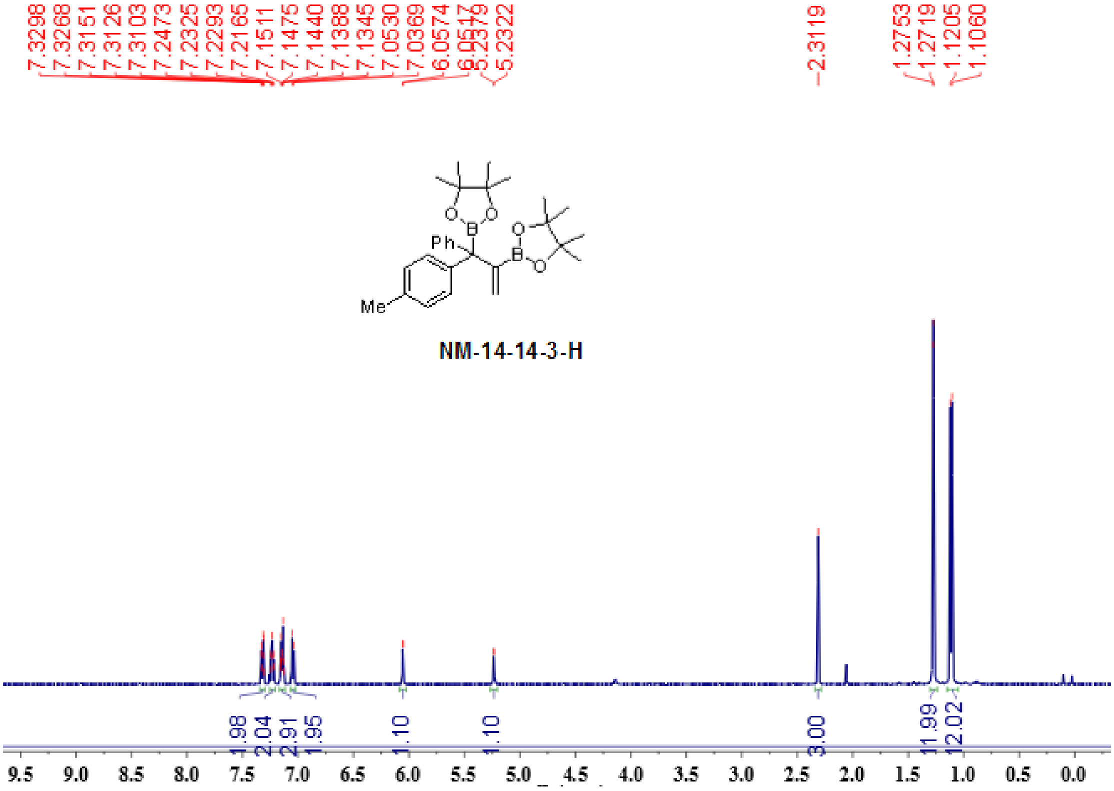

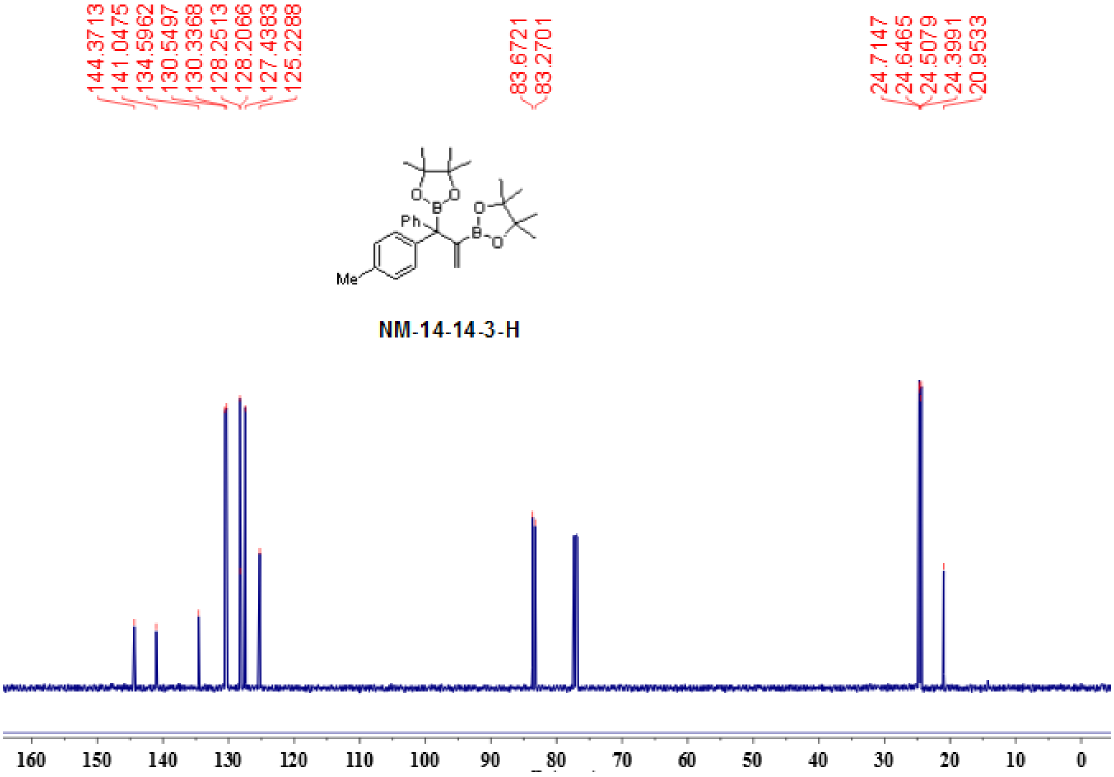

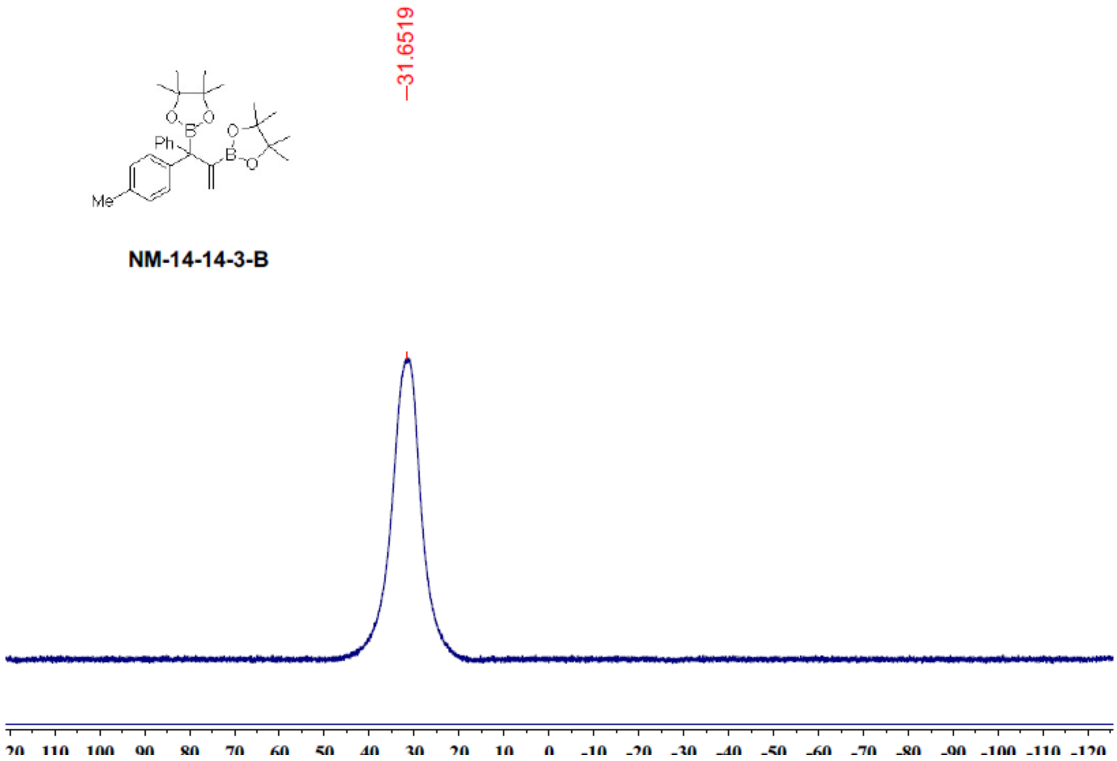

NMR spectra of 3af

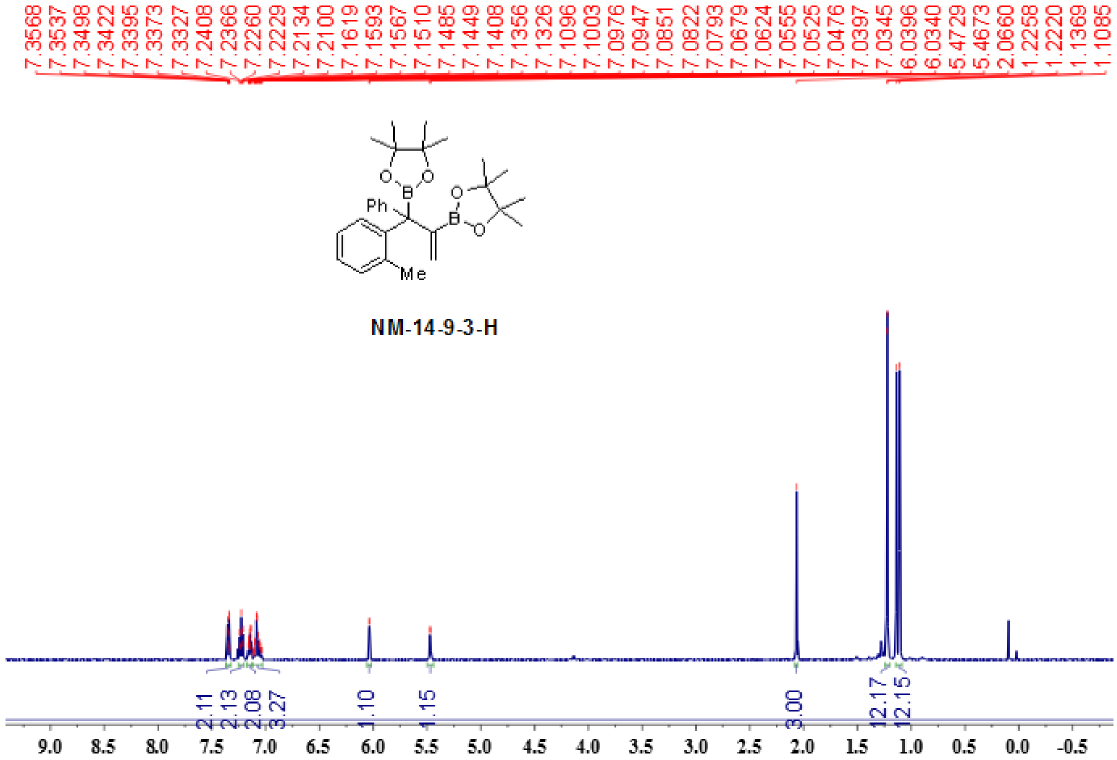

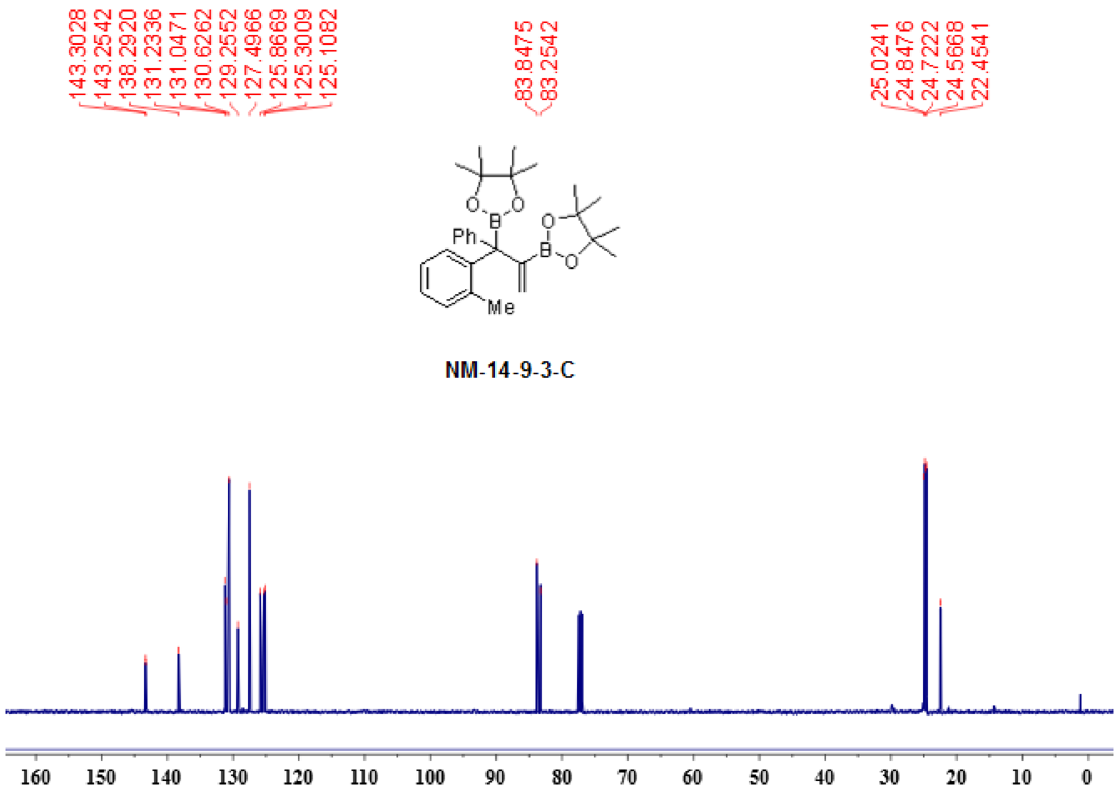
143.3028
143.2542
138.2920
131.2336
131.0471
130.6262
129.2552
127.4966
125.8669
125.3009
125.1082
83.8475
83.2542
25.0241
24.8476
24.7222
24.5668
22.4541
Ph
O
B
O
Me
NM-14-9-3-C
160 150 140 130 120 110 100 90 80 70 60 50 40 30 20 10 0

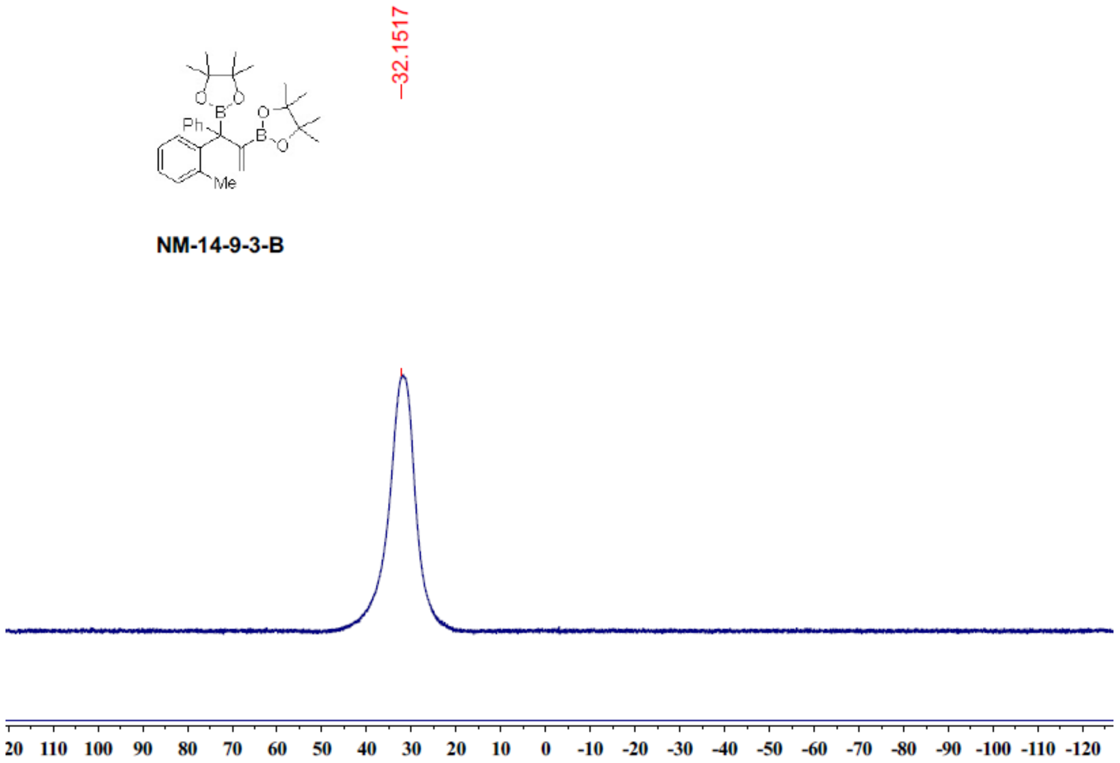
32.1517
Ph
O
B
O
Me
NM-14-9-3-B
20 110 100 90 80 70 60 50 40 30 20 10 0 -10 -20 -30 -40 -50 -60 -70 -80 -90 -100 -110 -120

NMR spectra of 4

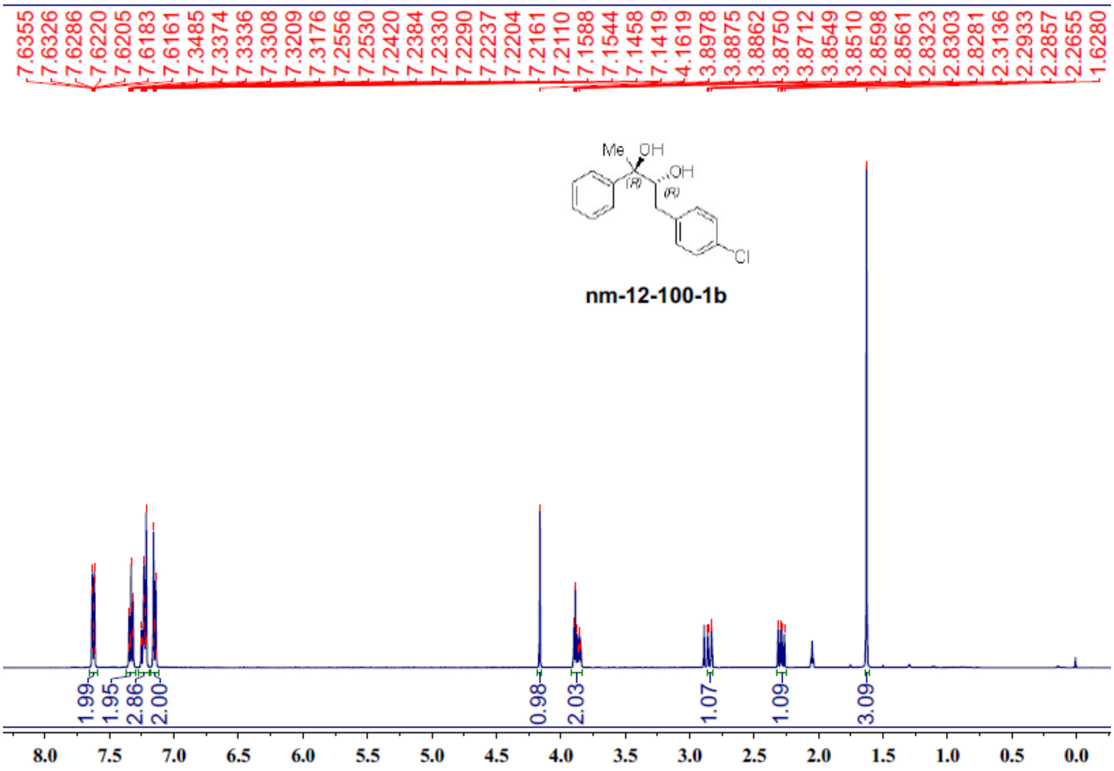

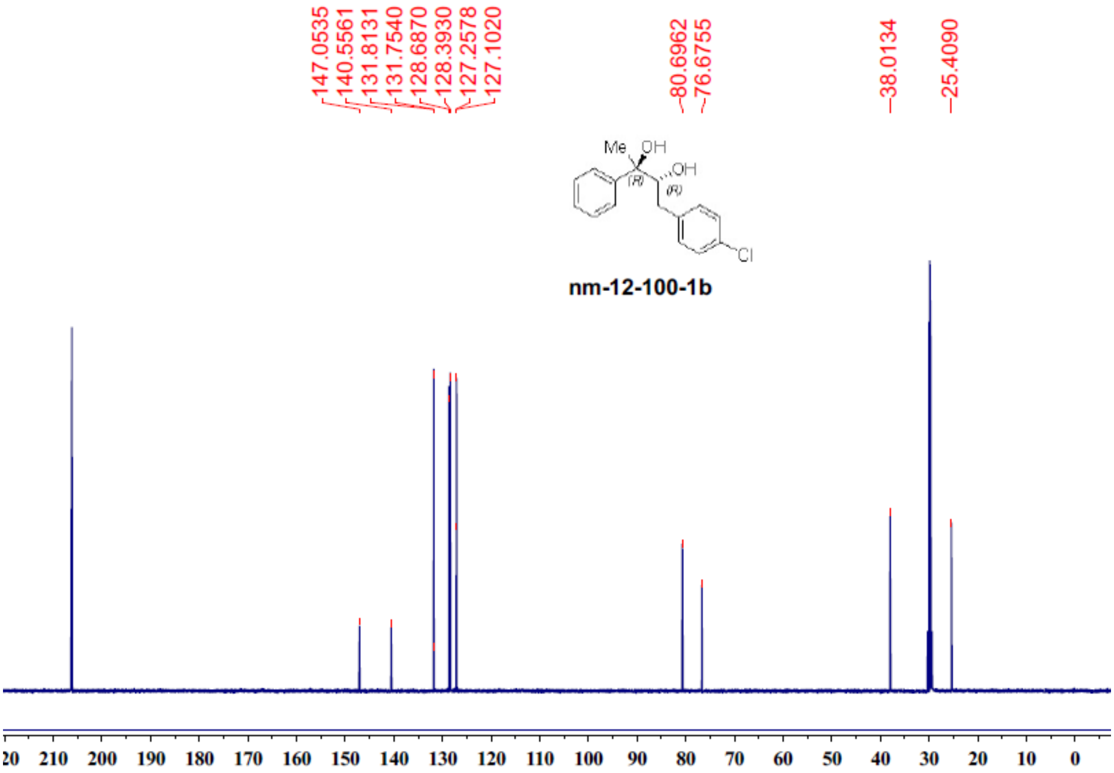

NMR spectra of 5

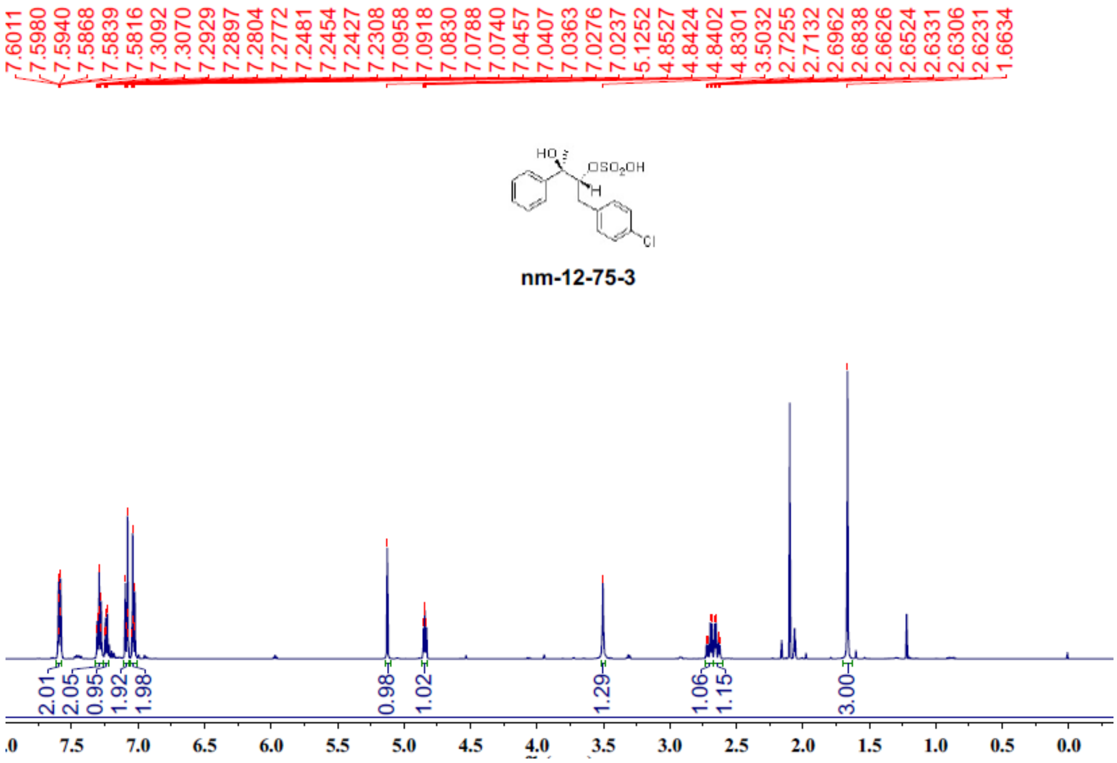

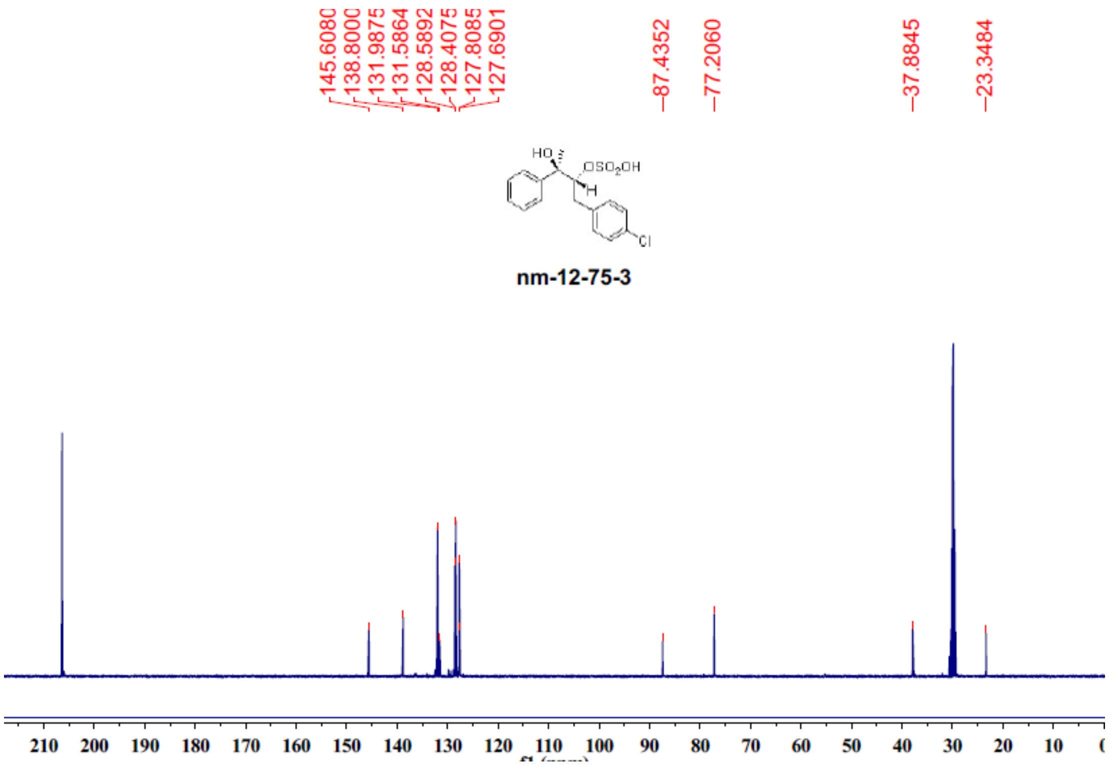

eNMR spectra of 6

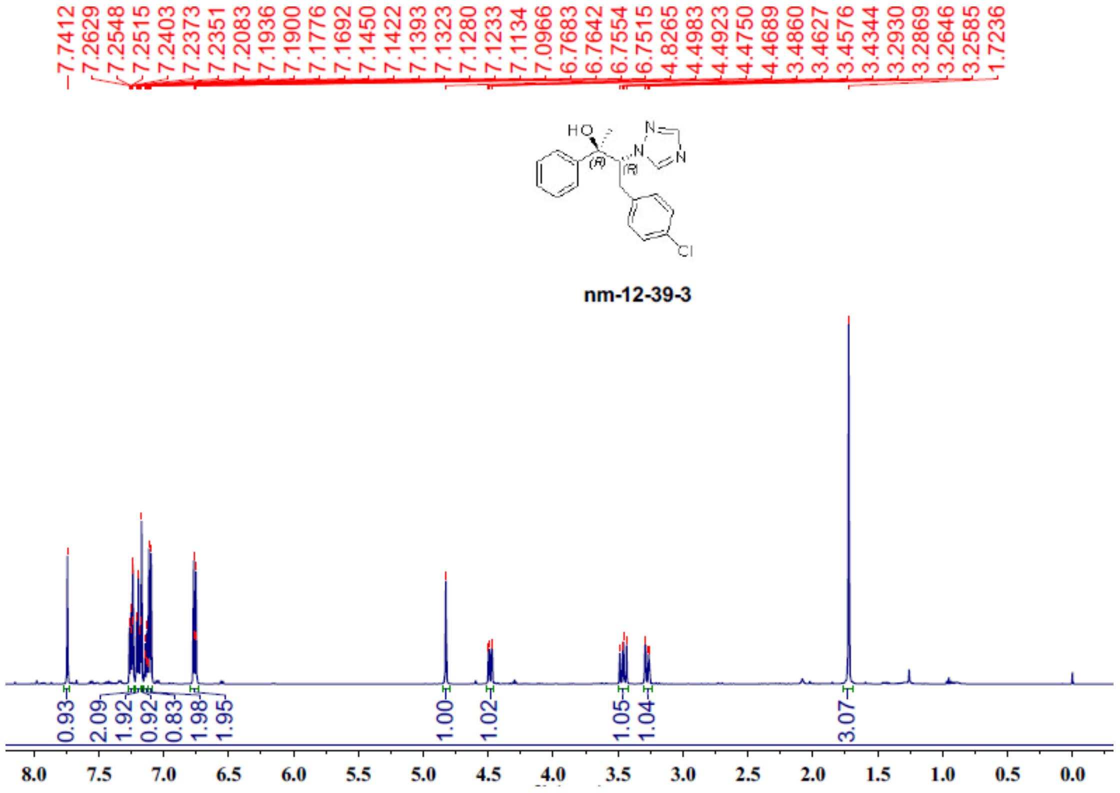

7.7412
7.2629
7.2548
7.2515
7.2403
7.2373
7.2351
7.2083
7.1936
7.1900
7.1776
7.1692
7.1450
7.1422
7.1393
7.1323
7.1280
7.1233
7.1134
7.0966
6.7683
6.7642
6.7554
6.7515
4.8265
4.4983
4.4923
4.4750
4.4689
3.4860
3.4627
3.4576
3.4344
3.2930
3.2869
3.2646
3.2585
1.7236
nm-12-39-3
0.93
2.09
1.92
0.92
0.83
1.98
1.95
1.00
1.02
1.05
1.04
3.07
8.0 7.5 7.0 6.5 6.0 5.5 5.0 4.5 4.0 3.5 3.0 2.5 2.0 1.5 1.0 0.5 0.0

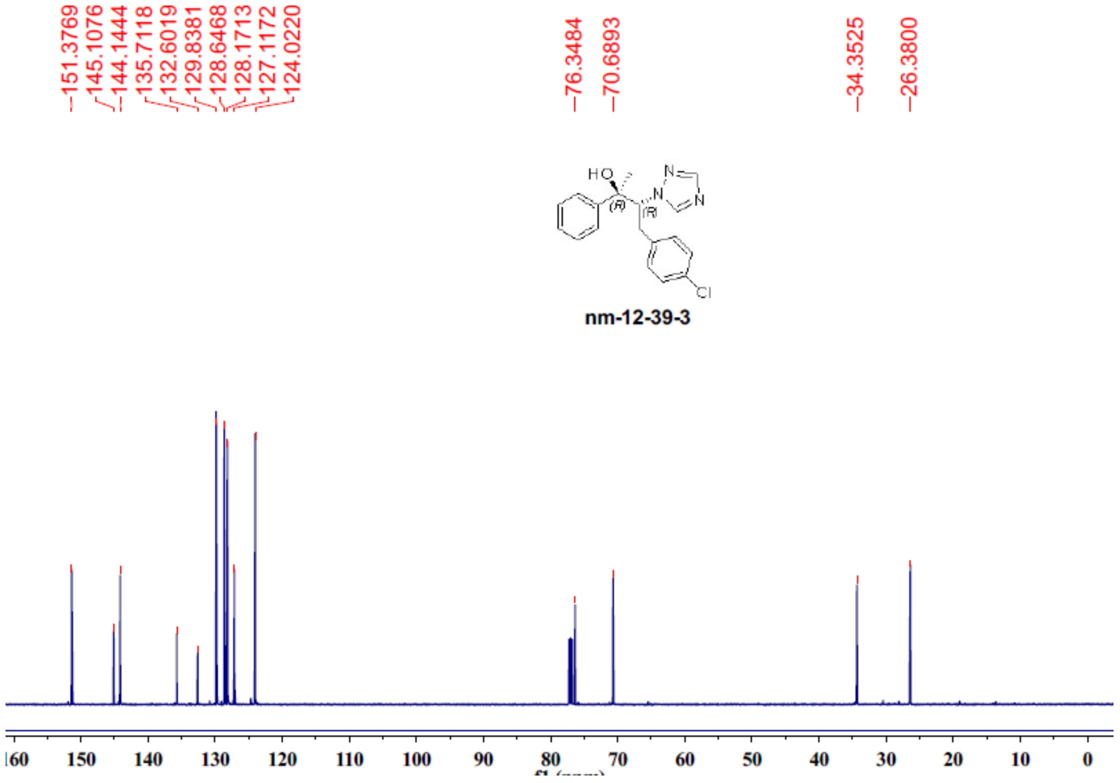

151.3769
145.1076
144.1444
135.7118
132.6019
129.8381
128.6468
128.1713
127.1172
124.0220
76.3484
70.6893
34.3525
26.3800
nm-12-39-3
160 150 140 130 120 110 100 90 80 70 60 50 40 30 20 10 0

NMR spectra of 7

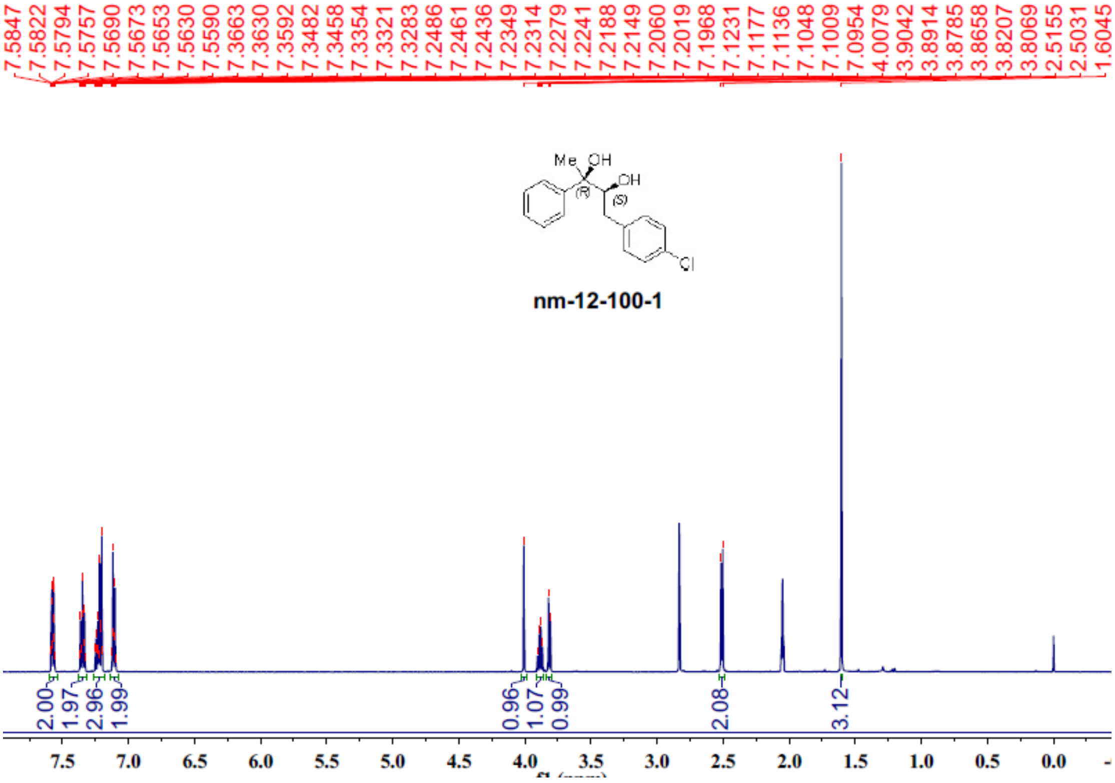

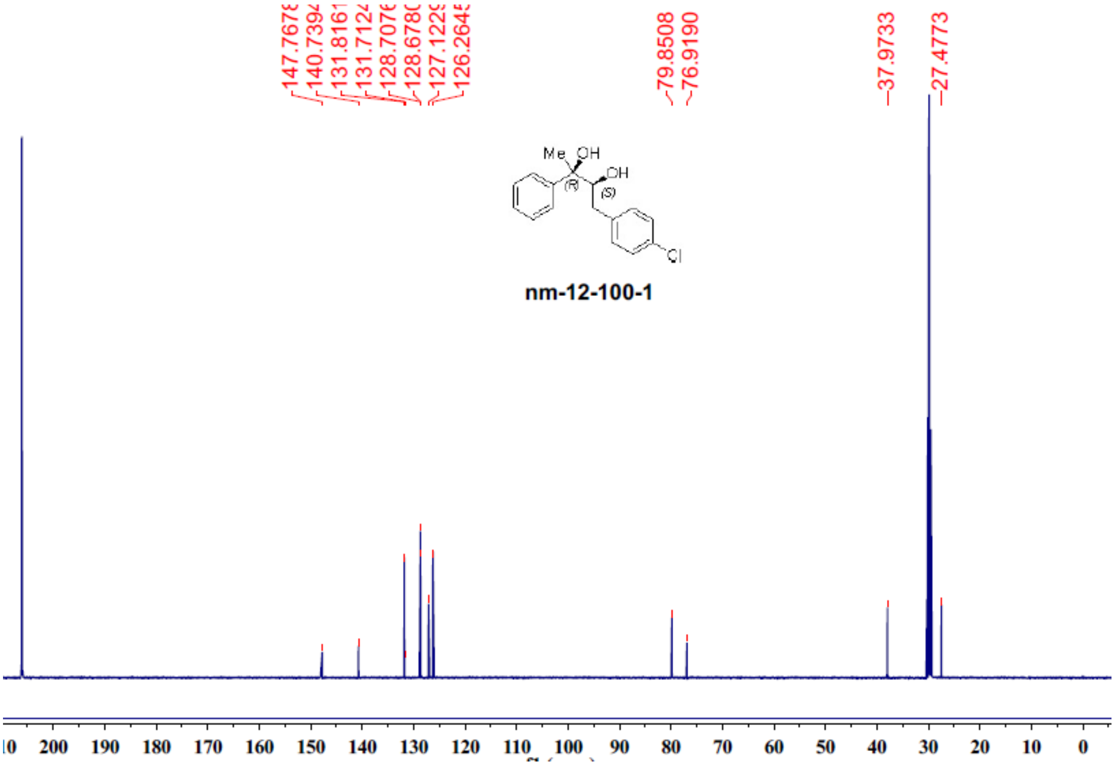

NMR spectra of 8

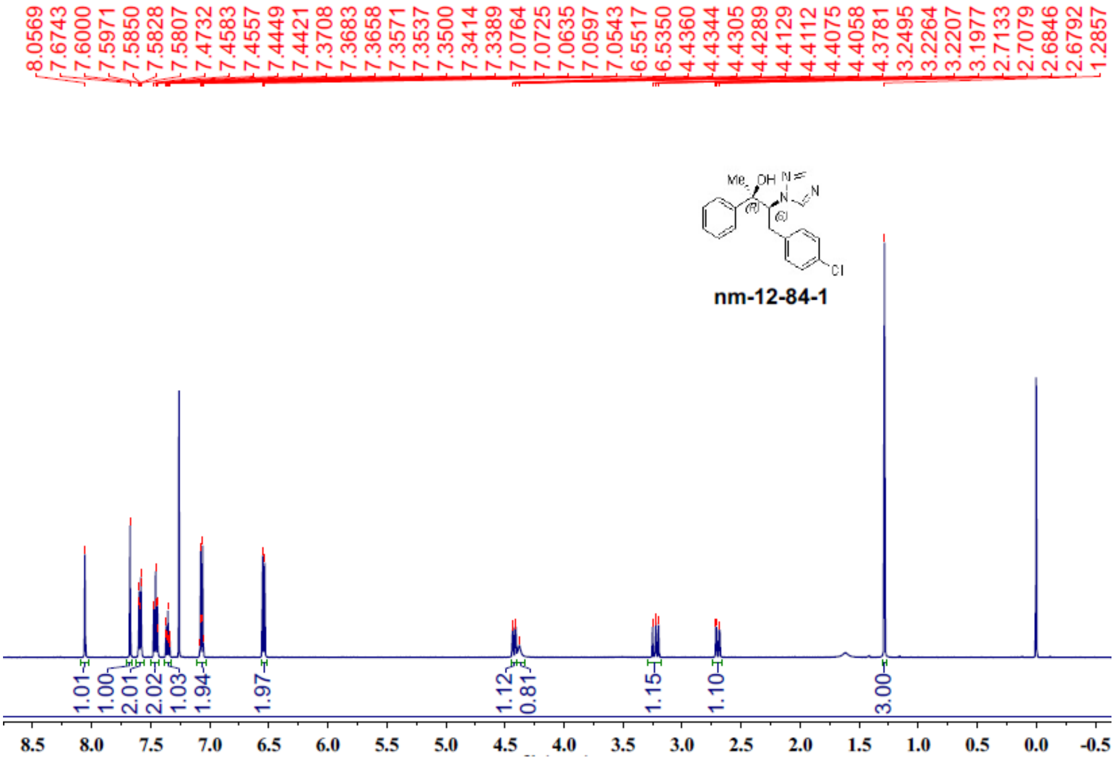

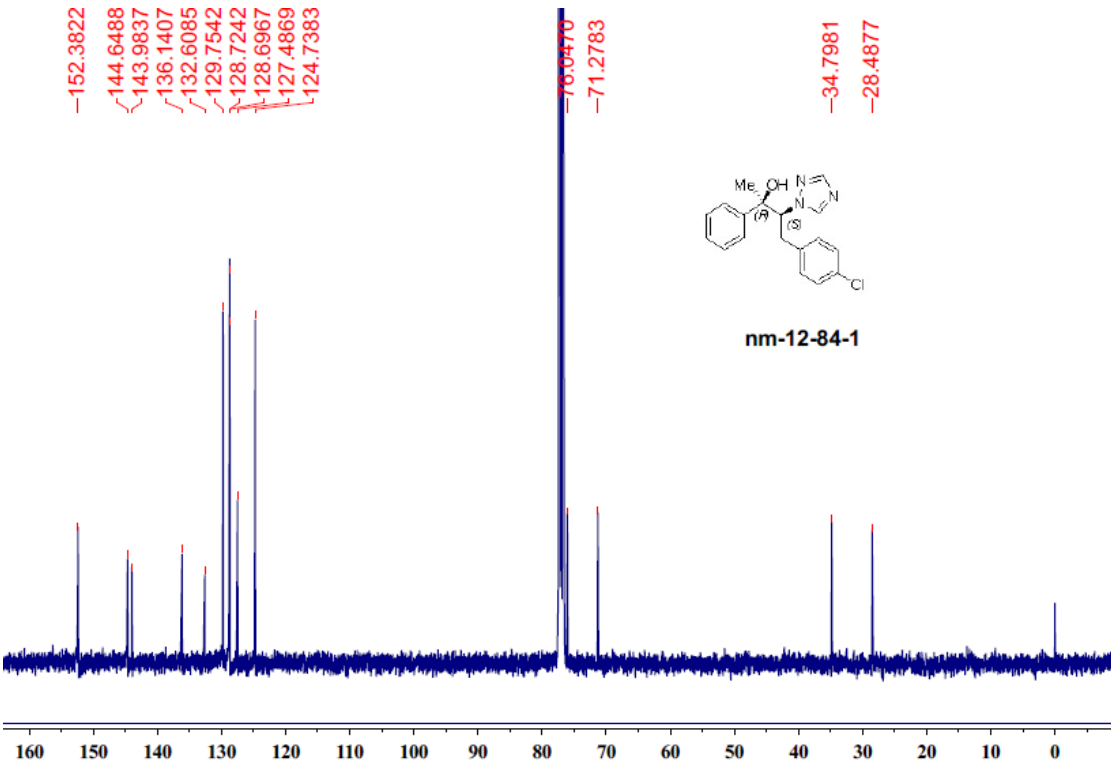

HPLC Chromatograms

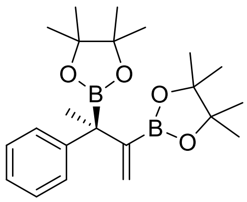

3a

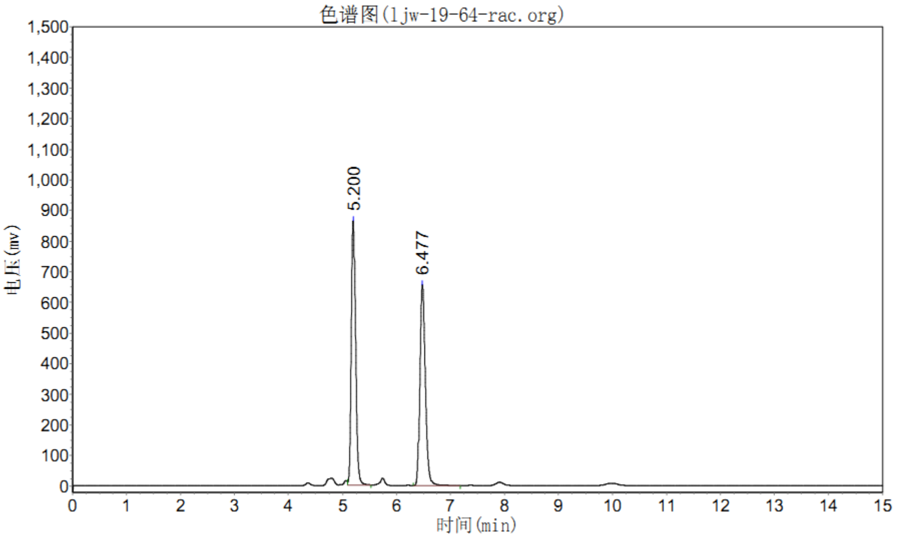

峰号	峰名	保留时间	峰高	峰面积	含量
1		5.200	862812.938	4594880.500	49.3899
2		6.477	656502.313	4708407.500	50.6101
总计			1519315.250	9303288.000	100.0000

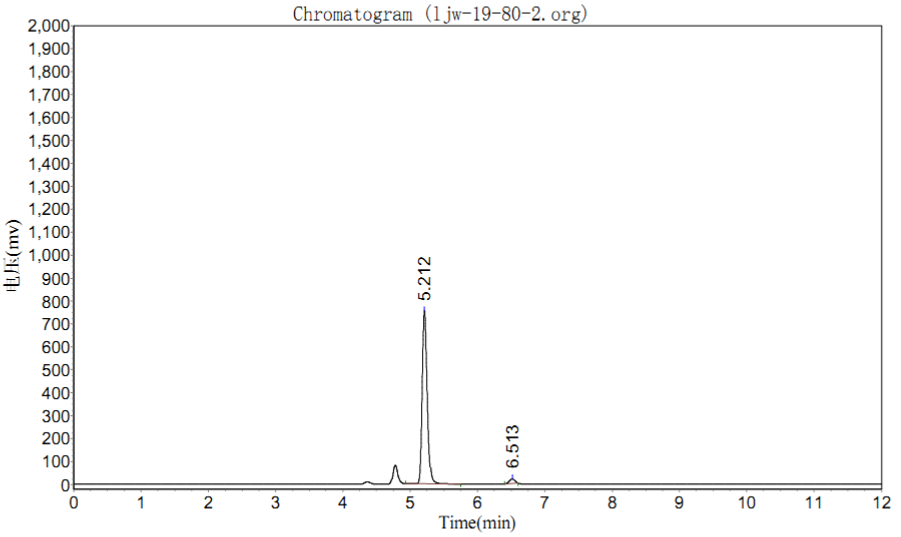

Peak No.	Peak ID	Ret Time	Height	Area	Conc.
1		5.212	754755.125	4042411.250	96.9931
2		6.513	20941.803	125319.508	3.0069
Total			775696.928	4167730.758	100.0000

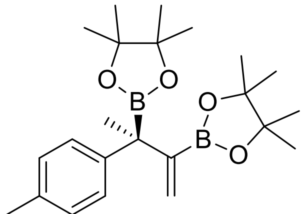

3b

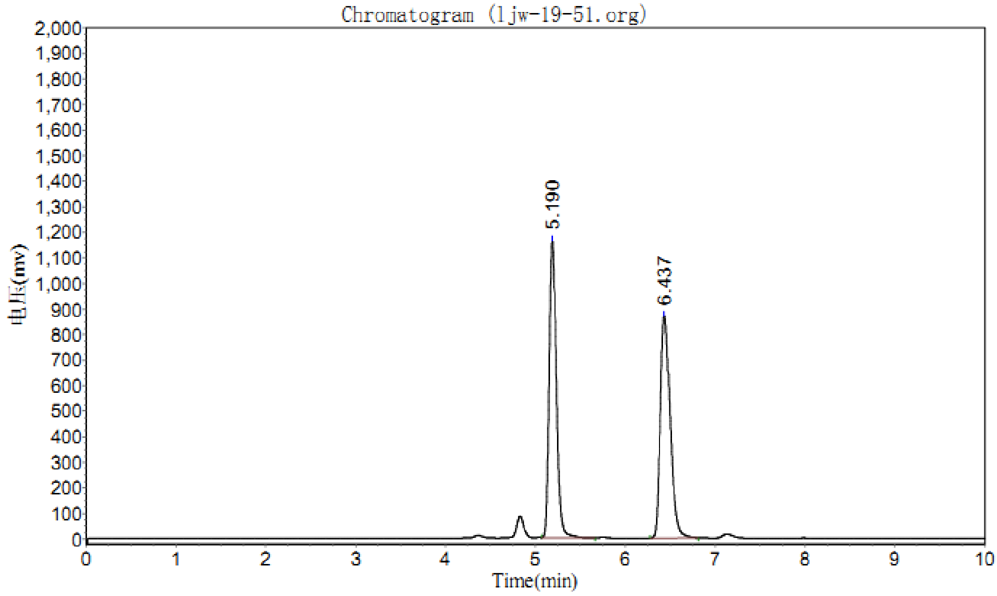

Peak No.	Peak ID	Ret Time	Height	Area	Conc.
1		5.190	1163921.625	6532170.500	49.3456
2		6.437	870803.375	6705427.000	50.6544
Total			2034725.000	13237597.500	100.0000

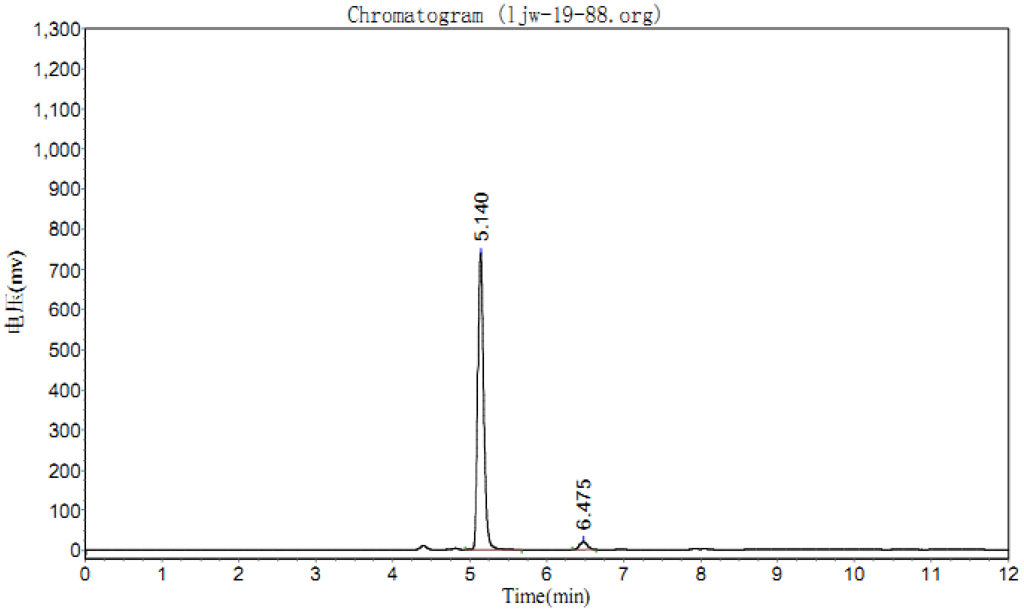

Peak No.	Peak ID	Ret Time	Height	Area	Conc.
1		5.140	737342.250	3987581.500	96.5031
2		6.475	20501.066	144494.297	3.4969
Total			757843.316	4132075.797	100.0000

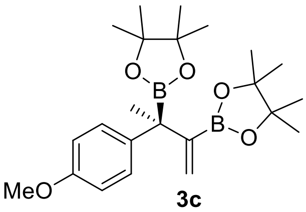

3c

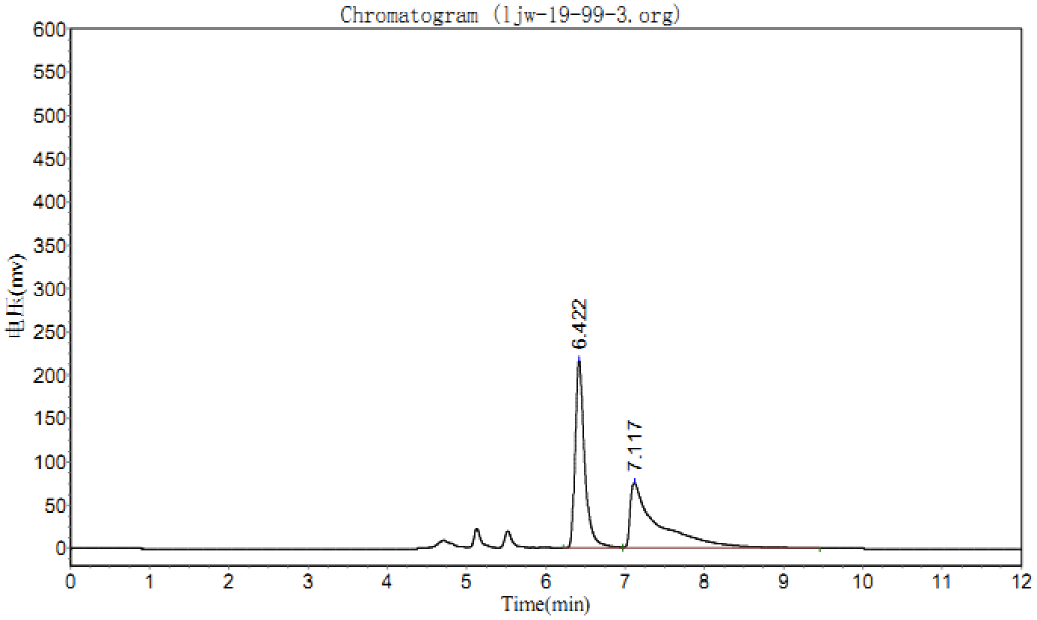

Peak No.	Peak ID	Ret Time	Height	Area	Conc.
1		6.422	215655.844	1836346.625	49.7242
2		7.117	75111.141	1856717.000	50.2758
Total			290766.984	3693063.625	100.0000

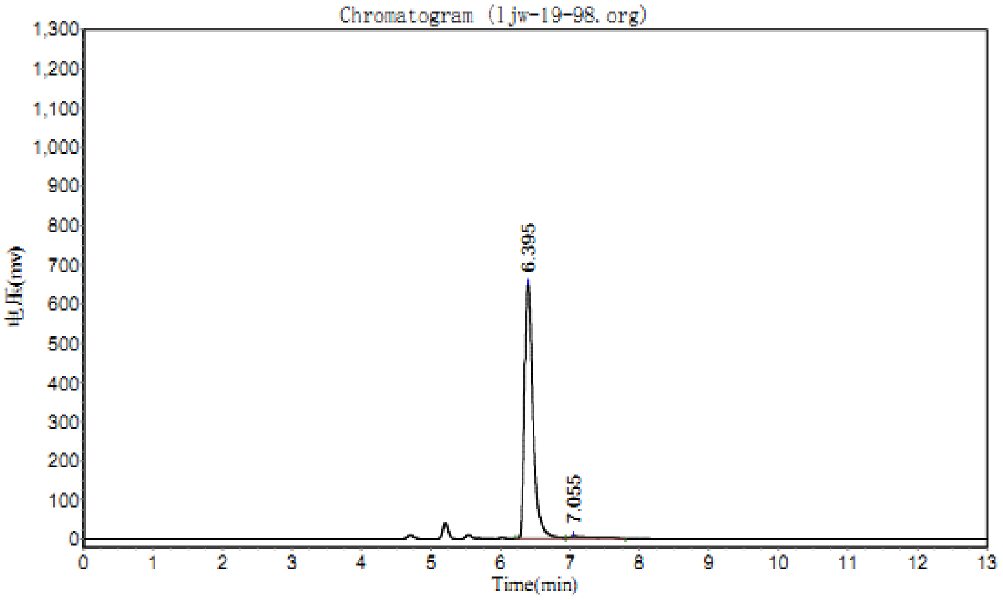

Peak No.	Peak ID	Ret Time	Height	Area	Conc.
1		6.395	649506.438	5469564.500	97.0784
2		7.055	6012.103	164605.500	2.9216
Total			655518.540	5634170.000	100.0000

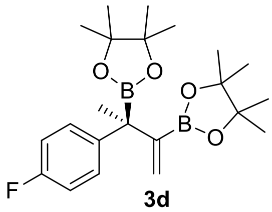

3d

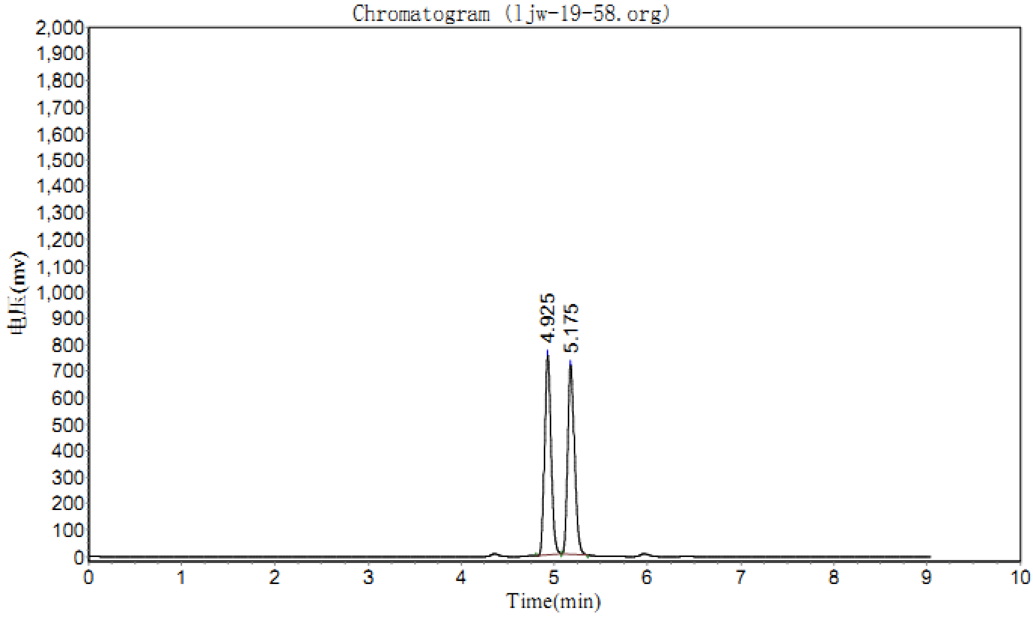

Peak No.	Peak ID	Ret Time	Height	Area	Conc.
1		4.925	752237.438	3641601.750	49.9913
2		5.175	713567.813	3642875.500	50.0087
Total			1465805.250	7284477.250	100.0000

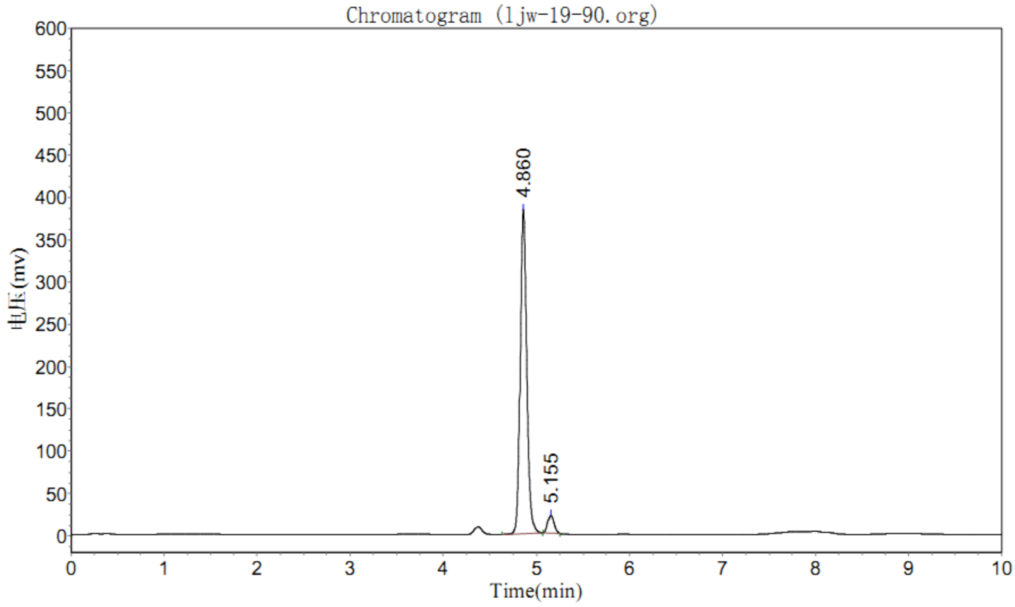

Peak No.	Peak ID	Ret Time	Height	Area	Conc.
1		4.860	383485.313	1892385.625	95.0337
2		5.155	20867.223	98892.391	4.9663
Total			404352.535	1991278.016	100.0000

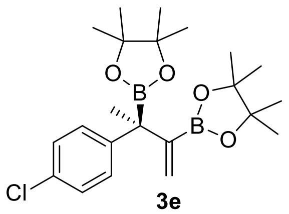

3e

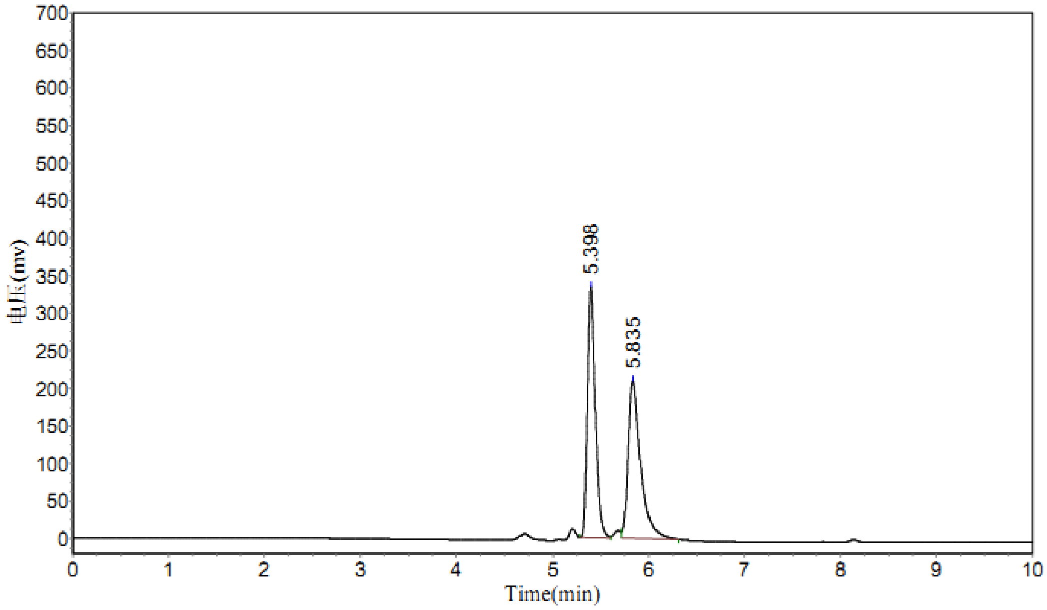

Peak No.	Peak ID	Ret Time	Height	Area	Conc.
1		5.398	333972.656	1921781.375	49.9010
2		5.835	209372.406	1929408.000	50.0990
Total			543345.063	3851189.375	100.0000

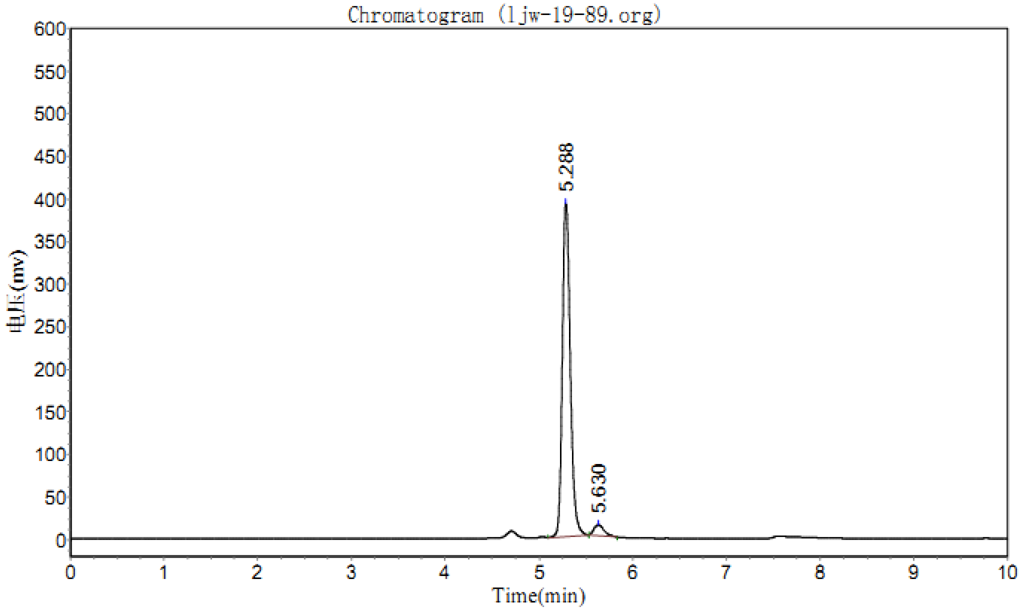

Peak No.	Peak ID	Ret Time	Height	Area	Conc.
1		5.288	391123.688	2237415.750	96.0148
2		5.630	12269.142	92865.797	3.9852
Total			403392.829	2330281.547	100.0000

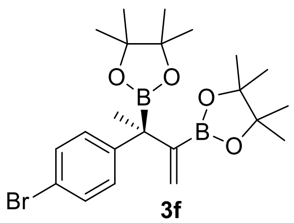

3f

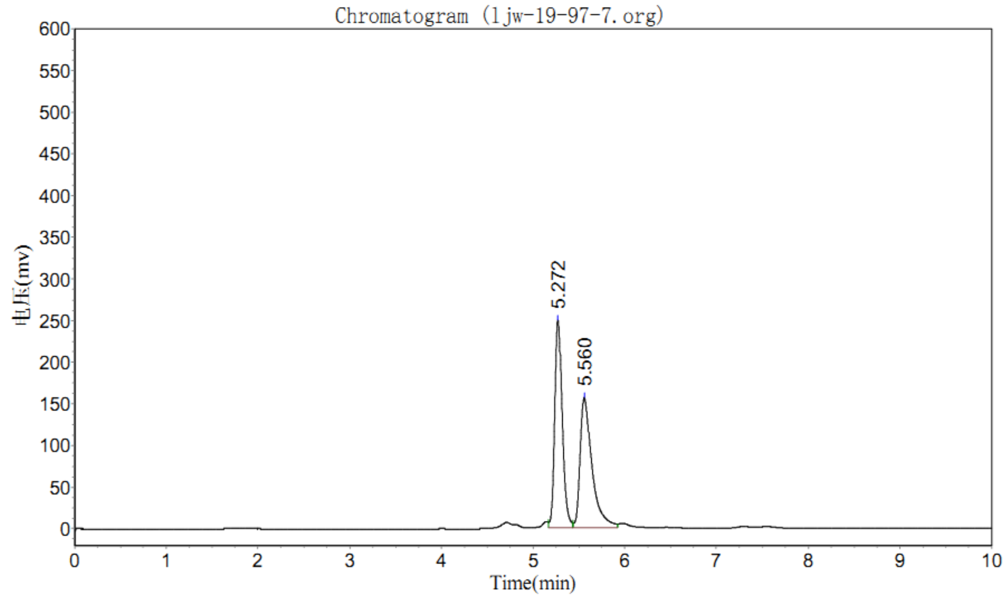

Peak No.	Peak ID	Ret Time	Height	Area	Conc.
1		5.272	249293.281	1424821.000	49.9347
2		5.560	155967.672	1428548.250	50.0653
Total			405260.953	2853369.250	100.0000

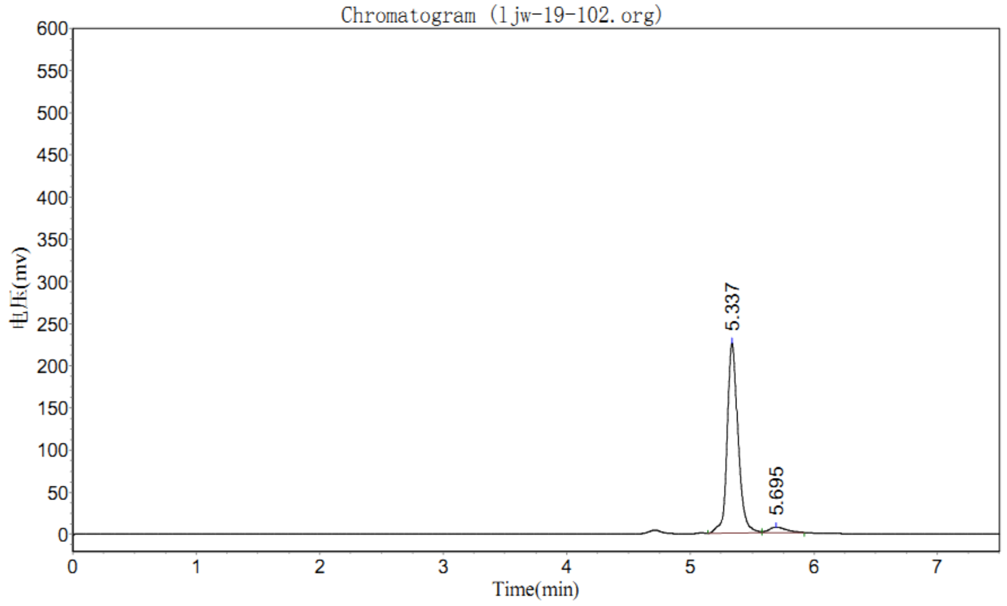

Peak No.	Peak ID	Ret Time	Height	Area	Conc.
1		5.337	225482.063	1380376.500	95.2810
2		5.695	6897.184	68366.430	4.7190
Total			232379.246	1448742.930	100.0000

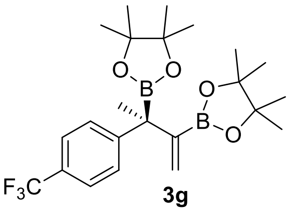

3g

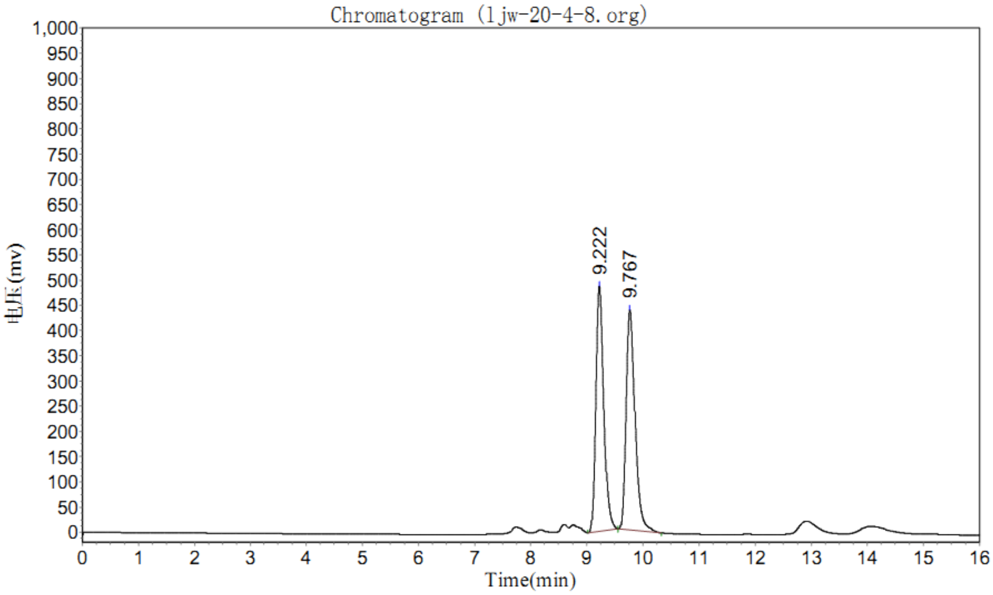

Peak No.	Peak ID	Ret Time	Height	Area	Conc.
1		9.222	485820.500	4819668.500	49.7508
2		9.767	436909.531	4867953.500	50.2492
Total			922730.031	9687622.000	100.0000

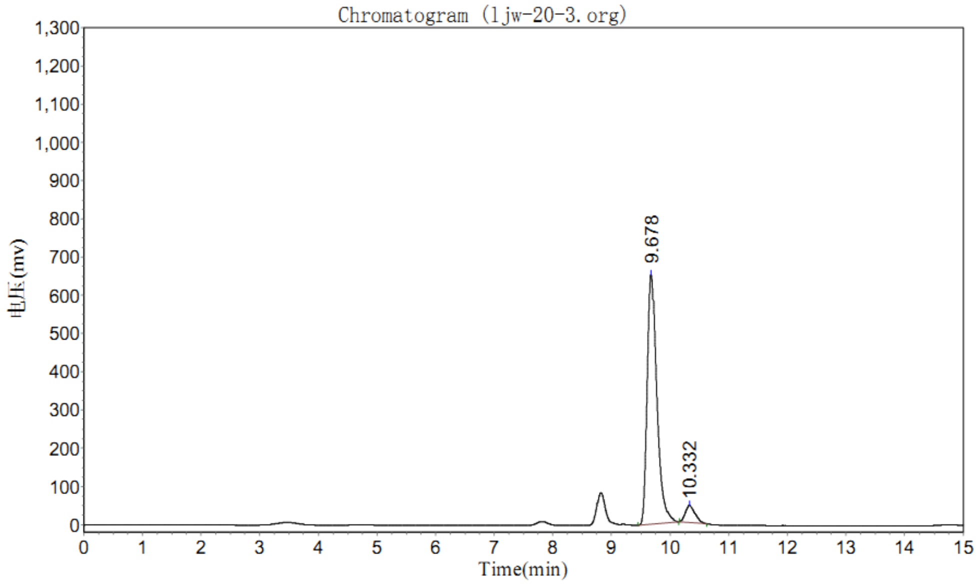

Peak No.	Peak ID	Ret Time	Height	Area	Conc.
1		9.678	652401.125	7481558.000	93.6820
2		10.332	43714.844	504566.781	6.3180
Total			696115.969	7986124.781	100.0000

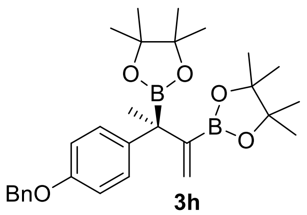

3h

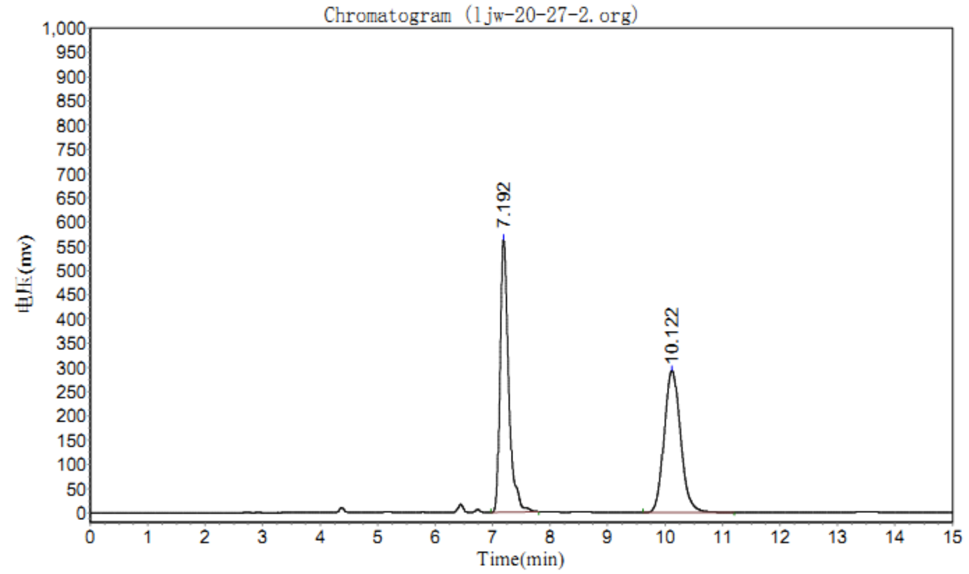

Peak No.	Peak ID	Ret Time	Height	Area	Conc.
1		7.192	562343.000	5884960.500	50.3005
2		10.122	292035.813	5814652.000	49.6995
Total			854378.813	11699612.500	100.0000

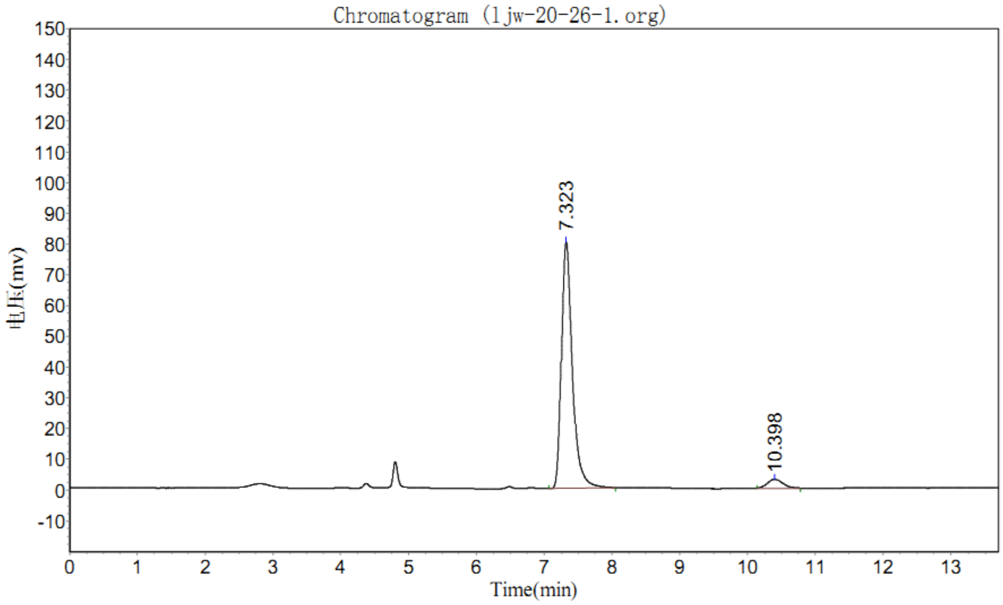

Peak No.	Peak ID	Ret Time	Height	Area	Conc.
1		7.323	80168.555	902015.813	95.0865
2		10.398	2942.202	46610.598	4.9135
Total			83110.756	948626.410	100.0000

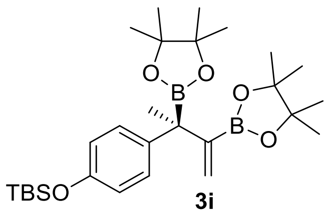

3i

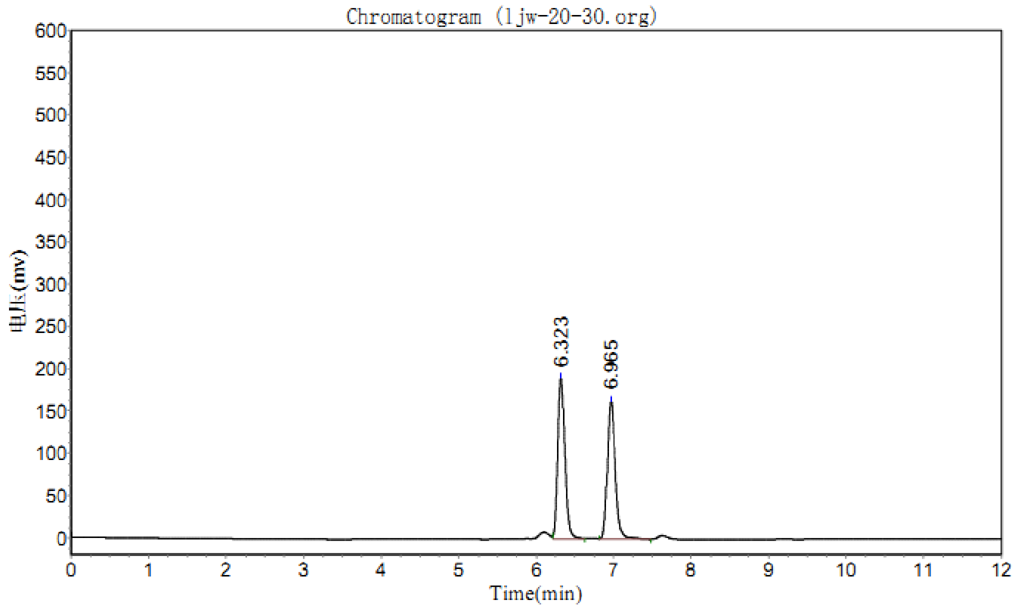

Peak No.	Peak ID	Ret Time	Height	Area	Conc.
1		6.323	190254.813	1197377.000	49.9567
2		6.965	163239.188	1199452.625	50.0433
Total			353494.000	2396829.625	100.0000

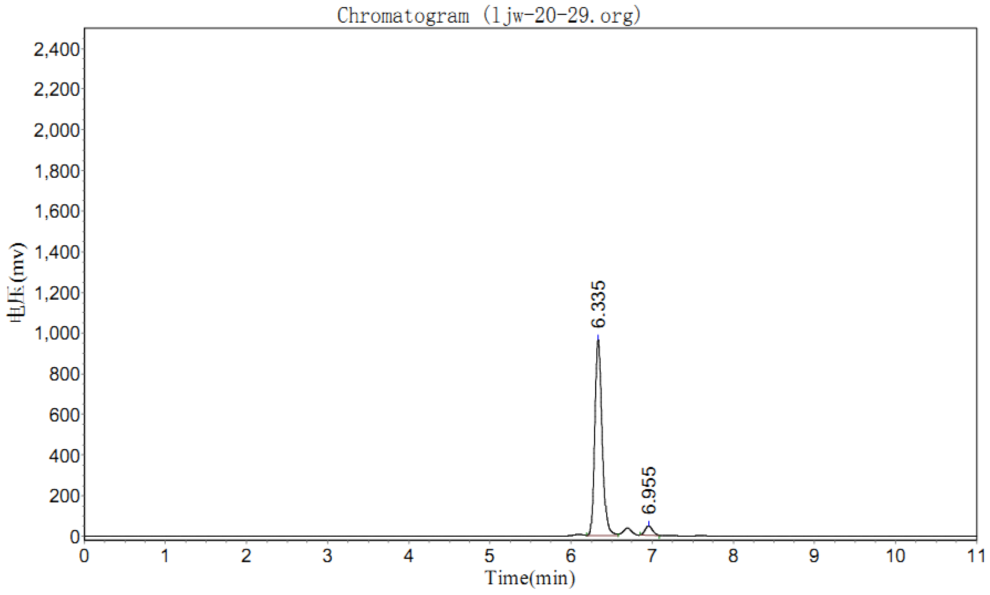

Peak No.	Peak ID	Ret Time	Height	Area	Conc.
1		6.335	964526.750	6172118.000	95.5166
2		6.955	44364.574	289708.906	4.4834
Total			1008891.324	6461826.906	100.0000

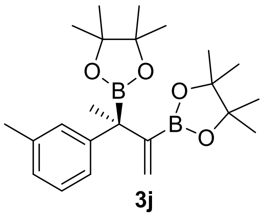

3j

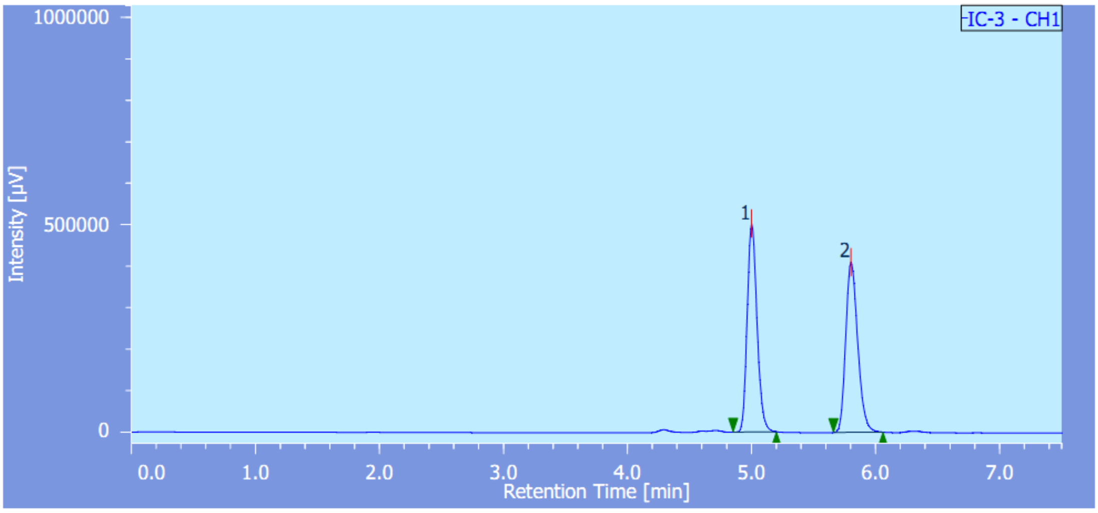

#	Peak Name	CH	tR [min]	Area [µV·sec]	Height [µV]	Area%	Height%	Quantity	NTP	Resolution	Symmetry Factor	Warning
1	Unknown	1	5.000	2760225	502476	50.014	55.041	N/A	19438	5.030	1.199	
2	Unknown	1	5.800	2758732	410444	49.986	44.959	N/A	17473	N/A	1.253	

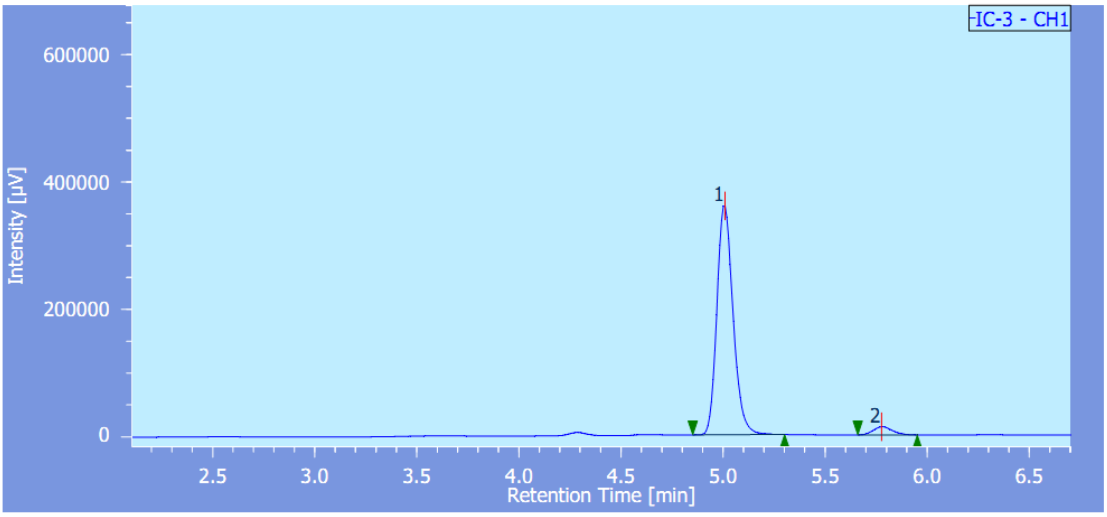

#	Peak Name	CH	tR [min]	Area [µV·sec]	Height [µV]	Area%	Height%	Quantity	NTP	Resolution	Symmetry Factor	Warning
1	Unknown	1	5.008	1986947	358894	96.031	96.562	N/A	19523	4.916	1.159	
2	Unknown	1	5.775	82131	12779	3.969	3.438	N/A	18602	N/A	1.206	

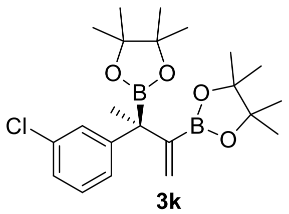

3k

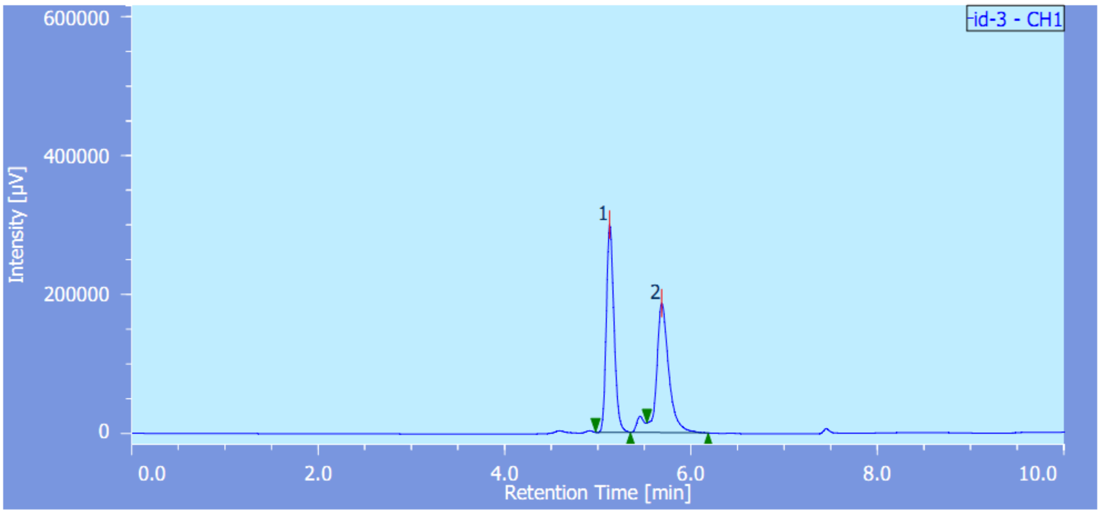

#	Peak Name	CH	tR [min]	Area [µV·sec]	Height [µV]	Area%	Height%	Quantity	NTP	Resolution	Symmetry Factor	Warning
1	Unknown	1	5.125	1710018	298694	50.823	61.594	N/A	19159	3.113	1.255	
2	Unknown	1	5.683	1654631	186249	49.177	38.406	N/A	11548	N/A	N/A	

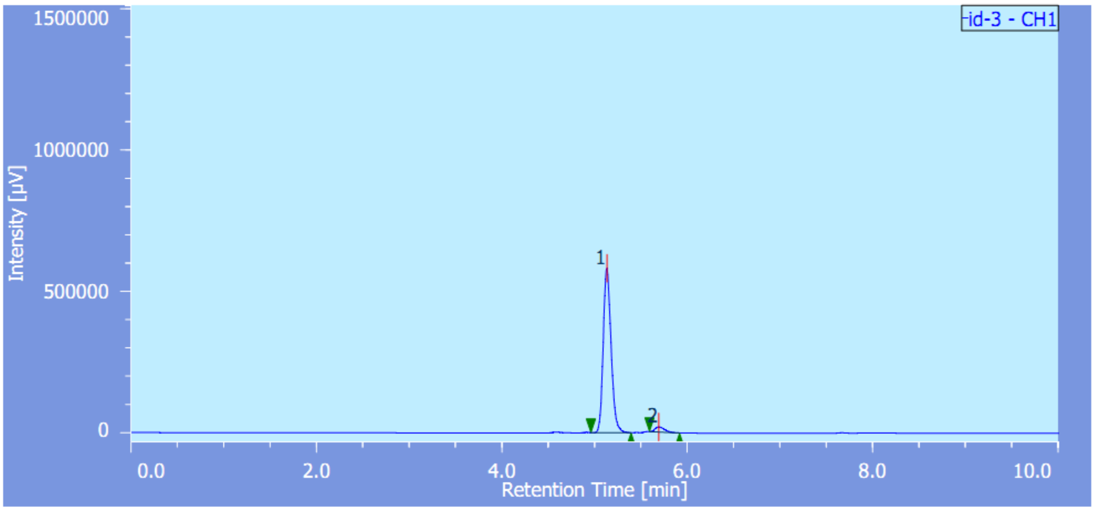

#	Peak Name	CH	tR [min]	Area [µV·sec]	Height [µV]	Area%	Height%	Quantity	NTP	Resolution	Symmetry Factor	Warning
1	Unknown	1	5.133	3433176	580941	96.373	96.997	N/A	18095	3.264	1.169	
2	Unknown	1	5.692	129208	17989	3.627	3.003	N/A	14298	N/A	1.415	

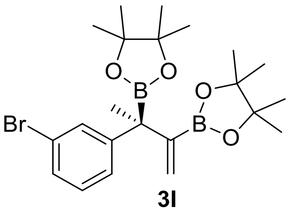

3l

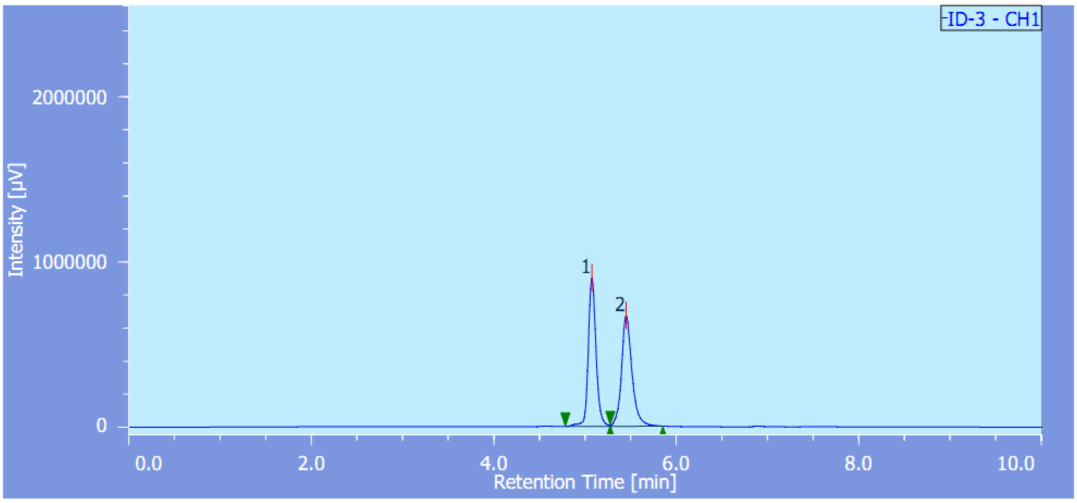

#	Peak Name	CH	tR [min]	Area [µV·sec]	Height [µV]	Area%	Height%	Quantity	NTP	Resolution	Symmetry Factor	Warning
1	Unknown	1	5.075	5308184	900052	49.801	57.260	N/A	18943	2.209	1.143	
2	Unknown	1	5.450	5350644	671821	50.199	42.740	N/A	12769	N/A	1.175	

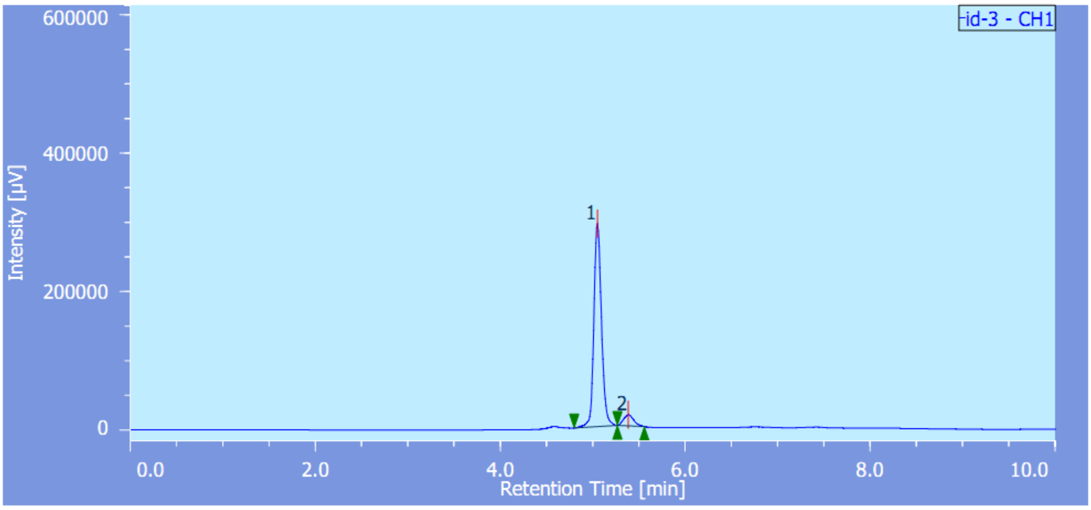

#	Peak Name	CH	tR [min]	Area [µV·sec]	Height [µV]	Area%	Height%	Quantity	NTP	Resolution	Symmetry Factor	Warning
1	Unknown	1	5.050	1668098	293412	93.077	94.794	N/A	19575	1.916	1.157	
2	Unknown	1	5.383	124067	16114	6.923	5.206	N/A	11088	N/A	1.232	

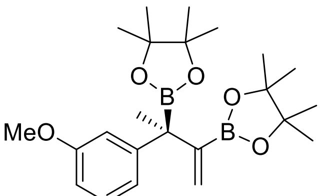

3m

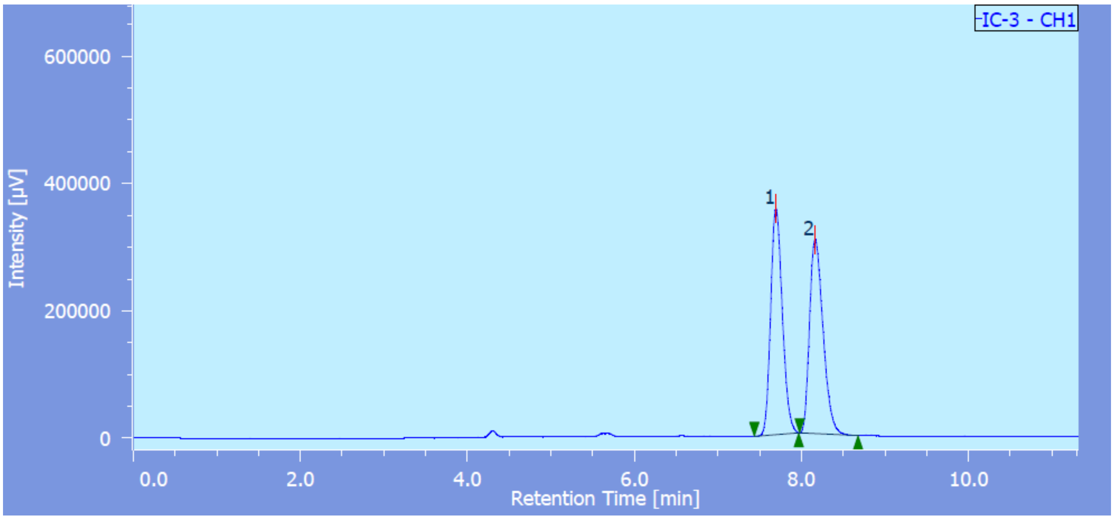

#	Peak Name	CH	tR [min]	Area [µV·sec]	Height [µV]	Area%	Height%	Quantity	NTP	Resolution	Symmetry Factor	Warning
1	Unknown	1	7.692	3439211	355473	50.157	53.856	N/A	14591	1.698	1.200	
2	Unknown	1	8.158	3417682	304566	49.843	46.144	N/A	12133	N/A	1.400	

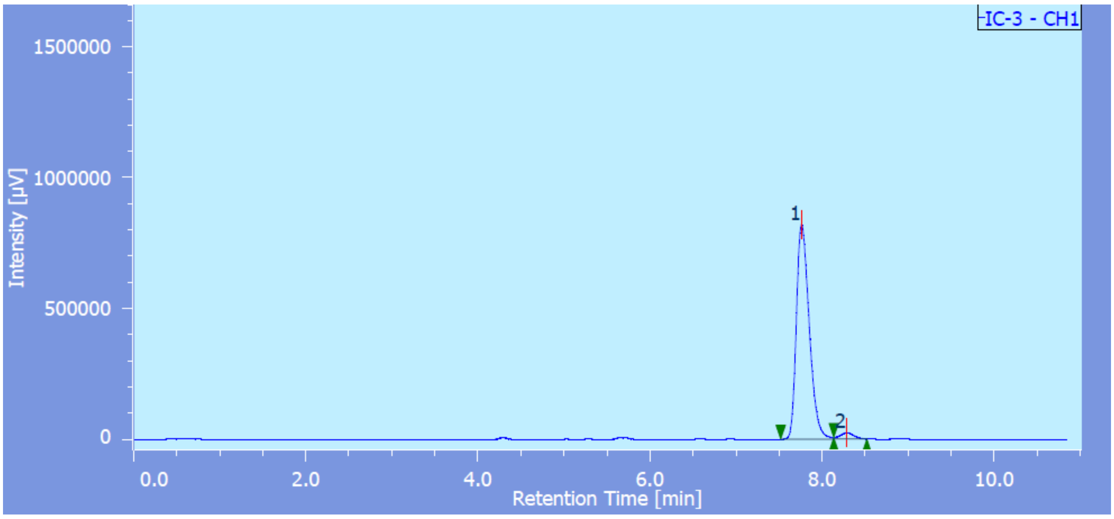

#	Peak Name	CH	tR [min]	Area [µV·sec]	Height [µV]	Area%	Height%	Quantity	NTP	Resolution	Symmetry Factor	Warning
1	Unknown	1	7.758	8553961	819293	97.006	97.156	N/A	12910	1.865	1.375	
2	Unknown	1	8.283	264014	23986	2.994	2.844	N/A	12937	N/A	N/A	

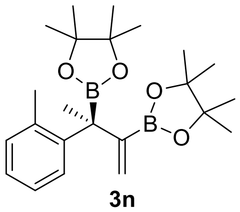

3n

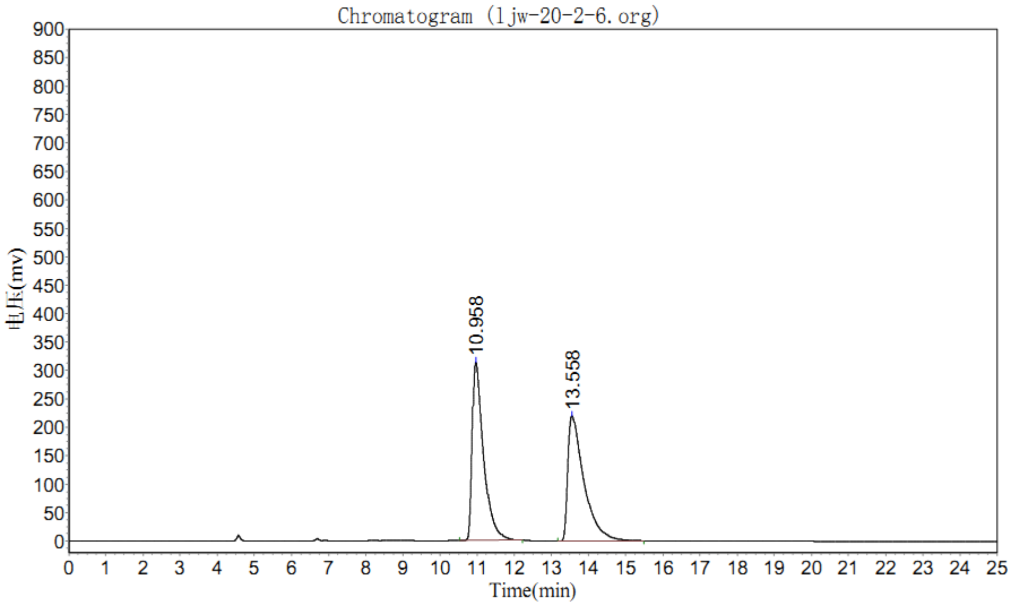

Peak No.	Peak ID	Ret Time	Height	Area	Conc.
1		10.958	312925.656	6606156.500	49.8662
2		13.558	219594.391	6641596.500	50.1338
Total			532520.047	13247753.000	100.0000

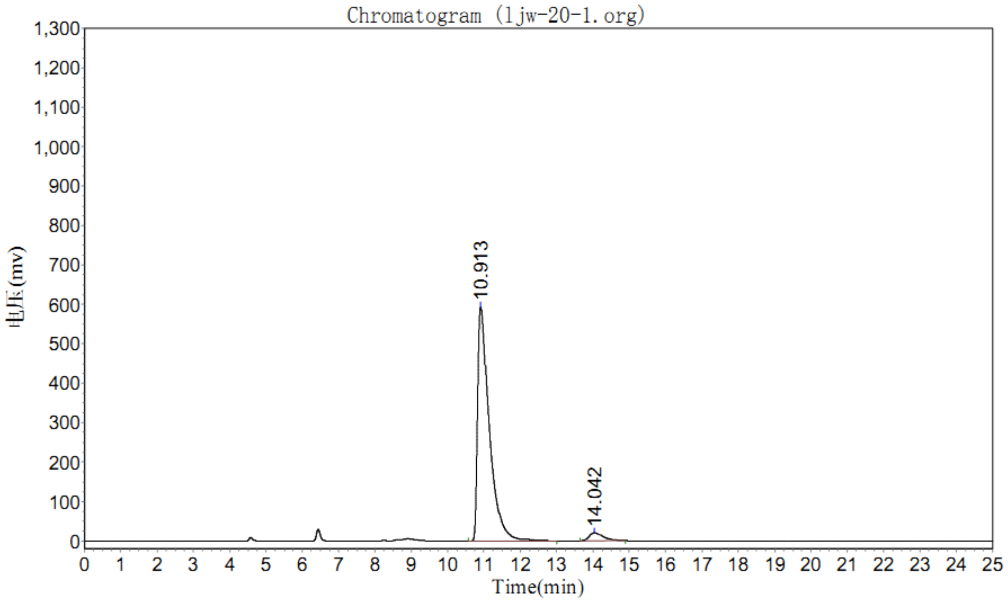

Peak No.	Peak ID	Ret Time	Height	Area	Conc.
1		10.913	593058.000	13318128.000	96.0389
2		14.042	19276.408	549298.813	3.9611
Total			612334.408	13867426.812	100.0000

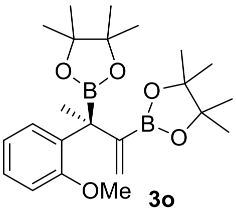

3o

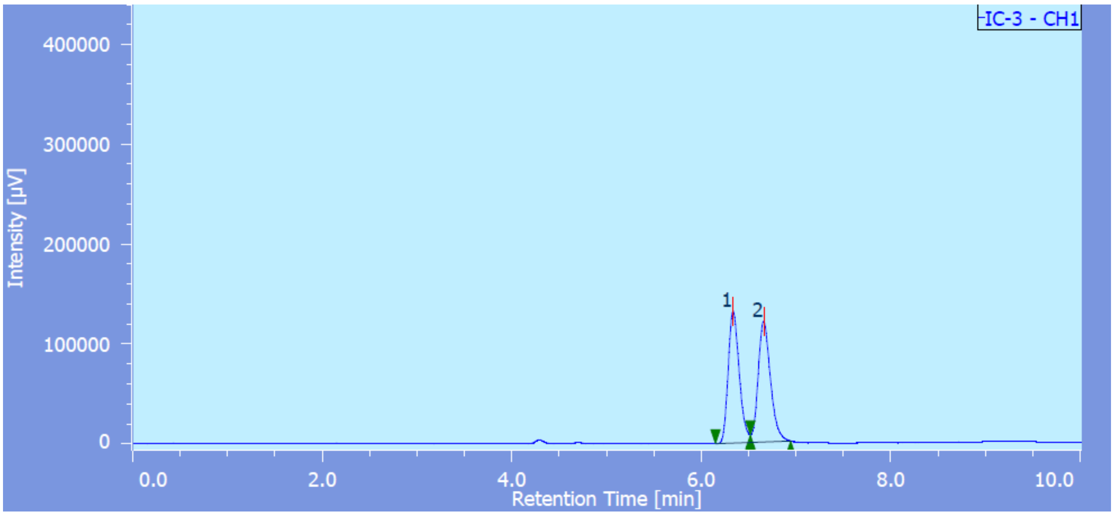

#	Peak Name	CH	tR [min]	Area [µV·sec]	Height [µV]	Area%	Height%	Quantity	NTP	Resolution	Symmetry Factor	Warning
1	Unknown	1	6.333	1073307	132057	49.601	52.243	N/A	14314	1.468	N/A	
2	Unknown	1	6.658	1090589	120716	50.399	47.757	N/A	13169	N/A	N/A	

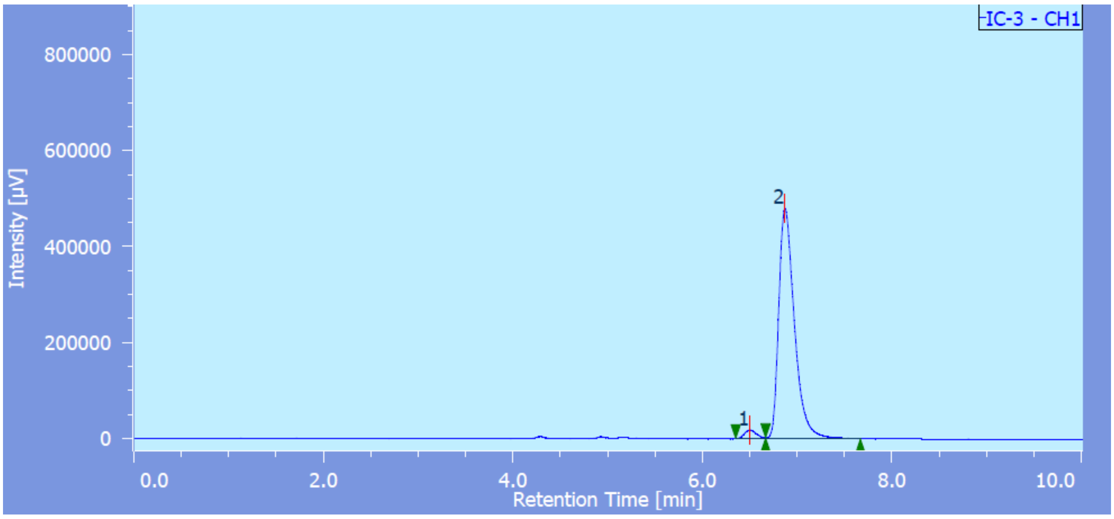

#	Peak Name	CH	tR [min]	Area [µV·sec]	Height [µV]	Area%	Height%	Quantity	NTP	Resolution	Symmetry Factor	Warning
1	Unknown	1	6.500	165176	18153	2.916	3.644	N/A	11518	1.383	N/A	
2	Unknown	1	6.867	5499336	480036	97.084	96.356	N/A	8999	N/A	1.534	

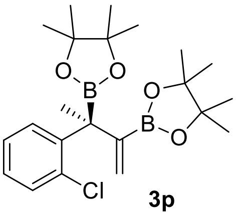

3p

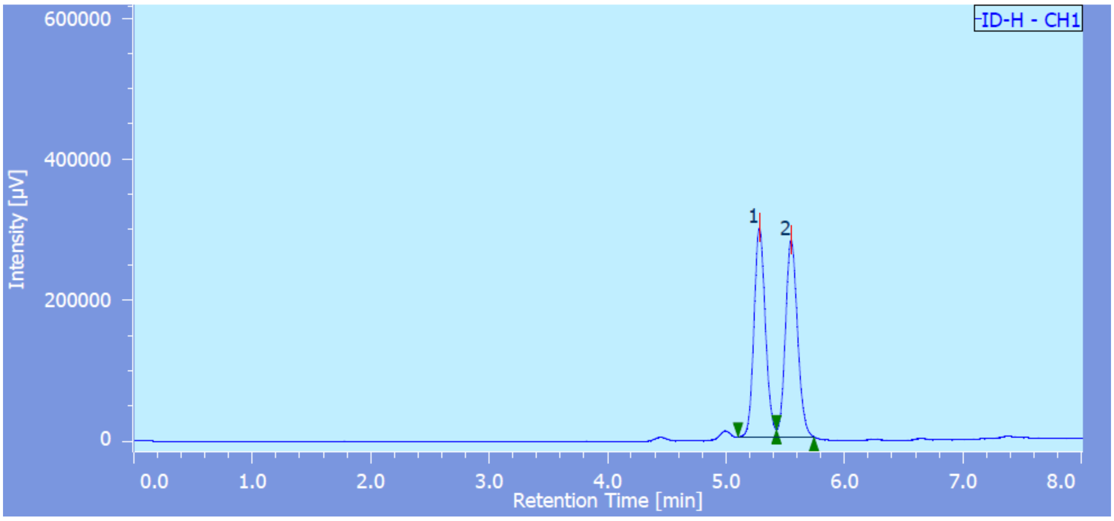

#	Peak Name	CH	tR [min]	Area [µV·sec]	Height [µV]	Area%	Height%	Quantity	NTP	Resolution	Symmetry Factor	Warning
1	Unknown	1	5.283	1980063	296657	49.980	51.507	N/A	14730	1.486	1.088	
2	Unknown	1	5.550	1981629	279295	50.020	48.493	N/A	14268	N/A	1.089	

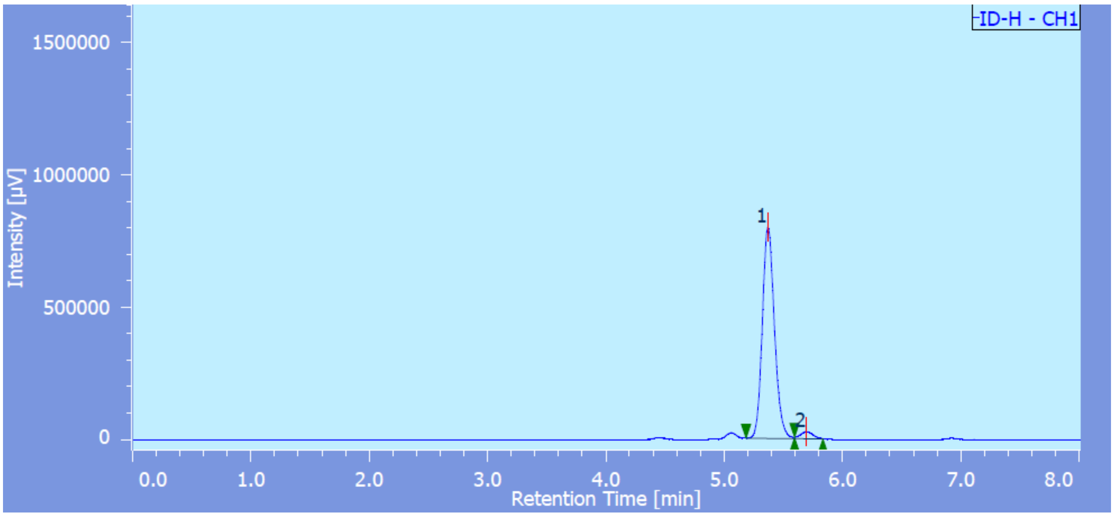

#	Peak Name	CH	tR [min]	Area [µV·sec]	Height [µV]	Area%	Height%	Quantity	NTP	Resolution	Symmetry Factor	Warning
1	Unknown	1	5.367	5679378	799058	96.663	96.780	N/A	13597	1.699	1.174	
2	Unknown	1	5.692	196034	26590	3.337	3.220	N/A	13037	N/A	N/A	

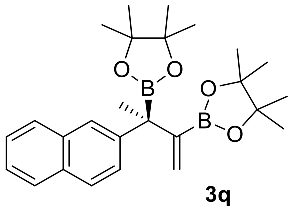

3q

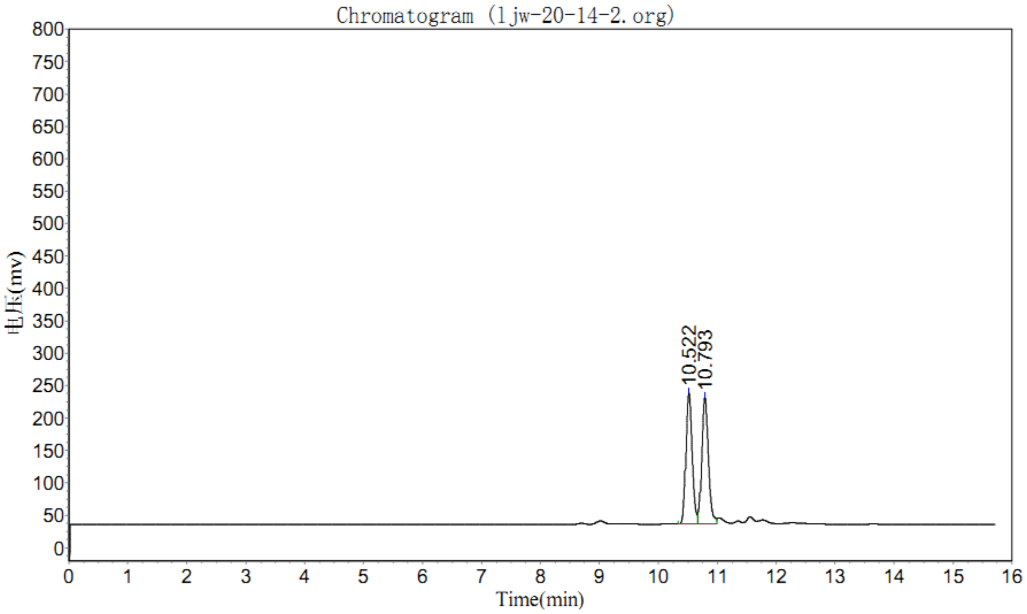

Peak No.	Peak ID	Ret Time	Height	Area	Conc.
1		10.522	202002.094	1512127.250	49.2366
2		10.793	195260.750	1559016.750	50.7634
Total			397262.844	3071144.000	100.0000

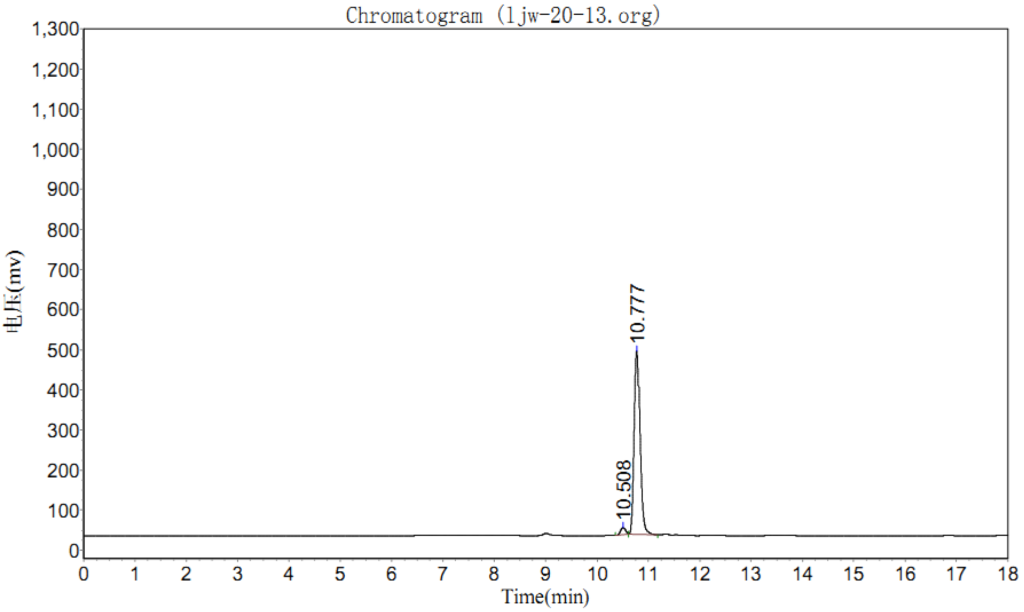

Peak No.	Peak ID	Ret Time	Height	Area	Conc.
1		10.508	17564.004	114069.539	3.0734
2		10.777	456951.625	3597414.250	96.9266
Total			474515.629	3711483.789	100.0000

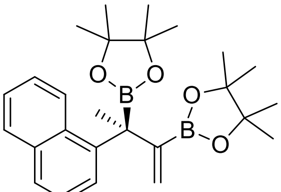

3r

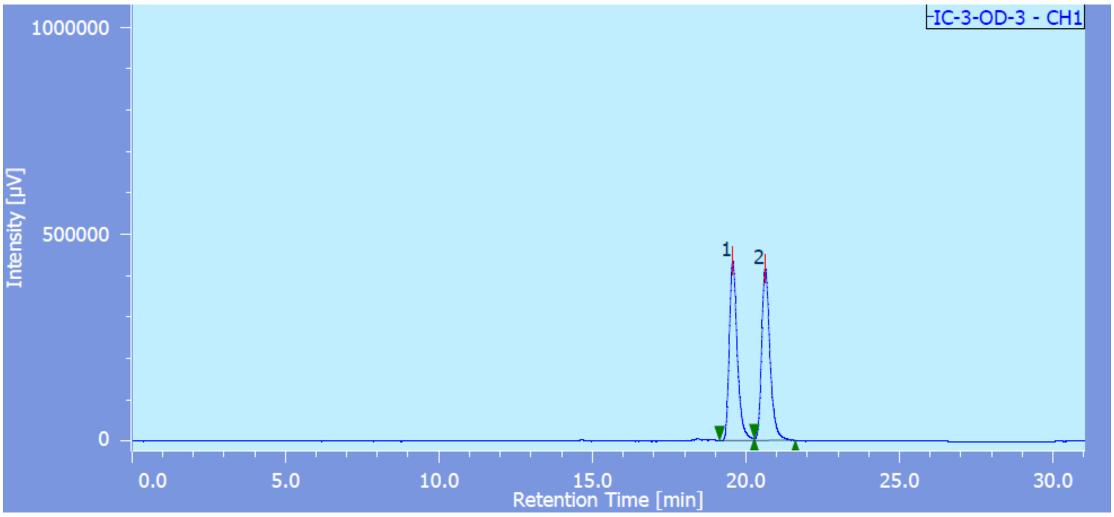

#	Peak Name	CH	tR [min]	Area [µV·sec]	Height [µV]	Area%	Height%	Quantity	NTP	Resolution	Symmetry Factor	Warning
1	Unknown	1	19.567	7809680	433992	49.869	51.085	N/A	29926	2.288	1.366	
2	Unknown	1	20.625	7850768	415561	50.131	48.915	N/A	30139	N/A	1.341	

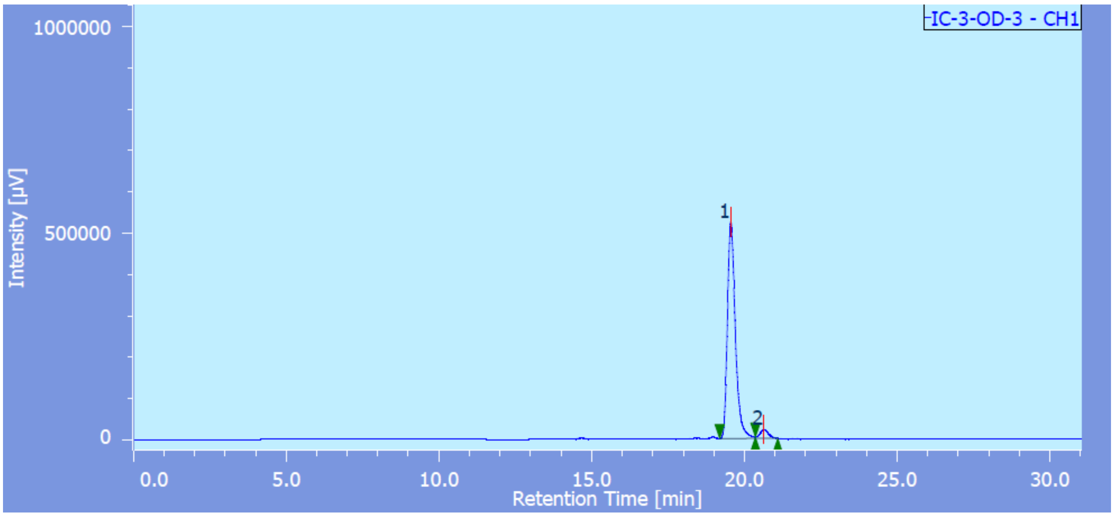

#	Peak Name	CH	tR [min]	Area [µV·sec]	Height [µV]	Area%	Height%	Quantity	NTP	Resolution	Symmetry Factor	Warning
1	Unknown	1	19.550	9423673	524540	95.724	96.067	N/A	30163	2.261	1.376	
2	Unknown	1	20.633	421003	21473	4.276	3.933	N/A	26151	N/A	N/A	

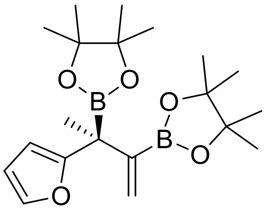

3s

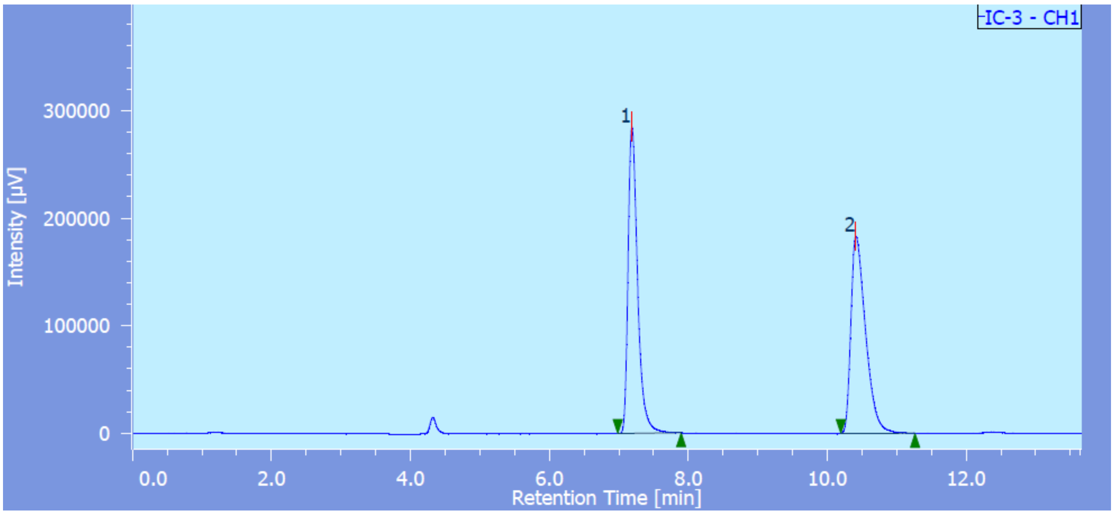

#	Peak Name	CH	tR [min]	Area [µV·sec]	Height [µV]	Area%	Height%	Quantity	NTP	Resolution	Symmetry Factor	Warning
1	Unknown	1	7.183	2725235	284973	50.089	60.914	N/A	14343	10.370	1.516	
2	Unknown	1	10.408	2715582	182855	49.911	39.086	N/A	11773	N/A	1.675	

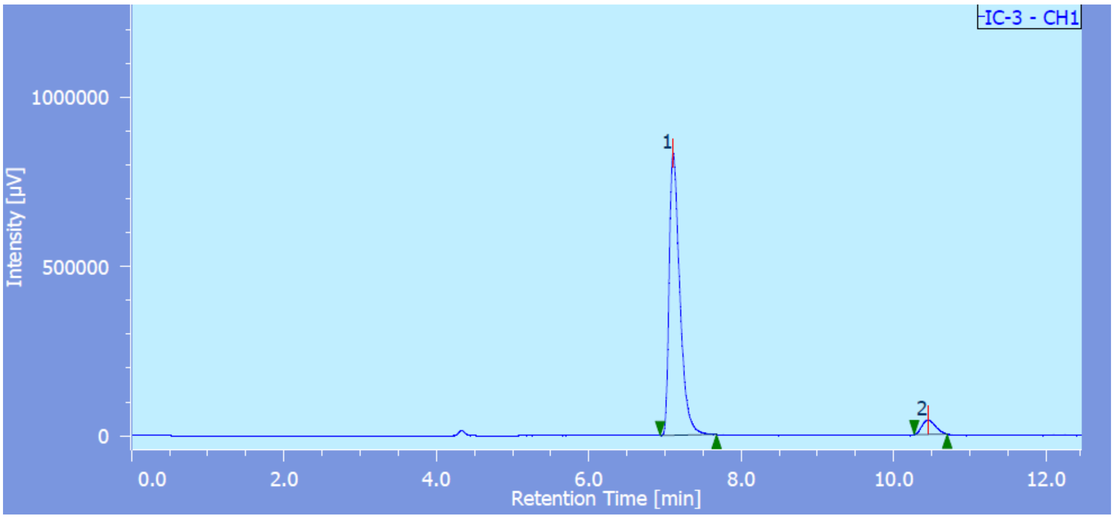

#	Peak Name	CH	tR [min]	Area [µV·sec]	Height [µV]	Area%	Height%	Quantity	NTP	Resolution	Symmetry Factor	Warning
1	Unknown	1	7.108	8090043	833495	93.814	95.071	N/A	13242	11.475	1.579	
2	Unknown	1	10.450	533492	43209	6.186	4.929	N/A	15397	N/A	1.179	

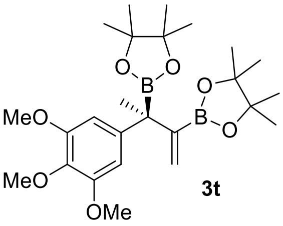

3t

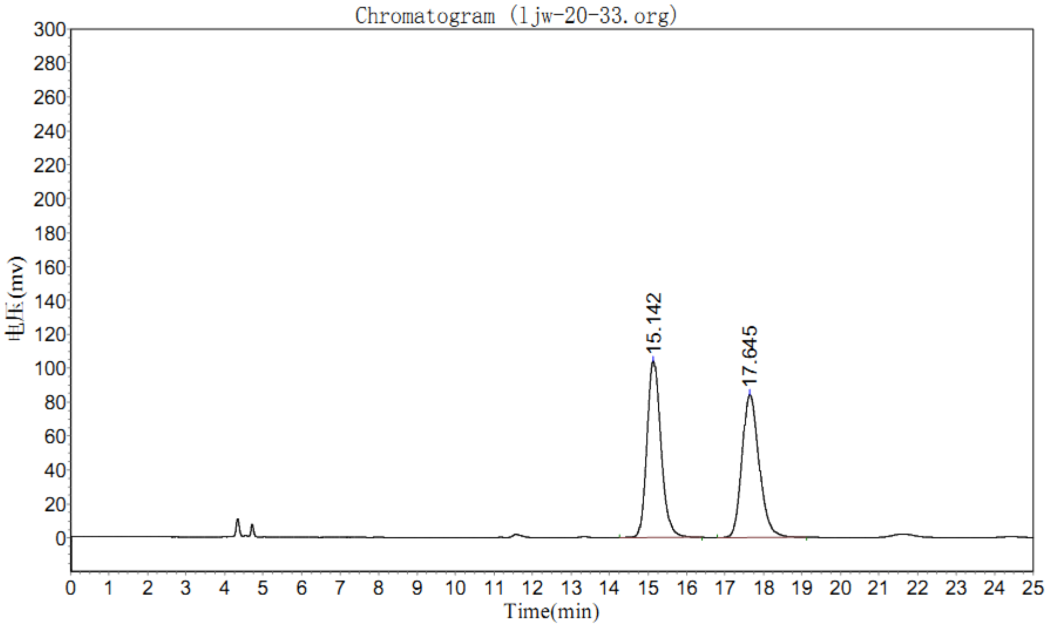

Peak No.	Peak ID	Ret Time	Height	Area	Conc.
1		15.142	104116.086	2649219.250	50.1819
2		17.645	84493.305	2630012.000	49.8181
Total			188609.391	5279231.250	100.0000

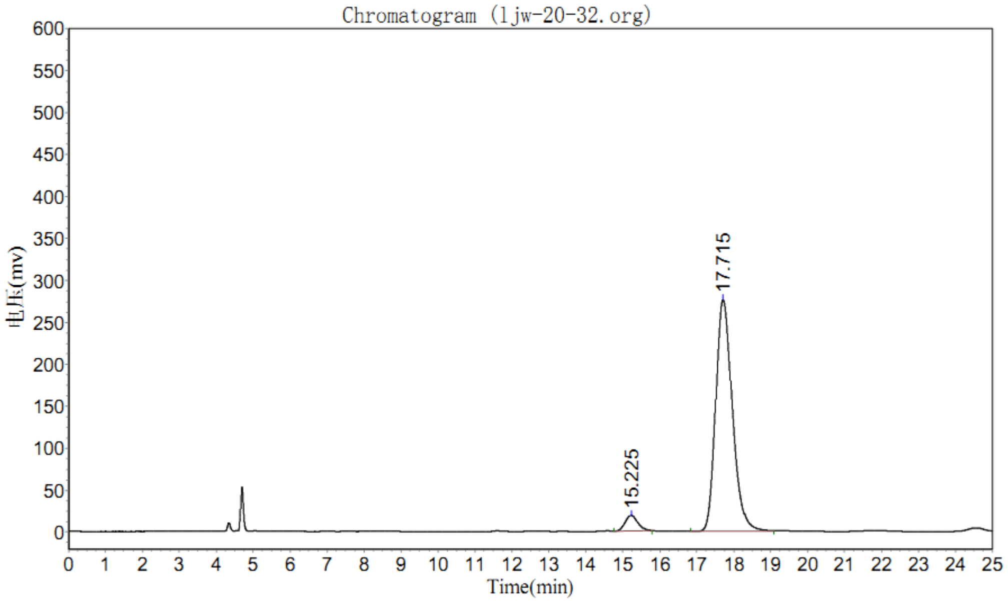

Peak No.	Peak ID	Ret Time	Height	Area	Conc.
1		15.225	18300.555	443189.594	4.8358
2		17.715	276260.406	8721627.000	95.1642
Total			294560.961	9164816.594	100.0000

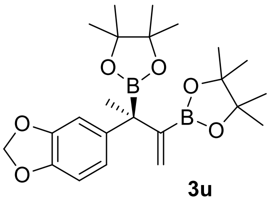

3u

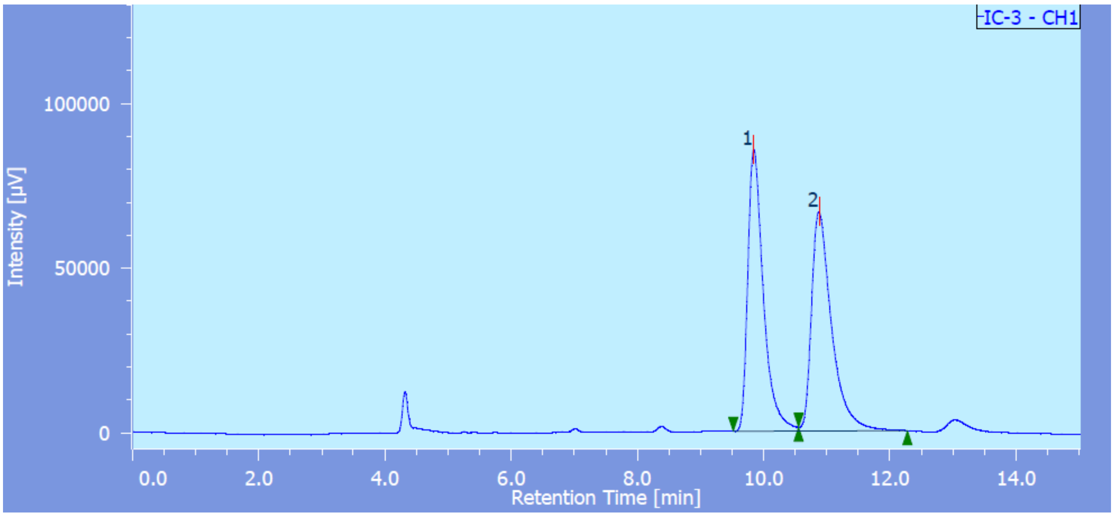

#	Peak Name	CH	tR [min]	Area [µV·sec]	Height [µV]	Area%	Height%	Quantity	NTP	Resolution	Symmetry Factor	Warning
1	Unknown	1	9.842	1470621	85671	49.927	56.251	N/A	8609	2.146	1.632	
2	Unknown	1	10.875	1474934	66630	50.073	43.749	N/A	6454	N/A	1.698	

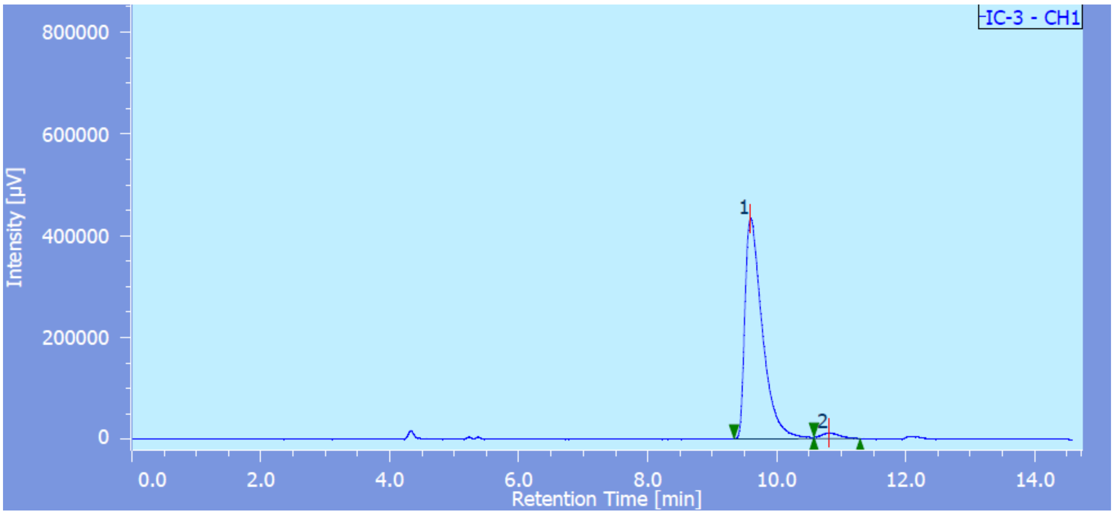

#	Peak Name	CH	tR [min]	Area [µV·sec]	Height [µV]	Area%	Height%	Quantity	NTP	Resolution	Symmetry Factor	Warning
1	Unknown	1	9.592	8103483	433285	97.178	97.415	N/A	6679	2.409	1.987	
2	Unknown	1	10.808	235350	11496	2.822	2.585	N/A	6334	N/A	N/A	

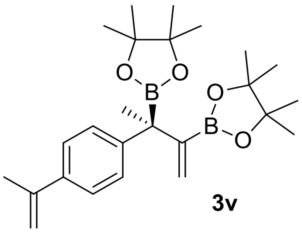

3v

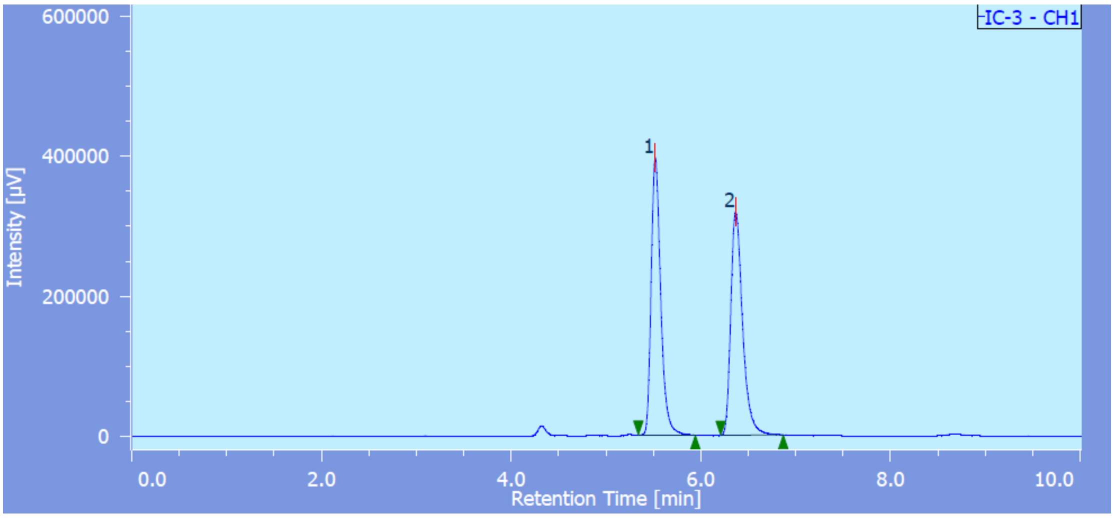

#	Peak Name	CH	tR [min]	Area [µV·sec]	Height [µV]	Area%	Height%	Quantity	NTP	Resolution	Symmetry Factor	Warning
1	Unknown	1	5.517	2762691	396502	49.919	55.357	N/A	15608	4.302	1.373	
2	Unknown	1	6.367	2771601	319758	50.081	44.643	N/A	13453	N/A	1.406	

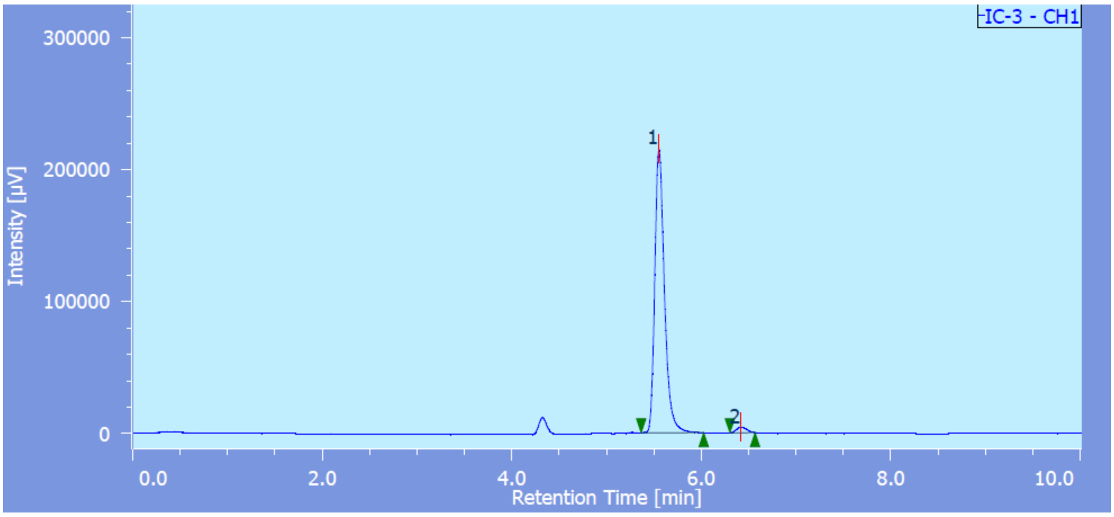

#	Peak Name	CH	tR [min]	Area [µV·sec]	Height [µV]	Area%	Height%	Quantity	NTP	Resolution	Symmetry Factor	Warning
1	Unknown	1	5.550	1525965	215246	97.921	98.070	N/A	15331	4.477	1.382	
2	Unknown	1	6.417	32406	4236	2.079	1.930	N/A	15089	N/A	1.157	

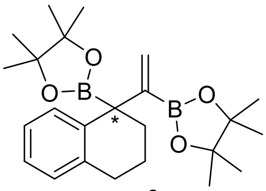

3w

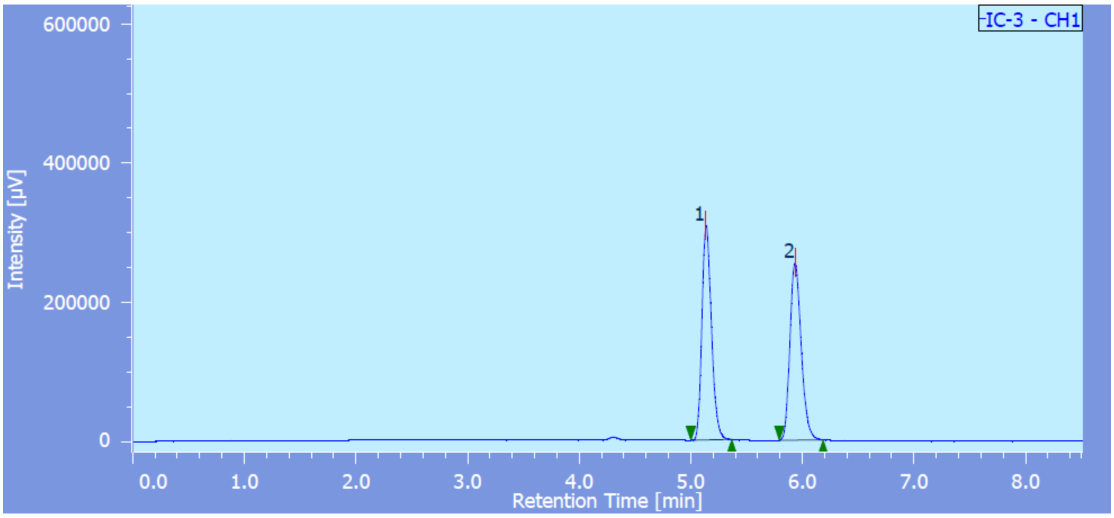

#	Peak Name	CH	tR [min]	Area [µV·sec]	Height [µV]	Area%	Height%	Quantity	NTP	Resolution	Symmetry Factor	Warning
1	Unknown	1	5.133	1818813	308157	50.161	54.684	N/A	17926	4.744	1.285	
2	Unknown	1	5.933	1807167	255367	49.839	45.316	N/A	16493	N/A	1.205	

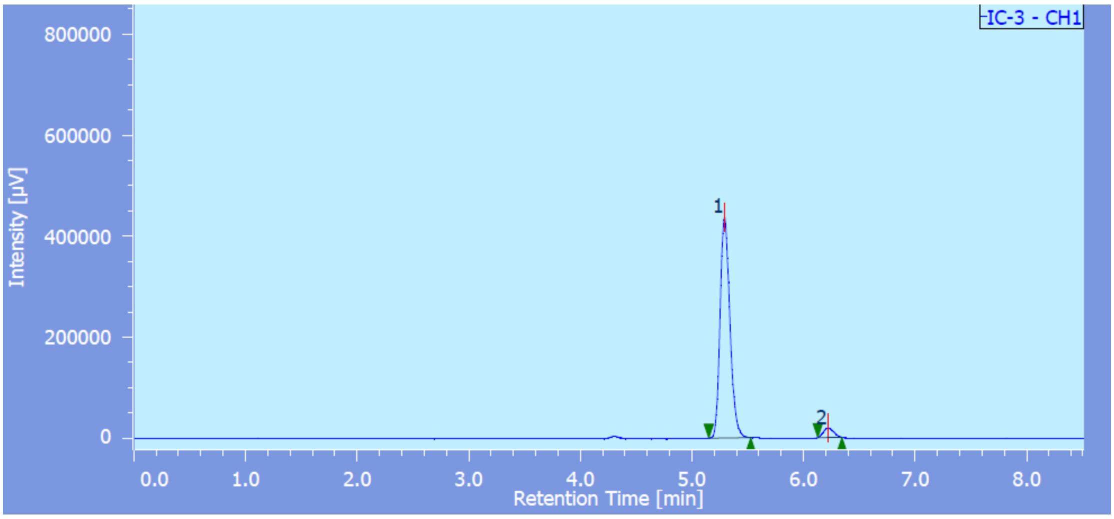

#	Peak Name	CH	tR [min]	Area [µV·sec]	Height [µV]	Area%	Height%	Quantity	NTP	Resolution	Symmetry Factor	Warning
1	Unknown	1	5.292	2663878	437318	95.682	95.920	N/A	17874	5.511	1.212	
2	Unknown	1	6.217	120215	18602	4.318	4.080	N/A	19455	N/A	1.167	

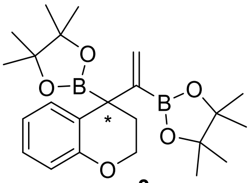

3x

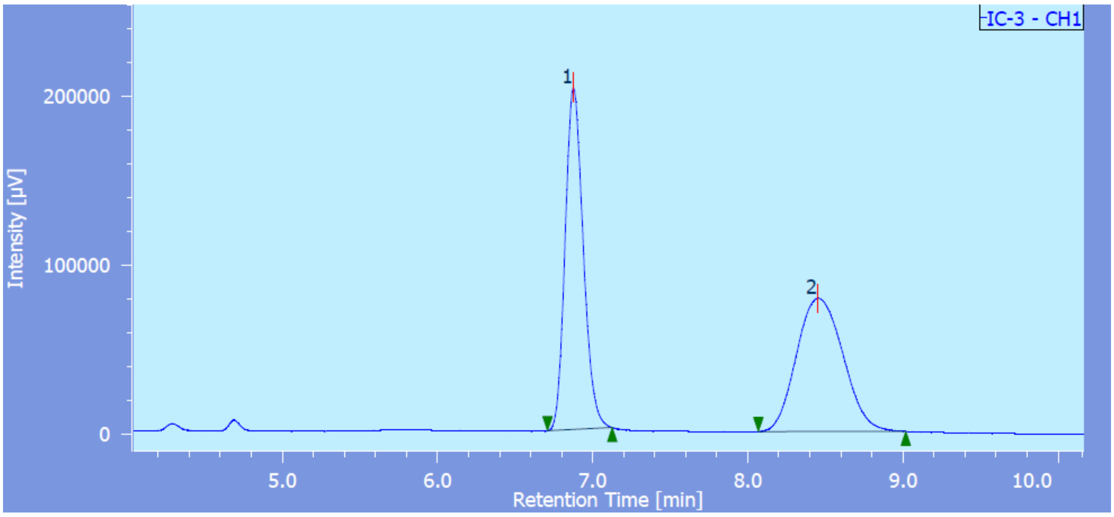

#	Peak Name	CH	tR [min]	Area [µV·sec]	Height [µV]	Area%	Height%	Quantity	NTP	Resolution	Symmetry Factor	Warning
1	Unknown	1	6.875	1645045	202484	50.021	71.978	N/A	16626	4.048	1.195	
2	Unknown	1	8.450	1643658	78828	49.979	28.022	N/A	3553	N/A	1.132	

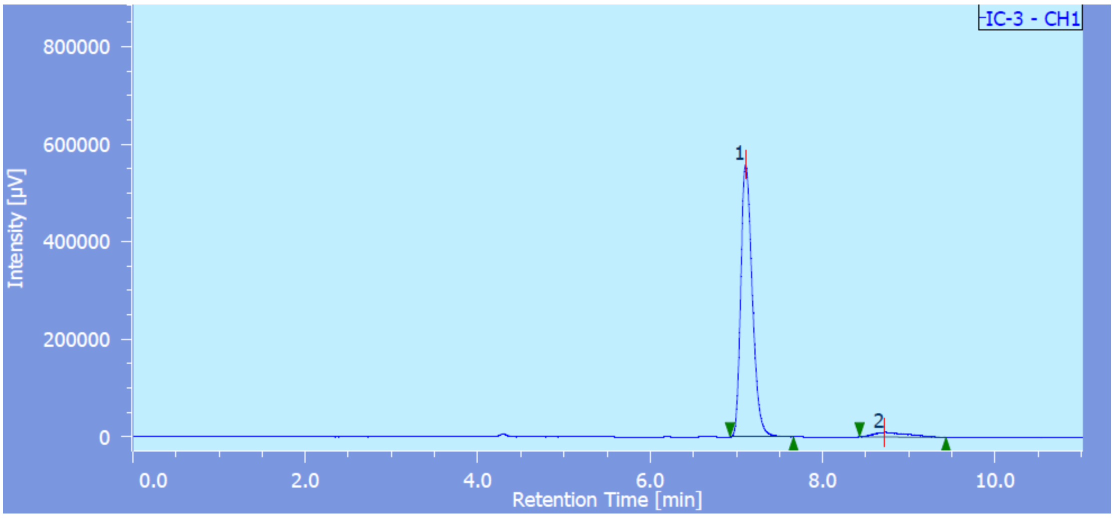

#	Peak Name	CH	tR [min]	Area [µV·sec]	Height [µV]	Area%	Height%	Quantity	NTP	Resolution	Symmetry Factor	Warning
1	Unknown	1	7.108	5195158	558114	95.103	98.384	N/A	13721	2.953	1.272	
2	Unknown	1	8.717	267533	9168	4.897	1.616	N/A	1685	N/A	1.624	

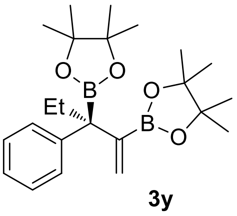

3y

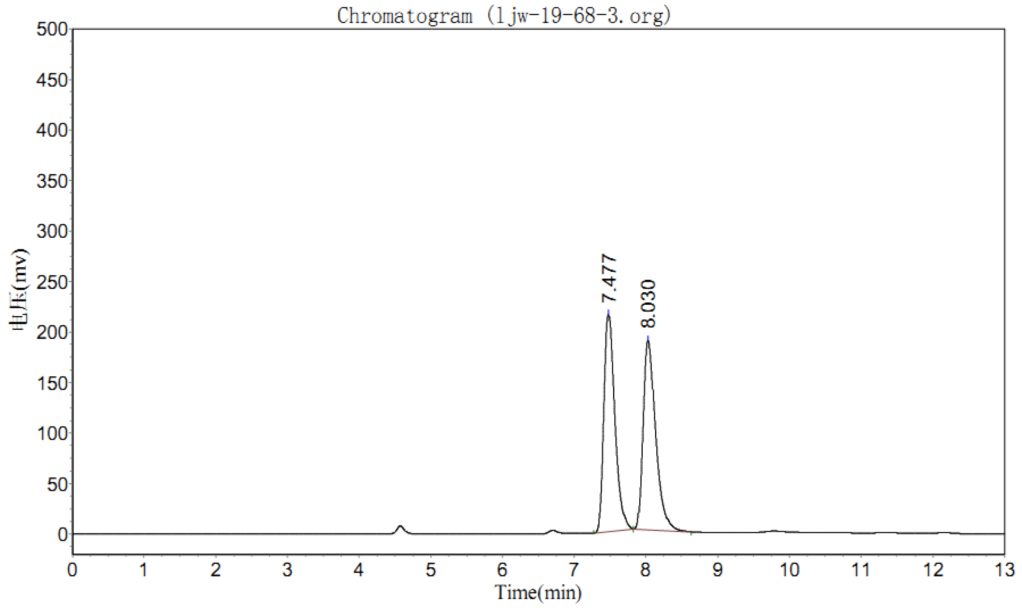

Peak No.	Peak ID	Ret Time	Height	Area	Conc.
1		7.477	214772.047	2283317.000	50.1251
2		8.030	187283.813	2271919.750	49.8749
Total			402055.859	4555236.750	100.0000

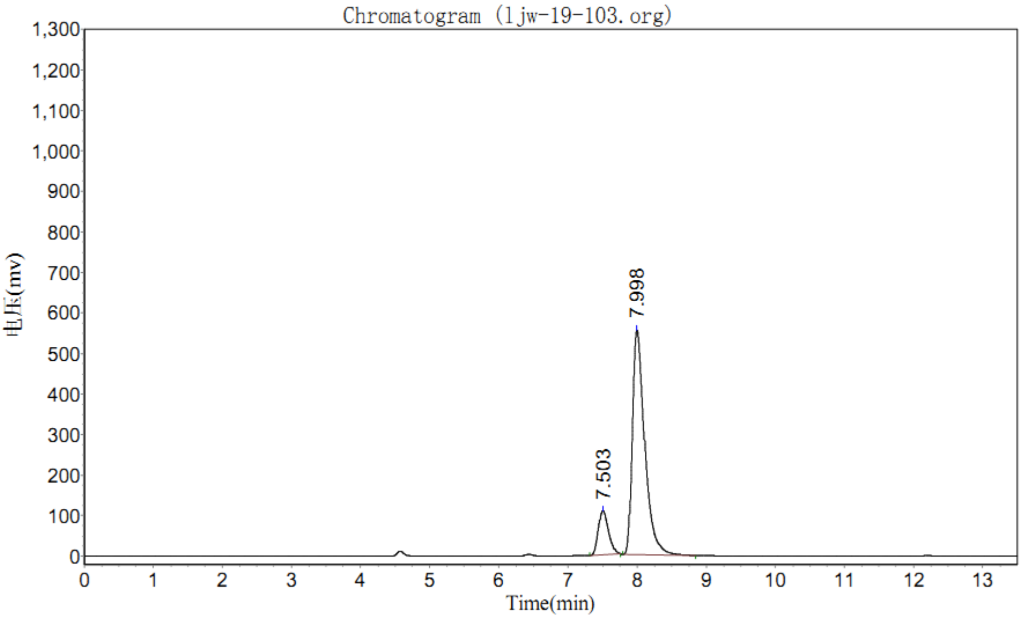

Peak No.	Peak ID	Ret Time	Height	Area	Conc.
1		7.503	108501.422	1118875.875	13.9547
2		7.998	553250.438	6899022.000	86.0453
Total			661751.859	8017897.875	100.0000

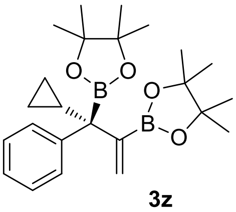

3z

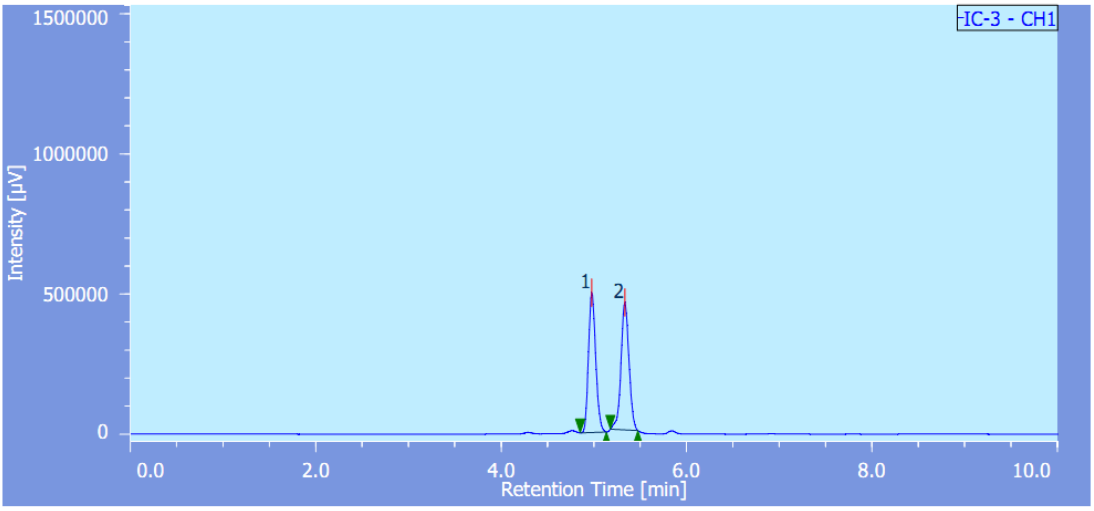

#	Peak Name	CH	tR [min]	Area [µV·sec]	Height [µV]	Area%	Height%	Quantity	NTP	Resolution	Symmetry Factor	Warning
1	Unknown	1	4.975	2700521	498706	49.693	52.288	N/A	19436	2.417	1.202	
2	Unknown	1	5.333	2733858	455060	50.307	47.712	N/A	19044	N/A	0.981	

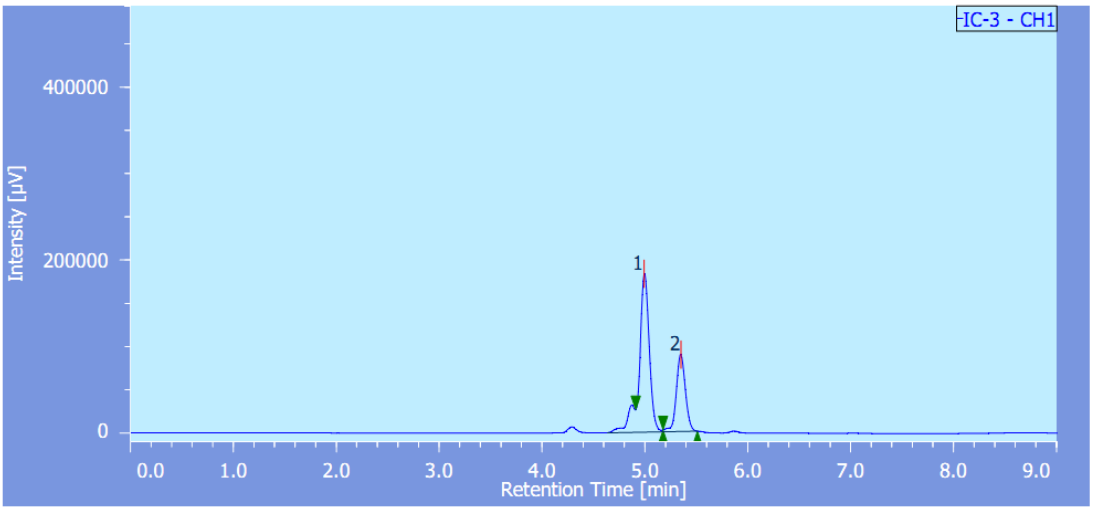

#	Peak Name	CH	tR [min]	Area [µV·sec]	Height [µV]	Area%	Height%	Quantity	NTP	Resolution	Symmetry Factor	Warning
1	Unknown	1	4.992	1112448	182965	67.140	67.309	N/A	15959	2.284	N/A	
2	Unknown	1	5.350	544464	88862	32.860	32.691	N/A	18707	N/A	1.001	

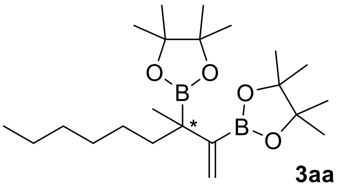

3aa

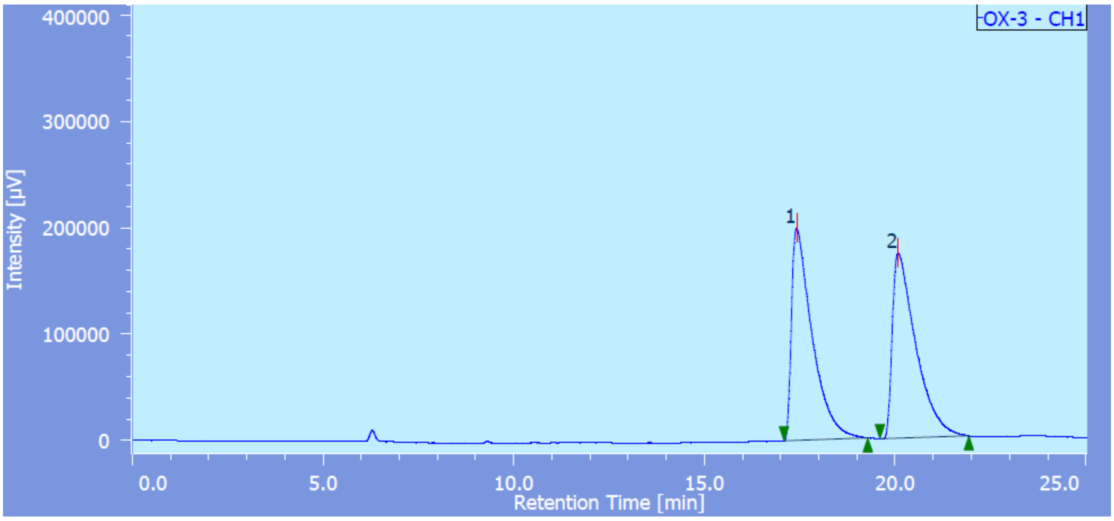

#	Peak Name	CH	tR [min]	Area [μV·sec]	Height [μV]	Area%	Height%	Quantity	NTP	Resolution	Symmetry Factor	Warning
1	Unknown	1	17.417	7508680	199402	50.299	53.428	N/A	5567	2.656	2.688	
2	Unknown	1	20.075	7419554	173817	49.701	46.572	N/A	5593	N/A	2.639	

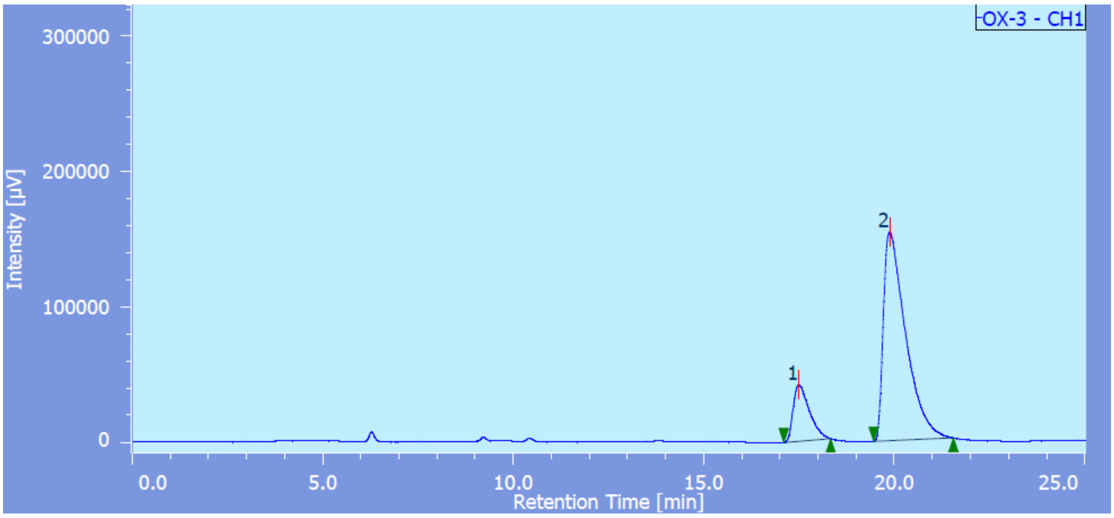

#	Peak Name	CH	tR [min]	Area [μV·sec]	Height [μV]	Area%	Height%	Quantity	NTP	Resolution	Symmetry Factor	Warning
1	Unknown	1	17.492	1238452	41693	16.525	21.271	N/A	8035	2.639	1.688	
2	Unknown	1	19.875	6255811	154312	83.475	78.729	N/A	5950	N/A	2.331	

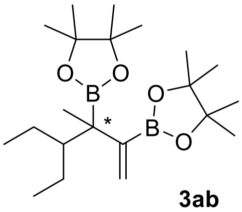

3ab

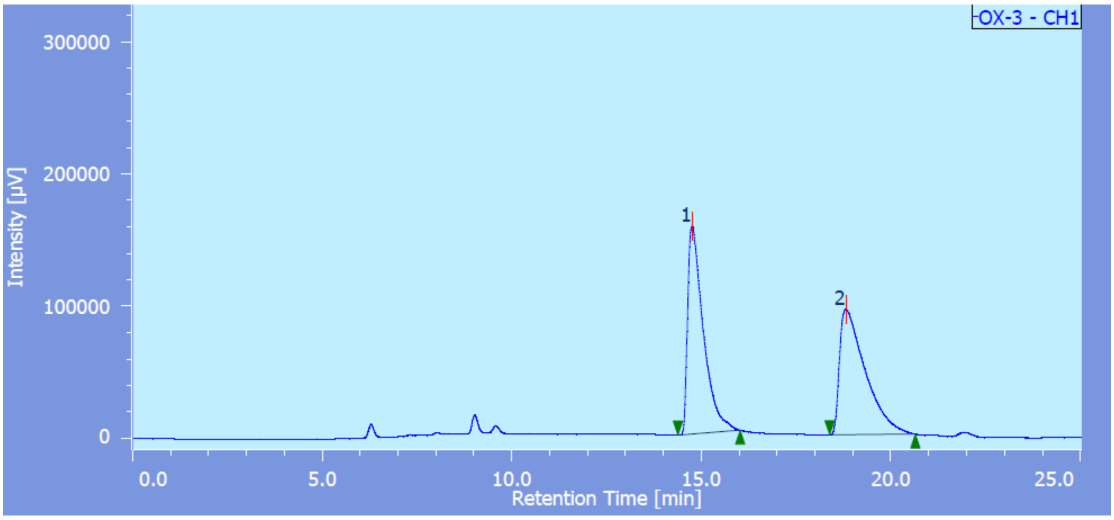

#	Peak Name	CH	tR [min]	Area [µV·sec]	Height [µV]	Area%	Height%	Quantity	NTP	Resolution	Symmetry Factor	Warning
1	Unknown	1	14.758	4507619	157259	50.121	62.374	N/A	6867	4.238	2.444	
2	Unknown	1	18.808	4485784	94863	49.879	37.626	N/A	3904	N/A	2.727	

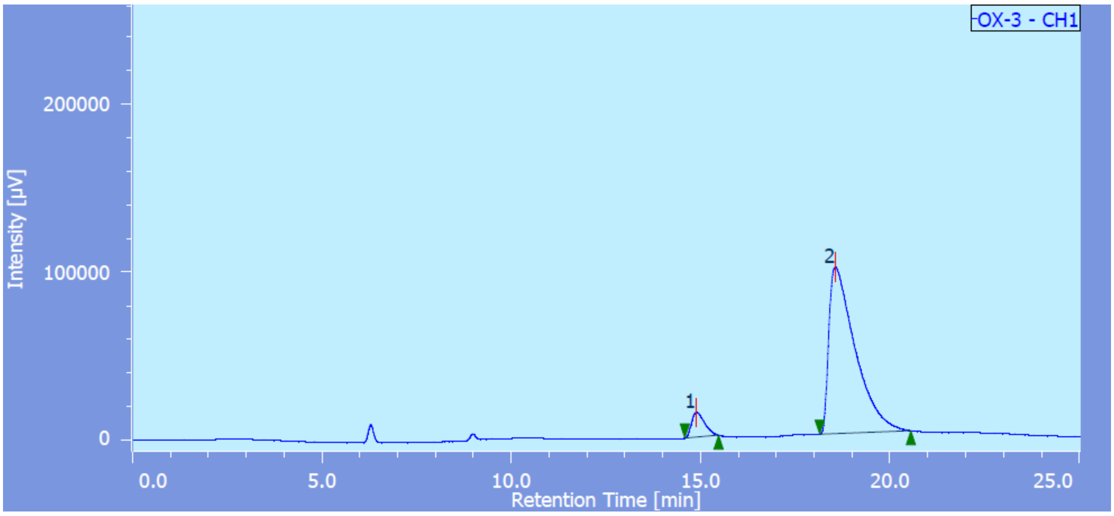

#	Peak Name	CH	tR [min]	Area [µV·sec]	Height [µV]	Area%	Height%	Quantity	NTP	Resolution	Symmetry Factor	Warning
1	Unknown	1	14.892	353827	14805	6.899	13.014	N/A	8650	3.943	1.474	
2	Unknown	1	18.558	4774502	98960	93.101	86.986	N/A	3677	N/A	2.716	

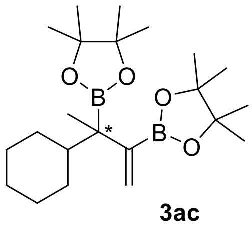

3ac

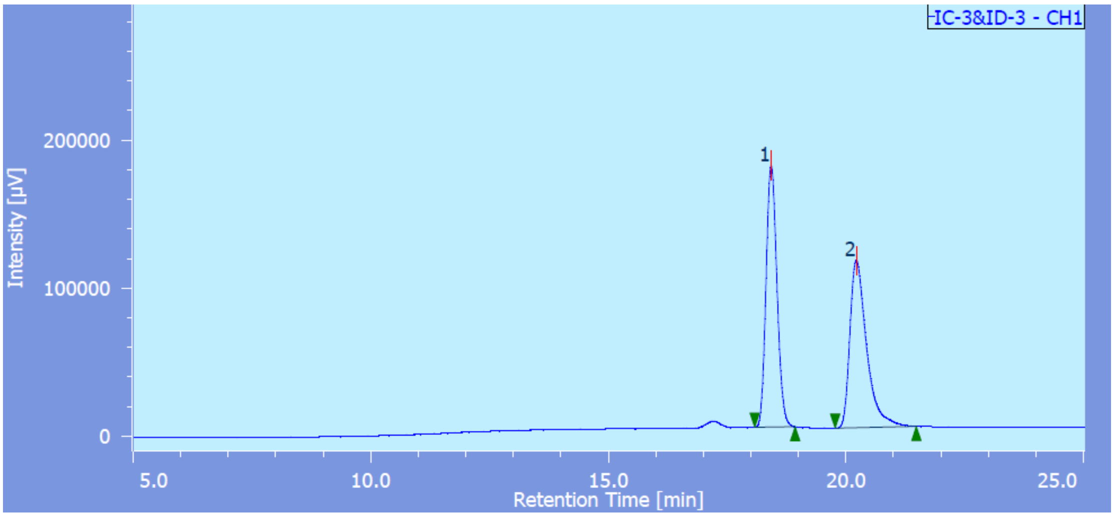

#	Peak Name	CH	tR [min]	Area [µV·sec]	Height [µV]	Area%	Height%	Quantity	NTP	Resolution	Symmetry Factor	Warning
1	Unknown	1	18.425	2810732	176580	50.334	61.030	N/A	30643	3.480	1.140	
2	Unknown	1	20.217	2773478	112753	49.666	38.970	N/A	17496	N/A	1.664	

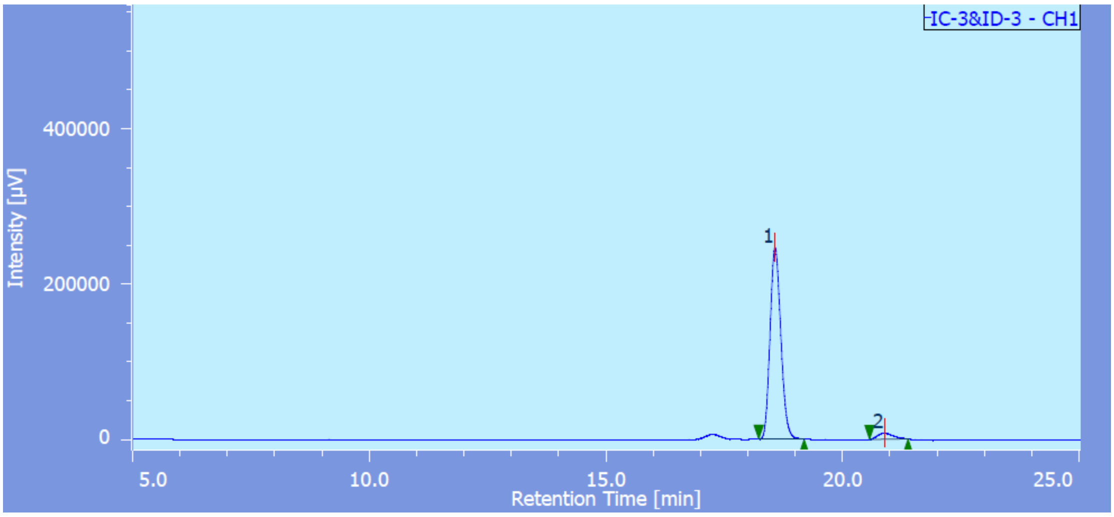

#	Peak Name	CH	tR [min]	Area [µV·sec]	Height [µV]	Area%	Height%	Quantity	NTP	Resolution	Symmetry Factor	Warning
1	Unknown	1	18.567	3890837	246491	95.483	96.853	N/A	31940	4.484	1.183	
2	Unknown	1	20.875	184061	8010	4.517	3.147	N/A	18328	N/A	1.305	

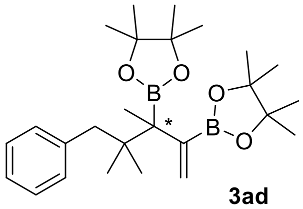

3ad

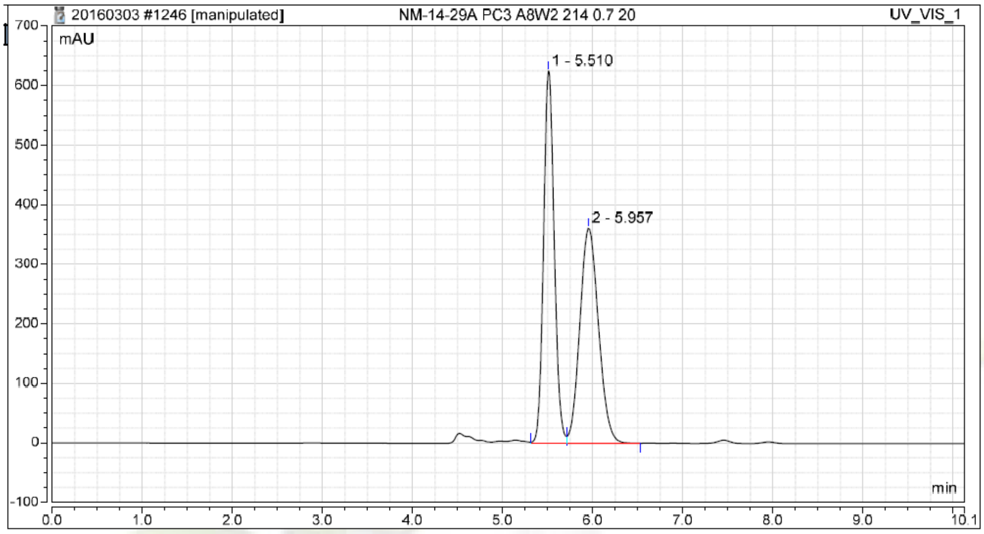

Integration Results				
No.	Retention Time<br>min	Area<br>mAU*min	Height<br>mAU	Relative Area<br>%
1	5.510	85.997	625.825	49.72
2	5.957	86.968	361.879	50.28
**Total:**		**172.965**	**987.704**	**100.00**

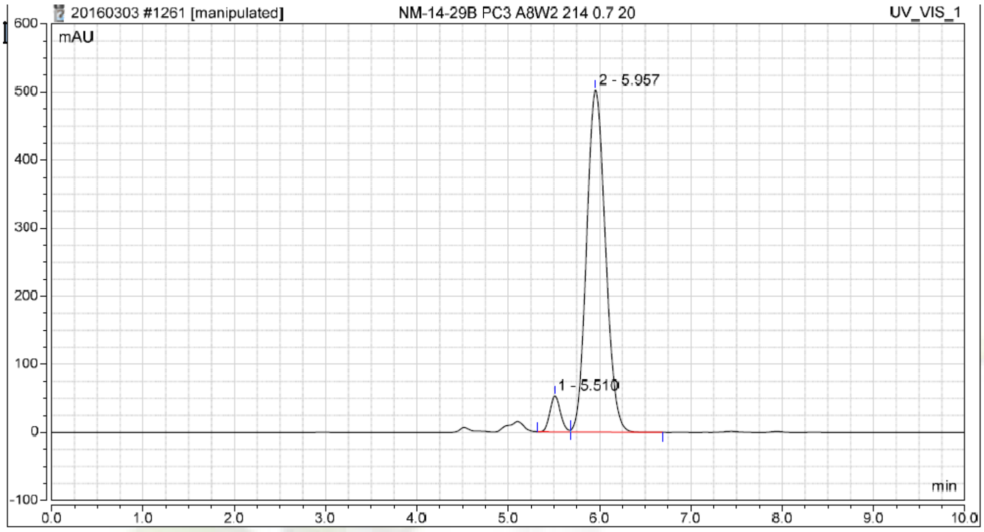

Integration Results				
No.	Retention Time<br>min	Area<br>mAU*min	Height<br>mAU	Relative Area<br>%
1	5.510	7.053	52.993	5.57
2	5.957	119.561	502.619	94.43
**Total:**		**126.614**	**555.612**	**100.00**

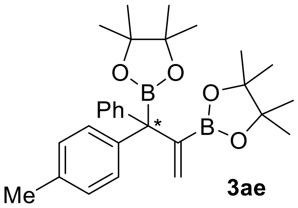

3ae

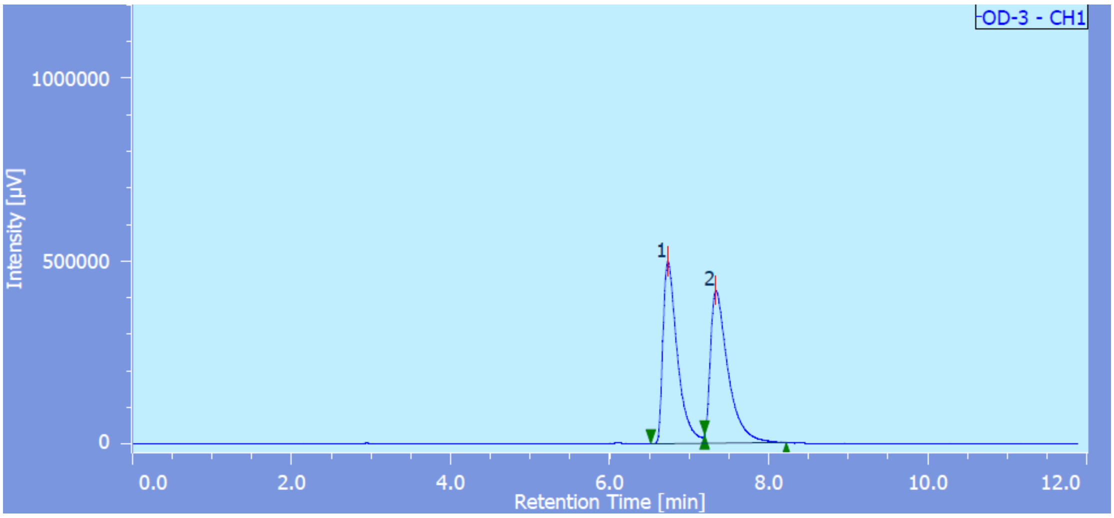

#	Peak Name	CH	tR [min]	Area [µV·sec]	Height [µV]	Area%	Height%	Quantity	NTP	Resolution	Symmetry Factor	Warning
1	Unknown	1	6.733	6281019	498289	49.463	54.430	N/A	7398	1.742	1.937	
2	Unknown	1	7.333	6417316	417185	50.537	45.570	N/A	6038	N/A	2.026	

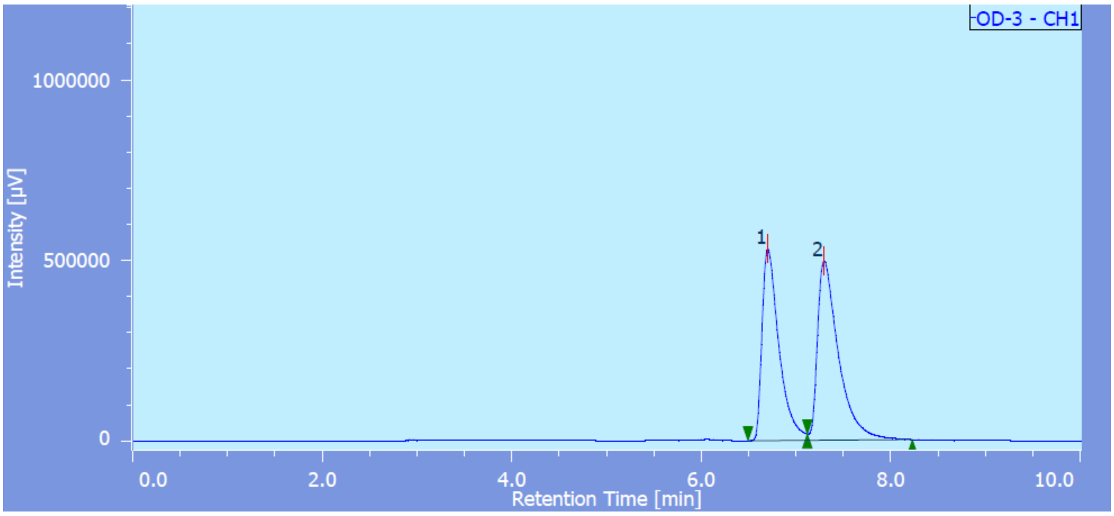

#	Peak Name	CH	tR [min]	Area [µV·sec]	Height [µV]	Area%	Height%	Quantity	NTP	Resolution	Symmetry Factor	Warning
1	Unknown	1	6.700	6576226	530309	46.233	51.669	N/A	7455	1.729	1.995	
2	Unknown	1	7.292	7647919	496045	53.767	48.331	N/A	6025	N/A	2.096	

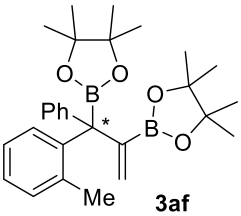

3af

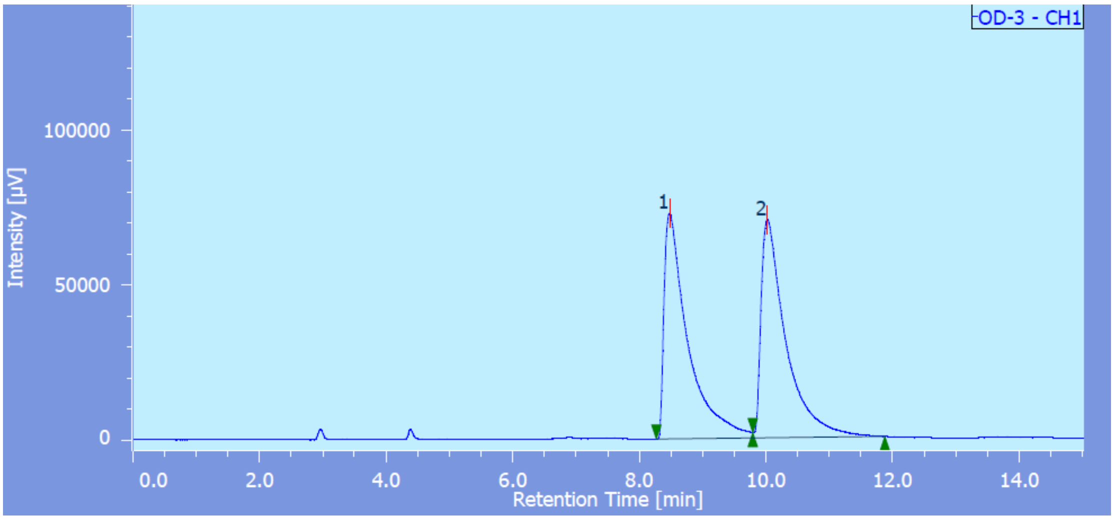

#	Peak Name	CH	tR [min]	Area [µV·sec]	Height [µV]	Area%	Height%	Quantity	NTP	Resolution	Symmetry Factor	Warning
1	Unknown	1	8.475	1891389	72741	49.743	50.868	N/A	3427	2.568	3.950	
2	Unknown	1	10.017	1910931	70260	50.257	49.132	N/A	4113	N/A	3.166	

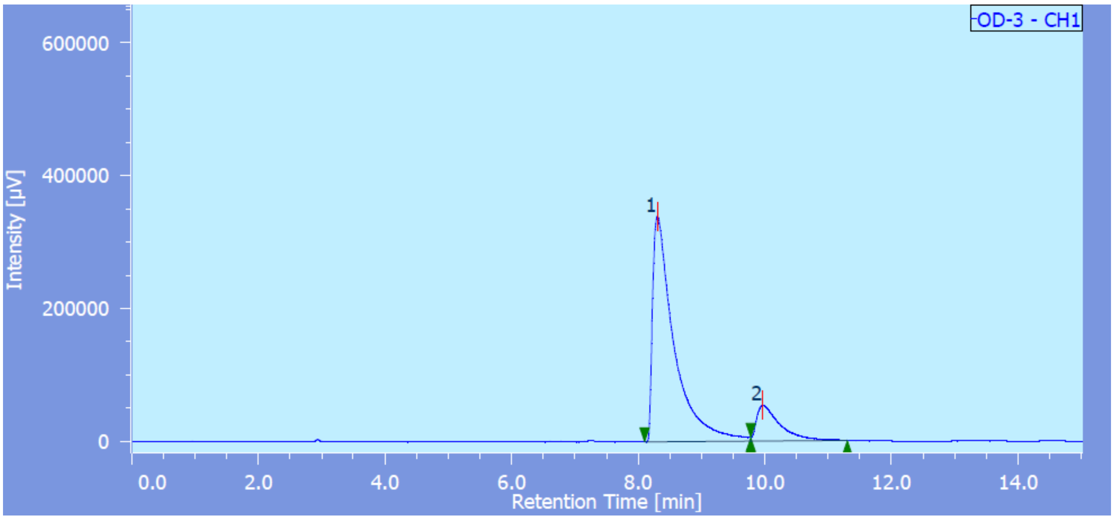

#	Peak Name	CH	tR [min]	Area [µV·sec]	Height [µV]	Area%	Height%	Quantity	NTP	Resolution	Symmetry Factor	Warning
1	Unknown	1	8.300	7997920	339131	85.204	86.356	N/A	3989	2.934	3.934	
2	Unknown	1	9.967	1388883	53581	14.796	13.644	N/A	4223	N/A	N/A	

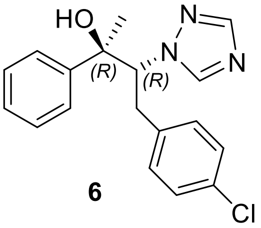

6

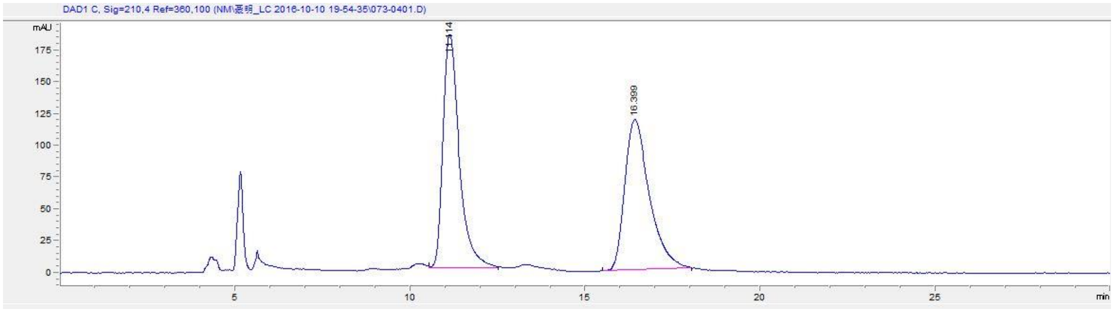

#	时间	峰面积	峰高	峰宽	对称因子	峰面积 %
1	11.114	6042.4	184	0.4871	0.635	50.908
2	16.399	5826.9	118.2	0.609	0.637	49.092

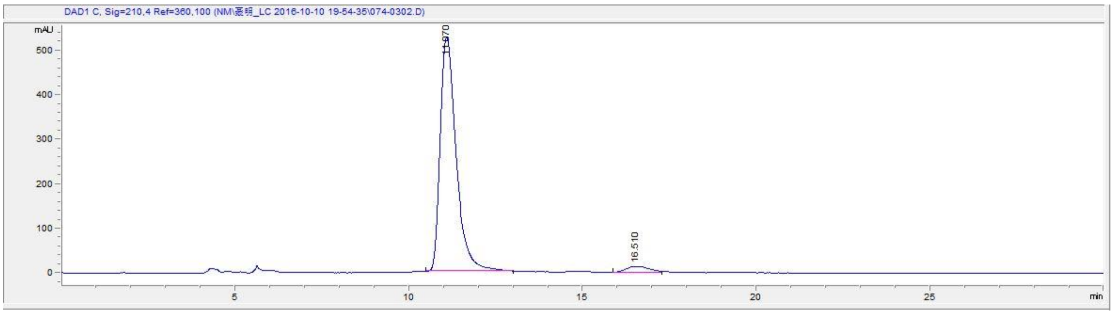

#	时间	峰面积	峰高	峰宽	对称因子	峰面积 %
1	11.07	16775.9	526.7	0.4876	0.605	96.197
2	16.51	663.2	14	0.5593	0.683	3.803

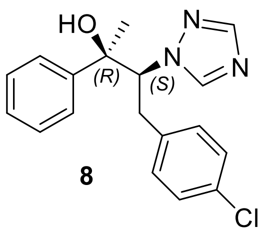

8

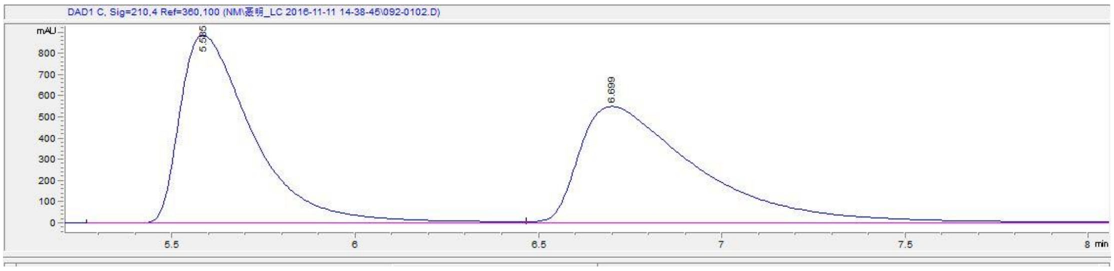

#	时间	峰面积	峰高	峰宽	对称因子	峰面积 %
1	5.585	12260.3	887.9	0.2091	0.422	49.460
2	6.699	12528	549.8	0.3338	0.338	50.540

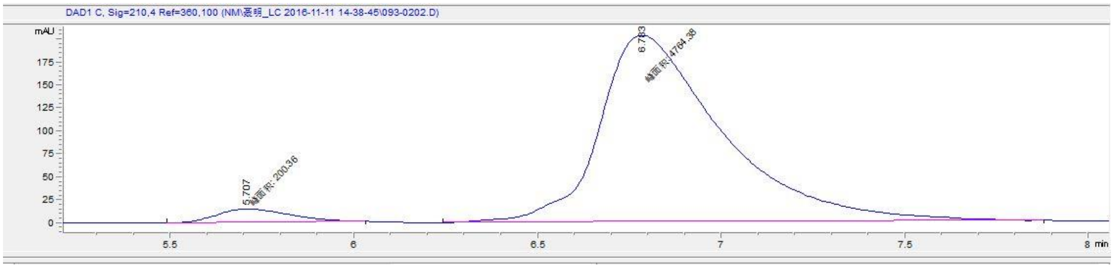

#	时间	峰面积	峰高	峰宽	对称因子	峰面积 %
1	5.707	200.4	14.5	0.2298	0.671	4.036
2	6.783	4764.4	203.1	0.391	0.518	95.964
